# The Arabidopsis miR396 mediates pathogen-associated molecular pattern-triggered immune responses against fungal pathogens

**DOI:** 10.1038/srep44898

**Published:** 2017-03-23

**Authors:** Mauricio Soto-Suárez, Patricia Baldrich, Detlef Weigel, Ignacio Rubio-Somoza, Blanca San Segundo

**Affiliations:** 1 Centre for Research in Agricultural Genomics (CRAG) CSIC-IRTA-UAB-UB. Edifici CRAG, Campus UAB, Bellaterra (Cerdanyola del Vallés), 08193 Barcelona, Spain; 2 Department of Molecular Biology, Max Planck Institute for Developmental Biology, 72076 Tübingen, Germany

## Abstract

MicroRNAs (miRNAs) play a pivotal role in regulating gene expression during plant development. Although a substantial fraction of plant miRNAs has proven responsive to pathogen infection, their role in disease resistance remains largely unknown, especially during fungal infections. In this study, we screened *Arabidopsis thaliana* lines in which miRNA activity has been reduced using artificial miRNA target mimics (*MIM* lines) for their response to fungal pathogens. Reduced activity of miR396 (*MIM396* plants) was found to confer broad resistance to necrotrophic and hemibiotrophic fungal pathogens. MiR396 levels gradually decreased during fungal infection, thus, enabling its GRF (*GROWTH-REGULATING FACTOR*) transcription factor target genes to trigger host reprogramming. Pathogen resistance in *MIM396* plants is based on a superactivation of defense responses consistent with a priming event during pathogen infection. Notably, low levels of miR396 are not translated in developmental defects in absence of pathogen challenge. Our findings support a role of miR396 in regulating plant immunity, and broaden our knowledge about the molecular players and processes that sustain defense priming. That miR396 modulates innate immunity without growth costs also suggests fine-tuning of miR396 levels as an effective biotechnological means for protection against pathogen infection.

Plants are constantly exposed to a variety of potentially pathogenic microorganisms. To prevent infections, plants have evolved a multi-tiered immune system^1^,^2^. A first layer is initiated by the recognition of conserved pathogen-associated molecular patterns (PAMPs, previously known as elicitors) through pattern recognition receptors (PRR) at the cell surface, a phenomenon referred to as PAMP-triggered immunity (PTI) or basal defense^2^. PTI components induce both physical and chemical defenses, such as callose and reactive oxygen species (ROS)^2^,^3^. To counteract this innate defense, pathogens deploy effectors that suppress PTI. Effectors, or the biochemical consequences of their activity, are in turn recognized by Resistance (R) proteins, which confer Effector-Triggered Immunity (ETI, formerly known as gene-for-gene resistance), an enhanced type of defense. ETI and PTI are both associated with qualitatively similar, but quantitatively and kinetically different transcriptional reprogramming in the host^4^,^5^.

Timing and intensity of host reprogramming during both PTI and ETI must be tightly regulated to best tailor the immune response to the faced threat. Dynamic reprogramming is orchestrated by dedicated genetic programs in which small RNAs, such as microRNAs (miRNAs), might have a pivotal role. miRNAs act at the post-transcriptional level, negatively controlling gene expression by triggering the cleavage or translational repression of their target transcripts^6^,^7^. The best characterized roles of plant miRNAs are in development, including leaf, flower and root development, and in responses to abiotic stresses^8^,^9^,^10^,^11^. The first evidence for miRNAs affecting pathogen defense came from *A. thaliana*, where a fragment of bacterial flagellin, flg22, causes an increase in miR393, a negative regulator of TIR1/AFB auxin receptors. The miR393-mediated repression of auxin signaling enhances resistance to bacterial pathogens^12^. A more direct role of miRNAs in plant defense is the targeting of transcripts of the major class of *R* genes, NLR (nucleotide binding site, leucine rich repeat) genes^13^,^14^,^15^,^16^ or the atypical ARLPK (receptor-like pseudokinase) genes that are known to be important for plant defense^17^.

Many other plant miRNAs have been shown to respond to pathogen challenge^18^,^19^, suggesting a broader involvement of miRNAs in defense. Here, we have systematically investigated the potential role of different miRNAs in disease resistance with *A. thaliana* lines in which activity of specific miRNA families is suppressed with miRNA target mimics^20^. Particularly interesting effects were seen in *MIM396* plants, in which miR396 activity is reduced. We show that *MIM396* plants are more resistant to infection by both necrotrophic fungi, *Plectosphaerella cucumerina* and *Botrytis cinerea*, and hemibiotrophic fungi, *Fusarium oxysporum* f. sp. *conglutinans* and *Colletotrichum higginsianum*. The limiting role of miR396 in these responses was confirmed with plants that overexpress *MIR396*, which are more susceptible to fungal infection. The evolutionary conserved miR396 targets a set of transcription factors belonging to the *GROWTH-REGULATING FACTOR (GRF*) family in many plant species, including important crops like rice, maize or tomato^21^,^22^,^23^. Fungal perception impinges on the activity of *MIR396* promoters leading to a gradual decline in mature miR396 levels and a concomitant increase of miR396 target expression during the normal course of PTI. MiR396 targets can regulate growth and stress responses^24^,^25^,^26^,^27^,^28^, *MIM396* plants grow and develop normally. This result is in agreement with the minor changes observed in the global transcriptome in non-pathogen challenged *MIM396* plants. The enhanced H_2_O_2_ accumulation, callose deposition and transcriptional reprogramming after *P. cucumerina* treatment suggest a potentiated induction of defense responses in *MIM396* plants, or “sensitization” that confers an improved reaction to pathogen infection. Such superactivation of defense responses is in agreement with a priming event^29^. Collectively, our results suggest that reduced levels of miR396 improve resistance to fungal pathogens through potentiation of defense mechanisms without major penalties for host growth and development. Despite priming being a long recognized phenomenon, little is known about the molecular mechanisms underlying such sensitization. This study broadens our knowledge about the molecular players participating in defense priming by adding miR396 and its transcription factor targets to the palette of elements involved.

## Results

### Increased resistance to fungal infection in *MIM396* plants

To identify miRNAs contributing to disease resistance in *A. thaliana*, we examined a collection of *MIM* lines designed to specifically interfere with the activity of distinct miRNAs^30^ (Table S1). Infection experiments were initially carried out with the necrotrophic fungus *P. cucumerina* (previously known as *Fusarium tabacinum*; anamorph *Plectosporium tabacinum*)^31^ which causes sudden death and blight in many dicot crops, and which also infects *A. thaliana*^32^,^33^,^34^,^35^.

Five-day-old seedlings and 3-week-old plants were challenged with *P. cucumerina*. Col-0 wild-type and transgenic plants containing the empty vector as well as *agb1.2* mutants, lacking activity of the heterotrimeric G-protein β-subunit and having enhanced susceptibility to *P. cucumerina*^33^, served as controls (Supplementary Table S1). At 4 days post-inoculation (dpi), a few brown spots were visible on cotyledons of wild-type and vector control seedlings, while *agb1.2* cotyledons were much more densely covered with brown spots (Supplementary Figure S1). Most *MIM* lines tested were similar to the controls, with the notable exception of *MIM396*, which had much reduced symptoms (Supplementary Figure S1).

**Figure 1:**
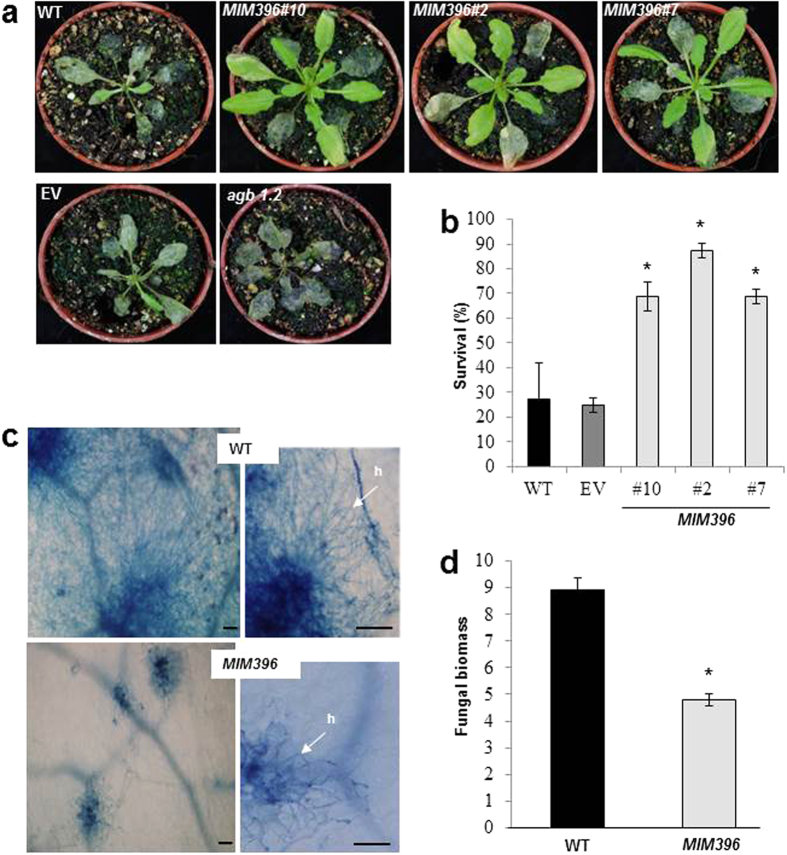
Reduced *P. cucumerina* infection of *MIM396* plants. (**a**) Whole-plant images of 3-week-old plants, 12 dpi after inoculation with *P. cucumerina* spores (1.4 × 10^6^ spores mL^−1^). *agb1.2* plants with enhanced susceptibility to this pathogen^33^ were used as positive control. (**b**) Survival rates of *MIM396* and control plants at 21 dpi. (**c**) Leaf colonization by *P. cucumerina*, visualized by Trypan blue staining. h, hyphae. Bars represent 50 μm. (**d**) Relative quantification of *P. cucumerina* DNA in wild-type (Col-0) and *MIM396* plants at 3 days post-inoculation using specific primers of *P. cucumerina β-tubulin*^35^. Values are fungal DNA levels normalized against the Arabidopsis *UBIQUITIN21* gene (At5g25760). Results are from one out of three independent experiments (12 plants/genotype), which gave similar results. Statistical significance in b and d was determined by ANOVA (*P ≤ 0.05). Histograms show the mean ± SD.

We further confirmed the enhanced resistance in *MIM396* adult plants with 3-week old plants that were inoculated with *P. cucumerina* spores (Fig. 1a; Supplementary Figure S2). While three quarters of *MIM396* plants had survived at 21 dpi, only a quarter of wild-type or empty vector controls were able to overcome infection (Fig. 1b). Trypan blue staining of infected leaves further confirmed limited fungal growth in *MIM396* plants (Fig. 1c), which was confirmed by quantitative PCR (qPCR) measurement of fungal DNA, an indicator of fungal biomass in host tissues (Fig. 1d).

**Figure 2:**
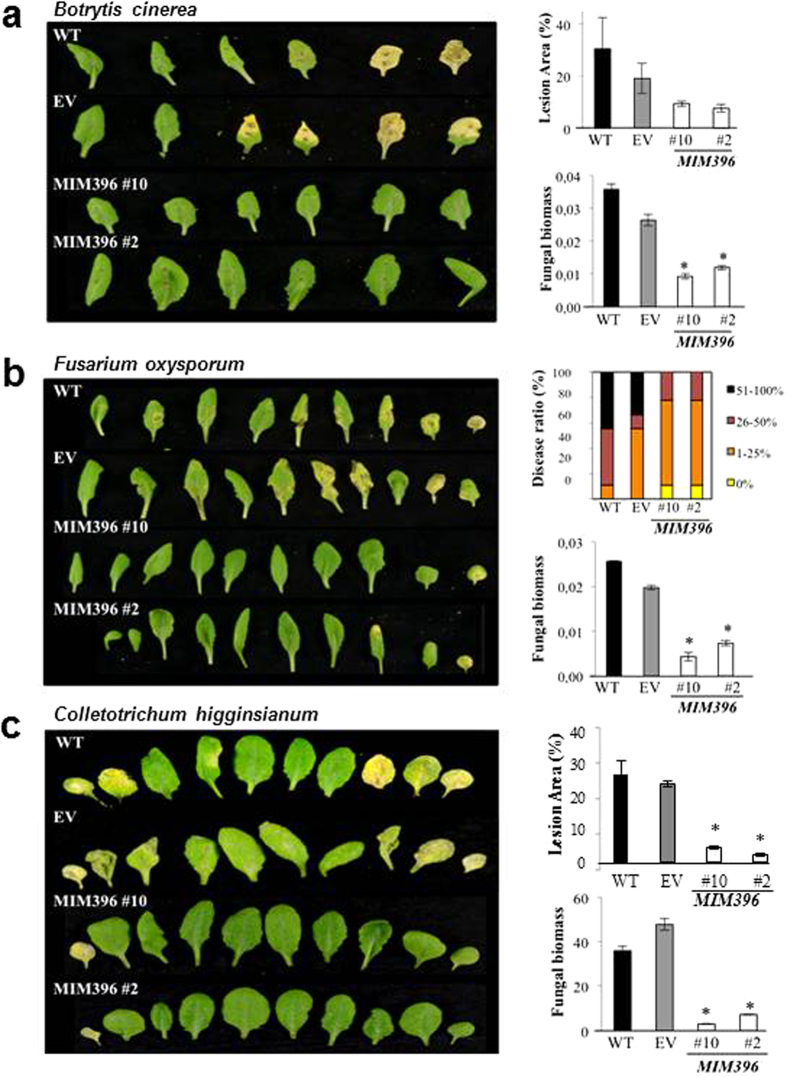
Reduced *B. cinerea*, FOC, and *C. higginsianum* infection of *MIM396* plants. Three-week-old plants (3 independent *MIM396* lines, 3 biological replicates, each with at least 12 plants per genotype) were used. For infection with *B. cinerea* and *C. higginsianum* (**a**,**c**), the plants were spray-inoculated with a spore suspension at the appropriate concentration. Inoculation with *FOC* (**b**) was carried out by applying the fungal spores to soil (**b**). (**a**) Leaves after inoculation with *B. cinerea* (2 × 10^6^ spores mL^−1^; 2 inoculations per leaf, and two leaves per plant). Lesion area was measured at 7 dpi, and *B. cinerea* DNA at 3 dpi (right panels). (**b**) Plants 12 dpi after inoculation with *FOC* (10^6^ spores mL^−1^). Note prominent chlorosis in infected controls, culminating in eventual leaf curling, yellowing and necrosis. *MIM396* lines exhibited much milder symptoms. Disease symptoms were determined 12 dpi, and fungal DNA 3 dpi (right panel). Disease ratio was calculated as the percentage of chlorotic leaves per plant. (**c**) Plants 10 dpi after inoculation with *C. higginsianum* (4 × 10^6^ spores mL^−1^). Diseased leaf area was quantified 10 dpi, and fungal DNA 3 dpi (right panels; values are fold fungal DNA levels relative to the Arabidopsis *Ubiquitin21* gene. Histograms show the mean ± SD (*P ≤ 0.05, determined by ANOVA). WT, wild type. EV, empty vector control.

To ascertain whether suppression of miR396 activity broadly confers resistance to other fungal pathogens, we challenged *MIM396* plants with *B. cinerea*, a necrotrophic fungus that causes grey mold in a wide range of hosts^36^ as well as *F. oxysporum* f. sp. *conglutinans* (FOC) and *C. higginsianum*, two hemibiotrophic fungi^37^,^38^. *MIM396* plants were visibly more resistant to infection by *B. cinerea*, FOC or *C. higginsianum* than the controls (Fig. 2a–c, left panels), which was confirmed by quantifying the leaf area with lesions and the amount of fungal DNA (Fig. 2a–c, right panels). Collectively, these results strongly support that impairment of miR396 activity enhances resistance to infection by fungal pathogens with different lifestyles.

### Enhanced immune responses to *P. cucumerina* infection in *MIM396* plants

**Figure 3:**
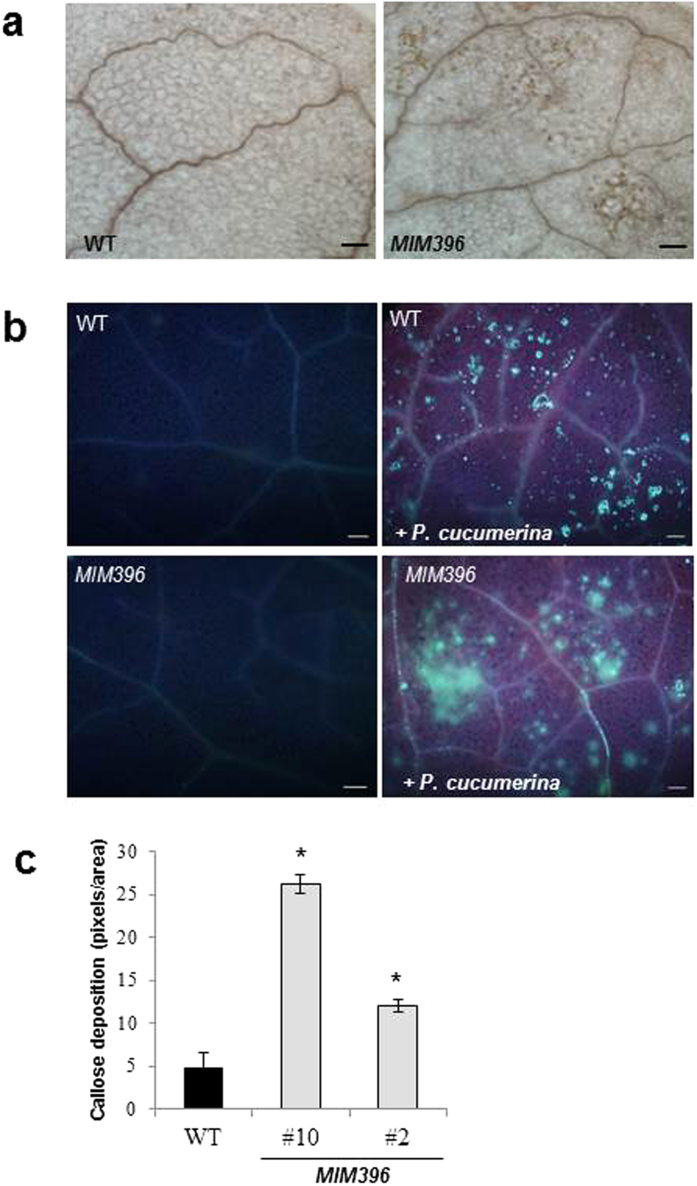
H_2_O_2_ accumulation and callose deposition in *P. cucumerina*-infected leaves. (**a**) DAB staining of leaves to visualize H_2_O_2_ accumulation in wild-type and *MIM396* plants prior inoculation and 24 hpi after inoculation with *P. cucumerina* spores. Patches of brown precipitate of oxidized DAB are prominent in *MIM396*. Bars represent 50 μm. (**b**) UV micrographs of aniline blue stained leaves to visualize callose 24 hpi. (**c**) Callose deposition was calculated as arbitrary units by quantifying the number of yellow pixels per million on digital micrographs of infected leaves. Statistical significance was determined by ANOVA (*P ≤ 0.05). Bars represent mean ± SD (n = 3 biological replicates, 3 independent lines; 9 plants per independent line, and 3 leaves per plant).

Two of the earliest plant responses to pathogen infection are a controlled burst of reactive oxygen species (ROS)^39^, and deposition of callose at the infection site^40^,^41^. Histochemistry showed that *MIM396* accumulated both more H_2_O_2_ and callose after challenge with *P. cucumerina* (Fig. 3a–c), or after treatment with a crude preparation of elicitors obtained by autoclaving and sonicating *P. cucumerina* mycelium (Supplementary Fig. S3). Importantly, *MIM396* plants accumulated callose only after inoculation with *P. cucumerina* (or elicitor application), indicating that the immune response is not constitutive, but stronger.

### Increased susceptibility to *P. cucumerina* caused by *MIR396B* overexpression

**Figure 4:**
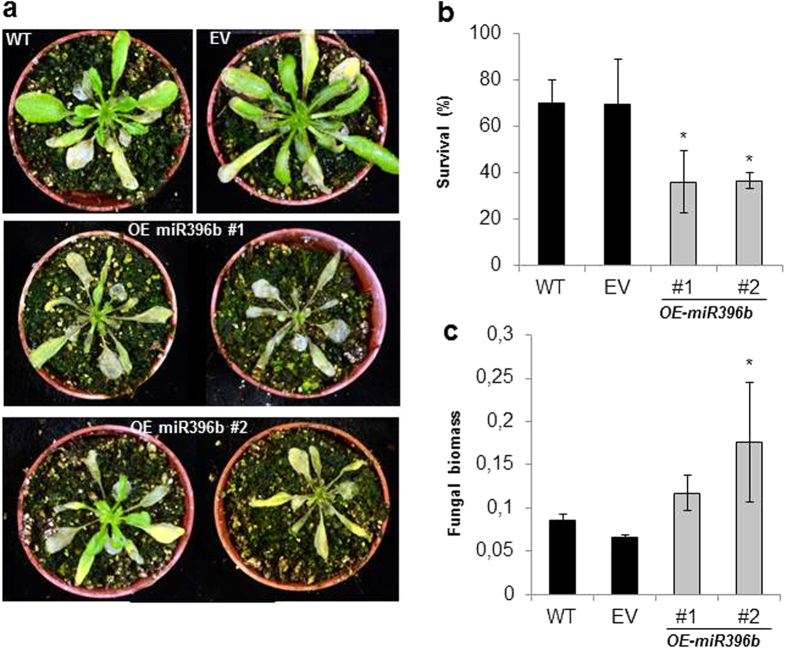
Increased *P. cucumerina* infection of *35Sprom::MIR396B* plants. (**a**) Phenotype of *35Sprom::MIR396B* plants at 10 dpi with *P. cucumerina* spores. Three independent homozygous *35Sprom::MIR396B* and control plants (EV, empty vector, WT and wild-type plants) were analyzed. Three independent experiments were carried out each with at least 12 plants per line. Results for two lines are shown; similar results were obtained for the third *35Sprom::MIR396B* line. (**b**) Survival of *35Sprom::MIR396B* plants at 12 dpi with *P. cucumerina*. Statistical significance was determined by ANOVA (*P ≤ 0.05, overexpressor line *vs* wild-type or empty vector). (**c**) Fungal biomass at 3dpi with *P. cucumerina* spores as determined by RT-qPCR (values are fold fungal DNA levels relative to the Arabidopsis *UBIQUITIN21* gene).

To determine whether miR396 might have an instructive role in immunity, we overexpressed *MIR396B* under the control of the *CaMV35S* promoter. Plants over-accumulating miR396 have been described to show a gradient of phenotypic manifestation that correlates with the reduction of its GRF targets^24^. To avoid that developmental defects caused by high levels of miR396 expression could confound our observations, we assayed *35Sprom::MIR396B* plants that despite accumulating higher levels of miR396, appeared otherwise normal under our growth conditions (Supplementary Fig. S4). *35Sprom::MIR396B* plants displayed enhanced susceptibility to infection by *P. cucumerina* (Fig. 4a). Survival rates of *35Sprom::MIR396B* at 10 days after fungal inoculation were lower than for wild-type and empty vector control plants (Fig. 4b), whereas fungal biomass increased in *MIR396B* overexpressor plants compared to control plants (Fig. 4c).

### miR396 and *GRF* expression during fungal infection

**Figure 5:**
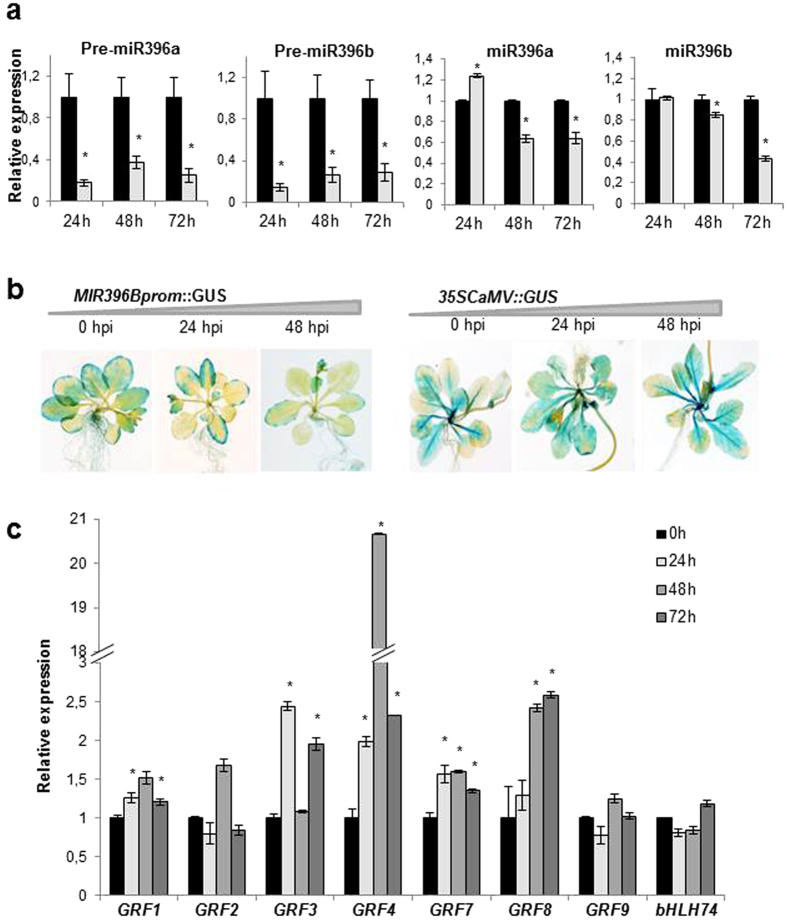
Expression of miR396 and miR396 targets upon inoculation with *P. cucumerina* spores. (**a**) Accumulation of pre-miR396 and mature miR396 sequences was determined by RT-qPCR analysis in wild type plants that have been inoculated with *P. cucumerina* spores. The expression level in mock-inoculated plants was set to 1.0. The values represent changes in the accumulation of miR396a and miR396b (light and dark grey bars, respectively) at the indicated times after inoculation (*P ≤ 0.05; ANOVA test, *P. cucumerina*-inoculated vs. mock-inoculated). (**b**) Histochemical analysis of GUS activity in *MIR396Bprom::GUS* plants 24 and 48 hpi with *P. cucumerina* (10^6^ spores mL^−1^). *35Sprom::GUS* plants served as controls. (**c**) Expression of miR396 targets in response to inoculation with *P. cucumerina*. For each gene, the values represent changes in transcript abundance after inoculation with *P. cucumerina* spores compared with the time zero. Bars with asterisks are statistically different (*P ≤ 0.05; ANOVA test). Histograms show the mean ± SD of one out of 3 biological replicates with similar results, each replicate with 12 plants per genotype.

The apparently negative effect of miR396 on resistance to fungal pathogens prompted us to ask whether miR396 and miR396-targets show changes in their accumulation during the normal host response to infection. In *A. thaliana*, miR396 is encoded by two loci, *MIR396A* and *MIR396B*. The accumulation of precursor transcripts and mature sequences for each miR396 family member was determined at different times after inoculation with *P. cucumerina*. There was a general and clear reduction in the accumulation of both precursors, pre-miR396a and pre-miR396b, at 24, 48 and 72 hpi (hours post-inoculation), which was paralleled by a delayed decrease in the accumulation of mature miR396a and miR396b (48 and 72 hpi) (Fig. 5a). A similar trend was observed after treatment with fungal elicitors (Supplementary Figure S5).

Next, we investigated whether the reduced pre-miR396 levels during infection were due to a reduced activity of the *MIR396* promoter. The *MIR396A* and *MIR396B* promoters have similar spatial expression patterns^42^,^43^,^44^. *MIR396Bprom::GUS* plants inoculated with *P. cucumerina* spores showed a specific gradual decrease in the activity of the *MIR396B* promoter (Fig. 5b), indicating that fungal infection leads to transcriptional repression.

MiR396 negatively regulates a sub-set of transcription factor genes, mainly from the *GRF* family and with important roles in development and stress responses^42^,^45^,^46^,^47^. In *A. thaliana*, miR396 additionally targets the *bHLH74* transcription factor gene^43^. Consistent with the gradual miR396 down-regulation observed during infection, most of the miR396-targeted *GRF* genes showed increased expression after inoculation with *P. cucumerina*, albeit with different kinetics and magnitude (Fig. 5c).

### Transcriptome changes in *MIM396* plants

To gain further insights into the molecular mechanisms underlying miR396-dependent susceptibility to pathogen infection, we compared pathogen-induced transcriptome changes in rosettes of 3 week-old wild-type and *MIM396* plants using microarrays. In mock-inoculated plants, we found only 20 genes at a significance threshold of 0.05 (Holm’s correction) and twofold change. Most of these genes were down-regulated in *MIM396* (Supplementary Table S2), including *EDS1 (ENHANCED DISEASE SUSCEPTIBILITY1*, At3g48080) and a TIR-NBS-LRR resistance gene homolog of unknown function (At5g45000). Thus, in the absence of pathogen challenges, reduced miR396 activity has only minimal effects on whole-rosette transcriptomes. Microarray analysis did not reveal any change in the expression of *GRF* genes. However, RT-qPCR analysis using gene-specific primers flanking the miR396 cleavage site confirmed up-regulation of most of the miR396-targeted genes in *MIM396* plants (Supplementary Figure S6). Discrepancies observed in *GRF* expression between microarray and RT-qPCR analysis can be explained by the stability of the resulting 3′ fragments from miRNA-targeted transcripts after miRNA-mediated cleavage (microarray probes are typically designed to recognize 3′ regions of mRNAs distantly located from the miRNA cleavage site). This interpretation is consistent with previous reports describing stability of 3′ cleavage products of miRNA targets and the development of more sensitive approaches to identify and quantify miRNA-triggered transcript cleavage such as degradome analysis^6^,^48^,^49^.

**Figure 6:**
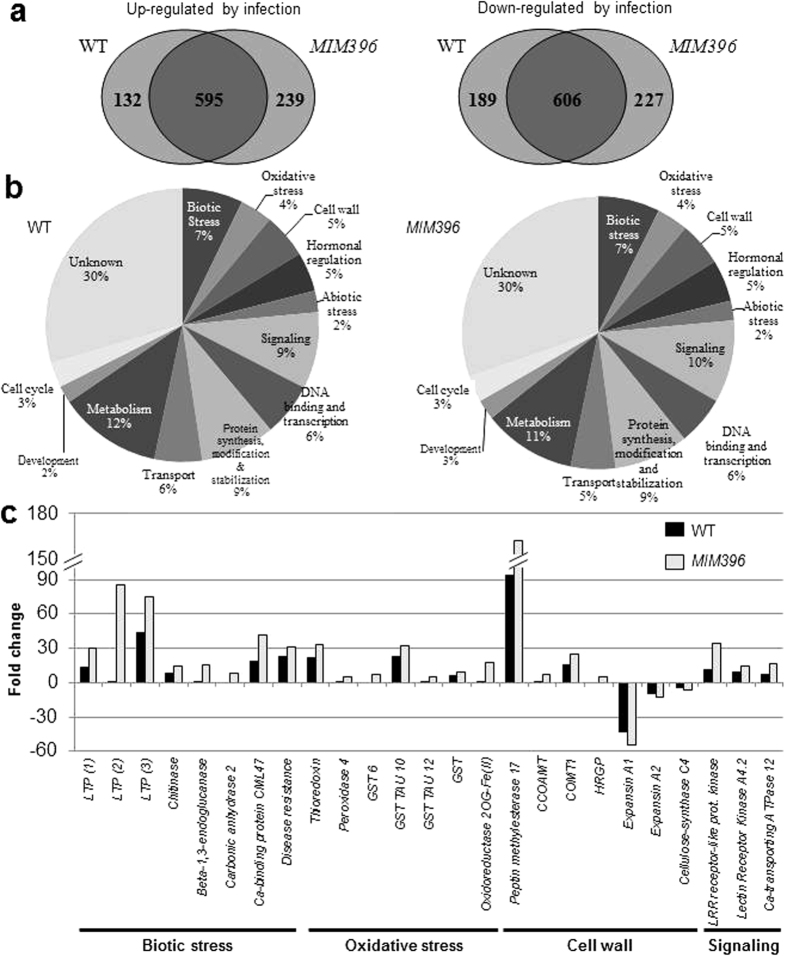
Comparison of wild-type and *MIM396* transcriptomes in response to *P. cucumerina* infection. (**a**) Comparison of induced and repressed genes 72 hpi (fold change ≥ 2, P ≤ 0.05), as determined by microarray analysis. (**b**) Comparison of functional categories of differentially expressed genes. (**c**) Expression of selected defense-related genes. Fold-induction or repression of gene expression (inoculated vs. non-inoculated) is shown.

*P. cucumerina* inoculation triggers changes in the expression of 1522 genes in wild-type plants 72 hours after inoculation, a time of infection in which the two miR396 family members accumulated at a low level, and 5 out of the 8 miR396-targeted genes were up-regulated (e.g. *GRF1, GRF3, GRF4, GRF7*, and *GRF8* (Fig. 5c). By this time, *GRF2, GRF9* and *bHLH74* expression was not significantly affected by fungal infection. Of the 1522 genes that are differentially expressed in wild-type plants, 727 genes were induced and 795 repressed (Fig. 6a, left panel; Supplementary Table S3). Gene Ontology (GO) classification indicated that many defense and stress response genes were affected (Fig. 6b; Supplementary Table S3). Similar numbers of genes were induced and repressed in *MIM396* plants challenged by *P. cucumerina*, with 834 up- and 833 down-regulated genes. Most of the changed genes were shared between wild-type and *MIM396* plants: 595 induced and 606 repressed genes (Fig. 6a,b; Supplementary Tables S3 and S4). Microarray analyses were validated by RT-qPCR (Supplementary Figures S7 and S8). Transcriptional reprogramming in wild-type and *MIM396* plants upon pathogen perception led to changes in the expression of i) genes encoding members of different *PR (PATHOGENESIS-RELATED*) families, ii) disease resistance gene, iii) genes encoding enzymes that function in cellular protection against oxidative stress and toxic compounds, iv) genes related to hormone-mediated defensive mechanisms, and v) genes involved in cell wall architecture and modification. We also noticed that many activated or repressed genes in wild-type plants responded more strongly in *MIM396* plants, i.e., were activated or repressed to a greater extent than in wild-type plants (Fig. 6c; Supplementary Table S5). Together, these observations suggest that the immune response in *MIM396* plants was not qualitatively different from wild-type plants, but more robust. The observed superactivation of defense responses in *MIM396* plants is reminiscent of defense priming, a phenomenon associated with different forms of induced resistance in which plants react to biotic stress with faster and/or stronger activation of defense^29^.

**Figure 7:**
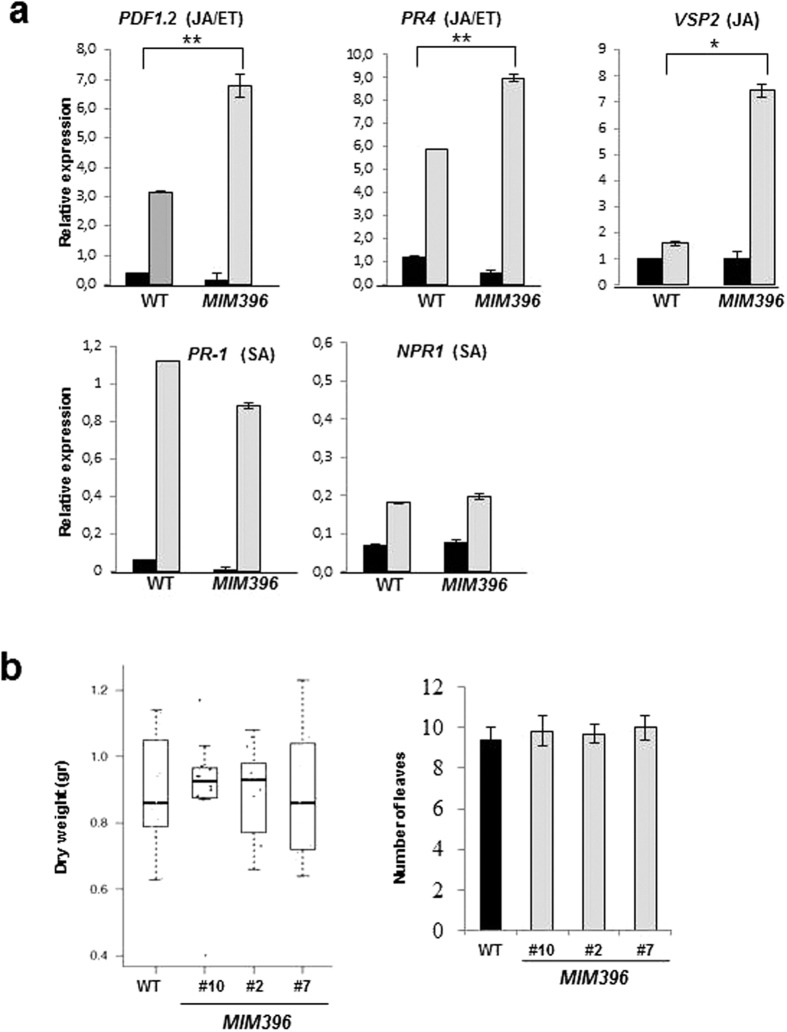
Expression of marker defense genes and growth performance of *MIM396* plants. (**a**) RT-qPCR analysis showing the expression of genes associated with JA and ET-dependent (*PDF1.2, PR4*), JA-dependent (*VSP2*), and SA-dependent (*PR1, NPR1*) responses. Mock-inoculated and *P. cucumerina*-inoculated plants are represented by black and grey bars, respectively. Statistical significance was determined by ANOVA (*P ≤ 0.05). Histograms show the mean ± SD of 3 biological replicates, each with 12 plants per genotype. (**b**) Growth and development proxies. Dry weight without inoculation with fungal spores (left panel) and flowering time (right panel) of *MIM396* plants. The same 12 individuals per genotype were used in both quantifications. Histograms show means ± SD of 12 randomized plants per genotype.

Additionally, we further studied the expression of hormone-related genes linked to different defense responses in fungi-inoculated *MIM396* plants. We found that marker genes for both JA/ET (*PDF1.2, PR4*) and JA (*VSP2*) pathways were more highly upregulated in *MIM396* plants than in wild-type plants upon inoculation with *P. cucumerina* (Fig. 7a, upper panels). In contrast, we did not observe any significant difference in markers for the SA signaling pathway (*PR1, NPR1*) between pathogen responses in *MIM396* and wild type plants (Fig. 7a, lower panel). Hence, we conclude that stronger activation of JA/ET-regulated defense sustains the enhanced resistance to pathogen infection in *MIM396* plants. Whether the observed effect on JA/ET-regulated defense genes is a direct consequence or an indirect effect of miR396/target gene(s) functioning needs to be elucidated. In summary, the resistance of *MIM396* plants to fungal infection is based on a more robust immune response that increases the plant’s ability to resist pathogen infection.

### Normal growth of *MIM396* plants in the absence of pathogen challenge

The constitutive expression of defense responses is often accompanied by a concomitant impairment of growth and/or development. In order to further support the lack of a constitutive defense response in *MIM396* plants that would affect their normal performance, we monitored two diagnostic parameters of such detrimental phenomenon; biomass production (dry weight of whole mature plants) and aging (using flowering time as proxy). As shown in (Fig. 7b), we found no significant differences when plants from 3 independent *MIM396* lines were grown along with wild type. This is in agreement with the finding that, in the absence of pathogen challenge, a reduction in miR396 function has a low impact on the whole-rosette transcriptome.

## Discussion

We have provided evidence that a gradual decrease of miR396 activity, and a concomitant increase of its transcription factor targets, play a central role in immune responses against necrotrophic and hemibiotrophic fungal pathogens. Furthermore, we have demonstrated that reduced miR396 activity results in superactivation of defense responses that enables a more successful immune response without interfering with normal growth or development. Potentiation of defense responses is an attractive focus for biotechnological approaches in order to develop varieties with improved performance upon pathogen pressure without detriment to normal plant development. As the miR396-GRF regulatory node is conserved among plant species^46^, results here presented will be useful in developing novel strategies for disease resistance in crops at the miRNA level.

Distinct miRNAs have been linked to the genetic programs involved in plant defense, although little is known about the molecular mechanisms underlying their role in those processes^15^,^50^,^51^. Even less is known about these mechanisms of defense against fungal pathogens as most studies carried out in Arabidopsis so far have focused on the role of miRNAs in immunity against the bacterial pathogen *Pseudomonas syringae*^12^,^15^,^51^,^52^. Results here presented expand our knowledge on the biological functions of miR396 miRNA in plants and establish link between miR396 function and immune responses against fungal pathogens in Arabidopsis plants.

The present work provides evidence that the transcriptional activity of *MIR396* progressively decreases upon fungal perception (inoculation with fungal spores, and treatment with fungal elicitors), while miR396 targets increase in expression. Our functional studies show that miR396 regulation of its transcription factor targets is pivotal within the dynamic response that allows appropriate modulation of plant defense responses. Distortion of this dynamic pattern by either prematurely reducing or increasing miR396 levels leads to improved performance (*MIM396* plants) or heightened susceptibility (*35Sprom:MIR396B* plants) to pathogen infection. That plants with constitutively higher miR396 levels are more susceptible to *P. cucumerina* suggest its targets limit a successful defense against fungal pathogens during host reprogramming. The existence of additional regulatory mechanisms acting in parallel to miR396-guided target gene expression should not be discarded.

Although initial reports identified GRFs as transcription factors with a function in leaf and stem development, recent studies have uncovered functions of GRFs in other developmental processes such as flowering, seed and root development and regulation of plant longevity^26^,^27^,^47^,^53^,^54^,^55^,^56^. Also, different stresses lead to changes in miR396 accumulation, including interactions with nematodes, bacteria, oomycetes and abiotic stress, among others^28^,^44^,^57^,^58^,^59^. However, little is known about the miR396-mediated molecular reprogramming in those different scenarios. A functional classification of AtGRF1 and AtGRF3 putative downstream targets revealed that these particular *GRF* genes contribute to the regulation of biological processes involved in defense responses and disease resistance (i.e. defense-related transcription factors, cell-wall modifications, cytokinin biosynthesis and signaling, and secondary metabolites accumulation)^27^. Studies relating miR396 regulation to plant-nematode interaction found that miR396-targeted GRFs regulate syncytial gene expression in roots, pointing to the existence of organ-specific GRF triggered transcriptional reprogramming^44^,^60^.

Results here presented indicated that the molecular and physiological responses to *P. cucumerina* perception are very similar in *MIM396* and wild-type plants, and include ROS production and callose deposition, central components of PTI^39^,^61^,^62^, as well as a large overlap in transcriptional responses. Nevertheless, *MIM396* plants showed a quantitatively stronger response with higher accumulation of ROS and callose and a more robust transcriptional reprogramming compared to wild-type plants upon pathogen challenge. In agreement with previous reports on the balance between JA/ET- and SA-based defenses, enhanced performance of *MIM396* plants relies on a stronger response of JA- and ET-mediated defense responses^35^,^41^,^63^,^64^,^65^. ABA and auxin signaling pathways have also been shown to mediate resistance to *P. cucumerina*^35^,^41^,^65^. Transcriptional reprogramming also shows stronger changes in genes involved in cell wall dynamics in line with previous results which pointed out the relevance of the cell wall in mediating Arabidopsis resistance to *P. cucumerina*^66^,^67^,^68^.

It is noteworthy that, in the absence of pathogen challenge, the transcriptome and development of *MIM396* are basically indistinguishable from those in wild-type plants. MiR396 down-regulation and release of its targets sensitize plants to mount a more robust defense response during pathogen infection, a situation that resembles the so called defense priming phenomenon^29^. Here, plants exposed to biotic or abiotic stress, as well as plants treated with certain natural or synthetic chemicals (i.e. β-aminobutyric acid, BABA), are often promoted to a primed state and deploy a faster and/or stronger defense response^29^. Despite being a long recognized phenomenon, little is known about the molecular mechanisms underlying defense priming which includes chromatin modifications and control of MAPK activity^69^,^70^. Priming is usually characterized by the lack of major changes in transcriptome prior to stress exposure, as it is also the case of *MIM396* plants. Now, it will be of interest to determine whether *MIM396* plants, in addition to mount a stronger immune response, also activate defense responses faster than wild-type plants. How these are related to the already known events involved in priming, chromatin remodeling, accumulation of latent, inactive proteins that function in the host immune system (e.g. MAPKs), or alterations in primary metabolism, merits further studies. Particularly, chromatin remodeling has emerged as an important factor during defense responses^71^, and might well be one of the possible explanations to the lack of constitutive defense response in *MIM396* plants derived from higher levels of miR396-targeted transcription factors. It will be of interest to assess whether fast chromatin remodeling is a prerequisite for transcriptome alterations in *MIM396* plants upon pathogen challenge. Alternatively, the accumulation of a pool of inactive proteins that play a regulatory role in the host defense response (i.e. proteins whose function is regulated by Ca^++^ levels, cellular redox status, or phosphorylation processes) might also explain the lack of constitutive defense responses in *MIM396* plants. These proteins will only become active under pathogen infection conditions allowing the plant to respond more rapidly and/or more strongly^72^,^73^. Further studies are needed to gain further insights into the molecular mechanisms underlying potentiation of defense responses in *MIM396* plants.

In conclusion, our results support that in addition to its role in controlling developmental processes, miR396 contributes to the dynamic defense response against necrotrophic and hemibiotrophic fungal pathogens. Whereas interference on miR396 activity increases resistance to pathogen infection, miR396 overexpression results in enhanced susceptibility to infection, thus, supporting that miR396 negatively regulates immunity against fungal pathogens in Arabidopsis. The finding that reduced miR396 activity contributes to superactivation of defense responses, including H_2_O_2_ accumulation and callose deposition, expands our knowledge about the events involved in defense priming. Results here presented will not only advance our fundamental understanding of how plants integrate and balance immune responses and normal growth programs, but, ultimately, also has the potential to aid in the development of novel strategies to improve plant resistance to pathogen infection.

## Methods

### Plant material and growth conditions

*Arabidopsis thaliana* plants were grown in phytochambers on a mixture of soil, perlite and vermiculite (2:1:1) under neutral day conditions (12 h:12 h, light:dark cycle), at 22 °C: 20 °C, day:night, and 60% relative humidity. The lines used in this study are listed in Supplementary Table S1. The *MIM* lines were described elsewhere^30^.

### Disease resistance assays and histochemistry

Fungi were grown at 28ªC on PDA (potato dextrose agar). Spores were collected by adding sterile water to the surface of the mycelium and adjusted to the appropriate concentration using a Bürker counting chamber. Seedlings were assayed for resistance to *P. cucumerina* using 96-well plate assays^74^. Seedlings were grown in microtiter plates for 5 days before inoculating them with *P. cucumerina* spores (PcBMM isolate, 10^5^ spores mL^−1^ 34).

For disease resistance assays in soil-grown plants, three-week-old plants were spray-inoculated with a suspension of *P. cucumerina* spores (4 × 10^6^ spores mL^−1^, 0.4 mL per plant), *F. oxysporum* f. sp. *conglutinans (FOC*) (10^6^ spores mL^−1^) and *C. higginsianum* (4 × 10^6^ spores mL^−1^). In the case of *FOC*, infection assays were also carried out when the spore suspension was applied to the soil using 10^7^ spores mL^−1^ and 200 μL per plant. Inoculation with *B. cinerea* spores was performed by placing 5 μL drops (2 × 10^6^ spores mL^−1^) onto needle-pick wounds (2 droplets per leaf). Infected plants, as well as mock-inoculated plants were maintained under high humidity and the progress of disease symptoms was followed with time. Three independent experiments were performed (at least 12 plants per genotype in each experiment). Statistically significant differences among genotypes were determined by one-way ANOVA test. Lesion areas were measured with software ASSESS v2.0 75 on 4 inoculated leaves per plant and 4 plants per each genotype.

Elicitor treatments were performed by spraying three-week old plants with an elicitor extract obtained from *P. cucumerina* (300 μg mL) as described^76^. Three independent experiments were performed (at least 9 plants for each genotype).

For trypan blue staining, leaves were fixed by vacuum infiltration for 1 h in ethanol:formaldehyde:acetic acid (80:3.5:5 v/v), stained with lactophenol blue solution for 4 h, washed with water for 5 min and observed using a Zeiss Axiophot microscope under bright-field illumination. Callose deposition was determined by aniline blue staining. For this, chlorophyll was first removed from leaves with 70% ethanol and then incubated with 70 mM phosphate buffer pH 9.0 supplemented with aniline blue (0.01% w/v) for 2 h. Leaves were mounted in slides with glycerol an observed with an epifluorescence microscope (Zeiss Axiophot) under UV illumination. Pixel quantification of callose was performed using Adobe Photoshop and callose deposition was quantified by determining the relative number of yellow pixels on digital photographs^32^,^61^.

Quantification of fungal DNA on infected leaves was carried out by real-time PCR using specific primers for the corresponding fungus and the *UBIQUITIN21* (At5g25760) gene as an internal control. PCR primers are listed in Supplementary Table S6.

To determine H_2_O_2_ levels, 15 day-old plants were immersed in a DAB (3,3′-diaminobenzidine) solution (1 mg mL) with vacuum treatment for 30 min, then incubated over-night on agitation, washed with 70% ethanol for 1 h and observed using a Zeiss Axiophot microscope under bright-field illumination. Histochemical staining of GUS enzyme activity was performed according to Jefferson *et al*.^77^.

### Transgenic plants

For *MIR396B* overexpression constructs, a 302-bp genomic sequence containing the miR396b precursor was obtained by PCR from *A. thaliana* Col-0 genomic DNA using primers designed to create *KpnI* and *XbaI* restriction sites (Supplementary Table S6). The purified PCR products were first cloned into a pGEM-T Easy vector and then cloned into the corresponding restriction sites of the binary vector pCAMBIA1300 under the control of the *CaMV 35 S* promoter. For plant transformation, the DNA construct was mobilized into *Agrobacterium tumefaciens* strain GV3101 and introduced into Col-0 by the floral dip method^78^. Homozygous lines were obtained for further characterization. An empty pCAMBIA1300 vector was used as a control. Transgenic lines harboring the pCAMBIA1300 empty vector were used as controls.

### RT-qPCR and stem-loop RT-PCR

Total RNA was extracted using TRIzol Reagent (Invitrogen). First-strand cDNA was synthesized from DNase-treated total RNA (1 μg) with SuperScript III reverse transcriptase (Invitrogen GmbH) and oligo-dT18 (Qiagen, Hilden, Germany). RT-qPCRs were performed in optical 96-well plates in a Light Cycler 480 (Roche) using SYBR^®^ Green. Primers were designed using Primer Express software (Applied Biosystems, Foster City, CA, USA). The β*-TUBULIN2* gene (At5g62690) was used to normalize the transcript level in each sample. The average cycle threshold (Ct) values from triplicate PCRs were normalized to the average Ct values for the *Actin2* gene from the same RNA preparations yielding the ΔCt value. Three independent biological replicates with three technical replicates were analysed. An ANOVA test was used to evaluate differences in gene expression. Accumulation of mature miR396 sequences was determined by stem-loop reverse transcription quantitative PCR as previously described^25^. Primer sequences are listed in Supplementary Table S6. Nucleotide sequencing confirmed the specific amplification of miR396 sequences.

### Microarray analysis

Total RNA (100 ng) was labeled using Low Input Quick Amp Labeling kit (Agilent 5190-2305) following manufacturer instructions. Briefly, mRNA was reverse transcribed in the presence of T7-oligo-dT primer to produce cDNA. cDNA was then *in vitro* transcribed with T7 RNA polymerase in the presence of Cy3-CTP to produce labeled cRNA. The labeled cRNA was hybridized to the Agilent *Arabidopsis thaliana* version 4 gene expression 4 × 44 K microarray (ID021169) according to the manufacturer’s protocol. The arrays were washed, and scanned on an Agilent G2565CA microarray scanner at 100% PMT and 3 μm resolution. Intensity data was extracted using the Feature Extraction software (Agilent). For the statistical analysis, raw data was taken from the Feature Extraction output files and was corrected for background noise using the normexp method^79^. To assure comparability across samples, we used quantile normalization. Differentially expressed genes were identified with an empirical Bayes approach on linear models (limma), excluding control probes on the array^80^. Results were corrected for multiple testing according to the False Discovery Rate (FDR) method^81^. Three biological replicates (each consisting on a pool of 6 plants), and three technical replicates for each biological replicate, were assayed. All statistical analyses were performed with the Bioconductor project (http://www.bioconductor.org/) in the R statistical environment (http://cran.r-project.org/)^82^. Differentially expressed genes were selected based on fold change (FC ≥ 2.0 as a threshold) and significance level (P ≤ 0.05).

Data sets from transcriptome analyses have been deposited at the NCBI Gene Expression Omnibus repository, accession number GSE76819. Gene Ontology (GO) classification was performed using agriGO (http://bioinfo.cau.edu.cn/agriGO/)^83^.

### Phenotyping

Plants were grown in a randomized fashion in individual pots at 23 °C in long day conditions (16 h: 8 h, light:dark) and 65% humidity. 36 days-old plants were collected in previously weighted paper envelopes and sealed. After 3 weeks drying, individual plants were weighted (whole plants, including reproductive organs and seeds). A minimum of 12 plants was included per genotype in the assay.

## Additional Information

**How to cite this article:** Soto-Suárez, M. *et al*. The Arabidopsis miR396 mediates pathogen-associated molecular pattern-triggered immune responses against fungal pathogens. *Sci. Rep.* **7**, 44898; doi: 10.1038/srep44898 (2017).

**Publisher's note:** Springer Nature remains neutral with regard to jurisdictional claims in published maps and institutional affiliations.

## Supplementary Material

Supplementary Information
