# Supplementary material for: The Arabidopsis miR396 mediates pathogen-associated molecular pattern-triggered immune responses against fungal pathogens
Source: Sci Rep. 2017 Mar 23;7:44898. doi: 10.1038/srep44898 (PMC5362962; doi:10.1038/srep44898)
Supplement: Supplementary Information [file srep44898-s1.pdf]

The Arabidopsis miR396 mediates pathogen-associated molecular pattern-triggered immune responses against fungal pathogens

Mauricio Soto-Suárez, Patricia Baldrich, Detlef Weigel, Ignacio Rubio-Somoza and Blanca San Segundo

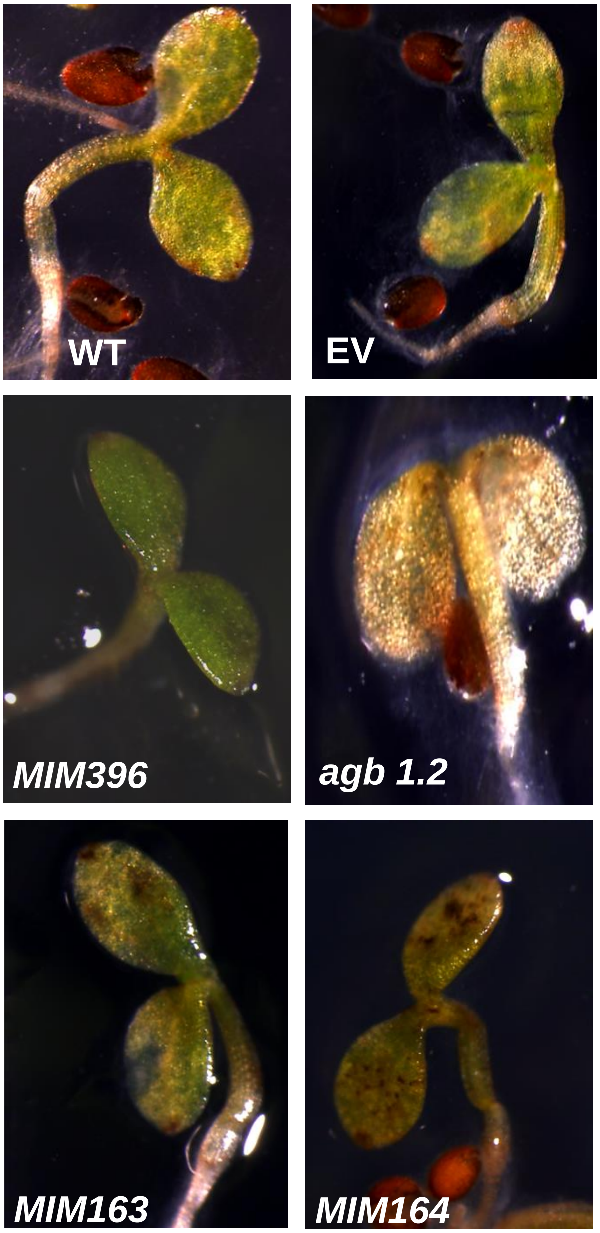

Supplementary Figure S1. Arabidopsis seedlings after inoculation with *P. cucumerina*. 5 day-old seedlings were inoculated with fungal spores (200 spores mL^{-1}) and images were taken at 4 dpi. Representative images of lines showing a clear phenotype of disease resistance (*MIM396*) or susceptibility (*MIM163, MIM164*) are shown. The *agb1.2* mutant is a control known to be susceptible to *P. cucumerina* infection (Llorente et al., 2005).

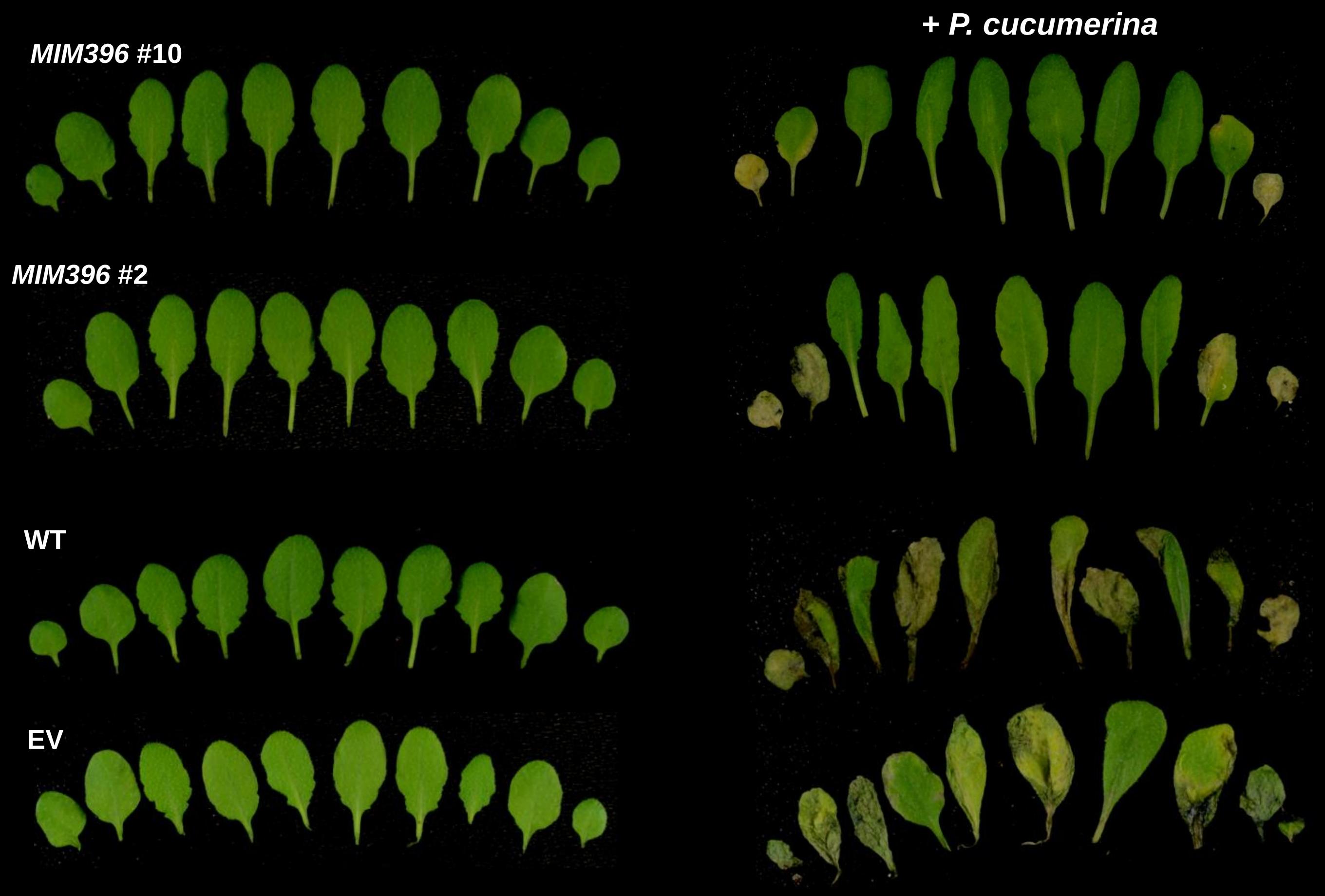

Supplementary Figure S2. **Dissected rosettes of 21 day old plants after inoculation with *P. cucumerina***. Mock-inoculated (left panel) and *P. cucumerina*-inoculated (4 x 10^6 spores mL^{-1}; right panel). Pictures were taken at 7 dpi.

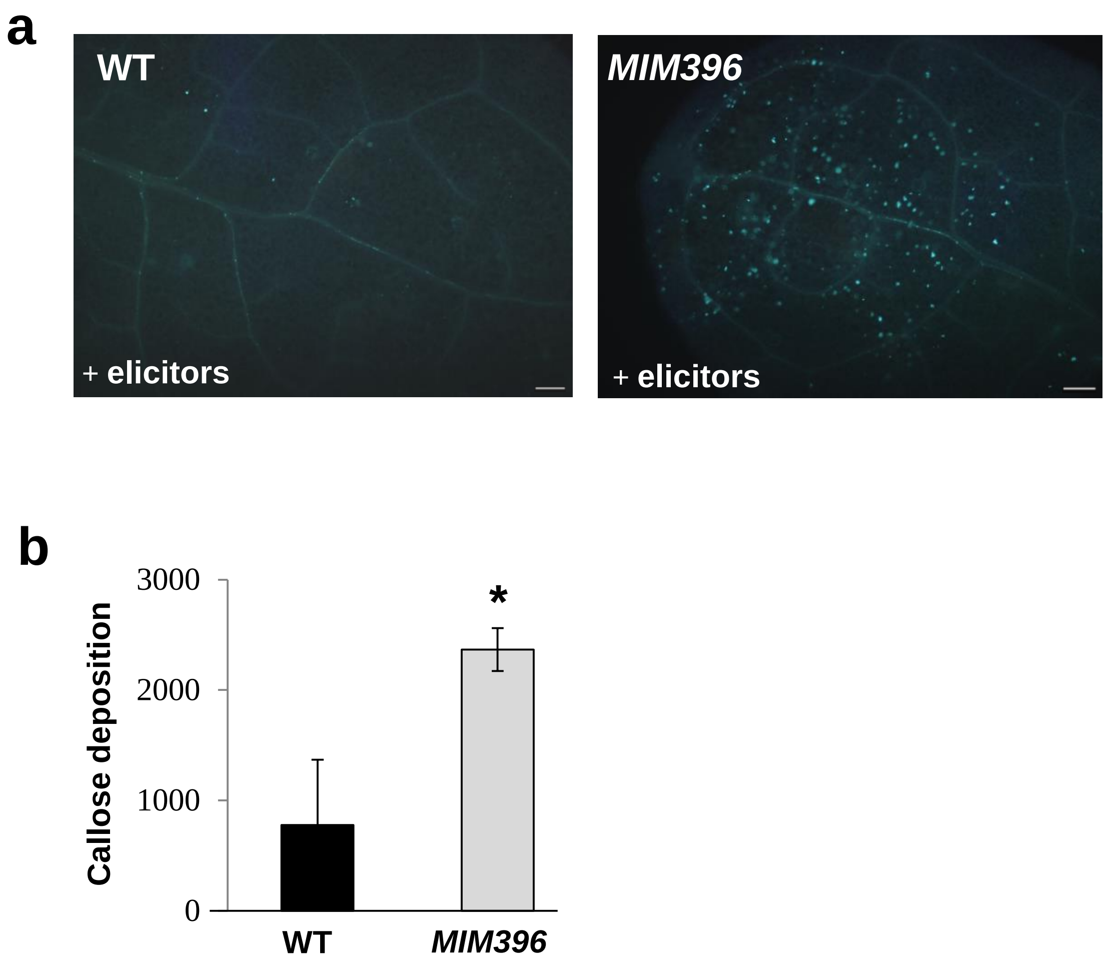

Suplementary Figure S3. Callose deposition in plants treated with a *P. cucumerina* extract. Callose deposition was calculated as arbitrary units by quantifying the number of yellow pixels per million on digital micrographs of infected leaves. Bars represent mean ± SD (n = 3 biological replicates, 3 independent lines; 9 plants per independent line, and 3 leaves per plant).

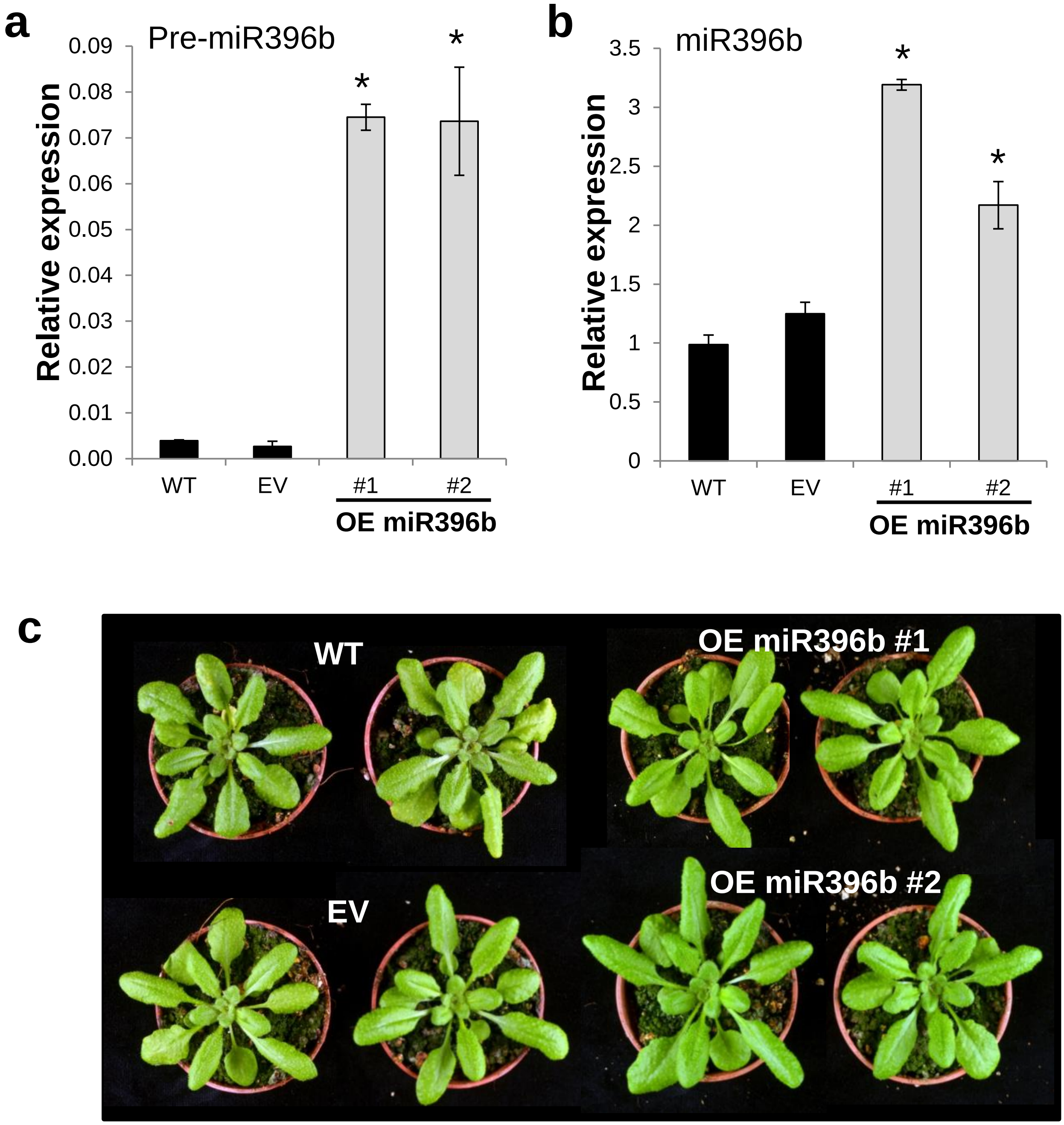

Supplementary Figure S4. **Characterization of non-infected *35Sprom::MIR396B* plants. (a, b)** Expression of pre-miR396b (a) and mature miR396b (b). Histograms show the mean ± SD of 2 biological replicates, each with 12 plants per genotype (ANOVA test; P ≤ 0.05). **(c)** Rosettes of 3-week-old plants.

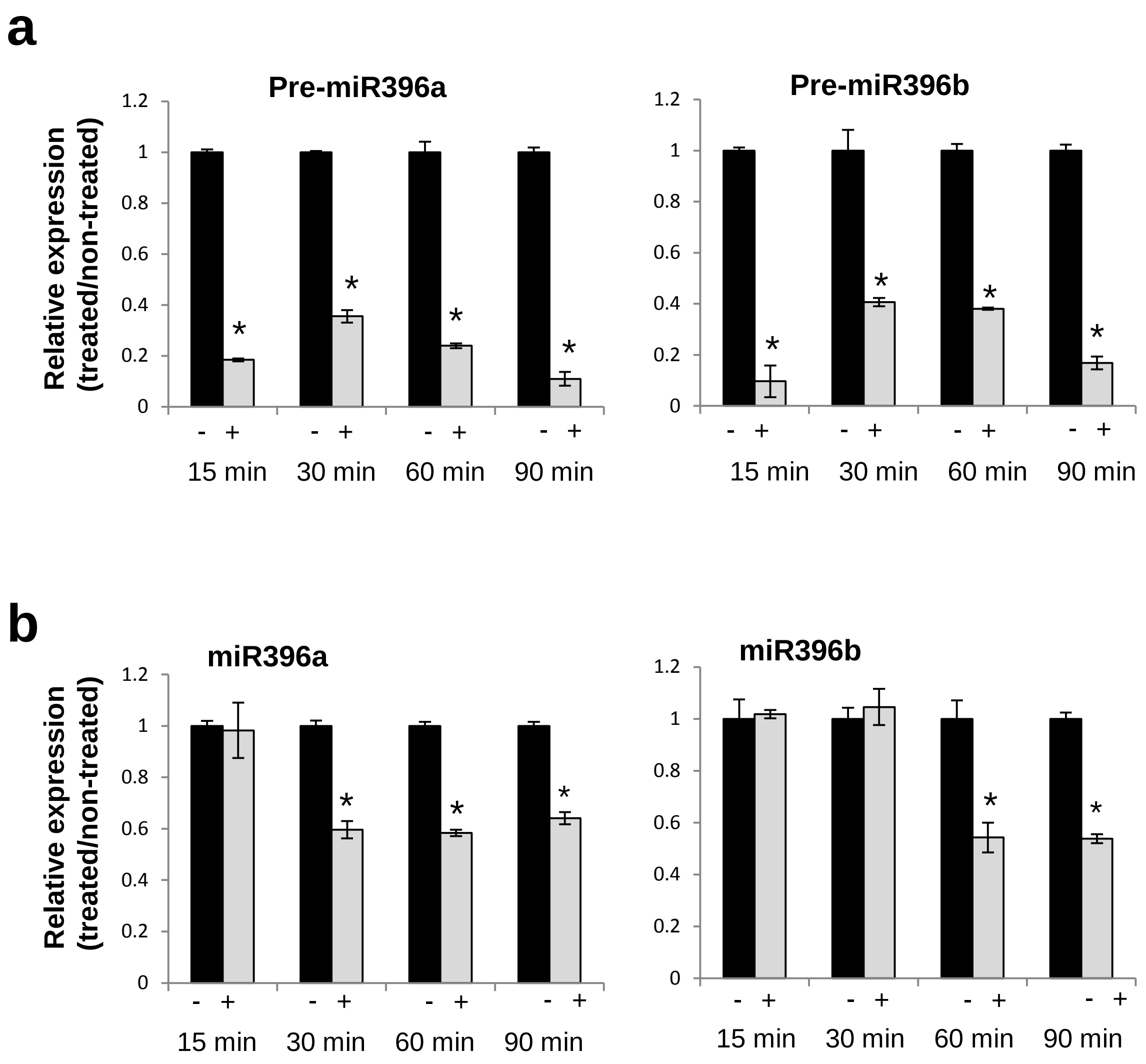

Supplementary Figure S5. Accumulation of pre- and mature miR396 sequences in wild-type plants. Plants were mock inoculated or treated with a *P. cucumerina* elicitors for the indicated time periods. **(a)** Accumulation of pre-miR396a and pre-miR396b sequences (light and dark grey bars, respectively). **(b)** Accumulation of mature miR396. The expression level in mock-inoculated plants was set to 1.0. The values represent changes in the accumulation of miR396a and miR396b at the indicated times after inoculation (*, P ≤ 0.05; ANOVA test, elicitor-treated vs. mock-treated each time point). Bars represent mean ±SD (n = 3 biological replicates, 3 independent lines; 9 plants per independent line).

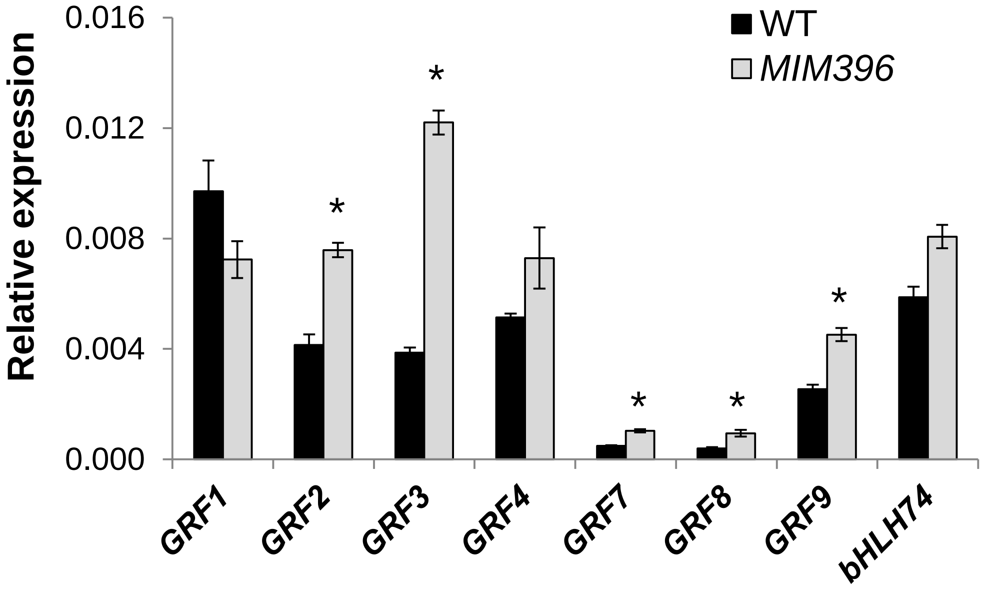

Supplementary Figure S6. Expression of miR396 targets in non-infected wild type and *MIM396* plants. For each gene, the values represent changes of expression in *MIM396* plants relative to wild-type plants (values in wild type plants were set to 1) as determined by RT-qPCR analysis. Statistical significance was determined by ANOVA (*, P ≤ 0.05). Histograms show the mean ± SD of 3 biological replicates, each with 12 plants per genotype.

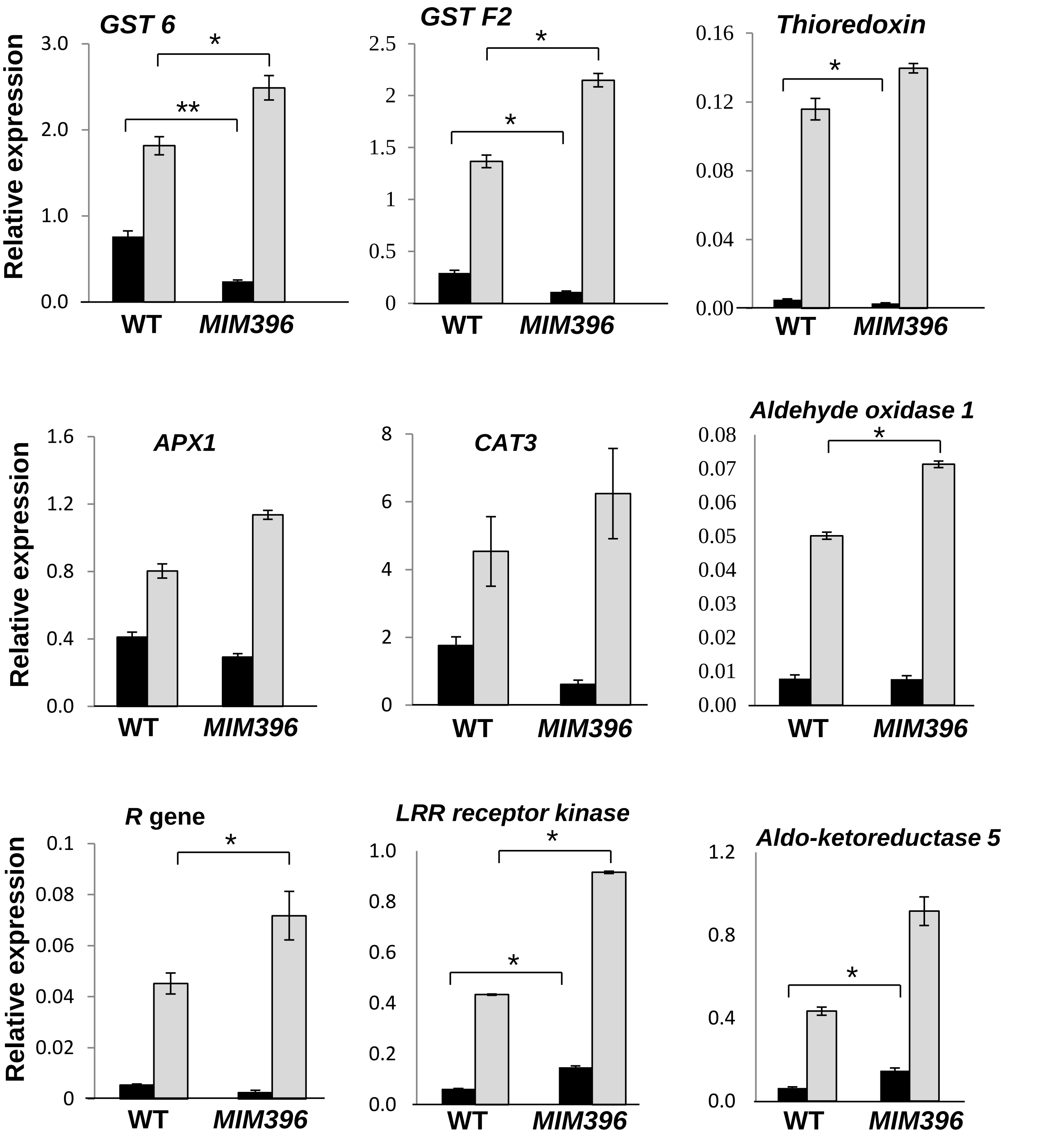

Supplementary Figure S7. RT-qPCR validation of differentially expressed genes identified by microarray analysis. Mock-inoculated or *P. cucumerina*-inoculated plants (black and grey bars, respectively). Defense gene expression is induced to higher levels in the *P. cucumerina*-inoculated *MIM396* plants. *GLUTATHIONE-S-TRANSFERASE6* (*GST6*, At1g02930), *GST-F2* (At4g02520), *THIOREDOXIN* (At5g38900), *ASCORBATE PEROXIDASE1* (*APX1*, At1g07890), *CATALASE3* (*CAT3*, At1g20620), *ALDEHYDE OXIDASE1* (At5g20960), an NLR gene (At4g11170), an LRR receptor kinase gene (At1g74360), and *ALDO-KETOREDUCTASE5* (At1g60730). *β-TUBULIN2* (At5g62690) was used for normalization. The experiment was carried out with 3 biological and three technical replicates). Asterisks indicate significant differences between the indicated genotypes or condition (*, $P \leq 0.05$; **, $P \leq 0.01$; one-way ANOVA test).

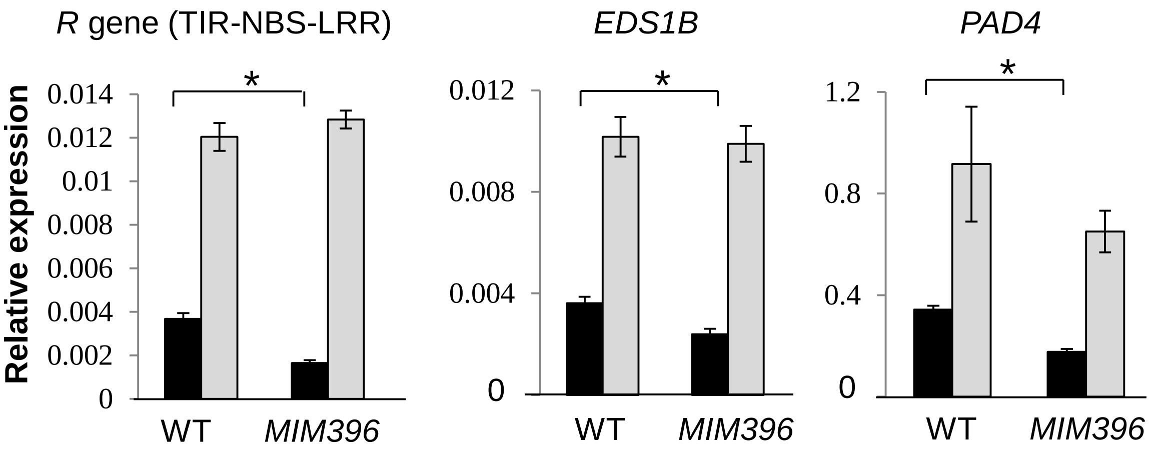

Supplementary Figure S8. RT-qPCR validation of differentially expressed genes identified by microarray analysis. Samples were mock-inoculated and *P. cucumerina*-inoculated plants, at 72 hours post-inoculation (black and grey bars, respectively). In the absence of pathogen infection, defense-related genes are expressed at a lower level in *MIM396* plants relative to WT plants, its expression being induced at similar levels by fungal infection in the two genotypes. NLR gene (TIR-NBS-LRR, At5g45000), *ENHANCED DISEASE SUSCEPTIBILITY1B* (*EDS1B*, At3g48080), and *PHYTOALEXIN DEFICIENT4* (PAD4, At3g52430). *β-TUBULIN2* (At5g62690) was used for normalization. Samples were the same as in Supplemenary Figure S7.

Supplementary Table S1. ***Arabidopsis thaliana*** **lines used in this work**

Name	Ecotype	Described by
WT	Columbia 0	-
EV (pGREEN vector)	Columbia 0	Todesco et al., 2010
MIM160	Columbia 0	Todesco et al., 2010
MIM163	Columbia 0	Todesco et al., 2010
MIM164	Columbia 0	Todesco et al., 2010
MIM165/166	Columbia 0	Todesco et al., 2010
MIM169	Columbia 0	Todesco et al., 2010
MIM170	Columbia 0	Todesco et al., 2010
MIM171a	Columbia 0	Todesco et al., 2010
MIM171bc	Columbia 0	Todesco et al., 2010
MIM172	Columbia 0	Todesco et al., 2010
MIM393	Columbia 0	Todesco et al., 2010
MIM395	Columbia 0	Todesco et al., 2010
MIM396	Columbia 0	Todesco et al., 2010
MIM413	Columbia 0	Todesco et al., 2010
MIM774	Columbia 0	Todesco et al., 2010
MIM841	Columbia 0	Todesco et al., 2010
agb1.2	Columbia 0	Llorente et al., 2005
35Sprom::GUS	Columbia 0	Present work
MIR396Bprom:GUS	Columbia 0	Debernardi et al., 2012
35Sprom::MIR396B	Columbia 0	Present work

Supplementary Table S2. Differentially expressed genes in *MIM396* plants relative to wild-type plants, sorted by functional category

Description	GeneName	GO category	MIM contr vs WT contr	
			Fold Change	adj.P.Val (Holm)
pathogen and circadian controlled 1 mRNA	AT3G22231	Biotic Stress	**-2.06**	0.009433915
cytochrome P450 71B23	AT3G26210	Biotic Stress	**-2.10**	0.033791542
lipase class 3 family protein / disease resistance protein-related protein (EDS1B)	AT3G48080	Biotic Stress	**-2.21**	0.03269516
glycine-rich protein	AT5G28630	Biotic Stress	**-2.70**	0.006118229
TIR-NBS-LRR class disease resistance protein	AT5G45000	Biotic Stress	**-2.86**	0.001199229
lectin-like protein	AT5G03350	Cell wall	**-2.14**	0.010078653
alpha-galactosidase 1	AT5G08380	Metabolism	**4.09**	2.89E-05
cysteine-rich receptor-like protein kinase 20	AT4G23280	Signaling	**-2.17**	0.032145341
protein kinase family protein	AT4G11890	Signaling	**-2.86**	0.030572973
protein kinase family protein	AT4G11890	Signaling	**-3.07**	0.005725439
Leucine-rich repeat protein kinase family protein	AT1G51850	Signaling	**-3.27**	0.003215724
leucine-rich repeat protein kinase-like protein	AT1G51790	Signaling	**-3.34**	5.56E-06
Pseudogene	AT3G30122	Unknown	**2.15**	0.008613401
uncharacterized protein	AT1G13520	Unknown	**-2.02**	0.003229077
PB1_UP2 domain-containing protein	AT3G26510	Unknown	**-2.31**	0.022248915
uncharacterized protein	AT3G22240	Unknown	**-2.33**	0.031154973
uncharacterized protein	AT3G22235	Unknown	**-2.44**	0.049435533
uncharacterized protein	AT3G22235	Unknown	**-2.57**	0.009594723
uncharacterized protein	AT1G01130	Unknown	**-2.77**	0.003430683
Unkcharacterized protein	At3g29646	Unknown	**-5.11**	0.000259162

Supplementary Table S3. Differentially expressed genes in wild type plants after challenge with *P. cucumerina*

WT_Pcc vs. WT_Mock				
Description	Description	Go Category	Fold Change	Ajusted p-value (Holm)
flavin-dependent monooxygenase 1 (FMO1)	AT1G19250	Biotic Stress	209.47	2.55E-08
GDSL lipase 1	AT5G40990	Biotic Stress	51.98	3.60E-07
AGD2-like defense response protein 1	AT2G13810	Biotic Stress	44.07	3.85E-07
lipid transfer protein (LTP)	AT3G22600	Biotic Stress	43.71	1.08E-04
cytochrome P450 71A12	AT2G30750	Biotic Stress	37.19	2.99E-04
chitinase class 4-like protein	AT2G43570	Biotic Stress	32.95	5.26E-04
PR-6 proteinase inhibitor family protein	AT5G43570	Biotic Stress	32.39	7.87E-04
disease resistance protein RMG1	AT4G11170	Biotic Stress	23.26	3.13E-07
protein AIG1 (AVRRPT2-Induced Gene 1)	AT1G33960	Biotic Stress	22.05	2.51E-06
calcium-binding protein CML47	AT3G47480	Biotic Stress	18.96	4.04E-04
cytochrome P450, family 71, subfamily A, polypeptide 13	AT2G30770	Biotic Stress	17.03	4.13E-04
chitinase A	AT5G24090	Biotic Stress	16.76	1.51E-05
Chitinase family protein	AT2G43590	Biotic Stress	16.41	2.19E-04
cysteine/histidine-rich C1 domain-containing protein	AT2G17740	Biotic Stress	15.96	2.31E-03
lipid-transfer protein/seed storage	AT4G12500	Biotic Stress	13.70	4.81E-02
uncharacterized protein	AT5G57510	Biotic Stress	13.28	2.97E-02
lipid-transfer protein/seed storage	AT1G36150	Biotic Stress	12.86	1.76E-02
BON1-associated protein 2	AT2G45760	Biotic Stress	11.94	2.53E-03
chitinase	AT3G47540	Biotic Stress	9.18	2.39E-02
uncharacterized protein	AT5G24200	Biotic Stress	8.88	4.75E-04
disease resistance protein	AT5G66890	Biotic Stress	8.64	4.70E-05
FAD-binding and BBE domain-containing protein	AT1G26420	Biotic Stress	8.63	2.85E-04
chitinase	AT2G43620	Biotic Stress	8.62	6.82E-03
basic chitinase B	AT3G12500	Biotic Stress	8.39	1.58E-03
TIR-NBS class of disease resistance protein	AT1G72890	Biotic Stress	7.72	1.48E-06
Isochorismate synthase 1	AT1G74710	Biotic Stress	6.87	3.51E-05
PR-6 proteinase inhibitor family protein	AT2G38870	Biotic Stress	6.84	6.09E-05
Toll-Interleukin-Resistance domain-containing protein	AT5G44920	Biotic Stress	6.06	4.37E-02
Disease resistance protein (TIR-NBS class)	AT1G17615	Biotic Stress	6.03	2.06E-05
UDP-dependent glycosyltransferase 76B1	AT3G11340	Biotic Stress	5.71	1.45E-05
downy mildew resistance 6 protein / oxidoreductase	AT5G24530	Biotic Stress	5.45	2.16E-04
C2 calcium/lipid-binding plant phosphoribosyltransferase	AT4G00700	Biotic Stress	5.36	4.70E-03
defensin-like protein 308	AT5G46871	Biotic Stress	4.91	5.07E-03
defensin-like protein 309	AT5G46874	Biotic Stress	4.45	2.80E-03
carboxyesterase 17	AT5G16080	Biotic Stress	4.41	4.62E-04
LURP1 protein	AT2G14560	Biotic Stress	4.40	7.40E-03
uncharacterized protein	AT5G64510	Biotic Stress	4.39	6.57E-05
RPW8 domain-containing powdery mildew resistance protein	AT3G26470	Biotic Stress	4.16	2.39E-03
uncharacterized protein	AT5G45410	Biotic Stress	3.91	8.39E-05
pathogenesis-related protein 1 domain-containing protein	AT4G07820	Biotic Stress	3.84	2.04E-02
cytochrome P450 71B23	AT3G26210	Biotic Stress	3.67	1.17E-04
LRR and NB-ARC domain-containing disease resistance protein	AT3G14460	Biotic Stress	3.59	1.03E-04
receptor like protein 50	AT4G13920	Biotic Stress	3.42	8.74E-05
MLO-like protein 3	AT3G45290	Biotic Stress	3.36	8.57E-05
avirulence induced protein (AIG1)	AT1G33950	Biotic Stress	3.29	3.05E-04
phospholipase-like protein (PEARLI 4) family	AT2G16900	Biotic Stress	3.27	8.26E-05
uncharacterized protein	AT4G29110	Biotic Stress	3.21	4.66E-04
VQ motif-containing protein	AT2G41180	Biotic Stress	2.95	1.48E-03
beta glucosidase 10	AT4G27830	Biotic Stress	2.95	1.43E-02
4-coumarate-CoA ligase 1	AT1G51680	Biotic Stress	2.90	3.10E-03
alcohol dehydrogenase-like 6	AT5G24760	Biotic Stress	2.77	5.43E-04
Bax inhibitor-1 family protein	AT5G47130	Biotic Stress	2.75	4.65E-03
methyl esterase 1	AT2G23620	Biotic Stress	2.69	1.49E-03
CC-NBS-LRR class disease resistance protein	AT1G12290	Biotic Stress	2.66	2.35E-04
haloacid dehalogenase-like hydrolase domain-containing protein	AT2G41250	Biotic Stress	2.65	1.16E-02
cytochrome P450 98A3	AT2G40890	Biotic Stress	2.61	2.39E-02
leucine-rich repeat-containing protein	AT5G45510	Biotic Stress	2.53	6.57E-03
defensin-like protein 36	AT1G69828	Biotic Stress	2.43	3.73E-02
calreticulin-3	AT1G08450	Biotic Stress	2.40	1.99E-04
FAD/NAD(P)-binding oxidoreductase family protein	AT4G38540	Biotic Stress	2.40	4.17E-05
TIR-NBS-LRR class disease resistance protein	AT5G51630	Biotic Stress	2.35	2.45E-04
NB-ARC domain-containing disease resistance protein	AT4G27220	Biotic Stress	2.35	1.04E-02
glycine-rich family protein	AT4G27850	Biotic Stress	2.23	7.36E-04

prolyl 4-hydroxylase 5	AT2G17720	Biotic Stress	2.16	5.53E-05
TIR-NBS-LRR class disease resistance protein	AT5G45000	Biotic Stress	2.14	2.68E-02
protein PHLOEM protein 2-LIKE A8	AT5G45070	Biotic Stress	2.13	1.52E-02
TIR-NBS-LRR class disease resistance protein	AT5G41740	Biotic Stress	-2.00	1.10E-04
leucine-rich repeat (LRR) family protein	AT1G33590	Biotic Stress	-2.13	1.96E-02
TIR-NBS-LRR class disease resistance protein	AT5G58120	Biotic Stress	-2.21	2.95E-04
chloroplast stem-loop binding protein	AT1G09340	Biotic Stress	-2.32	2.81E-02
TIR-NBS class of disease resistance protein	AT1G66090	Biotic Stress	-2.32	1.48E-02
TIR-NBS-LRR class disease resistance protein	AT1G56540	Biotic Stress	-2.32	1.21E-02
vegetative storage protein 2	AT5G24770	Biotic Stress	-2.33	4.63E-06
glycine-rich protein	AT5G61660	Biotic Stress	-2.33	2.99E-03
respiratory burst oxidase homologue D (AtRbohD)	AT5G47910	Biotic Stress	-2.36	3.16E-03
lipid-transfer protein/seed storage	AT2G27130	Biotic Stress	-2.40	4.45E-03
glycine-rich protein	AT4G29020	Biotic Stress	-2.49	3.15E-02
carbonic anhydrase 1	AT3G01500	Biotic Stress	-2.52	9.85E-03
TIR-NBS-LRR class disease resistance protein	AT4G19520	Biotic Stress	-2.57	3.04E-04
TIR-NBS-LRR class disease resistance protein	AT4G19530	Biotic Stress	-2.74	8.77E-03
uncharacterized protein	AT1G61260	Biotic Stress	-2.82	2.79E-05
TIR-NBS-LRR class disease resistance protein	AT3G44630	Biotic Stress	-2.85	6.03E-04
PLAT-plant-stress domain-containing protein	AT2G22170	Biotic Stress	-2.99	4.24E-03
MLO-like protein 4	AT1G11000	Biotic Stress	-3.02	6.42E-04
glycine-rich protein	AT1G07135	Biotic Stress	-3.09	1.42E-02
TIR-NBS-LRR class disease resistance protein	AT5G41750	Biotic Stress	-3.14	4.20E-04
lipid-transfer protein/seed storage	AT3G43720	Biotic Stress	-3.22	8.17E-04
Thaumatin-like protein	AT1G18250	Biotic Stress	-3.28	2.10E-02
Non-specific lipid-transfer protein-like protein	AT2G13820	Biotic Stress	-3.32	1.07E-03
uncharacterized protein	AT4G24275	Biotic Stress	-3.44	2.12E-02
uncharacterized protein	AT1G58420	Biotic Stress	-3.46	4.41E-02
RPM1-interacting protein 4 (RIN4) family protein	AT4G35655	Biotic Stress	-3.66	2.35E-02
Non-specific lipid-transfer protein-like protein	AT5G64080	Biotic Stress	-3.74	3.38E-03
MLO-like protein 8	AT2G17480	Biotic Stress	-4.23	2.94E-06
phytochelatin synthase 1	AT5G44070	Biotic Stress	-4.34	1.30E-04
TIR-NBS-LRR class disease resistance protein	AT5G46510	Biotic Stress	-4.65	8.30E-04
TIR class disease resistance protein	AT1G61100	Biotic Stress	-4.75	1.09E-04
ferrochelatase 1	AT5G26030	Biotic Stress	-4.96	3.24E-04
uncharacterized protein	AT3G57450	Biotic Stress	-4.98	3.52E-04
lipid-transfer protein/seed storage	AT1G73550	Biotic Stress	-5.01	2.24E-02
Toll-Interleukin-Resistance domain-containing protein	AT1G57850	Biotic Stress	-5.06	7.57E-03
dicarboxylate carrier 2	AT4G24570	Biotic Stress	-5.08	4.13E-02
pathogenesis-related thaumatin-like protein	AT1G19320	Biotic Stress	-6.01	3.72E-03
Pto-interacting 1-4	AT2G47060	Biotic Stress	-6.76	3.01E-08
glycine-rich cell wall protein-like protein	AT4G18280	Biotic Stress	-7.01	6.38E-06
TIR-NBS-LRR class disease resistance protein	AT5G22690	Biotic Stress	-7.47	3.07E-06
CBS domain-containing protein	AT5G53750	Biotic Stress	-10.27	3.09E-03
RPM1-interacting protein 4-like protein	AT2G17660	Biotic Stress	-12.03	4.96E-05
pathogenesis-related thaumatin family protein	AT4G36010	Biotic Stress	-13.27	6.99E-06
disease resistance-like protein/LRR domain-containing protein	AT2G34930	Biotic Stress	-20.26	5.18E-05
glycine-rich protein	AT5G28630	Biotic Stress	-23.90	1.10E-07
cytochrome P450, family 94, subfamily C, polypeptide 1	AT2G27690	Biotic Stress	-25.21	2.91E-05
cytochrome P450, family 94, subfamily B, polypeptide 1	AT5G63450	Biotic Stress	-31.68	3.00E-03
thioredoxin-dependent peroxidase 2	AT1G65970	Oxidative stress	56.04	0.00E+00
peroxidase 5	AT1G14550	Oxidative stress	37.46	2.23E-03
glutathione S-transferase tau 3	AT2G29470	Oxidative stress	32.47	1.31E-02
apoplastic peroxidase Prx37	AT4G08770	Oxidative stress	27.27	2.13E-02
peroxidase 52	AT5G05340	Oxidative stress	27.20	6.03E-05
glutathione S-transferase TAU 10	AT1G74590	Oxidative stress	22.36	9.32E-05
Thioredoxin superfamily protein	AT5G38900	Oxidative stress	21.93	2.94E-07
peroxidase	AT5G06730	Oxidative stress	16.52	1.20E-04
peroxidase 38	AT4G08780	Oxidative stress	10.00	1.09E-01
glutathione S-transferase TAU 11	AT1G69930	Oxidative stress	9.08	7.92E-03
peroxidase 50	AT4G37520	Oxidative stress	8.68	6.60E-04
glutathione transferase lambda 1	AT5G02780	Oxidative stress	8.41	3.25E-05
Regulator of Vps4 activity in the MVB pathway protein	AT1G13340	Oxidative stress	6.37	6.55E-05
Glutathione S-transferase family protein	AT5G44990	Oxidative stress	6.36	1.47E-06
peroxidase	AT5G19880	Oxidative stress	6.18	7.36E-04
glutaredoxin ATGRXS13	AT1G03850	Oxidative stress	5.36	5.69E-05
peroxidase 71	AT5G64120	Oxidative stress	4.71	1.57E-04
chlorophyll(ide) b reductase NYC1	AT4G13250	Oxidative stress	4.55	1.00E+00
glutathione S-transferase F2	AT4G02520	Oxidative stress	3.81	1.72E-04
glutaredoxin-like protein	AT1G64500	Oxidative stress	3.48	6.24E-03

glutathione peroxidase GPx	AT2G31570	Oxidative stress	2.84	5.99E-02
glutathione S-transferase zeta-class 1	AT2G02390	Oxidative stress	2.49	3.40E-03
aconitate hydratase 3	AT2G05710	Oxidative stress	2.46	3.71E-04
catalase 3	AT1G20620	Oxidative stress	2.44	2.02E-05
membrane-associated progesterone binding protein 3	AT3G48890	Oxidative stress	2.42	1.59E-03
glucose-6-phosphate dehydrogenase 4	AT1G09420	Oxidative stress	2.41	1.13E-03
Aldolase-type TIM barrel family protein	AT5G13420	Oxidative stress	2.39	1.01E-04
glutathione S-transferase	AT1G65820	Oxidative stress	2.37	1.36E-03
aconitase 2	AT4G26970	Oxidative stress	2.28	2.81E-03
protein reduce transmission through pollen	AT1G60420	Oxidative stress	2.20	1.79E-04
Pyridoxamine 5'-phosphate oxidase family protein	AT2G04690	Oxidative stress	2.19	3.70E-03
glutaredoxin-C1	AT5G63030	Oxidative stress	2.14	4.34E-04
glutaredoxin-C3	AT1G77370	Oxidative stress	2.14	9.02E-04
GroES-like zinc-binding alcohol dehydrogenase family protein	AT5G63620	Oxidative stress	2.13	3.70E-02
Thioredoxin H-type 2 - Arabidopsis thaliana (Mouse-ear cress), partial (74%)	AT5G39950	Oxidative stress	2.06	1.00E+00
Pyridoxamine 5'-phosphate oxidase family protein	AT2G04690	Oxidative stress	2.05	2.50E-04
atypical CYS HIS rich thioredoxin 4	AT1G08570	Oxidative stress	2.05	8.75E-03
thioredoxin F-type 1	AT3G02730	Oxidative stress	-1.99	0.00E+00
2-Cysteine peroxiredoxin	AT5G06290	Oxidative stress	-2.08	5.83E-02
Glutaredoxin family protein	AT5G01420	Oxidative stress	-2.11	5.40E-02
monothiol glutaredoxin-S2	AT5G18600	Oxidative stress	-2.12	3.66E-04
Thioredoxin superfamily protein	AT2G37240	Oxidative stress	-2.24	7.92E-04
Fe superoxide dismutase 2	AT5G51100	Oxidative stress	-2.39	2.86E-02
tRNA dihydrouridine synthase	AT3G63510	Oxidative stress	-2.39	2.28E-03
glutathione transferase lambda 2	AT3G55040	Oxidative stress	-2.49	1.55E-04
thioredoxin-like 2-2	AT4G29670	Oxidative stress	-2.59	5.16E-03
2-Cys peroxiredoxin BAS1	AT3G11630	Oxidative stress	-2.76	1.39E-02
laccase 8	AT5G01040	Oxidative stress	-3.21	2.22E-04
glutaredoxin-C12	AT2G47870	Oxidative stress	-3.23	2.80E-04
monothiol glutaredoxin-S1	AT1G03020	Oxidative stress	-3.36	7.71E-02
glyoxylate reductase 2	AT1G17650	Oxidative stress	-3.50	1.61E-02
NAD(P)-binding Rossmann-fold-containing protein	AT1G32220	Oxydative stress	-3.61	8.85E-04
monothiol glutaredoxin-S9	AT2G30540	Oxidative stress	-3.69	2.62E-02
peroxiredoxin Q	AT3G26060	Oxidative stress	-4.09	5.67E-02
monothiol glutaredoxin-S4	AT4G15680	Oxidative stress	-4.31	6.64E-05
monothiol glutaredoxin-S8	AT4G15660	Oxidative stress	-4.54	5.08E-03
glutathione S-transferase F11	AT3G03190	Oxidative stress	-4.59	1.86E-02
glutaredoxin-like protein	AT2G41330	Oxidative stress	-5.40	2.29E-07
monothiol glutaredoxin-S11	AT1G06830	Oxidative stress	-6.74	4.50E-03
alternative oxidase 1D	AT1G32350	Hormones	34.21	7.20E-02
1-aminocyclopropane-1-carboxylate synthase 2 (ACS2)	AT1G01480	Hormones	13.43	3.46E-01
ethylene-responsive transcription factor ERF096	AT5G43410	Hormone	12.70	5.65E-05
indole-3-acetic acid-amido synthetase GH3.2	AT4G37390	Hormones	9.53	1.64E-01
indole-3-acetic acid-amido synthetase GH3.3	AT2G23170	Hormone	9.10	1.25E-02
S-adenosyl-L-methionine-dependent methyltransferase-like protein	AT5G54400	Hormones	8.54	8.25E-02
aldehyde oxidase 1	AT5G20960	Hormone	8.09	7.17E-07
Oxoglutarate/iron-dependent oxygenase	AT5G59530	Hormone	7.84	4.85E-06
Mediator of ABA-regulated dormancy MARD1	AT3G63210	Hormone	5.61	9.69E-03
SAUR-like auxin-responsive protein	AT4G38860	Hormone	4.15	1.30E-04
aldo-keto reductase 5	AT1G60730	Hormone	4.07	4.74E-05
2-oxoglutarate (2OG) and Fe(II)-dependent oxygenase-like protein	AT2G30840	Hormone	3.66	6.81E-04
adenine nucleotide alpha hydrolases-like protein	AT1G09740	Hormone	3.59	1.36E-03
aluminum induced protein with YGL and LRDR motifs	AT4G27450	Hormones	3.30	1.17E-01
SAUR-like auxin-responsive protein	AT2G28085	Hormones	3.02	7.78E-01
ethylene-responsive transcription factor ERF060	AT4G39780	Hormones	2.95	3.73E-02
IAA-amino acid hydrolase ILR1	AT3G02875	Hormones	2.80	1.67E-01
BES1/BZR1 1	AT3G50750	Hormone	2.73	8.58E-03
auxin-induced in root cultures protein 12	AT3G07390	Hormone	2.57	4.17E-04
SAUR-like auxin-responsive protein	AT3G60690	Hormone	2.50	2.61E-05
gibberellin receptor GID1L2	AT3G63010	Hormone	2.42	2.46E-03
ABRE binding factor 4	AT3G19290	Hormone	2.39	1.19E-04
jacalin-like lectin domain-containing protein	AT1G52100	Hormone	2.34	5.53E-03
abscisic acid responsive element-binding factor 1	AT1G49720	Hormone	2.12	5.24E-03
auxin signaling F-box 3 protein	AT1G12820	Hormone	2.11	1.85E-02
ethylene-responsive transcription factor ERF070	AT1G71130	Hormones	2.10	2.64E-03
gibberellin receptor GID1L3	AT5G27320	Hormone	2.08	5.95E-03
dormancy/auxin associated protein	AT2G33830	Hormone	2.03	2.44E-03
SAUR-like auxin-responsive protein	AT4G36110	Hormones	2.02	2.99E-03
UDP-glucosyl transferase 71B6	AT3G21780	Hormones	1.99	0.00E+00
ferredoxin C2	AT1G32550	Hormone	-2.09	3.99E-03

12-oxophytodienoate reductase-like protein 2B	AT1G18020	Hormone	-2.14	5.15E-04
HVA22-like protein e	AT5G50720	Hormones	-2.17	1.53E-02
S-adenosyl-L-methionine-dependent methyltransferase	AT4G29590	Hormones	-2.37	2.34E-03
S-adenosyl-L-methionine-dependent methyltransferase-like protein	AT4G24805	Hormones	-2.38	3.04E-05
Ent-kaur-16-ene synthase	AT1G79460	Hormone	-2.50	2.62E-02
cytokinin oxidase/dehydrogenase 6	AT3G63440	Hormones	-2.52	5.79E-02
ethylene-responsive transcription factor ERF058	AT1G22190	Hormones	-2.59	1.40E-05
epithiospecifier protein	AT1G54040	Hormone	-2.59	5.77E-05
GRAM domain-containing protein / ABA-responsive protein-related	AT5G23350	Hormones	-2.60	4.67E-01
delta(14)-sterol reductase	AT3G52940	Hormone	-2.65	8.58E-05
S-adenosyl-L-methionine-dependent methyltransferase	AT3G21950	Hormone	-2.73	6.99E-05
SAUR-like auxin-responsive protein	AT4G00880	Hormones	-2.74	1.00E+00
uncharacterized protein	AT5G40460	Hormone	-2.86	2.10E-03
cytochrome P450 90B1 (steroid 22-alpha-hydrolase CYP90B1)	AT3G50660	Hormone	-2.90	4.24E-06
brassinosteroid metabolic pathway protein BEN1	AT2G45400	hormones	-2.95	7.92E-04
basic helix-loop-helix domain-containing protein	AT2G31730	Hormone	-2.98	3.24E-05
arabinogalactan protein 15	AT5G11740	Hormone	-3.17	4.37E-02
auxin-responsive protein IAA19	AT3G15540	Hormones	-3.17	1.40E-01
gibberellin 2-oxidase 6	AT1G02400	Hormone	-3.32	1.46E-02
auxin-responsive protein IAA17	AT1G04250	Hormones	-3.60	6.40E-03
heptahelical transmembrane protein2	AT4G30850	Hormone	-4.08	8.85E-04
S-adenosylmethionine-dependent methyltransferase domain-containing prote	AT1G69523	Hormones	-4.22	3.35E-03
auxin-responsive protein IAA5	AT1G15580	Hormones	-4.44	1.02E-02
SAUR-like auxin-responsive protein family	AT1G72430	Hormone	-4.98	7.68E-06
O-fucosyltransferase family protein	AT5G01100	Hormone	-5.94	1.79E-03
allene oxide synthase	AT5G42650	Hormone	-6.25	2.41E-02
cold and ABA inducible protein kin1	AT5G15960	Hormone	-6.60	1.03E-02
lipoxygenase 4	AT1G72520	Hormone	-7.84	9.73E-03
Col-0 2-oxoglutarate-dependent dioxygenase (AOP2) pseudogene	AT4G03060	Hormone	-9.18	3.43E-02
O-fucosyltransferase family protein	AT5G65470	Hormone	-9.32	1.74E-08
1-aminocyclopropane-1-carboxylate synthase 11 (ACS11)	AT4G08040	Hormone	-9.69	6.55E-05
indole-3-acetic acid 6	AT1G52830	Hormones	-10.55	7.20E-06
ethylene-responsive transcription factor ERF043	AT4G32800	Hormones	-11.20	6.43E-07
SAUR-like auxin-responsive protein	AT3G09870	Hormone	-13.06	5.58E-04
ethylene-responsive transcription factor ERF013	AT1G77640	Hormones	-16.37	3.87E-03
ethylene-responsive transcription factor ERF098	AT3G23230	Hormone	-17.01	9.94E-03
ethylene-responsive transcription factor ERF022	AT1G33760	Hormones	-24.81	1.01E-06
ethylene-responsive transcription factor ERF109	AT4G34410	Hormones	-28.69	1.00E+00
Late embryogenesis abundant-related protein	AT1G54890	Abiotic stress	32.14	1.72E-02
FAD-binding and BBE domain-containing protein	AT1G26410	Abiotic stress	28.31	1.18E-05
germin-like protein subfamily 1 member 19	AT5G39180	Abiotic Stress	24.06	7.31E-03
acireductone dioxygenase 3	AT2G26400	Abiotic stress	11.33	8.29E-02
mediator of RNA polymerase II transcription subunit 37b	AT1G09080	Abiotic Stress	6.95	1.12E-02
UDP-glycosyltransferase family protein	AT4G19460	Abiotic stress	4.45	8.17E-03
polyketide cyclase/dehydrase and lipid transport superfamily protein	AT1G23120	Abiotic Stress	4.34	1.24E-04
phosphopantothenoylcysteine decarboxylase	AT1G48605	Abiotic Stress	3.77	1.00E-04
bifunctional nuclease in basal defense response 1	AT1G75380	Abiotic Stress	3.12	2.75E-04
J-domain protein required for chloroplast accumulation response 1	AT1G75100	Abiotic stress	2.82	5.83E-02
uncharacterized protein	AT2G24550	Abiotic stress	2.51	1.00E+00
saposin B domain-containing protein	AT3G51730	Abiotic stress	2.17	5.79E-02
AFG1-like ATPase family protein	AT4G30490	Abiotic stress	2.08	6.46E-02
C2 domain-containing protein	AT5G55530	Abiotic stress	2.00	9.12E-03
methyltransferase PMT3	AT4G14360	Abiotic Stress	-2.04	1.21E-03
cold regulated 314 thylakoid membrane 2	AT1G29390	Abiotic stress	-2.05	1.08E-02
germin-like protein subfamily 3 member 1	AT1G72610	Abiotic Stress	-2.07	3.68E-03
glutathione S-transferase U17	AT1G10370	Abiotic stress	-2.19	1.00E+00
BCL-2-associated athanogene 1	AT5G52060	Abiotic Stress	-2.21	5.08E-03
dehydration-induced protein ERD15	AT2G41430	Abiotic Stress	-2.34	6.81E-04
dehydrin ERD14	AT1G76180	Abiotic Stress	-2.51	1.16E-02
Heat shock protein 81-3	AT2G35880	Abiotic stress	-2.56	4.95E-03
methyltransferase PMT14	AT4G18030	Abiotic Stress	-2.75	6.17E-04
protein dehydration-INDUCED 19-5	AT4G02200	Abiotic Stress	-2.83	9.36E-03
uncharacterized protein	AT2G32240	Abiotic Stress	-2.83	1.00E+00
methyltransferase PMT8	AT1G04430	Abiotic Stress	-3.07	8.07E-05
fumarate hydratase 2	AT5G50950	Abiotic stress	-3.08	1.57E-01
glutathione S-transferase tau 26	AT1G17190	Abiotic stress	-3.16	1.08E-01
chaperone DnaJ-domain containing protein	AT5G21430	Abiotic Stress	-3.34	4.57E-02
uncharacterized protein	AT1G51090	Abiotic stress	-3.86	1.27E-04
dehydrin COR47	AT1G20440	Abiotic Stress	-4.80	2.45E-05
heat shock protein class V 15.4	AT4G21870	Abiotic Stress	-5.52	2.10E-02

ERD4 protein	AT1G11960	Abiotic Stress	-5.90	6.21E-07
low-temperature-responsive protein 78/desiccation-responsive protein 29A	AT5G52310	Abiotic Stress	-6.53	3.51E-03
dehydrin ERD10	AT1G20450	Abiotic stress	-7.76	1.36E-05
abscisic acid 8'-hydroxylase 3	AT5G45340	Abiotic stress	-8.50	6.96E-07
allene oxide cyclase 1	AT3G25760	Abiotic stress	-11.54	6.92E-01
cold-regulated protein 15b	AT2G42530	Abiotic Stress	-14.86	5.08E-03
cold-regulated protein 15a	AT2G42540	Abiotic Stress	-49.46	2.46E-05
Peptin methylesterase 17 (PME17)	AT2G45220	Cell Wall	93.89	4.15E-03
extensin 3	AT1G21310	Cell Wall	37.37	9.62E-05
extensin 4	AT1G76930	Cell Wall	31.53	8.17E-03
pectin methylesterase 20 (PME20)	AT2G47550	Cell Wall	25.95	1.65E-02
mannose-6-phosphate isomerase	AT1G67070	Cell Wall	18.17	5.17E-05
caffeoyl-CoA 3-O-methyltransferase	AT1G67980	Cell wall	15.66	3.15E-06
FAD-binding and BBE domain-containing protein	AT5G44380	Cell wall	12.32	1.49E-03
polygalacturonase /pectinase	AT2G43870	Cell Wall	10.60	2.29E-02
UDP-arabinose 4-epimerase 4	AT5G44480	Cell Wall	8.68	8.15E-05
Proline-rich extensin-like family protein	AT2G43150	Cell Wall	7.70	1.36E-02
fucosyltransferase 6	AT1G14080	Cell Wall	6.02	5.02E-04
peptidoglycan-binding LysM domain-containing protein	AT5G62150	Cell Wall	5.24	1.53E-04
peroxidase 34	AT3G49120	Cell wall	4.36	1.00E+00
expansin A1	AT1G69530	Cell Wall	4.29	8.24E-05
trans-cinnamate 4-monooxygenase	AT2G30490	Cell wall	4.17	5.78E-03
Proline-rich extensin-like family protein	AT2G24980	Cell wall	3.93	6.94E-03
cinnamyl-alcohol dehydrogenase	AT1G72680	Cell wall	3.52	1.78E-02
UDP-arabinose 4-epimerase 3	AT4G20460	Cell Wall	3.24	1.11E-03
Proline-rich extensin-like family protein	AT4G08410	Cell wall	3.18	5.20E-02
lectin-like protein	AT5G03350	Cell wall	3.16	2.06E-02
xyloglucan endotransglucosylase/hydrolase protein 29	AT4G18990	Cell wall	3.15	0.00E+00
Exostosin family protein	AT3G42180	Cell wall	2.93	2.03E-01
ferulic acid 5-hydroxylase 1	AT4G36220	Cell wall	2.86	1.91E-04
glucuronidase 2	AT5G07830	Cell wall	2.82	2.32E-04
Proline-rich extensin-like family protein	AT1G23720	Cell wall	2.72	2.52E-02
uncharacterized protein	AT5G24460	Cell wall	2.05	1.71E-02
cinnamoyl coa reductase 1	AT1G15950	Cell wall	1.99	1.03E-03
LysM domain-containing GPI-anchored protein 2	AT1G77630	Cell wall	-2.00	1.83E-03
pectinacetylesterase family protein	AT3G62060	Cell Wall	-2.01	1.39E-02
uncharacterized protein	AT3G13674	Cell wall	-2.01	8.45E-02
arabinose 5-phosphate isomerase	AT3G54690	Cell Wall	-2.07	2.01E-04
mannan synthase 7	AT2G35650	Cell Wall	-2.09	3.76E-05
cinnamoyl-CoA reductase	AT1G80820	Cell wall	-2.16	2.06E-05
polygalacturonase-like protein	AT3G06770	Cell Wall	-2.24	8.50E-05
UDP-D-glucuronate 4-epimerase 6	AT3G23820	Cell Wall	-2.25	2.75E-02
glycosyl hydrolase 9B7	AT1G75680	Cell wall	-2.26	1.70E-02
pectin lyase-like superfamily protein	AT3G09540	Cell Wall	-2.30	3.82E-03
UDP-glucose 6-dehydrogenase 2	AT5G39320	Cell Wall	-2.31	1.89E-02
uncharacterized protein	AT5G01590	Cell wall	-2.36	8.38E-03
leucine-rich repeat-containing protein	AT1G33600	Cell Wall	-2.38	3.71E-02
beta-D-xylosidase 5	AT3G19620	Cell Wall	-2.39	2.45E-03
leucine-rich repeat extensin-like protein 5	AT4G18670	Cell Wall	-2.39	4.33E-03
FASCICLIN-like arabinogalactan protein 8	AT2G45470	Cell Wall	-2.48	1.71E-04
pectin methylesterase 34 (PME34)	AT3G49220	Cell Wall	-2.49	8.58E-05
pectinesterase	AT3G43270	Cell Wall	-2.56	3.00E-02
pectate lyase 1	AT1G04680	Cell Wall	-2.60	1.45E-02
peptidoglycan-binding LysM domain-containing protein	AT5G23130	Cell wall	-2.65	1.70E-06
pectinacetylesterase family protein	AT5G23870	Cell wall	-2.65	1.44E-01
extensin-like protein	AT1G12090	Cell wall	-2.73	3.72E-04
expansin A5	AT3G29030	Cell Wall	-2.80	1.35E-04
UDP-glucose 6-dehydrogenase 1	AT3G29360	Cell Wall	-2.83	5.57E-03
mannan synthase 3	AT1G23480	Cell Wall	-2.84	6.90E-06
mannose-6-phosphate isomerase	AT3G02570	Cell Wall	-2.96	1.08E-02
pectinacetylesterase family protein	AT5G23870	Cell wall	-3.03	5.08E-02
GDSL esterase/lipase	AT1G29670	Cell wall	-3.25	5.58E-04
cellulose synthase A5 (CESA 5)	AT5G09870	Cell Wall	-3.51	1.46E-02
UDP-D-glucuronate 4-epimerase 1	AT4G30440	Cell Wall	-3.70	2.63E-05
Alpha-expansin precursor	AT3G29030	Cell Wall	-3.73	4.70E-03
fasciclin-like arabinogalactan protein 7	AT2G04780	Cell Wall	-3.96	1.67E-02
fasciclin-like arabinogalactan protein 2	AT4G12730	Cell Wall	-3.98	4.05E-03
FASCICLIN-like arabinogalactan protein 18 precursor	AT3G11700	Cell Wall	-4.02	7.42E-05
pectinacetylesterase family protein	AT5G45280	Cell Wall	-4.14	2.31E-03
fasciclin-like arabinogalactan protein 9	AT1G03870	Cell Wall	-4.20	7.39E-03

GDSL esterase/lipase	AT5G14450	Cell wall	-4.25	3.99E-03
curculin-like (mannose-binding) lectin family protein	AT1G78850	Cell wall	-4.32	5.59E-02
xyloglucan glycosyltransferase 4 (Cellulose synthase-like C4)	AT3G28180	Cell Wall	-4.41	6.08E-05
esterase/lipase/thioesterase family protein	AT5G22460	Cell wall	-4.54	4.92E-01
early nodulin-like protein 17	AT5G15350	Cell wall	-4.86	1.52E-06
SKU5-like 5 protein	AT1G76160	Cell wall	-4.95	1.00E+00
protein EXORDIUM like 3	AT5G51550	Cell wall	-5.22	3.53E-03
arabinogalactan protein 20	AT3G61640	Cell Wall	-5.26	1.93E-04
peroxidase 31	AT3G28200	Cell wall	-6.10	3.40E-07
Lysine-rich arabinogalactan protein	AT2G23130	Cell Wall	-6.28	4.47E-04
arabinogalactan protein 21	AT1G55330	Cell Wall	-6.91	1.67E-03
leucine-rich repeat extensin-like protein 3	AT4G13340	Cell Wall	-7.05	1.82E-03
arabinogalactan protein 1	AT5G64310	Cell Wall	-7.24	7.83E-04
Lysine-rich arabinogalactan protein	AT2G23130	Cell Wall	-8.96	2.32E-04
uncharacterized protein	AT2G33570	Cell wall	-9.24	3.68E-06
Lysine-rich arabinogalactan protein 18	AT4G37450	Cell Wall	-9.33	5.73E-05
expansin-like A2 (EXLA2)	AT4G38400	Cell Wall	-9.45	1.67E-05
pectinesterase 25	AT3G10720	Cell Wall	-10.85	5.51E-07
uncharacterized protein	AT4G35320	Cell wall	-10.91	7.39E-07
fasciclin-like arabinogalactan protein 13	AT5G44130	Cell Wall	-11.40	2.76E-04
expansin-like A3	AT3G45960	Cell Wall	-28.69	2.24E-04
expansin-like A1	AT3G45970	Cell Wall	-43.15	1.72E-04
feruloyl CoA ortho-hydroxylase 1	AT3G13610	Cell cycle	35.21	3.33E-07
microtubule-associated protein 18	AT5G44610	Cell cycle	11.75	3.85E-02
ankyrin repeat-containing protein	AT4G14390	Cell cycle	7.17	4.14E-03
centrin 2	AT4G37010	Cell cycle	6.28	4.42E-04
ankyrin repeat family protein	AT5G54610	Cell cycle	6.24	4.20E-04
DNAse I-like superfamily protein	AT2G37440	Cell cycle	2.76	3.30E-04
MA3 domain-containing protein	AT5G63190	Cell cycle	2.40	2.34E-03
actin-related protein 8	AT5G56180	Cell cycle	2.34	1.50E-03
endonuclease/exonuclease/phosphatase family protein	AT4G36050	Cell cycle	2.24	2.95E-03
Ca-2+ dependent nuclease	AT3G56170	Cell cycle	2.15	1.13E-03
actin depolymerizing factor 9	AT4G34970	Cell cycle	2.08	1.36E-03
cell division control protein 48-A	AT3G09840	Cell cycle	2.07	1.60E-03
cyclin-dependent kinase inhibitor 4	AT2G32710	Cell cycle	2.04	8.46E-03
protein accelerated cell death 6	AT4G14400	Cell cycle	2.04	1.04E-03
membrin 11	AT2G36900	Cell cycle	2.03	1.60E-02
actin 8	AT1G49240	Cell cycle	2.03	2.80E-03
cyclin-dependent kinase inhibitor 2	AT3G50630	Cell cycle	2.01	2.64E-02
DNAse I-like superfamily protein	AT3G21530	Cell cycle	2.00	3.08E-03
ankyrin repeat-containing protein	AT3G54070	Cell cycle	-2.05	1.00E+00
protein ABIL1	AT2G46225	Cell cycle	-2.07	1.37E-02
uncharacterized protein	AT2G41830	Cell cycle	-2.08	1.47E-02
histone H3	AT1G09200	Cell cycle	-2.08	3.02E-02
cyclin-B1-4	AT2G26760	Cell cycle	-2.09	3.06E-02
Tubulin/FtsZ family protein	AT3G52750	Cell cycle	-2.12	3.45E-02
mitotic spindle checkpoint protein MAD2	AT3G25980	Cell cycle	-2.26	4.58E-03
microtubule end binding protein EB1A	AT3G47690	Cell cycle	-2.37	7.02E-04
actin binding Calponin homology domain-containing protein	AT5G55400	Cell cycle	-2.43	3.02E-02
proline-rich family protein	AT5G07020	Cell cycle	-2.47	7.49E-04
ankyrin repeat family protein	AT3G24530	Cell cycle	-2.57	9.03E-06
cyclin-B1-2	AT5G06150	Cell cycle	-2.60	2.06E-01
phloem protein 2-A11	AT1G63090	Cell cycle	-2.61	1.00E+00
transducin/WD40 domain-containing protein	AT3G51930	Cell cycle	-2.61	1.60E-03
ankyrin repeat domain-containing protein EMB506	AT5G40160	Cell cycle	-2.67	7.08E-02
3-methyladenine glycosylase I	AT5G44680	Cell cycle	-2.70	3.40E-02
Actin binding Calponin homology (CH) domain-containing protein	AT5G48460	Cell cycle	-3.14	1.13E-01
histone H3	AT5G10390	Cell cycle	-3.39	7.72E-03
copia-like retrotransposon	AT3G28160	Cell cycle	-3.55	5.92E-04
cyclin-A1-2	AT1G77390	Cell cycle	-3.91	2.12E-07
protein SCAR4	AT5G01730	Cell cycle	-4.65	1.00E+00
tetratricopeptide repeat domain-containing protein	AT3G27960	Cell cycle	-4.99	7.33E-05
Ankyrin repeat family protein	AT5G54710	Cell cycle	-5.26	1.74E-03
stress-induced protein KIN2	AT5G15970	Cell cycle	-5.83	1.23E-02
Ankyrin repeat family protein	AT2G24600	Cell cycle	-7.33	3.92E-05
histone-lysine N-methyltransferase MEDEA	AT1G02580	Development	6.35	3.90E-06
CLAVATA3/ESR (CLE)-related protein 2	AT1G63245	Development	6.07	1.75E-04
cysteine/histidine-rich C1 domain-containing protein	AT5G43520	Development	4.42	3.97E-02
nodulin MtN21 /EamA-like transporter family protein	AT5G40240	Development	3.95	8.51E-04

protein CLAVATA3/ESR-related 21	AT5G64800	Development	3.81	3.70E-05
nodulin MtN21 /EamA-like transporter family protein	AT5G40230	Development	3.67	9.44E-04
protein seedling plastid development 1	AT3G10420	Development	3.56	2.29E-02
major facilitator protein	AT2G39210	Development	3.10	3.00E-04
dormancy/auxin associated protein	AT1G56220	Development	3.05	1.63E-04
nodulin MtN21-like transporter UMAMIT38	AT4G15540	Development	2.86	1.89E-03
uncharacterized protein	AT1G74940	Development	2.62	3.20E-05
MEI2-like protein 5	AT1G29400	Development	2.46	1.64E-02
nodulin MtN21-like transporter family protein UMAMIT45	AT3G28100	Development	2.34	4.61E-02
Multiple acid move in and out transporter	AT3G28100	Development	2.16	6.94E-03
agamous-like MADS-box protein AGL15	AT5G13790	Development	-2.00	2.62E-03
seed storage albumin 1	AT4G27140	Development	-2.06	1.00E+00
Embryo-specific protein 3, (ATS3)	AT2G41475	Development	-2.12	8.55E-04
squamosa promoter-binding-like protein 2	AT5G43270	Development	-2.25	1.00E+00
protodermal factor 1	AT2G42840	Development	-2.43	1.00E+00
MAC/Perforin domain-containing protein	AT1G29690	Development	-2.43	7.68E-03
late embryogenesis abundant 3-like protein	AT1G02820	Development	-2.47	3.55E-04
methyltransferase	AT5G10830	Development	-2.73	3.69E-02
phytosulfokines 5 precursor	AT5G65870	Development	-2.77	1.64E-02
far-red-elongated hypocotyl1-like protein	AT5G02200	Development	-2.87	6.90E-05
HAD superfamily, subfamily IIIB acid phosphatase	AT5G44020	Development	-3.27	2.00E-04
protein FLOWERING LOCUS T	AT1G65480	Development	-3.28	7.86E-02
uncharacterized protein	AT4G30090	Development	-3.91	3.93E-05
desiccation-related protein LEA14	AT1G01470	Development	-4.21	2.62E-03
leucine-rich repeat receptor-like protein CLAVATA2	AT1G65380	Development	-4.52	1.10E-03
senescence/dehydration related protein	AT2G17840	Development	-5.00	2.75E-05
protein ULTRAPETALA 1	AT4G28190	Development	-5.61	0.00E+00
senescence/dehydration-associated protein	AT4G35985	Development	-6.15	1.06E-03
protein exordium like 5	AT2G17230	Development	-7.63	1.10E-06
protein exordium like 5	AT2G17230	Development	-9.25	1.01E-05
WRKY transcription factor 38	AT5G22570	DNA binding and transcriptio	16.78	0.00E+00
NAC transcription factor protein family	AT1G69490	DNA binding and transcriptio	14.23	3.38E-02
myb domain protein 122	AT1G74080	DNA binding and transcriptio	9.57	4.42E-04
WRKY transcription factor 51	AT5G64810	DNA binding and transcriptio	9.38	0.00E+00
WRKY transcription factor 61	AT1G18860	DNA binding and transcriptio	8.93	0.00E+00
WRKY transcription factor 62	AT5G01900	DNA binding and transcriptio	8.86	0.00E+00
cysteine/histidine-rich C1 domain-containing protein	AT2G42060	DNA binding and transcriptio	8.73	2.36E-04
WRKY DNA-binding protein 31	AT4G22070	DNA binding and transcriptio	8.26	0.00E+00
WRKY transcription factor 8	AT5G46350	DNA binding and transcriptio	7.19	0.00E+00
myb family transcription factor	AT1G71030	DNA binding and transcriptio	6.40	2.73E-05
WRKY transcription factor 71	AT1G29860	DNA binding and transcriptio	5.55	0.00E+00
WRKY transcription factor 63	AT1G66600	DNA binding and transcriptio	5.48	3.68E-04
WRKY transcription factor 72	AT5G15130	DNA binding and transcriptio	5.32	0.00E+00
myb domain protein 13	AT1G06180	DNA binding and transcriptio	5.28	6.56E-04
myb domain protein 45	AT3G48920	DNA binding and transcriptio	5.27	2.62E-04
LOB domain-containing protein 21	AT3G11090	DNA binding and transcriptio	4.97	1.98E-04
PLATZ transcription factor family protein	AT1G76590	DNA binding and transcriptio	4.89	1.69E-02
WRKY transcription factor 41	AT4G11070	DNA binding and transcriptio	4.61	0.00E+00
zinc finger CCCH domain-containing protein 49	AT4G29190	DNA binding and transcriptio	4.57	3.97E-07
WRKY transcription factor 50	AT5G26170	DNA binding and transcriptio	4.56	0.00E+00
PLATZ transcription factor family protein	AT1G21000	DNA binding and transcriptio	3.96	4.72E-04
LOB domain-containing protein 1	AT1G07900	DNA binding and transcriptio	3.45	6.22E-02
homeobox-leucine zipper protein HAT9	AT2G22800	DNA binding and transcriptio	3.18	3.23E-03
homeobox protein BEL1-like protein	AT5G41410	DNA binding and transcriptio	3.11	4.28E-04
homeobox-leucine zipper protein ATHB-21	AT2G18550	DNA binding and transcriptio	2.95	1.65E-02
myb domain protein 85	AT4G22680	DNA binding and transcriptio	2.93	3.57E-04
transcription factor MYB3	AT1G22640	DNA binding and transcriptio	2.83	1.66E-04
AP2/B3-like transcriptional factor family protein	AT3G11580	DNA binding and transcriptio	2.78	3.21E-02
plant regulator RWP-RK family protein	AT4G35270	DNA binding and transcriptio	2.75	7.10E-03
myb domain protein 78	AT5G49620	DNA binding and transcriptio	2.74	1.32E-01
B3 DNA-binding domain transcription factor	AT2G36080	DNA binding and transcriptio	2.69	3.38E-01
WRKY transcription factor 66	AT1G80590	DNA binding and transcriptio	2.59	0.00E+00
E2F/DP family winged-helix DNA-binding domain-containing protein	AT4G18870	DNA binding and transcriptio	2.57	8.79E-03
basic leucine zipper 9	AT5G24800	DNA binding and transcriptio	2.52	7.76E-03
GATA transcription factor 27	AT5G47140	DNA binding and transcriptio	2.48	1.44E-04
transcription factor HY5-like protein	AT3G17609	DNA binding and transcriptio	2.47	3.19E-03
MEI2-like 2 protein	AT2G42890	DNA binding and transcriptio	2.45	1.21E-01
transcription factor bHLH61	AT5G10570	DNA binding and transcriptio	2.43	3.41E-03
cycling DOF factor 2	AT5G39660	DNA binding and transcriptio	2.43	3.96E-04
transcription factor HY5	AT5G11260	DNA binding and transcriptio	2.41	2.27E-02

phospholipid-transporting ATPase 9	AT1G68710	DNA binding and transcriptio	2.36	1.25E-02
transcription factor bHLH66	AT2G24260	DNA binding and transcriptio	2.36	4.96E-02
Calcium-dependent lipid-binding (CaLB domain) family protein	AT1G70810	DNA binding and transcriptio	2.31	8.15E-05
SNF2 , helicase and zinc-finger domain-containing protein	AT1G11100	DNA binding and transcriptio	2.30	1.39E-02
homeobox-leucine zipper protein ATHB-4	AT2G44910	DNA binding and transcriptio	2.27	7.58E-04
PLATZ transcription factor domain-containing protein	AT1G32700	DNA binding and transcriptio	2.26	1.04E-05
transcription factor bHLH148	AT3G06590	DNA binding and transcriptio	2.06	8.83E-03
myb domain protein 14	AT2G31180	DNA binding and transcriptio	2.06	1.00E+00
WRKY transcription factor 60	AT2G25000	DNA binding and transcriptio	2.05	0.00E+00
oxidation-related zinc Finger 1	AT2G19810	DNA binding and transcriptio	2.01	1.95E-02
myb family transcription factor	AT3G16350	DNA binding and transcriptio	-2.01	2.66E-03
chloroplast RNA-binding protein 33	AT3G52380	DNA binding and transcriptio	-2.02	2.19E-02
DNA-binding storekeeper protein-related transcriptional regulator	AT4G25210	DNA binding and transcriptio	-2.05	2.41E-02
chloroplast RNA-binding protein 31B	AT5G50250	DNA binding and transcriptio	-2.11	2.79E-03
Cyclin/Brf1-like TBP-binding protein	AT2G45100	DNA binding and transcriptio	-2.16	5.12E-02
agamous-like MADS-box protein AGL3	AT2G03710	DNA binding and transcriptio	-2.19	1.33E-01
WRKY DNA-binding protein 30	AT5G24110	DNA binding and transcriptio	-2.23	0.00E+00
WUSCHEL-related homeobox 4	AT1G46480	DNA binding and transcriptio	-2.25	0.00E+00
ribonuclease III-like protein	AT1G55140	DNA binding and transcriptio	-2.28	1.28E-02
WUSCHEL-related homeobox 2	AT5G59340	DNA binding and transcriptio	-2.32	0.00E+00
myb domain protein 76	AT5G07700	DNA binding and transcriptio	-2.33	1.16E-02
RNA recognition motif-containing protein	AT1G67950	DNA binding and transcriptio	-2.34	3.56E-03
ribosome-binding factor A	AT4G34730	DNA binding and transcriptio	-2.40	1.62E-03
homeobox-leucine zipper protein ATHB-54	AT1G27045	DNA binding and transcriptio	-2.45	1.00E+00
Rossmann-fold NAD(P)-binding domain-containing protein	AT4G35250	DNA binding and transcriptio	-2.49	2.54E-02
DREB subfamily A-4 of ERF/AP2 transcription factor	AT1G63040	DNA binding and transcriptio	-2.62	5.67E-02
transcription factor TCP9	AT2G45680	DNA binding and transcriptio	-2.64	6.50E-02
RNA methyltransferase	AT5G10620	DNA binding and transcriptio	-2.69	1.22E-02
ethylene-responsive transcription factor ERF061	AT1G64380	DNA binding and transcriptio	-2.79	2.17E-02
bZIP protein	AT5G04840	DNA binding and transcriptio	-2.80	3.64E-04
myb domain protein 77	AT3G50060	DNA binding and transcriptio	-2.84	2.03E-05
transcription factor bHLH96	AT1G72210	DNA binding and transcriptio	-2.88	5.15E-04
chloroplast stem-loop binding protein-41	AT3G63140	DNA binding and transcriptio	-2.91	8.21E-02
protein AGAMOUS-like 87	AT1G22590	DNA binding and transcriptio	-3.12	8.54E-03
activation-tagged BRI1 suppressor 1-interacting factor 1	AT3G05800	DNA binding and transcriptio	-3.19	1.50E-02
homeodomain-like transcriptional regulator	AT5G58900	DNA binding and transcriptio	-3.25	5.95E-03
RNA recognition motif-containing protein	AT1G22330	DNA binding and transcriptio	-3.27	1.25E-06
RNA recognition motif-containing protein	AT1G03457	DNA binding and transcriptio	-3.46	1.25E-03
transcription factor jumonji family protein / zinc finger (C5HC2 type) family pr	AT5G46910	DNA binding and transcriptio	-3.47	1.67E-04
zinc finger transcription factor BZS1	AT4G39070	DNA binding and transcriptio	-3.53	3.43E-03
myb domain protein 50	AT1G57560	DNA binding and transcriptio	-3.65	1.00E+00
two-component response regulator ARR15	AT1G74890	DNA binding and transcriptio	-3.86	4.13E-02
Myb transcription factor	AT5G62470	DNA binding and transcriptio	-3.98	6.26E-04
homeobox-leucine zipper protein HAT1	AT4G17460	DNA binding and transcriptio	-4.26	9.36E-04
two-component response regulator ARR6	AT5G62920	DNA binding and transcriptio	-4.52	6.47E-04
myb domain protein 29	AT5G07690	DNA binding and transcriptio	-4.76	4.34E-01
two-component response regulator ARR7	AT1G19050	DNA binding and transcriptio	-4.93	7.47E-03
PLATZ transcription factor family protein	AT1G43000	DNA binding and transcriptio	-5.17	3.47E-03
GATA transcription factor 8	AT3G54810	DNA binding and transcriptio	-5.55	2.32E-08
heat shock transcription factor A3	AT5G03720	DNA binding and transcriptio	-6.79	1.96E-05
U-box domain-containing protein	AT1G66160	DNA binding and transcriptio	-7.60	7.53E-04
dual transcription unit and alternative splicing protein GLAUCE	AT1G65450	DNA binding and transcriptio	-8.17	3.31E-01
transcription factor bHLH137	AT5G50915	DNA binding and transcriptio	-9.29	9.88E-07
ethylene-responsive transcription factor ERF012	AT1G21910	DNA binding and transcriptio	-57.97	1.38E-05
ethylene-responsive transcription factor ERF019	AT1G22810	DNA binding and transcriptio	-71.44	2.99E-06
photosystem I reaction center subunit psaK	AT1G30380	Light	-2.07	1.00E+00
protein plastid transcriptionally active 16	AT3G46780	Light	-2.10	1.00E+00
cofactor assembly, complex C (B6F)	AT5G36120	Light	-2.14	2.46E-01
carboxylesterase 6	AT1G68620	Metabolism	42.79	1.13E-02
methionine sulfoxide reductase B8	AT4G21840	Metabolism	22.15	4.20E-04
Terpenoid cyclases family protein	AT1G66960	Metabolism	12.85	3.82E-04
glutamate decarboxylase 1	AT5G17330	Metabolism	10.33	1.52E-04
flavonol synthase 5	AT5G63600	Metabolism	9.95	4.33E-03
aldehyde oxidase 1	AT5G20960	Metabolism	8.98	1.33E-06
Inositol monophosphatase family protein	AT5G09290	Metabolism	8.70	3.87E-04
HXXXD-type acyl-transferase-like protein	AT5G42830	Metabolism	8.34	4.78E-05
branched-chain-amino-acid aminotransferase 2	AT1G10070	Metabolism	7.65	1.78E-04
phosphoglycerate mutase-like protein	AT1G09935	Metabolism	7.11	4.05E-04
lipid-transfer protein/seed storage	AT3G22620	Metabolism	6.91	2.69E-02

beta-fructofuranosidase, insoluble isoenzyme CWINV1	AT3G13790	Metabolism	5.92	8.28E-05
2-oxoglutarate (2OG) and Fe(II)-dependent oxygenase superfamily protein	AT2G36690	Metabolism	5.89	7.36E-04
methionine gamma-lyase	AT1G64660	Metabolism	5.58	7.67E-02
long-chain acyl-CoA synthetase	AT1G64400	Metabolism	5.38	2.48E-05
glycosyl hydrolase 9A2	AT1G65610	Metabolism	5.31	6.73E-03
GDSL esterase/lipase	AT5G03610	Metabolism	5.20	3.53E-03
tryptophan N-monooxygenase 1	AT4G39950	Metabolism	5.15	3.55E-03
alpha 1,4-glycosyltransferase family protein	AT5G01250	Metabolism	4.95	1.79E-03
glutamine synthetase 1;1	AT5G37600	Metabolism	4.78	1.23E-07
GDSL esterase/lipase 4	AT3G14225	Metabolism	4.68	5.88E-04
beta-galactosidase 4	AT5G56870	Metabolism	4.57	3.12E-04
glycolate oxidase	AT4G18360	Metabolism	3.72	3.18E-03
glucuronidase 1	AT5G61250	Metabolism	3.66	1.46E-05
UDP-glycosyltransferase 76E4	AT3G46690	Metabolism	3.57	8.64E-04
pyruvate kinase	AT5G63680	Metabolism	3.54	1.33E-06
flavodoxin-like quinone reductase 1	AT5G54500	Metabolism	3.54	5.69E-05
phospholipase A1-Igamma2	AT2G30550	Metabolism	3.21	1.05E-04
copper amine oxidase	AT3G43670	Metabolism	3.13	2.79E-03
O-methyltransferase-like protein	AT1G33030	Metabolism	2.98	4.38E-04
lysophospholipase 2	AT1G52760	Metabolism	2.91	1.87E-05
D-3-phosphoglycerate dehydrogenase	AT4G34200	Metabolism	2.84	9.79E-05
aldose 1-epimerase	AT3G47800	Metabolism	2.81	4.25E-03
calcium-independent phospholipase A	AT4G19860	Metabolism	2.80	2.78E-05
Long-chain-alcohol oxidase FAO4B	AT4G28570	Metabolism	2.80	2.36E-03
nudix hydrolase 18	AT1G14860	Metabolism	2.79	1.00E+00
Sphingosine-1-phosphate lyase	AT1G27980	Metabolism	2.78	1.14E-04
cytochrome P450, family 706, subfamily A, polypeptide 4	AT4G12300	Metabolism	2.74	1.78E-03
acyl-coenzyme A oxidase 4	AT3G51840	Metabolism	2.68	3.62E-03
adenine nucleotide alpha hydrolases-domain containing protein kinase	AT1G77280	Metabolism	2.55	6.36E-03
pfkB-like carbohydrate kinase family protein	AT5G43910	Metabolism	2.54	4.85E-04
metallo-beta-lactamase family protein	AT4G33540	Metabolism	2.51	1.31E-01
SAL3 phosphatase	AT5G63990	Metabolism	2.49	1.93E-03
HXXXD-type acyl-transferase family protein	AT1G28680	Metabolism	2.44	3.22E-03
copper amine oxidase 1	AT1G62810	Metabolism	2.37	7.11E-04
UDP-glycosyltransferase-like protein	AT2G18560	Metabolism	2.37	3.46E-02
strigolactone esterase D14	AT3G03990	Metabolism	2.36	4.54E-03
trehalose-phosphate phosphatase D	AT1G35910	Metabolism	2.36	3.39E-01
acyl activating enzyme 5	AT5G16370	Metabolism	2.35	7.02E-03
tetraketide alpha-pyrone reductase 2-like protein	AT1G25460	Metabolism	2.33	6.57E-02
copper amine oxidase 1	AT1G62810	Metabolism	2.30	1.52E-03
metallo-beta-lactamase family protein	AT4G33540	Metabolism	2.30	1.90E-01
GDSL esterase/lipase	AT1G28580	Metabolism	2.29	1.89E-02
P-loop containing nucleoside triphosphate hydrolases superfamily protein	AT1G04280	Metabolism	2.25	3.38E-04
GDSL esterase/lipase	AT2G38180	Metabolism	2.25	7.28E-01
alkaline/neutral invertase CINV1	AT1G35580	Metabolism	2.25	1.38E-03
phospholipase A(1) LCAT3	AT3G03310	Metabolism	2.19	1.40E-01
melibiase family protein	AT3G56310	Metabolism	2.18	3.77E-03
glutathione S-conjugate transporting ATPase	AT1G30400	Metabolism	2.17	1.17E-02
Hydrolases superfamily protein	AT4G00500	Metabolism	2.16	5.15E-04
Cyclopropane-fatty-acyl-phospholipid synthase	AT3G23530	Metabolism	2.16	3.67E-02
aldehyde dehydrogenase 2B4	AT3G48000	Metabolism	2.14	2.03E-02
NAD(P)-binding Rossmann-fold superfamily protein	AT5G52810	Metabolism	2.14	6.21E-05
stearoyl-acyl-carrier-protein desaturase	AT5G16230	Metabolism	2.13	1.97E-02
alpha-galactosidase 1	AT5G08380	Metabolism	2.08	2.01E-02
EPSP synthase	AT2G45300	Metabolism	2.07	3.90E-03
glycine-rich protein / oleosin	AT5G56100	Metabolism	2.07	1.00E+00
Class-II DAHP synthetase family protein	AT1G22410	Metabolism	2.05	1.58E-02
fatty acid binding protein 2	AT2G26310	Metabolism	2.04	1.25E-02
UDP-glycosyltransferase 72B1	AT4G01070	Metabolism	2.04	3.35E-02
digalactosyldiacylglycerol synthase 1	AT3G11670	Metabolism	2.04	2.83E-03
cytokinin riboside 5'-monophosphate phosphoribohydrolase LOG7	AT5G06300	Metabolism	-2.00	1.35E-02
adenosine kinase 1	AT3G09820	Metabolism	-2.01	2.05E-03
uncharacterized protein	AT2G46890	Metabolism	-2.02	4.52E-01
P-loop containing nucleoside triphosphate hydrolases superfamily protein	AT3G01820	Metabolism	-2.03	6.85E-02
acyl carrier protein 4	AT4G25050	Metabolism	-2.03	3.27E-02
3-ketoacyl-CoA synthase 5	AT1G25450	Metabolism	-2.04	2.98E-01
tRNA pseudouridine synthase	AT2G30320	Metabolism	-2.06	2.36E-03
glutamine synthetase	AT5G35630	Metabolism	-2.06	3.30E-02
glutamate--glyoxylate aminotransferase 2	AT1G70580	Metabolism	-2.07	5.21E-01
dihydrolipoyl dehydrogenase	AT4G16155	Metabolism	-2.09	9.06E-04
2Fe-2S iron-sulfur cluster binding domain-containing protein	AT4G32590	Metabolism	-2.10	4.05E-02

aminomethyltransferase	AT1G11860	Metabolism	-2.11	1.03E-01
1-(5-phosphoribosyl)-5-	AT2G36230	Metabolism	-2.14	2.08E-02
isoleucyl-tRNA synthetase	AT5G49030	Metabolism	-2.14	1.00E+00
phosphoglycerate kinase 1	AT3G12780	Metabolism	-2.15	1.00E+00
bifunctional aspartokinase/homoserine dehydrogenase 1	AT1G31230	Metabolism	-2.15	1.37E-03
adenylate kinase family protein	AT5G35170	Metabolism	-2.16	1.19E-01
NAD(P)H:plastoquinone dehydrogenase complex subunit O	AT1G74880	Metabolism	-2.17	1.00E+00
glyceraldehyde-3-phosphate dehydrogenase (NADP+) (phosphorylating)	AT1G12900	Metabolism	-2.18	5.59E-02
2Fe-2S iron-sulfur cluster binding domain-containing protein	AT4G32590	Metabolism	-2.18	2.69E-02
NAD(P)-linked oxidoreductase superfamily protein	AT2G27680	Metabolism	-2.18	6.53E-02
mevalonate kinase	AT5G27450	Metabolism	-2.18	2.67E-02
esterase/lipase/thioesterase family protein	AT3G50790	Metabolism	-2.18	2.73E-04
pfkB-like carbohydrate kinase family protein	AT1G66430	Metabolism	-2.19	1.98E-03
3-ketoacyl-CoA synthase 10	AT2G26250	Metabolism	-2.19	1.27E-01
aminotransferase	AT1G77670	Metabolism	-2.20	2.75E-05
Enoyl-ACP reductase 1	AT2G05990	Metabolism	-2.24	1.16E-02
ATP synthase protein I-related protein	AT2G31040	Metabolism	-2.24	4.04E-01
branched-chain-amino-acid aminotransferase 3	AT3G49680	Metabolism	-2.28	2.71E-03
GDSL esterase/lipase	AT5G45920	Metabolism	-2.29	2.96E-02
myo-inositol-1-phosphate synthase	AT5G10170	Metabolism	-2.31	1.00E+00
phosphoglycerate mutase family protein	AT5G62840	Metabolism	-2.31	1.60E-03
glycine dehydrogenase	AT4G33010	Metabolism	-2.32	2.92E-02
trehalose-phosphate phosphatase H	AT4G39770	Metabolism	-2.35	1.49E-03
myo-inositol monophosphatase like 1	AT1G31190	Metabolism	-2.35	1.88E-04
myo-inositol monophosphatase like 1	AT1G31190	Metabolism	-2.35	4.21E-02
photosystem II reaction center PsbP family protein	AT1G76450	Metabolism	-2.36	6.17E-04
2-phosphoglycolate phosphatase 1	AT5G36700	Metabolism	-2.39	1.00E+00
fructokinase-like 1	AT3G54090	Metabolism	-2.40	9.28E-03
nucleotide-diphospho-sugar transferase	AT1G64980	Metabolism	-2.40	3.93E-05
1-deoxy-D-xylulose 5-phosphate reductoisomerase	AT5G62790	Metabolism	-2.42	1.58E-03
glycine dehydrogenase	AT4G33010	Metabolism	-2.43	2.23E-01
low psii accumulation2 protein	AT5G51545	Metabolism	-2.46	1.27E-03
photosystem I light harvesting complex protein 5	AT1G45474	Metabolism	-2.47	7.27E-03
1,4-alpha-glucan branching enzyme	AT5G03650	Metabolism	-2.48	8.87E-03
glucan endo-1,3-beta-glucosidase 11	AT1G32860	Metabolism	-2.48	2.49E-05
glutamate-1-semialdehyde 2,1-aminomutase 2	AT3G48730	Metabolism	-2.49	6.94E-04
fructose-1,6-bisphosphatase	AT3G54050	Metabolism	-2.50	1.81E-01
bifunctional sn-glycerol-3-phosphate 2-O-acyltransferase/phosphatase	AT4G00400	Metabolism	-2.50	1.60E-03
3-hydroxyacyl-ACP dehydratase	AT2G22230	Metabolism	-2.51	1.46E-04
alpha-galactosidase 2	AT5G08370	Metabolism	-2.52	3.75E-01
NDH-dependent cyclic electron flow 1	AT3G16250	Metabolism	-2.52	1.00E+00
PsbP domain-containing protein 5	AT5G11450	Metabolism	-2.57	6.47E-04
cysteine synthase 26	AT3G03630	Metabolism	-2.59	2.26E-03
HXXXD-type acyl-transferase-like protein	AT2G39980	Metabolism	-2.62	2.53E-04
glucose-1-phosphate adenylyltransferase small subunit	AT5G48300	Metabolism	-2.63	1.17E-02
NAD(P)-linked oxidoreductase superfamily protein	AT1G04420	Metabolism	-2.65	4.54E-03
phosphomethylpyrimidine synthase	AT2G29630	Metabolism	-2.65	0.00E+00
aldose 1-epimerase family protein	AT5G66530	Metabolism	-2.67	1.48E-03
chloroplast thylakoid lumen protein	AT4G02530	Metabolism	-2.70	3.53E-04
2-C-methyl-D-erythritol 4-phosphate cytidylyltransferase	AT2G02500	Metabolism	-2.73	1.35E-03
carboxyvinyl-carboxyphosphonate phosphorylmutase	AT1G77060	Metabolism	-2.77	2.64E-06
lipase domain-containing protein	AT5G50890	Metabolism	-2.82	2.05E-04
fatty acyl-CoA reductase 1	AT5G22500	Metabolism	-2.87	8.32E-05
Monogalactosyldiacylglycerol synthase 1	AT4G31780	Metabolism	-2.89	1.76E-02
hexokinase-like 1	AT1G50460	Metabolism	-2.95	1.72E-04
tropinone reductase-like protein	AT2G29290	Metabolism	-3.00	3.21E-02
fatty acid desaturase 7	AT3G11170	Metabolism	-3.04	1.67E-03
fatty acid hydroxylase 1	AT2G34770	Metabolism	-3.08	3.60E-03
Flavonoid 3'-monooxygenase	AT5G07990	Metabolism	-3.13	0.00E+00
alpha-L-fucosidase 2	AT4G34260	Metabolism	-3.13	7.70E-04
galacturonosyltransferase-like 6	AT4G02130	Metabolism	-3.15	3.21E-04
FAD/NAD(P)-binding oxidoreductase domain-containing protein	AT1G57770	Metabolism	-3.16	9.53E-05
adenine phosphoribosyl transferase 3	AT4G22570	Metabolism	-3.22	3.53E-03
galacturonosyltransferase 15	AT3G58790	Metabolism	-3.23	7.86E-06
3-ketoacyl-CoA synthase 20	AT5G43760	Metabolism	-3.24	3.48E-03
UDP-glycosyltransferase family protein	AT5G04480	Metabolism	-3.30	1.11E-04
3-isopropylmalate dehydrogenase 1	AT1G31180	Metabolism	-3.32	2.15E-01
Type I inositol-1,4,5-trisphosphate 5-phosphatase 2	AT4G18010	Metabolism	-3.32	6.77E-03
fatty acid hydroxylase 1	AT2G34770	Metabolism	-3.37	9.29E-06
adenosylhomocysteinase 2	AT3G23810	Metabolism	-3.40	1.00E+00
cell wall / vacuolar inhibitor of fructosidase 2	AT5G64620	Metabolism	-3.50	2.68E-04

phosphoenolpyruvate carboxylase family protein	AT1G21440	Metabolism	-3.53	2.45E-02
plant glycogenin-like starch initiation protein 7	AT2G35710	Metabolism	-3.54	7.87E-07
fatty-acid-binding protein 3	AT1G53520	Metabolism	-3.54	2.28E-02
Haloacid dehalogenase-like hydrolase (HAD) superfamily protein	AT3G48420	Metabolism	-3.70	1.63E-01
fatty-acid-binding protein 3	AT1G53520	Metabolism	-3.84	5.87E-02
galacturonosyltransferase-like 8	AT1G24170	Metabolism	-3.86	3.33E-07
RNA-directed DNA methylase	AT1G13790	Metabolism	-3.96	5.62E-03
trehalose-phosphate phosphatase-like protein	AT5G51460	Metabolism	-4.18	5.08E-08
Chalcone-flavanone isomerase family protein	AT5G05270	Metabolism	-4.48	5.37E-03
isoprenylcysteine alpha-carbonyl methylesterase ICMEL2	AT3G02410	Metabolism	-4.57	4.76E-02
3-ketoacyl-CoA synthase 4	AT1G19440	Metabolism	-4.58	2.90E-05
delta-9 desaturase-like 5 protein	AT1G06360	Metabolism	-4.61	1.55E-01
fatty acid desaturase 8	AT5G05580	Metabolism	-4.64	1.02E-02
galacturonosyltransferase-like 9	AT1G70090	Metabolism	-4.92	3.19E-09
acyl-CoA sterol acyl transferase 1	AT3G51970	Metabolism	-5.21	2.87E-04
isopropylmalate dehydrogenase 1	AT5G14200	Metabolism	-5.23	2.98E-02
GDSL esterase/lipase	AT4G28780	Metabolism	-5.34	4.80E-02
cytochrome P450 83A1	AT4G13770	Metabolism	-5.40	5.14E-01
flavin-containing monooxygenase FMO GS-OX3	AT1G62560	Metabolism	-5.43	2.04E-03
sodium/metabolite cotransporter BASS5	AT4G12030	Metabolism	-5.68	3.07E-02
oxidoreductase	AT3G60290	Metabolism	-5.78	3.79E-05
O-glycosyl hydrolases family 17 protein	AT1G64760	Metabolism	-6.02	1.09E-06
phosphatidylinositol:ceramide inositolphosphotransferase 1	AT3G54020	Metabolism	-6.10	2.27E-06
S-adenosylmethionine synthase 4	AT3G17390	Metabolism	-6.44	3.08E-05
isopropylmalate isomerase 1	AT3G58990	Metabolism	-6.95	4.92E-02
peptide methionine sulfoxide reductase B5	AT4G04830	Metabolism	-7.20	7.14E-01
homocysteine S-methyltransferase 3	AT3G22740	Metabolism	-7.37	8.37E-02
delta-9 acyl-lipid desaturase 1	AT1G06080	Metabolism	-8.05	1.00E+00
chalcone synthase	AT5G13930	Metabolism	-8.41	0.00E+00
3-ketoacyl-CoA synthase 1	AT1G01120	Metabolism	-8.42	9.03E-06
isopropylmalate isomerase 2	AT2G43100	Metabolism	-8.59	7.31E-03
inositol oxygenase 1	AT1G14520	Metabolism	-9.54	5.43E-08
methionine sulfoxide reductase B6	AT4G04840	Metabolism	-9.67	1.04E-03
methylthioalkylmalate synthase 1	AT5G23010	Metabolism	-10.00	2.51E-02
flavin-containing monooxygenase FMO GS-OX1	AT1G65860	Metabolism	-10.32	3.94E-04
glutathione S-transferase TAU 20	AT1G78370	Metabolism	-11.00	6.43E-04
cytidine/deoxycytidylate deaminase-like protein	AT4G29610	Metabolism	-11.80	3.13E-05
branched-chain aminotransferase4	AT3G19710	Metabolism	-16.76	1.41E-01
sn-glycerol-3-phosphate 2-O-acyltransferase	AT4G01950	Metabolism	-17.58	2.13E-07
dihomomethionine N-hydroxylase	AT1G16410	Metabolism	-19.44	7.12E-02
aspartyl protease family protein	AT5G48430	Protein synthesis, modificatio	41.55	3.86E-05
aspartyl protease family protein	AT1G44130	Protein synthesis, modificatio	27.44	8.22E-07
U-box domain-containing protein 36	AT3G61390	Protein synthesis, modificatio	12.09	7.27E-08
matrix metalloproteinase	AT1G70170	Protein synthesis, modificatio	9.96	4.24E-04
C3H4 type zinc finger protein	AT5G49665	Protein synthesis, modificatio	7.64	1.60E-01
exocyst subunit exo70 family protein H2	AT2G39380	Protein synthesis, modificatio	7.60	2.79E-04
chaperone protein dnaJ 11	AT4G36040	Protein synthesis, modificatio	7.51	6.60E-04
F-box/kelch-repeat protein	AT2G44130	Protein synthesis, modificatio	6.98	6.73E-03
E3 ubiquitin-protein ligase ATL41	AT2G42360	Protein synthesis, modificatio	6.09	1.11E-04
calcium-binding protein CML21	AT4G26470	Protein synthesis, modificatio	5.91	6.00E-04
concanavalin A-like lectin kinase-like protein	AT3G45410	Protein synthesis, modificatio	5.71	1.43E-03
F-box/kelch-repeat protein	AT1G80440	Protein synthesis, modificatio	5.49	1.82E-05
vacuolar sorting receptor 6	AT1G30900	Protein synthesis, modificatio	5.35	0.00E+00
aspartyl protease family protein	AT3G51330	Protein synthesis, modificatio	5.03	1.99E-05
F-box protein	AT3G44326	Protein synthesis, modificatio	4.31	5.79E-02
subtilase family protein	AT1G32950	Protein synthesis, modificatio	4.15	8.95E-02
AAA-type ATPase family protein	AT5G57480	Protein synthesis, modificatio	4.00	3.13E-03
AAA-ATPase 1	AT5G40010	Protein synthesis, modificatio	3.97	1.13E-03
ubiquitin-like protein	AT1G53980	Protein synthesis, modificatio	3.90	9.16E-03
aspartyl protease family protein	AT5G10760	Protein synthesis, modificatio	3.67	4.66E-03
uncharacterized protein	AT3G01175	Protein synthesis, modificatio	3.50	4.85E-04
ubiquitin 13	AT1G65350	Protein synthesis, modificatio	3.36	0.00E+00
RING-H2 finger protein ATL79	AT5G47610	Protein synthesis, modificatio	3.09	1.24E-03
P-loop containing nucleoside triphosphate hydrolases superfamily protein	AT5G17760	Protein synthesis, modificatio	3.01	5.66E-04
RING-H2 finger protein ATL70	AT2G35910	Protein synthesis, modificatio	3.00	1.44E-04
transmembrane Fragile-X-F-associated protein	AT1G68820	Protein synthesis, modificatio	2.97	4.84E-02
RING/U-box domain-containing protein	AT4G26580	Protein synthesis, modificatio	2.89	1.26E-02
RING/U-box domain-containing protein	AT5G55970	Protein synthesis, modificatio	2.87	7.66E-04
adenine nucleotide alpha hydrolases-domain containing protein kinase	AT1G21590	Protein synthesis, modificatio	2.86	5.41E-03
autophagy-related protein 8a	AT4G21980	Protein synthesis, modificatio	2.85	8.33E-03

histone H3 K4-specific methyltransferase SET7/9 family protein	AT1G77660	Protein synthesis, modificatio	2.80	6.81E-03
F-box stress induced protein 2	AT4G21510	Protein synthesis, modificatio	2.69	4.43E-05
Unknown	AT1G18670	Protein synthesis, modificatio	2.67	1.02E-02
vacuolar-processing enzyme gamma	AT4G32940	Protein synthesis, modificatio	2.66	8.45E-05
F-box protein SKP2B	AT1G77000	Protein synthesis, modificatio	2.66	1.00E+00
F-box domain-containing protein	AT5G18780	Protein synthesis, modificatio	2.59	9.64E-06
autophagy substrate NBR1	AT4G24690	Protein synthesis, modificatio	2.59	6.56E-04
RING/U-box superfamily protein	AT5G24870	Protein synthesis, modificatio	2.58	3.12E-03
RING/U-box domain-containing protein	AT1G63840	Protein synthesis, modificatio	2.55	4.01E-04
ubiquitin 4	AT5G20620	Protein synthesis, modificatio	2.53	0.00E+00
vacuolar-processing enzyme gamma	AT4G32940	Protein synthesis, modificatio	2.53	2.12E-04
F-box/kelch-repeat protein	AT3G23880	Protein synthesis, modificatio	2.51	2.35E-04
RING/FYVE/PHD zinc finger-containing protein	AT2G37950	Protein synthesis, modificatio	2.50	1.72E-03
uncharacterized protein	AT1G21670	Protein synthesis, modificatio	2.49	6.77E-03
E3 ubiquitin-protein ligase ARI12	AT1G05880	Protein synthesis, modificatio	2.48	1.16E-02
cathepsin B-like cysteine protease	AT4G01610	Protein synthesis, modificatio	2.46	8.15E-05
E3 ubiquitin-protein ligase ARI5	AT1G05890	Protein synthesis, modificatio	2.44	2.98E-03
E3 ubiquitin-protein ligase ARI5	AT1G05890	Protein synthesis, modificatio	2.44	2.28E-02
RING/U-box domain-containing protein	AT1G24440	Protein synthesis, modificatio	2.41	6.28E-03
aspartyl protease family protein	AT3G51360	Protein synthesis, modificatio	2.33	1.80E-03
ARM repeat superfamily protein	AT5G67340	Protein synthesis, modificatio	2.33	3.52E-04
F-box/kelch-repeat protein	AT1G51550	Protein synthesis, modificatio	2.33	1.45E-04
subtilase 4.12	AT5G59090	Protein synthesis, modificatio	2.33	1.00E+00
RING/U-box domain-containing protein	AT4G19670	Protein synthesis, modificatio	2.32	6.73E-03
F-box/kelch-repeat protein	AT1G23390	Protein synthesis, modificatio	2.29	1.29E-04
F-box/kelch-repeat protein	AT3G24760	Protein synthesis, modificatio	2.28	2.93E-03
RING/U-box domain-containing protein	AT3G47160	Protein synthesis, modificatio	2.26	3.01E-03
peptidase C15, pyroglutamyl peptidase I-like protein	AT1G23440	Protein synthesis, modificatio	2.22	2.03E-03
atypical dual-specificity phosphatase	AT2G32960	Protein synthesis, modificatio	2.20	5.79E-02
aspartyl protease family protein	AT3G02740	Protein synthesis, modificatio	2.20	2.52E-03
beta-1,3-galactosyltransferase 7	AT1G77810	Protein synthesis, modificatio	2.18	1.15E-05
F-box/RNI-like superfamily protein	AT5G67140	Protein synthesis, modificatio	2.15	3.86E-01
RING/U-box domain-containing protein	AT3G06330	Protein synthesis, modificatio	2.10	8.43E-04
C3H2C3-type RING E3 Ub ligase	AT4G23450	Protein synthesis, modificatio	2.08	8.01E-03
histone deacetylase 8	AT1G08460	Protein synthesis, modificatio	2.07	2.34E-04
ubiquitin-associated (UBA)/TS-N domain-containing protein	AT2G12550	Protein synthesis, modificatio	2.05	1.87E-04
RNI-like superfamily protein	AT1G80570	Protein synthesis, modificatio	2.04	5.28E-02
autophagy-related protein 8f	AT4G16520	Protein synthesis, modificatio	2.03	3.58E-03
RNI-like superfamily protein	AT5G07670	Protein synthesis, modificatio	2.02	4.96E-02
DWD motif protein	AT3G45620	Protein synthesis, modificatio	2.01	7.59E-05
RING/U-box superfamily protein	AT1G49850	Protein synthesis, modificatio	2.01	1.25E-02
F-box/kelch-repeat protein SKIP11	AT2G02870	Protein synthesis, modificatio	2.00	1.72E-02
ubiquitin-like protein ATG12A	AT1G54210	Protein synthesis, modificatio	1.99	2.07E-02
chaperone protein dnaJ 20	AT4G13830	Protein synthesis, modificatio	1.99	4.10E-04
uncharacterized protein	AT1G09070	Protein synthesis, modificatio	-2.00	1.00E+00
Matrixin family protein	AT1G59970	Protein synthesis, modificatio	-2.00	1.54E-03
uncharacterized protein	AT1G67700	Protein synthesis, modificatio	-2.02	4.76E-02
Co-chaperone GrpE family protein	AT1G36390	Protein synthesis, modificatio	-2.03	9.98E-03
ubiquitin-specific protease family C19-related protein	AT1G16860	Protein synthesis, modificatio	-2.06	1.80E-04
50S ribosomal protein L13	AT1G78630	Protein synthesis, modificatio	-2.09	3.28E-02
ribosomal L18p/L5e family protein	AT5G27820	Protein synthesis, modificatio	-2.12	1.45E-01
peptidyl-prolyl cis-trans isomerase CYP20-2	AT5G13120	Protein synthesis, modificatio	-2.13	1.20E-03
FKBP-type peptidyl-prolyl cis-trans isomerase 5	AT1G18170	Protein synthesis, modificatio	-2.14	3.13E-04
50S ribosomal protein L1	AT3G63490	Protein synthesis, modificatio	-2.19	5.49E-03
cysteine proteinase-like protein	AT3G02070	Protein synthesis, modificatio	-2.19	2.03E-03
translation elongation factor EF1B/ribosomal protein S6 family protein	AT1G64510	Protein synthesis, modificatio	-2.21	4.79E-03
ARM repeat superfamily protein	AT1G67530	Protein synthesis, modificatio	-2.23	2.39E-03
30S ribosomal protein S10-like	AT3G13120	Protein synthesis, modificatio	-2.23	2.44E-02
serine carboxypeptidase-like 25	AT3G02110	Protein synthesis, modificatio	-2.25	1.00E+00
protein phosphatase 2C 58	AT4G28400	Protein synthesis, modificatio	-2.26	1.86E-02
peptidyl-tRNA hydrolase family protein	AT5G38290	Protein synthesis, modificatio	-2.27	1.32E-02
30S ribosomal protein S5	AT2G33800	Protein synthesis, modificatio	-2.28	1.79E-02
30S ribosomal protein S10	AT3G13120	Protein synthesis, modificatio	-2.28	9.28E-03
plastid-specific 50S ribosomal protein 6	AT5G17870	Protein synthesis, modificatio	-2.29	1.00E+00
50S ribosomal protein L3-1	AT2G43030	Protein synthesis, modificatio	-2.30	4.54E-03
chaperonin-60 alpha	AT2G28000	Protein synthesis, modificatio	-2.33	8.09E-03
ribosomal protein L35	AT2G24090	Protein synthesis, modificatio	-2.35	1.76E-02
50S ribosomal protein L24	AT5G54600	Protein synthesis, modificatio	-2.37	5.16E-02
30S ribosomal protein 3-1	AT1G68590	Protein synthesis, modificatio	-2.40	2.74E-02
ubiquitin-conjugating enzyme E2 20	AT1G50490	Protein synthesis, modificatio	-2.41	0.00E+00
ER lumen protein retaining receptor-like protein	AT1G19970	Protein synthesis, modificatio	-2.44	1.01E-03

50S ribosomal protein L28	AT2G33450	Protein synthesis, modificatio	-2.45	2.22E-02
peptidyl-prolyl cis-trans isomerase FKBP16-3	AT2G43560	Protein synthesis, modificatio	-2.46	7.11E-04
50S ribosomal protein L5	AT4G01310	Protein synthesis, modificatio	-2.47	1.57E-02
FtsH extracellular protease	AT4G23940	Protein synthesis, modificatio	-2.51	7.06E-03
50S ribosomal protein related protein	AT5G16200	Protein synthesis, modificatio	-2.52	3.22E-03
30S ribosomal protein S20	AT3G15190	Protein synthesis, modificatio	-2.54	1.19E-01
aspartyl protease family protein	AT3G54400	Protein synthesis, modificatio	-2.56	8.67E-05
photosystem II stability/assembly factor HCF136	AT5G23120	Protein synthesis, modificatio	-2.57	1.22E-02
protein disulfide-isomerase LQY1	AT1G75690	Protein synthesis, modificatio	-2.64	3.93E-02
peptidyl-prolyl cis-trans isomerase FKBP16-4	AT3G10060	Protein synthesis, modificatio	-2.64	1.14E-01
S2P-like metalloprotease	AT5G05740	Protein synthesis, modificatio	-2.65	8.66E-03
FKBP-type peptidyl-prolyl cis-trans isomerase 3	AT5G45680	Protein synthesis, modificatio	-2.66	8.77E-04
F-box protein	AT1G78100	Protein synthesis, modificatio	-2.68	4.33E-03
50S ribosomal protein L34	AT1G29070	Protein synthesis, modificatio	-2.69	4.66E-03
50S ribosomal protein L21	AT1G35680	Protein synthesis, modificatio	-2.69	3.65E-03
plastid ribosomal protein S21	AT3G27160	Protein synthesis, modificatio	-2.70	2.44E-02
chaperonin 60 subunit beta 1	AT1G55490	Protein synthesis, modificatio	-2.80	7.67E-02
peptidyl-prolyl cis-trans isomerase FKBP17-3	AT1G73655	Protein synthesis, modificatio	-2.83	7.88E-03
RING/U-box domain-containing protein	AT1G45180	Protein synthesis, modificatio	-2.84	3.30E-06
50S ribosomal protein L19-2	AT5G47190	Protein synthesis, modificatio	-2.94	2.19E-03
peptidyl-prolyl cis-trans isomerase CYP20-3	AT3G62030	Protein synthesis, modificatio	-2.95	1.00E+00
Hypersensitive-induced response protein 2	AT1G69840	Protein synthesis, modificatio	-3.01	5.08E-03
aspartyl protease family protein	AT1G66180	Protein synthesis, modificatio	-3.34	7.59E-06
U-box domain-containing protein 31	AT5G65920	Protein synthesis, modificatio	-3.43	1.90E-03
peptidyl-prolyl cis-trans isomerase CYP37	AT3G15520	Protein synthesis, modificatio	-3.47	3.18E-02
ARM repeat superfamily protein	AT1G23030	Protein synthesis, modificatio	-3.52	2.25E-04
palmitoyl protein thioesterase family protein	AT5G47330	Protein synthesis, modificatio	-4.04	3.00E-01
membrane-anchored ubiquitin-fold protein 4	AT3G26980	Protein synthesis, modificatio	-4.17	4.51E-04
phenazine biosynthesis PhzC/PhzF family protein	AT4G02850	Protein synthesis, modificatio	-4.79	9.36E-04
serine protease inhibitor, Kazal-type family protein	AT4G01575	Protein synthesis, modificatio	-4.80	2.33E-04
RING-H2 finger protein ATL17	AT4G15975	Protein synthesis, modificatio	-5.09	7.06E-03
uncharacterized protein	AT3G59310	Protein synthesis, modificatio	-7.78	5.25E-08
chaperone DnaJ-domain containing protein	AT1G72416	Protein synthesis, modificatio	-9.26	7.13E-07
aspartyl protease family protein	AT4G16563	Protein synthesis, modificatio	-42.42	1.31E-03
FLG22-induced receptor-like kinase 1 (FRK1)	AT2G19190	Signaling	58.47	1.09E-05
PAR1 protein	AT5G52390	Signaling	16.76	2.11E-03
inorganic pyrophosphatase 1	AT1G73010	Signaling	13.41	1.00E+00
protein kinase-like protein	AT3G46280	Signaling	11.85	2.06E-05
LRR receptor-like protein kinase	AT1G51890	Signaling	11.68	7.96E-07
concanavalin A-like lectin kinase-like protein	AT5G06740	Signaling	11.30	1.23E-04
glutamate receptor 1.2	AT5G48400	Signaling	10.98	8.99E-06
glutamate receptor 2.5	AT5G11210	Signaling	10.30	1.05E-04
Lectin-domain containing receptor kinase A4.2	AT5G01550	Signaling	9.40	1.79E-03
calcium-transporting ATPase 12	AT3G63380	Signaling	7.65	3.49E-06
G-type lectin S-receptor-like serine/threonine-protein kinase	AT1G61480	Signaling	6.82	2.09E-05
cysteine-rich receptor-like protein kinase 7	AT4G23150	Signaling	6.50	2.35E-04
Receptor-like protein kinase-related family protein	AT3G22060	Signaling	6.14	9.03E-06
LRR receptor-like serine/threonine-protein kinase	AT1G51860	Signaling	6.02	2.15E-06
glutamate receptor 2.9	AT2G29100	Signaling	5.57	8.66E-07
cysteine-rich receptor-like protein kinase 37	AT4G04500	Signaling	5.27	2.83E-06
calcium-binding protein CML43	AT5G44460	Signaling	5.22	1.00E-03
Protein kinase family protein	AT5G38250	Signaling	4.76	2.45E-04
cysteine-rich receptor-like protein kinase 13	AT4G23210	Signaling	4.73	3.71E-06
Leucine-rich repeat transmembrane protein kinase	AT1G56120	Signaling	4.70	1.23E-04
for hypothetical protein, clone: RAFL21-67-K19	AT1G29715	Signaling	4.21	2.07E-04
protein MATERNAL EFFECT EMBRYO ARREST 62	AT5G45800	Signaling	4.06	1.94E-03
receptor-like protein kinase	AT1G72540	Signaling	4.05	6.02E-06
inactive leucine-rich repeat receptor-like protein kinase	AT1G66830	Signaling	4.05	6.26E-04
cysteine-rich receptor-like protein kinase 36	AT4G04490	Signaling	4.04	4.35E-06
Cysteine/Histidine-rich C1 domain family protein	AT2G21850	Signaling	4.02	9.10E-04
wall-associated receptor kinase-like 16	AT3G25490	Signaling	4.01	7.24E-02
concanavalin A-like lectin protein kinase family protein	AT3G08870	Signaling	3.83	2.09E-03
plasmodesmata-located protein 5	AT1G70690	Signaling	3.67	1.00E-04
SEC7-like guanine nucleotide exchange family protein	AT4G35380	Signaling	3.63	1.05E-02
G-type lectin S-receptor-like serine/threonine-protein kinase	AT1G61550	Signaling	3.56	2.32E-04
SEC14 family protein	AT4G35750	Signaling	3.46	2.36E-05
concanavalin A-like lectin kinase-like protein	AT3G45330	Signaling	3.36	2.23E-04
inactive receptor kinase	AT5G53320	Signaling	3.27	3.39E-02
cysteine-rich receptor-like protein kinase 20	AT4G23280	Signaling	3.15	1.23E-06
glutamate receptor 1.4	AT3G07520	Signaling	3.02	9.40E-03

atypical dual-specificity phosphatase 4	AT4G03960	Signaling	3.00	1.18E-04
Wall-associated receptor kinase	AT5G53110	Signaling	2.99	1.32E-03
G-type lectin S-receptor-like serine/threonine-protein kinase	AT1G67520	Signaling	2.92	8.43E-04
SNF1-related kinase	AT2G25090	Signaling	2.92	4.45E-01
leucine-rich repeat protein kinase-like protein	AT5G07150	Signaling	2.79	1.85E-03
protein IQ-DOMAIN 14	AT2G43680	Signaling	2.76	9.69E-02
cysteine-rich receptor-like protein kinase 22	AT4G23300	Signaling	2.73	2.92E-05
protein kinase family protein	AT3G61960	Signaling	2.68	1.97E-04
receptor-like protein kinase ANXUR1	AT3G04690	Signaling	2.59	1.08E-03
CBL-interacting protein kinase 5	AT5G10930	Signaling	2.42	1.92E-02
U-box domain-containing protein kinase family protein	AT5G65500	Signaling	2.38	1.88E-02
leucine-rich repeat (LRR) family protein	AT1G13910	Signaling	2.31	6.28E-03
Cam-binding protein 60-like G	AT5G26920	Signaling	2.30	3.29E-03
Histone H3 K4-specific methyltransferase SET7/9 family protein	AT4G17080	Signaling	2.22	4.41E-02
receptor serine/threonine kinase	AT1G70250	Signaling	2.21	7.84E-03
protein kinase	AT1G48490	Signaling	2.17	2.40E-05
concanavalin A-like lectin kinase-like protein	AT5G59260	Signaling	2.16	4.80E-02
purple acid phosphatase 22	AT3G52820	Signaling	2.14	1.00E+00
WD40 domain-containing protein	AT5G42010	Signaling	2.13	1.23E-05
receptor-like protein kinase 1	AT5G60900	Signaling	2.10	1.00E+00
mitogen-activated protein kinase 1	AT1G10210	Signaling	2.09	8.05E-03
calcineurin B-like protein 6	AT4G16350	Signaling	2.08	6.03E-05
protein kinase family protein	AT5G42440	Signaling	2.08	3.35E-04
protein kinase family protein	AT4G21366	Signaling	2.07	1.16E-04
CBL-interacting serine/threonine-protein kinase 23	AT1G30270	Signaling	2.00	2.21E-05
RabGAP/TBC domain-containing protein	AT5G54780	Signaling	1.99	5.04E-03
leucine-rich receptor-like protein kinase family protein	AT2G25790	Signaling	-1.99	2.12E-04
protein-tyrosine phosphatase	AT3G44620	Signaling	-2.00	4.46E-03
protein kinase family protein	AT2G25220	Signaling	-2.02	6.40E-03
ROP guanine nucleotide exchange factor 5	AT5G05940	Signaling	-2.02	2.93E-02
Rho GTPase activating protein with PAK-box/P21-Rho-binding domain	AT4G03100	Signaling	-2.03	1.43E-01
calcium-dependent protein kinase 4	AT4G09570	Signaling	-2.04	4.96E-02
protein kinase family protein	AT3G57120	Signaling	-2.05	7.06E-03
plasmodesmata-located protein 7	AT5G37660	Signaling	-2.05	1.00E+00
LRR receptor-like serine/threonine-protein kinase	AT3G47570	Signaling	-2.06	1.95E-04
Rac-like GTP-binding protein ARAC5	AT1G75840	Signaling	-2.07	9.19E-04
GTP-binding protein	AT4G02790	Signaling	-2.09	5.66E-03
Leucine-rich repeat protein kinase family protein	AT4G23740	Signaling	-2.13	7.22E-03
proline-rich receptor-like protein kinase PERK15	AT1G52290	Signaling	-2.17	8.98E-04
HAD superfamily, subfamily IIIB acid phosphatase	AT4G29270	Signaling	-2.21	6.60E-01
photolyase/blue-light receptor 2	AT2G47590	Signaling	-2.24	1.00E+00
RabGAP/TBC domain-containing protein	AT4G27100	Signaling	-2.24	3.44E-03
leucine-rich receptor-like protein kinase	AT4G20940	Signaling	-2.27	9.03E-03
protein kinase family protein	AT3G09830	Signaling	-2.29	1.53E-01
protein kinase family protein	AT5G40540	Signaling	-2.29	7.24E-04
cysteine-rich receptor-like protein kinase 42	AT5G40380	Signaling	-2.30	2.43E-07
receptor-like protein kinase	AT2G39360	Signaling	-2.31	1.03E-02
calmodulin binding protein-like protein	AT5G62570	Signaling	-2.37	9.40E-04
calmodulin-binding protein	AT1G27460	Signaling	-2.37	1.00E+00
CBL-interacting protein kinase 2	AT5G07070	Signaling	-2.38	2.60E-02
protein IQ-domain 2	AT5G03040	Signaling	-2.39	1.20E-05
Protein kinase protein with tetratricopeptide repeat domain	AT1G63500	Signaling	-2.41	2.59E-03
protein kinase family protein	AT1G33770	Signaling	-2.42	3.29E-03
mitogen-activated protein kinase 3	AT3G45640	Signaling	-2.45	9.94E-03
calcium-binding endonuclease/exonuclease/phosphatase family protein	AT1G02270	Signaling	-2.50	1.22E-04
mitogen-activated protein kinase 13	AT1G07880	Signaling	-2.53	1.78E-02
leucine-rich receptor-like protein kinase	AT1G72180	Signaling	-2.53	5.66E-03
protein phosphatase 2C 47	AT3G51470	Signaling	-2.54	8.93E-04
serine/threonine-protein kinase aurora-2	AT2G25880	Signaling	-2.55	8.80E-02
protein kinase superfamily protein	AT3G61080	Signaling	-2.56	8.29E-02
protein phosphatase 2C 5	AT1G09160	Signaling	-2.60	1.17E-04
protein TIC 62	AT3G18890	Signaling	-2.62	2.95E-02
serine/threonine-protein kinase	AT1G01540	Signaling	-2.64	2.07E-03
calmodulin 2	AT2G41110	Signaling	-2.66	4.29E-02
protein ralf-like 24	AT3G23805	Signaling	-2.67	1.00E+00
leucine-rich repeat protein 1	AT5G16590	Signaling	-2.68	9.36E-05
GTP1/OBG family protein	AT5G18570	Signaling	-2.72	4.33E-03
copine-like protein BONZAI 1	AT5G61900	Signaling	-2.74	2.46E-04
GTP-binding protein	AT1G56050	Signaling	-2.78	2.75E-04
phosphatidylinositol-4-phosphate 5-kinase 6	AT3G07960	Signaling	-2.80	5.75E-04
calcium dependent protein kinase 1	AT5G04870	Signaling	-2.85	1.93E-05

mechanosensitive channel of small conductance-like 6	AT1G78610	Signaling	-2.90	1.57E-04
leucine-rich repeat protein kinase-like protein	AT3G23750	Signaling	-2.90	9.10E-04
leucine-rich repeat (LRR) family protein	AT1G33610	Signaling	-3.01	2.49E-03
protein kinase-like protein	AT5G61570	Signaling	-3.10	5.90E-03
cysteine-rich receptor-like protein kinase 10	AT4G23180	Signaling	-3.11	1.45E-04
protein IQ-domain 23	AT5G62070	Signaling	-3.12	4.89E-06
LRR receptor-like serine/threonine-protein kinase FLS2	AT5G46330	Signaling	-3.16	1.71E-02
HAD superfamily, subfamily IIIB acid phosphatase	AT1G04040	Signaling	-3.25	4.61E-02
calmodulin-like protein 11	AT3G22930	Signaling	-3.29	8.00E-01
phosphoinositide phospholipase C 7	AT3G55940	Signaling	-3.44	5.36E-04
protein phosphatase 2C 12	AT1G47380	Signaling	-3.53	6.16E-05
serine/threonine kinase	AT2G31010	Signaling	-3.73	1.01E-04
RAB GTPase-like protein 7A	AT2G21880	Signaling	-3.81	1.00E+00
mitogen-activated protein kinase kinase 6	AT5G56580	Signaling	-3.98	5.78E-03
transducin/WD40 domain-containing protein	AT5G53500	Signaling	-4.15	8.21E-04
calcium-dependent lipid-binding domain-containing protein	AT4G34150	Signaling	-4.16	3.84E-04
calcium-binding protein CML40	AT3G01830	Signaling	-4.20	4.53E-02
Remorin family protein	AT5G23750	Signaling	-4.36	7.16E-05
Remorin family protein	AT2G02170	Signaling	-4.40	1.00E-04
protein EXORDIUM like 2	AT5G64260	Signaling	-4.46	1.89E-03
leucine-rich repeat protein kinase family protein	AT1G51805	Signaling	-4.69	7.20E-02
phosphoglucan phosphatase LSF2	AT3G10940	Signaling	-4.79	7.62E-04
serine/threonine kinase	AT1G74330	Signaling	-4.92	4.20E-06
receptor-like protein kinase THESEUS 1	AT5G54380	Signaling	-4.97	1.00E+00
calcium-dependent protein kinase 32	AT3G57530	Signaling	-5.43	1.09E-05
cysteine-rich receptor-like protein kinase 29	AT4G21410	Signaling	-5.44	1.78E-04
calcium-dependent protein kinase 28	AT5G66210	Signaling	-5.64	4.32E-06
PTI1-like tyrosine-protein kinase 3	AT3G59350	Signaling	-5.87	2.30E-06
calcium-binding protein CML24	AT5G37770	Signaling	-5.90	1.55E-02
calcium-binding protein CML42	AT4G20780	Signaling	-5.92	9.42E-02
leucine-rich repeat protein kinase family protein	AT1G51805	Signaling	-6.31	1.97E-03
cysteine-rich receptor-like protein kinase 41	AT4G00970	Signaling	-7.59	2.74E-04
Calmodulin-binding protein	AT4G31000	Signaling	-10.17	2.21E-04
phosphate-responsive 1 family protein	AT4G08950	Signaling	-20.16	1.06E-07
uncharacterized protein	AT2G39530	Transport	27.98	9.67E-07
cation/H(+) antiporter 17	AT4G23700	Transport	25.75	4.90E-07
amino acid transporter 1	AT4G21120	Transport	15.70	3.83E-04
glucose-6-phosphate/phosphate translocator 2	AT1G61800	Transport	8.99	2.58E-02
MATE efflux family protein	AT2G04100	Transport	8.28	2.25E-05
ADP/ATP carrier 3 protein	AT4G28390	Transport	6.61	2.05E-04
copper transport family protein	AT5G52720	Transport	5.87	6.32E-03
inositol transporter 2	AT1G30220	Transport	5.55	2.93E-04
auxin efflux carrier family protein	AT2G17500	Transport	5.47	1.48E-01
exocyst subunit exo70 family protein H1	AT3G55150	Transport	4.44	3.39E-05
urea-proton symporter DUR3	AT5G45380	Transport	4.42	7.03E-06
inorganic phosphate transporter 1-1	AT5G43350	Transport	4.39	1.00E+00
inorganic phosphate transporter 1-4	AT2G38940	Transport	3.89	1.00E+00
vesicle-associated protein 3-1	AT2G23830	Transport	3.80	4.53E-02
MATE efflux family protein	AT2G04070	Transport	3.48	1.30E-03
multidrug resistance-associated protein 8	AT3G13090	Transport	3.46	5.00E-03
cation/H(+) antiporter 16	AT1G64170	Transport	3.39	5.73E-05
peptide/nitrate transporter	AT5G14940	Transport	3.35	1.02E-02
ABC transporter A family member 7	AT3G47780	Transport	3.32	3.61E-02
Sec14p-like phosphatidylinositol transfer family protein	AT1G75170	Transport	3.12	3.09E-02
ABC transporter C family member 7	AT3G13100	Transport	2.95	3.25E-06
MATE efflux family protein	AT1G71140	Transport	2.88	1.11E-01
potassium transporter 6	AT1G70300	Transport	2.88	1.16E-04
aromatic and neutral transporter 1	AT3G11900	Transport	2.84	4.87E-03
copper transport protein	AT4G05030	Transport	2.80	2.31E-03
copper-transporting ATPase HMA5	AT1G63440	Transport	2.75	1.33E-01
ABC transporter G family member 18	AT3G55110	Transport	2.67	2.08E-04
sugar transport protein 13	AT5G26340	Transport	2.57	3.75E-04
plasma-membrane choline transporter family protein	AT3G03700	Transport	2.56	9.87E-04
MATE efflux family protein	AT3G21690	Transport	2.55	2.43E-02
sugar transporter ERD6-like 17	AT5G27350	Transport	2.54	1.00E+00
ABC transporter C family member 4	AT2G47800	Transport	2.51	8.77E-04
TRAF-like family protein	AT5G26280	Transport	2.50	1.03E-02
auxin efflux transmembrane transporter MDR4	AT2G47000	Transport	2.35	8.99E-03
zinc transporter ZIP2 - like protein	AT1G55910	Transport	2.33	0.00E+00
ABC transporter G family member 31	AT2G29940	Transport	2.32	1.00E+00

zinc transporter	AT3G08650	Transport	2.25	4.41E-02
cation/H(+) antiporter 20	AT3G53720	Transport	2.24	3.57E-03
clathrin assembly protein	AT5G35200	Transport	2.16	4.13E-05
phospholipid-transporting ATPase 12	AT1G26130	Transport	2.08	1.76E-04
SNARE-like family protein	AT4G27840	Transport	2.07	6.97E-02
phospholipid-transporting ATPase 1	AT5G04930	Transport	2.05	2.28E-03
H(+)-ATPase 2	AT4G30190	Transport	2.02	3.75E-03
MATE efflux family protein	AT3G26590	Transport	1.99	2.88E-03
cationic amino acid transporter 2	AT1G58030	Transport	1.99	4.91E-03
K efflux antiporter KEA1	AT1G01790	Transport	-2.03	1.07E-02
nucleotide-sugar transporter	AT2G43240	Transport	-2.05	1.13E-03
SNARE associated Golgi family protein	AT1G12450	Transport	-2.07	2.76E-05
transmembrane amino acid transporter family protein	AT5G41800	Transport	-2.09	5.11E-01
cationic amino acid transporter 7	AT3G10600	Transport	-2.10	1.50E-01
aluminum activated malate transporter family protein	AT5G46600	Transport	-2.16	3.19E-03
ABC transporter G family member 27	AT3G52310	Transport	-2.18	2.44E-02
exocyst subunit exo70 family protein B1	AT5G58430	Transport	-2.21	3.81E-03
patellin-5	AT4G09160	Transport	-2.23	2.18E-03
peptide transporter PTR5	AT5G01180	Transport	-2.24	1.00E+00
nucleotide-sugar transporter family protein	AT5G04160	Transport	-2.34	2.40E-04
golgi nucleotide sugar transporter 3	AT1G76340	Transport	-2.38	5.08E-05
cyclic nucleotide-gated channel 15	AT2G28260	Transport	-2.42	2.92E-01
ABC transporter B family member 21	AT3G62150	Transport	-2.52	4.96E-02
peptide/nitrate transporter	AT1G33440	Transport	-2.55	5.04E-05
nucleotide-sugar transporter-like protein	AT1G21070	Transport	-2.62	3.00E-02
Sodium/calcium exchanger family protein	AT5G17850	Transport	-2.74	1.14E-03
Sec14p-like phosphatidylinositol transfer protein patellin-4	AT1G30690	Transport	-2.76	7.24E-04
peptide/nitrate transporter	AT5G13400	Transport	-2.81	2.11E-02
sec14p-like phosphatidylinositol transfer-like protein	AT5G56160	Transport	-2.84	1.49E-04
sugar transporter ERD6-like 4	AT1G19450	Transport	-2.91	3.01E-04
inorganic carbon transport protein-related protein	AT1G70760	Transport	-2.97	5.65E-01
phosphatidylinositol transfer protein COW1	AT4G34580	Transport	-3.13	6.14E-02
phosphoenolpyruvate (pep)/phosphate translocator 2	AT3G01550	Transport	-3.14	1.00E+00
amino acid permease 5	AT1G44100	Transport	-3.20	8.51E-04
sugar transporter ERD6	AT1G08930	Transport	-3.20	2.16E-02
aluminum-activated, malate transporter 12	AT4G17970	Transport	-3.29	1.42E-02
hydrolase-like protein	AT5G17670	Transport	-3.35	1.28E-02
sulfate transporter 2;1	AT5G10180	Transport	-3.36	1.00E+00
phosphate transporter PHO1-8	AT1G35350	Transport	-3.61	3.11E-05
nucleotide-sugar transporter family protein	AT4G18205	Transport	-3.64	1.56E-02
aquaporin TIP1-1	AT2G36830	Transport	-3.65	1.31E-03
MATE efflux family protein	AT1G15150	Transport	-3.70	5.70E-03
phosphate transporter PHO1-7	AT1G26730	Transport	-3.78	1.67E-03
MATE efflux family protein	AT5G17700	Transport	-3.84	7.28E-01
aquaporin TIP1-1	AT2G36830	Transport	-3.98	1.05E-02
autoinhibited Ca2+-ATPase 1	AT1G27770	Transport	-4.09	1.71E-02
exocyst subunit exo70 family protein H7	AT5G59730	Transport	-4.47	9.65E-03
glucosinolate transporter 1	AT3G47960	Transport	-5.33	5.68E-06
cyclic nucleotide-gated channel 14	AT2G24610	Transport	-5.96	6.38E-05
purine permease 18	AT1G57990	Transport	-7.95	1.00E+00
MATE efflux family protein	AT1G61890	Transport	-10.63	5.71E-04
MATE efflux family protein	AT5G52050	Transport	-12.52	3.00E-04
FAD-binding Berberine family protein	AT1G26390	Unknown	104.75	1.87E-04
uncharacterized protein	AT1G13520	Unknown	47.75	2.95E-02
cytochrome P450, family 82, subfamily C, polypeptide 2	AT4G31970	Unknown	45.42	2.17E-04
plant invertase/pectin methylesterase inhibitor domain-containing protein	AT5G46960	Unknown	44.83	2.18E-05
germin-like protein subfamily 1 member 8	AT4G14630	Unknown	42.21	1.22E-03
uncharacterized protein	AT3G55790	Unknown	36.72	1.09E-05
membrane lipoprotein	AT3G18250	Unknown	33.90	1.27E-03
invertase/pectin methylesterase inhibitor family protein	AT5G46950	Unknown	27.29	1.97E-03
uncharacterized protein	AT2G39518	Unknown	25.43	3.45E-04
uncharacterized protein	AT5G37840	Unknown	22.81	6.42E-05
uncharacterized protein	AT3G19615	Unknown	22.74	1.39E-02
FAD-binding Berberine family protein	AT1G30700	Unknown	20.95	7.22E-03
Cupredoxin superfamily protein	AT2G15780	Unknown	20.42	2.87E-03
Adenine nucleotide alpha hydrolases-like superfamily protein	AT5G47740	Unknown	20.31	7.37E-05
cytochrome P450, family 82, subfamily C, polypeptide 3	AT4G31950	Unknown	19.77	3.74E-03
PLAC8 family protein	AT1G68630	Unknown	17.81	8.88E-07
SPFH/Band 7/PHB domain-containing membrane-associated protein	AT5G25260	Unknown	17.15	3.23E-07
beta glucosidase 46	AT1G61820	Unknown	16.76	3.19E-02

uncharacterized protein	AT1G36640	Unknown	15.65	2.73E-06
uncharacterized protein	AT3G15536	Unknown	12.62	1.41E-02
uncharacterized protein	AT1G15385	Unknown	12.60	4.18E-04
uncharacterized protein	AT5G44575	Unknown	12.57	9.25E-06
uncharacterized protein	AT1G53625	Unknown	12.33	4.42E-04
beta glucosidase 27	AT3G60120	Unknown	12.04	1.42E-01
clone 155459 sequence	AT1G55525	Unknown	11.73	2.17E-01
phloem protein 2-A7	AT5G45090	Unknown	11.38	1.00E+00
uncharacterized protein	AT3G13950	Unknown	10.78	2.94E-04
uncharacterized protein	AT4G11655	Unknown	10.57	3.74E-05
plant invertase/pectin methylesterase inhibitor	AT1G62760	Unknown	10.13	1.31E-03
pollen Ole e 1 allergen and extensin family protein	AT4G17215	Unknown	9.90	4.76E-06
O-Glycosyl hydrolases family 17 protein	AT5G64790	Unknown	9.31	1.83E-03
VQ motif-containing protein	AT4G20000	Unknown	9.11	4.74E-05
uncharacterized protein	AT1G36622	Unknown	8.48	2.89E-04
polynucleotidyl transferase, ribonuclease H-like superfamily protein	AT3G12470	Unknown	8.32	1.02E-02
glycosyl hydrolase family protein 17	AT5G63225	Unknown	8.29	6.94E-05
germin-like protein 6	AT5G39100	Unknown	8.23	1.52E-02
uncharacterized protein	AT3G61198	Unknown	7.88	1.23E-03
uncharacterized protein	AT3G26440	Unknown	7.86	1.64E-07
late embryogenesis abundant (LEA) hydroxyproline-rich glycoprotein	AT1G65690	Unknown	7.72	6.42E-06
uncharacterized protein	AT2G45360	Unknown	7.66	5.74E-03
uncharacterized protein	AT1G65481	Unknown	7.59	5.94E-06
uncharacterized protein	AT1G53620	Unknown	7.34	2.00E-06
uncharacterized protein	AT3G26440	Unknown	7.29	5.54E-04
cytochrome P450, family 714, subfamily A, polypeptide 1	AT5G24910	Unknown	7.07	1.20E-02
serine-rich protein-like protein	AT3G56500	Unknown	6.77	8.75E-03
uncharacterized protein	AT2G21185	Unknown	6.58	5.20E-06
uncharacterized protein	AT2G29430	Unknown	6.26	5.71E-02
AAA-type ATPase family protein	AT3G28540	Unknown	6.13	9.91E-04
uncharacterized protein	AT4G28460	Unknown	6.07	2.50E-02
alpha/beta-Hydrolases superfamily protein	AT1G08310	Unknown	5.83	3.66E-02
cytochrome P450 71B6	AT2G24180	Unknown	5.81	8.67E-05
pyruvate kinase-like protein	AT3G49160	Unknown	5.80	6.22E-02
uncharacterized protein	AT3G29240	Unknown	5.66	3.76E-05
for hypothetical protein, clone: RAFL22-67-I17	AT5G01732	Unknown	5.61	9.95E-06
uncharacterized protein	AT5G44585	Unknown	5.57	1.42E-04
heat stress transcription factor B-1	AT4G36990	Unknown	5.52	3.45E-05
uncharacterized protein	AT1G65500	Unknown	5.43	2.30E-03
carbohydrate-binding X8 domain-containing protein	AT2G03505	Unknown	5.32	1.54E-03
pollen Ole e 1 allergen and extensin family protein	AT2G40113	Unknown	5.29	5.04E-03
uncharacterized protein	AT3G57950	Unknown	5.24	2.53E-03
heavy-metal-associated domain-containing protein	AT5G26690	Unknown	5.19	6.09E-04
phosphorylase superfamily protein	AT4G28940	Unknown	5.19	8.93E-04
VQ motif-containing protein	AT4G15120	Unknown	5.12	2.41E-03
uncharacterized protein	AT3G29240	Unknown	5.10	3.94E-02
uncharacterized protein	AT1G10140	Unknown	4.98	6.77E-03
Endosomal targeting BRO1-like domain-containing protein	AT1G13310	Unknown	4.96	9.10E-04
uncharacterized protein	AT4G37900	Unknown	4.92	1.13E-02
uncharacterized protein	AT5G44572	Unknown	4.86	2.98E-06
cryptdin-related protein	AT1G51915	Unknown	4.85	9.06E-04
uncharacterized protein	AT5G03230	Unknown	4.83	3.95E-05
uncharacterized protein	AT3G14280	Unknown	4.82	3.00E-04
uncharacterized protein	AT3G15760	Unknown	4.80	3.67E-04
Ninja-family protein AFP3	AT3G29575	Unknown	4.79	1.00E-02
cytochrome P450 71B2	AT1G13080	Unknown	4.68	8.41E-08
uncharacterized protein	AT4G19970	Unknown	4.65	1.90E-04
uncharacterized protein	AT3G13435	Unknown	4.60	6.81E-03
Wound-responsive family protein	AT4G05070	Unknown	4.59	2.65E-05
uncharacterized protein	AT3G07350	Unknown	4.43	6.34E-01
uncharacterized protein	AT1G13480	Unknown	4.42	5.56E-05
Ninja-family protein AFP3	AT3G29575	Unknown	4.41	1.23E-02
uncharacterized protein	AT1G51913	Unknown	4.37	4.29E-03
cytochrome P450, family 81, subfamily H, polypeptide 1	AT4G37310	Unknown	4.36	7.07E-05
uncharacterized protein	AT1G49470	Unknown	4.35	1.84E-02
pyridoxal phosphate phosphatase-related protein	AT1G17710	Unknown	4.34	2.68E-02
SRPBCC ligand-binding domain-containing protein	AT4G32870	Unknown	4.07	2.20E-03
uncharacterized protein	AT5G22270	Unknown	4.05	2.41E-03
uncharacterized protein	AT4G34630	Unknown	4.02	1.98E-05
uncharacterized protein	AT5G40720	Unknown	3.96	4.25E-02
uncharacterized protein	AT5G02020	Unknown	3.89	2.35E-02

uncharacterized protein	AT1G23560	Unknown	3.88	7.28E-02
D-3-phosphoglycerate dehydrogenase	AT1G17745	Unknown	3.79	5.39E-05
cytochrome P450, family 89, subfamily A, polypeptide 5	AT1G64950	Unknown	3.78	3.20E-03
cytochrome P450 monooxygenase	AT3G26220	Unknown	3.76	1.23E-04
Methyltransferase-related protein	AT5G58375	Unknown	3.68	6.42E-06
uncharacerized protein	AT4G36988	Unknown	3.65	2.73E-03
TRAF-like family protein	AT3G46190	Unknown	3.62	6.99E-05
gunnii alcohol dehydrogenase-like protein	AT1G09480	Unknown	3.61	1.09E-02
uncharacterized protein	AT3G15534	Unknown	3.61	1.19E-02
Mo25 family protein	AT2G03410	Unknown	3.60	6.40E-03
uncharacterized protein	AT4G25070	Unknown	3.56	1.07E-04
core-2/I-branching beta-1,6-N-acetylglucosaminyltransferase	AT1G68390	Unknown	3.45	3.47E-03
calcium-dependent lipid-binding domain-containing protein	AT1G23140	Unknown	3.40	2.36E-04
late embryogenesis abundant hydroxyproline-rich glycoprotein	AT4G35170	Unknown	3.40	1.11E-06
cytochrome P450, family 87, subfamily A, polypeptide 6	AT2G12190	Unknown	3.39	8.75E-03
Exostosin family protein	AT5G25820	Unknown	3.38	3.47E-03
uncharacterized protein	AT3G25240	Unknown	3.37	1.62E-01
UDP-glycosyltransferase 86A1	AT2G36970	Unknown	3.35	5.17E-05
uncharacterized protein	AT1G13990	Unknown	3.33	1.49E-03
copper amine oxidase family protein	AT4G12290	Unknown	3.30	9.92E-05
uncharacterized protein	AT4G30230	Unknown	3.29	1.04E-02
clone 119768 sequence	AT3G12502	Unknown	3.27	4.26E-01
uncharacterized protein	AT2G27830	Unknown	3.26	2.39E-02
lysophospholipase 1-like protein	AT2G39410	Unknown	3.24	1.05E-03
formin-like protein 16	AT5G07770	Unknown	3.23	1.55E-03
uncharacterized protein	AT4G19370	Unknown	3.18	2.48E-03
cytochrome P450 89A2	AT1G64900	Unknown	3.13	1.03E-04
TRAM, LAG1 and CLN8 (TLC) lipid-sensing domain containing protein	AT3G27270	Unknown	3.13	3.10E-03
Flavin-binding monooxygenase family protein	AT1G12160	Unknown	3.10	2.60E-02
uncharacterized protein	AT1G13530	Unknown	3.10	7.70E-04
uncharacterized protein	AT1G10410	Unknown	3.09	1.53E-04
uncharacterized protein	AT3G13432	Unknown	3.09	1.60E-02
uncharacterized protein	AT4G39610	Unknown	3.07	4.66E-03
uncharacterized protein	AT1G64405	Unknown	3.06	7.02E-04
O-acyltransferase (WSD1-like) family protein	AT5G12420	Unknown	3.05	2.85E-03
uncharacterized protein	AT3G52480	Unknown	3.04	4.21E-04
uncharacterized protein	AT3G04700	Unknown	3.03	2.58E-04
uncharacterized protein	AT5G60630	Unknown	3.02	5.40E-02
RNI-like superfamily protein	AT5G45500	Unknown	3.00	3.54E-04
uncharacterized protein	AT3G21710	Unknown	2.99	1.17E-02
cytochrome P450, family 705, subfamily A, polypeptide 33	AT3G20960	Unknown	2.97	1.65E-02
DnaJ domain-containing protein	AT3G08970	Unknown	2.97	1.84E-04
uncharacterized protein	AT2G22320	Unknown	2.95	9.65E-02
uncharacterized protein	AT1G68650	Unknown	2.94	4.08E-03
uncharacterized protein	AT1G21050	Unknown	2.92	8.11E-05
polyadenylate-binding protein-interacting protein 2	AT4G14270	Unknown	2.90	1.19E-05
uncharacterized protein	AT1G71970	Unknown	2.89	2.78E-03
protein DJ-1-like A	AT3G14990	Unknown	2.89	1.01E-02
cytochrome P450 71B26	AT3G26290	Unknown	2.86	1.53E-03
cytochrome P450, family 76, subfamily C, polypeptide 5	AT1G33730	Unknown	2.85	1.93E-03
uncharacterized protein	AT1G13360	Unknown	2.83	1.83E-04
uncharacterized protein	AT5G44578	Unknown	2.81	8.33E-02
TRAM, LAG1 and CLN8 (TLC) lipid-sensing domain containing protein	AT4G19645	Unknown	2.79	3.70E-02
uncharacterized protein	AT1G70160	Unknown	2.78	9.45E-05
uncharacterized protein	AT1G71910	Unknown	2.77	6.85E-03
Exostosin family protein	AT5G11610	Unknown	2.77	2.19E-03
uncharacterized protein	AT3G19660	Unknown	2.69	5.39E-05
leucine-rich repeat-containing protein	AT1G15740	Unknown	2.69	1.31E-03
HXXXD-type acyl-transferase-like protein	AT4G15400	Unknown	2.68	3.17E-02
uncharacterized protein	AT4G33666	Unknown	2.64	3.23E-01
alpha/beta-Hydrolases superfamily protein	AT3G51000	Unknown	2.60	6.17E-04
calcium-dependent lipid-binding domain-containing protein	AT3G55470	Unknown	2.60	1.11E-03
uncharacterized protein	AT2G31160	Unknown	2.60	5.00E-03
uncharacterized protein	AT5G65207	Unknown	2.59	6.30E-02
uncharacterized protein	AT1G13550	Unknown	2.58	1.07E-04
uncharacterized protein	AT3G03870	Unknown	2.57	2.05E-03
for hypothetical protein, clone: RAFL21-14-O08	AT3G26165	Unknown	2.57	1.59E-03
uncharacterized protein	AT3G13432	Unknown	2.56	1.46E-03
cytochrome P450 71B24	AT3G26230	Unknown	2.55	9.82E-04
cytochrome P450 71A22	AT3G48310	Unknown	2.53	1.05E-03
uncharacterized protein	AT1G52855	Unknown	2.53	2.05E-03

late embryogenesis abundant hydroxyproline-rich glycoprotein	AT2G46150	Unknown	2.52	1.86E-04
uncharacterized protein	AT5G59400	Unknown	2.52	3.06E-02
Agenet domain-containing protein	AT5G52070	Unknown	2.51	1.72E-03
cytochrome P450 71B20	AT3G26180	Unknown	2.49	3.77E-02
ARM repeat superfamily protein	AT3G03440	Unknown	2.49	1.95E-03
HIPL2 protein	AT5G62630	Unknown	2.48	7.96E-04
cytochrome P450, family 81, subfamily D, polypeptide 5	AT4G37320	Unknown	2.48	3.52E-05
uncharacterized protein	AT5G01610	Unknown	2.47	2.49E-04
uncharacterized protein	AT4G23885	Unknown	2.47	1.20E-06
uncharacterized protein	AT5G47580	Unknown	2.44	1.47E-04
uncharacterized protein	AT2G40390	Unknown	2.44	6.94E-03
uncharacterized protein	AT2G28570	Unknown	2.43	4.00E-03
cytochrome P450, family 704, subfamily A, polypeptide 2	AT2G45510	Unknown	2.43	1.28E-04
uncharacterized protein	AT1G10690	Unknown	2.42	7.35E-03
reticulon-like protein B9	AT3G18260	Unknown	2.42	2.61E-02
clone asmbl_2021 unknown sequence	AT1G32172	Unknown	2.42	3.23E-01
uncharacterized protein	AT3G27880	Unknown	2.42	9.03E-03
Myosin heavy chain-related protein	AT4G40020	Unknown	2.39	5.62E-04
calcium-dependent lipid-binding domain-containing protein	AT2G01540	Unknown	2.39	9.81E-03
uncharacterized protein	AT1G65845	Unknown	2.39	2.62E-05
uncharacterized protein	AT4G23880	Unknown	2.38	6.70E-01
SART-1 family protein	AT3G14700	Unknown	2.38	1.93E-03
uncharacterized protein	AT4G09830	Unknown	2.34	5.16E-03
Per1-like family protein	AT5G62130	Unknown	2.33	4.08E-01
uncharacterized protein	AT2G15695	Unknown	2.33	6.43E-04
uncharacterized protein	AT3G10250	Unknown	2.30	1.27E-04
ribosomal protein L18ae family protein	AT4G26060	Unknown	2.29	1.40E-04
Rossmann-fold NAD(P)-binding domain-containing protein	AT4G09750	Unknown	2.29	2.23E-02
uncharacterized protein	AT3G24927	Unknown	2.27	3.57E-03
clone 7553 sequence	AT1G67328	Unknown	2.26	2.91E-03
uncharacterized protein	AT1G15790	Unknown	2.25	1.18E-03
uncharacterized protein	AT4G11860	Unknown	2.24	7.10E-03
uncharacterized protein	AT5G62900	Unknown	2.24	2.18E-02
uncharacterized protein	AT4G14620	Unknown	2.24	1.80E-02
uncharacterized protein	AT5G57910	Unknown	2.20	3.86E-02
uncharacterized protein	AT5G10946	Unknown	2.17	7.92E-04
ARM repeat superfamily protein	AT3G51980	Unknown	2.17	2.46E-03
cytochrome P450 71B11	AT5G25120	Unknown	2.17	1.64E-02
uncharacterized protein	AT3G54880	Unknown	2.16	1.53E-02
uncharacterized protein	AT4G11350	Unknown	2.16	1.34E-02
Erythronate-4-phosphate dehydrogenase family protein	AT1G19400	Unknown	2.15	1.38E-02
uncharacterized protein	AT1G01730	Unknown	2.15	2.51E-04
uncharacterized protein	AT2G41660	Unknown	2.14	5.83E-02
camphor resistance CrcB-like protein	AT2G41705	Unknown	2.12	3.58E-04
uncharacterized protein	AT5G04790	Unknown	2.12	7.24E-02
cyclic phosphodiesterase	AT4G18930	Unknown	2.11	2.96E-03
Carbohydrate-binding protein	AT1G10150	Unknown	2.11	2.21E-02
uncharacterized protein	AT1G08180	Unknown	2.09	1.90E-04
uncharacterized protein	AT1G33050	Unknown	2.09	2.18E-02
uncharacterized protein	AT5G22550	Unknown	2.09	2.71E-01
uncharacterized protein	AT1G29240	Unknown	2.08	3.62E-03
Mediator complex, subunit Med10	AT1G26665	Unknown	2.07	5.57E-03
uncharacterized protein	AT1G65720	Unknown	2.06	1.52E-02
uncharacterized protein	AT5G19860	Unknown	2.06	7.72E-03
uncharacterized protein	AT5G49525	Unknown	2.06	7.08E-02
ACT domain-containing protein	AT2G39570	Unknown	2.05	5.45E-03
uncharacterized protein	AT4G06676	Unknown	2.04	2.10E-02
Yippee family zinc-binding protein	AT2G40110	Unknown	2.03	8.21E-03
cytochrome P450, family 705, subfamily A, polypeptide 28	AT3G20935	Unknown	2.03	5.16E-02
uncharacterized protein	AT1G28070	Unknown	2.03	1.29E-01
uncharacterized protein	AT2G10560	Unknown	2.02	8.37E-05
protein narrow leaf 1-like protein	AT3G12950	Unknown	2.02	6.32E-03
uncharacterized protein	AT5G44574	Unknown	2.01	6.60E-04
aldehyde dehydrogenase 2C4	AT3G24503	Unknown	2.01	2.62E-04
electron transfer flavoprotein subunit alpha	AT1G50940	Unknown	2.01	3.85E-03
chaperone protein dnaJ 20	AT4G13830	Unknown	2.01	9.99E-04
Erythronate-4-phosphate dehydrogenase family protein	AT1G75180	Unknown	2.01	1.37E-02
uncharacterized protein	AT1G65985	Unknown	2.00	2.15E-03
protein PHLOEM protein 2-LIKE A9	AT1G31200	Unknown	2.00	1.00E+00
protein Fes1C	AT5G02150	Unknown	1.99	3.13E-03
B-cell receptor-associated protein 31-like protein	AT3G07190	Unknown	-1.99	3.87E-02

uncharacterized protein	AT3G15480	Unknown	-2.00	2.00E-05
uncharacterized protein	AT2G21960	Unknown	-2.01	1.07E-02
uncharacterized protein	AT5G41100	Unknown	-2.01	4.58E-03
uncharacterized protein	AT2G25250	Unknown	-2.01	1.81E-02
uncharacterized protein	AT1G73020	Unknown	-2.02	1.57E-03
Vps4 regulator of MVB pathway	AT2G19710	Unknown	-2.02	1.85E-02
receptor like protein 51	AT4G18760	Unknown	-2.02	8.68E-01
pentatricopeptide repeat-containing protein	AT5G01110	Unknown	-2.02	1.67E-03
uncharacterized protein	AT3G61840	Unknown	-2.03	1.00E+00
uncharacterized protein	AT1G59865	Unknown	-2.03	1.46E-01
mesophyll-cell RNAi library line 7-like protein	AT2G31840	Unknown	-2.03	1.36E-02
Unknown	AT3G53010	Unknown	-2.04	2.00E-04
uncharacterized protein	AT2G34670	Unknown	-2.04	1.54E-02
uncharacterized protein	AT5G50610	Unknown	-2.05	1.37E-02
cotton-Golgi related 3	AT5G65810	Unknown	-2.05	3.92E-03
uncharacterized protein	AT3G50340	Unknown	-2.05	1.65E-03
uncharacterized protein	AT5G27560	Unknown	-2.05	1.47E-02
uncharacterized protein	AT1G64700	Unknown	-2.08	1.78E-02
armadillo/beta-catenin-like repeat-containing protein	AT5G50900	Unknown	-2.09	6.90E-03
tetratricopeptide repeat domain-containing protein	AT3G47080	Unknown	-2.11	5.52E-05
uncharacterized protein	AT4G15790	Unknown	-2.15	1.74E-02
alpha/beta-hydrolase-like protein	AT1G29840	Unknown	-2.18	1.76E-05
double Clp-N motif-containing P-loop nucleoside triphosphate	AT4G30350	Unknown	-2.19	1.61E-02
alpha/beta-Hydrolases superfamily protein	AT5G09430	Unknown	-2.20	2.07E-03
chromosome transmission fidelity protein 8	AT5G52220	Unknown	-2.21	1.18E-02
uncharacterized protein	AT5G64850	Unknown	-2.21	1.06E-05
uncharacterized protein	AT3G23170	Unknown	-2.22	7.94E-02
NDH dependent flow 6 protein	AT1G18730	Unknown	-2.24	1.00E+00
uncharacterized protein	AT5G42110	Unknown	-2.24	2.21E-03
uncharacterized protein	AT3G59880	Unknown	-2.25	1.00E+00
methyltransferase	AT5G01710	Unknown	-2.25	5.28E-04
uncharacterized protein	AT3G28760	Unknown	-2.26	1.29E-02
uncharacterized protein	AT4G01150	Unknown	-2.26	2.00E-01
uncharacterized protein	AT1G03730	Unknown	-2.27	1.11E-04
uncharacterized protein	AT5G66675	Unknown	-2.28	1.05E-04
uncharacterized protein	AT3G29280	Unknown	-2.28	3.68E-02
cold regulated 413 plasma membrane 1	AT2G15970	Unknown	-2.28	1.60E-02
uncharacterized protein	AT1G68430	Unknown	-2.30	1.41E-02
fringe-related protein	AT4G00300	Unknown	-2.30	2.71E-02
uncharacterized protein	AT5G11070	Unknown	-2.31	9.87E-04
tetratricopeptide repeat-containing protein	AT3G05625	Unknown	-2.32	5.86E-03
uncharacterized protein	AT1G01570	Unknown	-2.33	4.47E-04
myb domain protein 73	AT4G37260	Unknown	-2.33	2.96E-03
protein LUTEIN DEFICIENT 5	AT1G31800	Unknown	-2.33	8.91E-03
uncharacterized protein	AT4G30996	Unknown	-2.34	1.09E-05
uncharacterized protein	AT3G06150	Unknown	-2.34	7.31E-03
uncharacterized protein	AT2G43340	Unknown	-2.36	2.38E-02
uncharacterized protein	AT5G22390	Unknown	-2.37	7.64E-03
thylakoid lumenal protein 17.9	AT4G24930	Unknown	-2.38	1.49E-02
BSD domain-containing protein	AT1G69030	Unknown	-2.38	4.92E-02
uncharacterized protein	AT5G61412	Unknown	-2.39	1.02E-03
uncharacterized protein	AT4G09640	Unknown	-2.39	1.80E-05
major facilitator protein	AT2G16990	Unknown	-2.40	2.25E-03
uncharacterized protein	AT4G22830	Unknown	-2.40	4.55E-04
uncharacterized protein	AT3G53010	Unknown	-2.41	1.35E-05
uncharacterized protein	AT3G13275	Unknown	-2.41	2.10E-04
cytochrome P450 86A2	AT4G00360	Unknown	-2.42	7.06E-03
uncharacterized protein	AT5G48790	Unknown	-2.43	3.43E-01
tetratricopeptide repeat-containing protein	AT1G78915	Unknown	-2.44	1.00E-01
GTP-binding protein Era	AT5G66470	Unknown	-2.44	7.55E-03
uncharacterized protein	AT5G43880	Unknown	-2.44	3.84E-04
Rubber elongation factor protein	AT2G47780	Unknown	-2.45	2.31E-03
transmembrane protein 97	AT1G05210	Unknown	-2.45	3.79E-03
uncharacterized protein	AT5G03670	Unknown	-2.45	2.49E-02
uncharacterized protein	AT4G24175	Unknown	-2.50	1.33E-04
uncharacterized protein	AT1G68330	Unknown	-2.51	1.07E-01
beta glucosidase 40	AT1G26560	Unknown	-2.51	2.76E-02
uncharacterized protein	AT1G10020	Unknown	-2.51	3.25E-04
calcium-dependent lipid-binding domain-containing protein	AT3G19830	Unknown	-2.52	1.00E+00
uncharacterized protein	AT4G17000	Unknown	-2.54	3.25E-01
uncharacterized protein	AT4G21570	Unknown	-2.54	9.98E-03

Rossmann-fold NAD(P)-binding domain-containing protein	AT4G20760	Unknown	-2.55	4.45E-02
uncharacterized protein	AT1G10522	Unknown	-2.56	9.07E-03
alpha/beta-Hydrolases superfamily protein	AT1G13820	Unknown	-2.57	2.93E-04
uncharacterized protein	AT5G48470	Unknown	-2.57	5.82E-03
uncharacterized protein	AT5G43950	Unknown	-2.58	1.06E-02
uncharacterized protein	AT2G38465	Unknown	-2.58	9.34E-02
uncharacterized protein	AT5G62960	Unknown	-2.58	1.88E-04
uncharacterized protein	AT2G42110	Unknown	-2.59	2.88E-02
uncharacterized protein	AT3G07380	Unknown	-2.60	3.08E-05
uncharacterized protein	AT1G69890	Unknown	-2.62	6.26E-04
uncharacterized protein	AT1G27030	Unknown	-2.62	2.77E-05
uncharacterized protein	AT3G02640	Unknown	-2.64	4.12E-01
uncharacterized protein	AT5G37360	Unknown	-2.66	7.46E-05
pollen Ole e 1 allergen and extensin family protein	AT2G16630	Unknown	-2.67	1.00E+00
uncharacterized protein	AT2G36410	Unknown	-2.67	8.07E-05
transducin/WD40 repeat-like superfamily protein	AT1G78070	Unknown	-2.68	1.12E-04
dihydrodipicolinate reductase 1	AT2G44040	Unknown	-2.69	1.89E-04
methyltransferase	AT3G28460	Unknown	-2.70	1.80E-03
LORELEI-LIKE-GPI-ANCHORED PROTEIN 1	AT5G56170	Unknown	-2.71	4.79E-03
Unknown	AT5G01542	Unknown	-2.72	4.39E-05
hydrolase, alpha/beta fold family protein	AT4G12830	Unknown	-2.72	4.17E-02
uncharacterized protein	AT2G21120	Unknown	-2.74	7.86E-02
polyketide cyclase / dehydrase and lipid transport protein	AT4G01883	Unknown	-2.74	3.55E-03
beta carbonic anhydrase 4	AT1G70410	Unknown	-2.77	3.64E-04
uncharacterized protein	AT5G11000	Unknown	-2.77	1.67E-01
uncharacterized protein	AT2G28140	Unknown	-2.77	2.03E-02
uncharacterized protein	AT2G33400	Unknown	-2.77	4.88E-02
D-mannose binding lectin protein	AT1G78820	Unknown	-2.78	3.99E-04
uncharacterized protein	AT3G55646	Unknown	-2.78	1.00E+00
uncharacterized protein	AT4G24265	Unknown	-2.79	7.40E-02
uncharacterized protein	AT1G69760	Unknown	-2.79	2.10E-05
uncharacterized protein	AT4G27350	Unknown	-2.81	3.16E-03
uncharacterized protein	AT2G20724	Unknown	-2.81	1.03E-03
uncharacterized protein	AT3G07460	Unknown	-2.81	6.94E-05
uncharacterized protein	AT5G44005	Unknown	-2.82	3.11E-02
plastidal glycolate/glycerate translocator 1	AT1G32080	Unknown	-2.82	1.18E-01
uncharacterized protein	AT4G29400	Unknown	-2.82	2.58E-02
uncharacterized protein	AT3G01960	Unknown	-2.83	3.14E-03
magnesium chelatase subunit I2	AT5G45930	Unknown	-2.84	4.38E-04
uncharacterized protein	AT4G38545	Unknown	-2.85	1.40E-01
uncharacterized protein	AT5G48310	Unknown	-2.85	5.59E-02
uncharacterized protein	AT4G18540	Unknown	-2.85	4.96E-01
heptahelical transmembrane protein1	AT5G20270	Unknown	-2.87	4.53E-02
uncharacterized protein	AT3G60990	Unknown	-2.87	2.96E-05
CASP-like protein	AT2G35760	Unknown	-2.88	1.50E-02
cadmium tolerance 1	AT1G52827	Unknown	-2.89	1.05E-01
uncharacterized protein	AT1G75860	Unknown	-2.92	3.42E-04
thylakoid lumenal protein 2	AT5G52970	Unknown	-2.95	6.26E-04
uncharacterized protein	AT1G53633	Unknown	-2.95	9.73E-02
cytokine-induced anti-apoptosis inhibitor 1, Fe-S biogenesis	AT5G18400	Unknown	-2.96	4.65E-02
uncharacterized protein	AT4G20170	Unknown	-2.96	2.45E-04
hydrolase, alpha/beta fold family protein	AT5G02970	Unknown	-2.97	4.42E-04
uncharacterized protein	AT3G13437	Unknown	-2.99	2.35E-01
conserved peptide upstream open reading frame 32	AT1G73602	Unknown	-3.01	2.52E-02
Stress responsive alpha-beta barrel domain protein	AT2G32500	Unknown	-3.03	1.79E-02
uncharacterized protein	AT3G14190	Unknown	-3.04	2.68E-01
uncharacterized protein	AT1G05540	Unknown	-3.06	3.68E-03
HXXXD-type acyl-transferase-like protein	AT3G23840	Unknown	-3.07	5.32E-02
uncharacterized protein	AT5G10750	Unknown	-3.09	3.36E-04
formin-like protein 7	AT1G59910	Unknown	-3.09	4.76E-06
uncharacterized protein	AT1G69430	Unknown	-3.11	1.95E-04
uncharacterized protein	AT5G44010	Unknown	-3.16	1.66E-04
PLAC8 family protein	AT1G11380	Unknown	-3.17	1.75E-04
O-Glycosyl hydrolases family 17 protein	AT5G55180	Unknown	-3.17	3.53E-03
uncharacterized protein	AT3G46110	Unknown	-3.19	1.20E-01
uncharacterized protein	AT1G29980	Unknown	-3.20	6.20E-03
uncharacterized protein	AT3G46880	Unknown	-3.21	1.81E-01
for hypothetical protein, clone: RAFL21-49-A06	AT5G36002	Unknown	-3.28	9.19E-04
early nodulin-like protein 15	AT4G31840	Unknown	-3.30	6.63E-01
CASP-like protein	AT2G38480	Unknown	-3.33	3.03E-03
uncharacterized protein	AT4G18070	Unknown	-3.35	5.39E-05

O-Glycosyl hydrolases family 17 protein	AT3G07320	Unknown	-3.38	1.08E-02
uncharacterized protein	AT2G24330	Unknown	-3.40	4.82E-05
uncharacterized protein	AT1G01130	Unknown	-3.44	4.47E-04
uncharacterized protein	AT1G20070	Unknown	-3.44	4.70E-01
uncharacterized protein	AT5G03390	Unknown	-3.45	1.38E-03
cysteine/histidine-rich C1 domain-containing protein	AT4G02540	Unknown	-3.45	8.45E-05
pollen Ole e 1 allergen and extensin family protein	AT3G26960	Unknown	-3.46	2.04E-03
FAD-binding and BBE domain-containing protein	AT4G20820	Unknown	-3.47	5.13E-05
uncharacterized protein	AT3G07470	Unknown	-3.49	3.74E-05
uncharacterized protein	AT3G63160	Unknown	-3.49	1.21E-01
hydrolase	AT1G52510	Unknown	-3.57	2.20E-03
uncharacterized protein	AT5G57770	Unknown	-3.59	7.33E-05
uncharacterized protein	AT4G17240	Unknown	-3.60	2.09E-02
uncharacterized protein	AT5G27290	Unknown	-3.63	2.38E-02
C1 domain-containing protein	AT5G03360	Unknown	-3.64	6.00E-04
for hypothetical protein, clone: RAFL09-28-L17	AT1G79245	Unknown	-3.72	1.46E-03
O-fucosyltransferase family protein	AT2G44500	Unknown	-3.72	1.13E-03
Galactose-binding protein	AT1G22882	Unknown	-3.76	1.74E-03
Rossmann-fold NAD(P)-binding domain-containing protein	AT2G37540	Unknown	-3.76	5.75E-04
Exostosin family protein	AT4G32790	Unknown	-3.85	1.84E-06
early nodulin-like protein 13	AT5G25090	Unknown	-3.87	7.24E-02
uncharacterized protein	AT3G49720	Unknown	-3.97	5.81E-06
uncharacterized protein	AT3G11420	Unknown	-4.00	2.74E-06
uncharacterized protein	AT2G35658	Unknown	-4.05	3.61E-02
uncharacterized protein	AT3G61920	Unknown	-4.09	1.14E-04
cysteine/histidine-rich C1 domain-containing protein	AT2G23100	Unknown	-4.09	1.44E-06
cytochrome P450, family 96, subfamily A, polypeptide 4	AT5G52320	Unknown	-4.15	4.09E-02
major facilitator protein	AT4G27720	Unknown	-4.17	9.34E-06
uncharacterized protein	AT1G18060	Unknown	-4.18	5.24E-03
alpha carbonic anhydrase 1	AT3G52720	Unknown	-4.18	5.90E-05
uncharacterized protein	AT2G12400	Unknown	-4.36	1.34E-03
Unknown	AT1G74330	Unknown	-4.44	4.72E-04
uncharacterized protein	AT1G61667	Unknown	-4.48	1.05E-03
uncharacterized protein	AT4G25830	Unknown	-4.50	2.91E-06
uncharacterized protein	AT1G79245	Unknown	-4.53	2.69E-04
uncharacterized protein	AT3G11420	Unknown	-4.56	2.16E-06
uncharacterized protein	AT2G27402	Unknown	-4.57	4.78E-05
uncharacterized protein	AT4G29780	Unknown	-4.76	7.37E-05
regulator of Vps4 activity protein	AT4G32350	Unknown	-4.95	2.52E-02
ATP sulfurylase	AT4G14680	Unknown	-5.08	2.55E-02
Actin cross-linking protein	AT1G69900	Unknown	-5.11	2.36E-04
uncharacterized protein	AT4G39840	Unknown	-5.25	1.83E-03
Regulator of Vps4 activity in the MVB pathway protein	AT4G35730	Unknown	-5.31	5.36E-04
NADPH--cytochrome P450 reductase 2	AT4G30210	Unknown	-5.36	5.73E-05
Heavy metal transport/detoxification superfamily protein	AT5G05365	Unknown	-5.37	6.20E-06
uncharacterized protein	AT1G06475	Unknown	-5.47	4.42E-07
gb|AYBHY07TR pooled cDNA populations Arabidopsis thaliana cDNA	AT1G79245	Unknown	-5.50	4.34E-04
uncharacterized protein	AT3G06070	Unknown	-5.58	4.78E-05
Hs1pro-1 protein	AT3G55840	Unknown	-5.68	3.95E-03
VQ motif-containing protein	AT2G22880	Unknown	-5.85	4.62E-03
uncharacterized protein	AT2G34510	Unknown	-5.88	4.45E-06
uncharacterized protein	AT2G44230	Unknown	-5.94	9.66E-04
beta-glucosidase 47	AT4G21760	Unknown	-6.01	1.19E-03
uncharacterized protein	AT2G33850	Unknown	-6.07	6.01E-01
uncharacterized protein	AT5G25240	Unknown	-6.17	1.35E-02
UDP-glycosyltransferase-like protein	AT3G46650	Unknown	-6.27	5.59E-02
hydroxyproline-rich glycoprotein family protein	AT1G72790	Unknown	-6.36	3.81E-05
uncharacterized protein	AT5G36920	Unknown	-6.59	1.31E-02
uncharacterized protein	AT1G56660	Unknown	-6.64	5.04E-03
uncharacterized protein	AT1G16850	Unknown	-7.19	5.36E-04
uncharacterized protein	AT3G14870	Unknown	-7.25	9.12E-06
O-methyltransferase family protein	AT1G21110	Unknown	-7.30	1.00E+00
gb|016G06 Infected Arabidopsis Leaf Arabidopsis thaliana cDNA	AT1G79245	Unknown	-7.31	1.32E-03
uncharacterized protein	AT2G32200	Unknown	-7.31	1.62E-02
late embryogenesis abundant hydroxyproline-rich glycoprotein	AT2G27080	Unknown	-7.51	5.77E-05
uncharacterized protein	AT1G19380	Unknown	-7.60	8.38E-04
uncharacterized protein	AT2G36145	Unknown	-7.65	4.89E-04
uncharacterized protein	AT3G19680	Unknown	-7.91	3.87E-09
uncharacterized protein	AT4G04745	Unknown	-8.31	1.38E-01
O-fucosyltransferase family protein	AT3G05320	Unknown	-8.33	2.06E-05
uncharacterized protein	AT2G20835	Unknown	-8.64	4.87E-03

Late embryogenesis abundant protein, group 6	AT2G23120	Unknown	-9.16	3.45E-08
copper amine oxidase	AT1G31690	Unknown	-9.23	2.51E-03
uncharacterized protein	AT5G03120	Unknown	-9.59	2.23E-04
uncharacterized protein	AT2G23690	Unknown	-9.92	2.22E-05
hydrolase, alpha/beta fold family protein	AT3G24420	Unknown	-10.98	2.03E-01
uncharacterized protein	AT5G28610	Unknown	-11.61	7.19E-04
uncharacterized protein	AT4G37240	Unknown	-13.24	4.59E-06
uncharacterized protein	AT5G57760	Unknown	-15.84	2.46E-06
uncharacterized protein	AT1G50040	Unknown	-18.03	6.74E-07
uncharacterized protein	AT2G35290	Unknown	-22.34	3.81E-06

Supplementary Table S4. Differentially expressed genes in *MIM396* plants after challenge with *P. cucumerina* .

MIM_Pcc vs. MIM_Mock				
Description	Description		MIM_Pcc vs. MIM_Mock	
flavin-dependent monooxygenase 1 (FMO1)	AT1G19250	Biotic stress	178.82	3.5E-08
lipid-transfer protein/seed storage	AT4G12490	Biotic stress	85.79	0.00398
lipid transfer protein (LTP)	AT3G22600	Biotic stress	75.44	2.7E-05
cytochrome P450 71A12	AT2G30750	Biotic stress	73.58	5.1E-05
GDSL lipase 1	AT5G40990	Biotic stress	59.93	2.5E-07
AGD2-like defense response protein 1	AT2G13810	Biotic stress	50.46	2.7E-07
calcium-binding protein CML47	AT3G47480	Biotic stress	41.57	3.6E-05
disease resistance protein RMG1	AT4G11170	Biotic stress	31.45	1.2E-07
cysteine/histidine-rich C1 domain-containing protein	AT2G17740	Biotic stress	30.75	0.00027
cytochrome P450, family 71, subfamily A, polypeptide 13	AT2G30770	Biotic stress	30.17	6.6E-05
lipid-transfer protein/seed storage	AT4G12500	Biotic stress	29.87	0.00387
chitinase class 4-like protein	AT2G43570	Biotic stress	27.95	0.00085
PR-6 proteinase inhibitor family protein	AT5G43570	Biotic stress	27.73	0.00124
protein AIG1 (AVRRPT2-Induced Gene 1)	AT1G33960	Biotic stress	23.93	1.9E-06
FAD-binding and BBE domain-containing protein	AT1G26420	Biotic stress	19.94	9.8E-06
chitinase A	AT5G24090	Biotic stress	19.00	9.6E-06
beta-1,3-endoglucanase	AT4G16260	Biotic stress	15.07	0.01549
chitinase	AT2G43620	Biotic stress	14.55	0.00078
uncharacterized protein	AT5G24200	Biotic stress	12.59	0.0001
Chitinase family protein	AT2G43590	Biotic stress	12.04	0.00072
Isochorismate synthase 1	AT1G74710	Biotic stress	9.41	5.8E-06
cytochrome P450 71B23	AT3G26210	Biotic stress	9.20	4.6E-07
PR-6 proteinase inhibitor family protein	AT2G38870	Biotic stress	9.11	1.5E-05
disease resistance protein	AT5G66890	Biotic stress	7.30	0.00011
C2 calcium/lipid-binding plant phosphoribosyltransferase	AT4G00700	Biotic stress	7.29	0.00088
beta-1,3-glucanase 3	AT3G57240	Biotic stress	7.22	0.03853
LURP1 protein	AT2G14560	Biotic stress	6.73	0.0006
defensin-like protein 308	AT5G46871	Biotic stress	6.53	0.00098
downy mildew resistance 6 protein / oxidoreductase	AT5G24530	Biotic stress	6.19	0.0001
UDP-dependent glycosyltransferase 76B1	AT3G11340	Biotic stress	5.99	2E-06
uncharacterized protein	AT5G64510	Biotic stress	5.87	1E-05
Tautomerase/MIF superfamily protein	AT3G51660	Biotic stress	5.71	0.0052
receptor like protein 21	AT2G25470	Biotic stress	5.54	0.03635
Disease resistance protein (TIR-NBS class)	AT1G17615	Biotic stress	5.53	3.4E-05
TIR-NBS-LRR class disease resistance protein	AT5G45000	Biotic stress	5.49	8.2E-06
RPW8 domain-containing powdery mildew resistance protein	AT3G26470	Biotic stress	5.46	0.00041
defensin-like protein 309	AT5G46874	Biotic stress	5.41	0.00083
TIR-NBS class of disease resistance protein	AT1G72890	Biotic stress	5.40	8.8E-06
carboxyesterase 17	AT5G16080	Biotic stress	5.13	0.00017
basic chitinase B	AT3G12500	Biotic stress	5.04	0.01067
NB-ARC domain-containing disease resistance protein	AT3G14470	Biotic stress	4.94	0.00129
MLO-like protein 3	AT3G45290	Biotic stress	4.93	5E-06
LRR and NB-ARC domain-containing disease resistance protein	AT3G14460	Biotic stress	4.46	2E-05
COBRA-like protein 5	AT5G60950	Biotic stress	4.35	0.00657
pathogenesis-related protein 1 domain-containing protein	AT4G07820	Biotic stress	4.34	0.0087
uncharacterized protein	AT4G29110	Biotic stress	4.26	5.1E-05
phospholipase-like protein (PEARLI 4) family	AT2G16900	Biotic stress	4.05	1.5E-05
FAD/NAD(P)-binding oxidoreductase family protein	AT4G38540	Biotic stress	3.67	1.6E-06
leucine-rich repeat-containing protein	AT5G45510	Biotic stress	3.53	7.9E-05
avirulence induced protein	AT1G33950	Biotic stress	3.43	0.00021
4-coumarate--CoA ligase 1	AT1G51680	Biotic stress	3.37	0.00084
uncharacterized protein	AT5G45410	Biotic stress	3.36	0.00027
lipase class 3 family protein / disease resistance protein-related protein (EDS1B)	AT3G48080	Biotic stress	3.34	0.00047
beta glucosidase 10	AT4G27830	Biotic stress	3.31	0.00528
Bax inhibitor-1 family protein	AT5G47130	Biotic stress	3.27	0.00096
CC-NBS-LRR class disease resistance protein	AT1G12290	Biotic stress	3.26	3.4E-05
calreticulin-3	AT1G08450	Biotic stress	3.23	2.5E-05
chitinase	AT1G02360	Biotic stress	3.19	0.01524
alpha dioxygenase	AT1G73680	Biotic stress	3.18	0.00586
Chitinase family protein	AT4G01700	Biotic stress	3.13	0.00072
MLO-like protein	AT2G39200	Biotic stress	2.94	0.00458

VQ motif-containing protein	AT2G41180	Biotic stress	2.91	0.00168
prolyl 4-hydroxylase 5	AT2G17720	Biotic stress	2.89	1.9E-06
cytochrome P450 98A3	AT2G40890	Biotic stress	2.71	0.01589
cysteine-rich receptor-like protein kinase 18	AT4G23260	Biotic stress	2.66	1E-05
flavin monooxygenase.	AT1G12200	Biotic stress	2.64	0.03276
receptor like protein 2	AT1G17240	Biotic stress	2.61	0.00167
alcohol dehydrogenase-like 6	AT5G24760	Biotic stress	2.57	0.00168
methyl esterase 1	AT2G23620	Biotic stress	2.56	0.00245
receptor like protein 43	AT3G28890	Biotic stress	2.49	0.0404
BAX inhibitor 1	AT5G47120	Biotic stress	2.46	0.02624
TIR-NBS-LRR class disease resistance protein	AT5G51630	Biotic stress	2.45	0.00015
LRR receptor-like serine/threonine-protein kinase	AT1G74360	Biotic stress	2.30	0.03849
disease resistance protein	AT4G14610	Biotic stress	2.16	0.00261
glycine-rich family protein	AT4G27850	Biotic stress	2.02	0.00263
glycine-rich protein	AT5G61660	Biotic stress	-2.00	0.02074
late embryogenesis abundant hydroxyproline-rich glycoprotein	AT3G44220	Biotic stress	-2.06	0.02949
elicitor peptide 4	AT5G09980	Biotic stress	-2.12	0.03662
TIR-NBS-LRR class disease resistance protein	AT5G41750	Biotic stress	-2.12	0.02616
pathogenesis-related thaumatin family protein	AT4G36000	Biotic stress	-2.23	0.02941
TIR-NBS-LRR class disease resistance protein	AT1G63860	Biotic stress	-2.25	2.6E-05
TIR-NBS class of disease resistance protein	AT1G66090	Biotic stress	-2.25	0.02074
TIR-NBS-LRR class disease resistance protein	AT5G41740	Biotic stress	-2.27	1.5E-05
defensin-like protein 302	AT3G13403	Biotic stress	-2.29	0.0002
carboxyesterase 5	AT1G49660	Biotic stress	-2.32	0.00295
TIR-NBS-LRR class disease resistance protein	AT4G19520	Biotic stress	-2.35	0.00016
alkenal/one oxidoreductase	AT1G23740	Biotic stress	-2.35	0.04804
TIR-NBS-LRR class disease resistance protein	AT5G58120	Biotic stress	-2.36	0.00013
glucan endo-1,3-beta-glucosidase 10	AT5G42100	Biotic stress	-2.36	0.01268
TIR-NBS-LRR class disease resistance protein	AT1G56540	Biotic stress	-2.44	0.00698
hydroxyproline-rich glycoprotein family protein	AT4G25620	Biotic stress	-2.48	0.013
TIR-NBS-LRR class disease resistance protein	AT1G63750	Biotic stress	-2.48	0.00429
TIR-NBS-LRR class disease resistance protein	AT3G44630	Biotic stress	-2.55	0.00185
lipid-transfer protein/seed storage	AT2G27130	Biotic stress	-2.60	0.00185
Non-specific lipid-transfer protein-like protein	AT2G13820	Biotic stress	-2.62	0.00813
lipid-transfer protein/seed storage	AT3G43720	Biotic stress	-2.85	0.00241
Non-specific lipid-transfer protein-like protein	AT5G64080	Biotic stress	-2.89	0.02816
uncharacterized protein	AT1G61260	Biotic stress	-2.95	2.4E-05
TIR-NBS-LRR class disease resistance protein	AT4G19530	Biotic stress	-2.99	0.02074
PLAT-plant-stress domain-containing protein	AT2G22170	Biotic stress	-3.25	0.00202
pathogenesis-related protein	AT4G25780	Biotic stress	-3.44	0.04961
phytochelatin synthase 1	AT5G44070	Biotic stress	-3.55	0.00059
glycine-rich protein	AT3G04640	Biotic stress	-3.65	0.01618
uncharacterized protein	AT1G58420	Biotic stress	-3.78	0.02239
RPM1-interacting protein 4 (RIN4) family protein	AT4G35655	Biotic stress	-3.79	0.01807
MLO-like protein 4	AT1G11000	Biotic stress	-3.83	8.7E-05
ferrochelatase 1	AT5G26030	Biotic stress	-4.27	0.00088
TIR-NBS-LRR class disease resistance protein	AT5G46510	Biotic stress	-4.43	0.00115
MLO-like protein 8	AT2G17480	Biotic stress	-4.64	1.5E-06
uncharacterized protein	AT4G24275	Biotic stress	-4.91	0.0018
TIR class disease resistance protein	AT1G61100	Biotic stress	-5.09	0.00016
glycine-rich protein	AT1G07135	Biotic stress	-5.28	0.0003
hydroxyproline-rich glycoprotein family protein	AT1G70985	Biotic stress	-6.02	0.00706
Toll-Interleukin-Resistance domain-containing protein	AT1G57850	Biotic stress	-6.15	0.00246
lipid-transfer protein/seed storage	AT1G73550	Biotic stress	-6.56	0.005
uncharacterized protein	AT3G57450	Biotic stress	-6.83	5.6E-05
TIR-NBS-LRR class disease resistance protein	AT5G22690	Biotic stress	-7.33	3.4E-06
Pto-interacting 1-4	AT2G47060	Biotic stress	-7.46	1.8E-08
glycine-rich cell wall protein-like protein	AT4G18280	Biotic stress	-12.04	4.9E-07
CBS domain-containing protein	AT5G53750	Biotic stress	-12.54	0.00135
dicarboxylate carrier 2	AT4G24570	Biotic stress	-13.73	0.00037
pathogenesis-related thaumatin family protein	AT4G36010	Biotic stress	-15.87	3.5E-06
glycine-rich protein	AT5G28630	Biotic stress	-18.15	2.9E-07
RPM1-interacting protein 4-like protein	AT2G17660	Biotic stress	-21.94	4.7E-06
cytochrome P450, family 94, subfamily B, polypeptide 1	AT5G63450	Biotic stress	-28.26	0.00415
disease resistance-like protein/LRR domain-containing protein	AT2G34930	Biotic stress	-31.34	1.3E-05
cytochrome P450, family 94, subfamily C, polypeptide 1	AT2G27690	Biotic stress	-34.89	1.1E-05
Thioredoxin superfamily protein	AT5G38900	Oxidative stress	32.83	2.9E-07
glutathione S-transferase TAU 10	AT1G74590	Oxidative stress	32.75	9.3E-05
apoplastic peroxidase Prx37	AT4G08770	Oxidative stress	32.41	0.02134
glutathione S-transferase tau 3	AT2G29470	Oxidative stress	27.15	0.01313
peroxidase 52	AT5G05340	Oxidative stress	24.36	6E-05

peroxidase 5	AT1G14550	Oxidative stress	23.52	0.00223
oxidoreductase, 2OG-Fe(II) oxygenase family protein	AT4G10500	Oxidative stress	17.39	0.04922
peroxidase	AT5G06730	Oxidative stress	14.84	0.00012
glutathione transferase lambda 1	AT5G02780	Oxidative stress	12.28	3.2E-05
peroxidase 50	AT4G37520	Oxidative stress	11.29	0.00066
Glutathione S-transferase family protein	AT5G44990	Oxidative stress	9.24	1.5E-06
glutathione S-transferase TAU 11	AT1G69930	Oxidative stress	8.50	0.00792
Regulator of Vps4 activity in the MVB pathway protein	AT1G13340	Oxidative stress	8.39	6.6E-05
peroxidase 71	AT5G64120	Oxidative stress	8.18	0.00016
peroxidase	AT5G19880	Oxidative stress	7.90	0.00074
glutathione S-transferase 6	AT1G02930	Oxidative stress	7.26	0.00067
glutaredoxin ATGRXS13	AT1G03850	Oxidative stress	5.97	5.7E-05
glutathione S-transferase F2	AT4G02520	Oxidative stress	5.63	0.00017
PLAC8 family protein	AT1G52200	Oxidative stress	5.41	9.3E-05
glutathione S-transferase TAU 12	AT1G69920	Oxidative stress	5.08	0.0425
peroxidase 4	AT1G14540	Oxidative stress	5.02	0.00433
serine-type endopeptidase inhibitor	AT1G72060	Oxidative stress	3.13	0.01516
glutaredoxin-like protein	AT1G64500	Oxidative stress	3.06	0.00624
glutathione S-transferase zeta-class 1	AT2G02390	Oxidative stress	2.85	0.0034
glucose-6-phosphate dehydrogenase 4	AT1G09420	Oxidative stress	2.81	0.00113
membrane-associated progesterone binding protein 3	AT3G48890	Oxidative stress	2.75	0.00159
glutathione S-transferase	AT1G65820	Oxidative stress	2.72	0.00136
catalase 3	AT1G20620	Oxidative stress	2.71	2E-05
aconitase 2	AT4G26970	Oxidative stress	2.59	0.00281
Aldolase-type TIM barrel family protein	AT5G13420	Oxidative stress	2.52	0.0001
aconitate hydratase 3	AT2G05710	Oxidative stress	2.51	0.00037
glutaredoxin-C1	AT5G63030	Oxidative stress	2.39	0.00043
Homeobox even-skipped homolog protein 1 (EVX-1). - Gallus gallus, partial (9%)	AT4G08390	Oxidative stress	2.35	0.01231
glutaredoxin-C3	AT1G77370	Oxidative stress	2.34	0.0009
protein reduce transmission through pollen	AT1G60420	Oxidative stress	2.31	0.00018
L-ascorbate oxidase	AT4G39830	Oxidative stress	2.14	0.01419
L-ascorbate peroxidase 1	AT1G07890	Oxidative stress	2.14	0.00883
Pyridoxamine 5'-phosphate oxidase family protein	AT2G04690	Oxidative stress	2.14	0.00025
atypical CYS HIS rich thioredoxin 4	AT1G08570	Oxidative stress	2.11	0.00875
Thioredoxin H-type 2 - Arabidopsis thaliana (Mouse-ear cress), partial (74%)	AT5G39950	Oxidative stress	2.07	0.00019
6-phosphogluconate dehydrogenase, decarboxylating 3	AT3G02360	Oxidative stress	2.05	0.04044
uncharacterized protein	AT3G14430	Oxidative stress	2.04	0.00924
NADPH--cytochrome P450 reductase 1	AT4G24520	Oxidative stress	2.00	0.00039
SOUL heme-binding protein	AT5G20140	Oxidative stress	-2.00	0.01524
thioredoxin-like protein	AT1G21350	Oxidative stress	-2.01	0.00353
Fe superoxide dismutase 2	AT5G51100	Oxidative stress	-2.28	0.02865
glutathione transferase lambda 2	AT3G55040	Oxidative stress	-2.31	0.00016
glutaredoxin-C5	AT4G28730	Oxidative stress	-2.49	0.00387
Thioredoxin superfamily protein	AT2G37240	Oxidative stress	-2.55	0.00079
2-Cys peroxiredoxin BAS1	AT3G11630	Oxidative stress	-2.60	0.01394
laccase 8	AT5G01040	Oxidative stress	-2.62	0.00022
thioredoxin-like 2-2	AT4G29670	Oxidative stress	-2.62	0.00516
tRNA dihydrouridine synthase	AT3G63510	Oxidative stress	-2.74	0.00228
glutaredoxin-C12	AT2G47870	Oxidative stress	-3.12	0.00028
monothiol glutaredoxin-S2	AT5G18600	Oxidative stress	-3.14	0.00037
glyoxylate reductase 2	AT1G17650	Oxidative stress	-3.34	0.0161
monothiol glutaredoxin-S11	AT1G06830	Oxidative stress	-3.63	0.0045
monothiol glutaredoxin-S9	AT2G30540	Oxidative stress	-3.97	0.02624
monothiol glutaredoxin-S7	AT4G15670	Oxidative stress	-4.40	0.03491
glutathione S-transferase F11	AT3G03190	Oxidative stress	-4.41	0.01864
monothiol glutaredoxin-S8	AT4G15660	Oxidative stress	-4.79	0.00508
monothiol glutaredoxin-S4	AT4G15680	Oxidative stress	-5.92	6.6E-05
glutaredoxin-like protein	AT2G41330	Oxidative stress	-7.70	2.3E-07
NAD(P)-binding Rossmann-fold-containing protein	AT1G32220	Oxidative stress	-3.56	0.00089
ornithine N-delta-acetyltransferase	AT2G39030	Hormone	14.80	0.00698
Oxoglutarate/iron-dependent oxygenase	AT5G59530	Hormone	10.57	4.8E-06
ethylene-responsive transcription factor ERF096	AT5G43410	Hormone	8.87	5.6E-05
alpha carbonic anhydrase 2	AT2G28210	Hormone	8.15	0.0069
aldo-keto reductase 5	AT1G60730	Hormone	5.47	4.7E-05
Mediator of ABA-regulated dormancy MARD1	AT3G63210	Hormone	4.71	0.00969
2-oxoglutarate (2OG) and Fe(II)-dependent oxygenase-like protein	AT2G30840	Hormone	4.65	0.00068
indole-3-acetic acid-amido synthetase GH3.3	AT2G23170	Hormone	4.20	0.01251
adenine nucleotide alpha hydrolases-like protein	AT1G09740	Hormone	4.13	0.00136
SAUR-like auxin-responsive protein	AT3G60690	Hormone	3.74	2.6E-05
multiprotein-bridging factor 1c	AT3G24500	Hormone	3.64	0.00135
nitrile specifier protein 5	AT5G48180	Hormone	3.38	0.00998

ethylene-responsive transcription factor ERF094	AT1G06160	Hormone	3.37	0.01569
gibberellin receptor GID1L2	AT3G63010	Hormone	3.24	0.00246
SAUR-like auxin-responsive protein	AT4G38860	Hormone	2.89	0.00013
brassinosteroid-responsive RING-H2	AT3G61460	Hormone	2.69	3.5E-05
IAA-amino acid hydrolase ILR1-like 3	AT5G54140	Hormone	2.68	0.00092
auxin-induced in root cultures protein 12	AT3G07390	Hormone	2.57	0.00042
jacalin-like lectin domain-containing protein	AT1G52100	Hormone	2.54	0.00553
BES1/BZR1 1	AT3G50750	Hormone	2.49	0.00858
ABRE binding factor 4	AT3G19290	Hormone	2.48	0.00012
SAUR-like auxin-responsive protein	AT1G16510	Hormone	2.44	0.03328
AT-hook protein of GA feedback 2	AT3G55560	Hormone	2.43	0.02279
abscisic acid responsive element-binding factor 1	AT1G49720	Hormone	2.27	0.00524
receptor-like protein kinase BRI1-like 3	AT3G13380	Hormone	2.26	0.01198
12-oxophytodienoate reductase-like protein 1	AT1G09400	Hormone	2.20	0.00231
auxin signaling F-box 3 protein	AT1G12820	Hormone	2.05	0.01848
gibberellin receptor GID1L3	AT5G27320	Hormone	2.04	0.00595
O-fucosyltransferase-like protein	AT3G02250	Hormone	-2.08	0.00433
ferredoxin C2	AT1G32550	Hormone	-2.19	0.00399
SAUR-like auxin-responsive family protein	AT4G34750	Hormone	-2.22	0.00948
Ent-kaur-16-ene synthase	AT1G79460	Hormone	-2.27	0.02624
arabinogalactan protein 15	AT5G11740	Hormone	-2.46	0.04369
gibberellin 2-beta-dioxygenase 2	AT1G30040	Hormone	-2.56	9E-05
12-oxophytodienoate reductase 1	AT1G76680	Hormone	-2.58	0.00214
transcription factor TCP21	AT5G08330	Hormone	-2.59	0.00079
uncharacterized protein	AT5G40460	Hormone	-2.68	0.0021
S-adenosyl-L-methionine-dependent methyltransferase	AT3G21950	Hormone	-2.70	7E-05
epithiospecifier protein	AT1G54040	Hormone	-2.72	5.8E-05
auxin-responsive protein	AT4G12980	Hormone	-2.75	0.0268
abscisic acid 8'-hydroxylase 1	AT4G19230	Hormone	-2.78	0.00702
12-oxophytodienoate reductase-like protein 2B	AT1G18020	Hormone	-2.79	0.00052
basic helix-loop-helix domain-containing protein	AT2G31730	Hormone	-3.05	3.2E-05
delta(14)-sterol reductase	AT3G52940	Hormone	-3.10	8.6E-05
heptahelical transmembrane protein2	AT4G30850	Hormone	-3.15	0.00089
SAUR-like auxin-responsive protein 9	AT4G34760	Hormone	-3.16	0.00285
gibberellin 2-oxidase 6	AT1G02400	Hormone	-3.47	0.01455
allene oxide synthase	AT5G42650	Hormone	-3.62	0.02412
cytochrome P450 90B1 (steroid 22-alpha-hydrolase CYP90B1)	AT3G50660	Hormone	-3.86	4.2E-06
O-fucosyltransferase family protein	AT5G01100	Hormone	-4.45	0.00179
cold and ABA inducible protein kin1	AT5G15960	Hormone	-5.39	0.01026
ethylene-responsive transcription factor 11	AT1G28370	Hormone	-5.59	0.02964
SAUR-like auxin-responsive protein family	AT1G72430	Hormone	-5.73	7.7E-06
Col-0 2-oxoglutarate-dependent dioxygenase (AOP2) pseudogene	AT4G03060	Hormone	-6.67	0.03431
SAUR-like auxin-responsive protein	AT4G12410	Hormone	-7.60	0.01662
lipoxygenase 4	AT1G72520	Hormone	-7.95	0.00973
ethylene responsive element binding factor 6	AT4G17490	Hormone	-8.30	0.01268
1-aminocyclopropane-1-carboxylate synthase 11	AT4G08040	Hormone	-9.27	6.6E-05
O-fucosyltransferase family protein	AT5G65470	Hormone	-12.97	1.7E-08
ethylene-responsive transcription factor ERF098	AT3G23230	Hormone	-14.94	0.00994
SAUR-like auxin-responsive protein	AT3G09870	Hormone	-18.55	0.00056
S-adenosyl-L-methionine-dependent methyltransferase	AT1G15125	Hormones	5.81	0.02748
ethylene-responsive transcription factor ERF060	AT4G39780	Hormones	2.94	0.03726
ethylene-responsive transcription factor ERF070	AT1G71130	Hormones	2.19	0.00264
auxin response factor 16	AT4G30080	Hormones	2.06	0.0112
auxin-responsive protein IAA7	AT3G23050	Hormones	-2.17	0.0236
AP2-like ethylene-responsive transcription factor AIL5	AT5G57390	hormones	-2.28	0.00107
S-adenosyl-L-methionine-dependent methyltransferase-like protein	AT4G24805	Hormones	-2.36	3E-05
methyltransferase S-adenosyl-L-methionine-dependent methyltransferase	AT4G29590	Hormones	-2.38	0.00234
cytokinin riboside 5'-monophosphate phosphoribohydrolase LOG1	AT2G28305	Hormones	-2.78	0.0298
ethylene-responsive transcription factor RAP2-10	AT4G36900	Hormones	-2.94	0.00025
ethylene-responsive transcription factor ERF018	AT1G74930	Hormones	-3.35	9.9E-05
S-adenosylmethionine-dependent methyltransferase domain-containing protein	AT1G69523	Hormones	-3.49	0.00335
ethylene-responsive transcription factor CRF2	AT4G23750	Hormones	-3.53	0.0059
auxin-responsive protein IAA17	AT1G04250	Hormones	-3.70	0.0064
brassinosteroid metabolic pathway protein BEN1	AT2G45400	hormones	-3.79	0.00079
ethylene-responsive transcription factor ERF058	AT1G22190	Hormones	-4.07	1.4E-05
auxin-responsive protein IAA5	AT1G15580	Hormones	-4.81	0.01018
ethylene-responsive transcription factor ABR1	AT5G64750	Hormones	-11.08	0.0304
ethylene-responsive transcription factor ERF013	AT1G77640	Hormones	-12.92	0.00387
ethylene-responsive transcription factor ERF043	AT4G32800	Hormones	-13.07	6.4E-07
indole-3-acetic acid 6	AT1G52830	Hormones	-17.27	7.2E-06
ethylene-responsive transcription factor ERF022	AT1G33760	Hormones	-48.29	1E-06

FAD-binding and BBE domain-containing protein	AT1G26410	Abiotic stress	36.41	1.2E-05
Late embryogenesis abundant-related protein	AT1G54890	Abiotic stress	25.30	0.01723
germin-like protein subfamily 1 member 19	AT5G39180	Abiotic Stress	24.09	0.00731
mediator of RNA polymerase II transcription subunit 37b	AT1G09080	Abiotic Stress	8.70	0.01116
heat shock protein 90.1	AT5G52640	Abiotic Stress	6.36	0.00944
polyketide cyclase/dehydrase and lipid transport superfamily protein	AT1G23120	Abiotic Stress	5.57	0.00012
UDP-glycosyltransferase family protein	AT4G19460	Abiotic stress	4.42	0.00817
bifunctional nuclease in basal defense response 1	AT1G75380	Abiotic Stress	3.95	0.00028
phosphopantothenoylcysteine decarboxylase	AT1G48605	Abiotic Stress	3.83	0.0001
chaperone protein dnaJ 8	AT1G80920	Abiotic Stress	3.13	0.02508
germin-like protein 5	AT1G09560	Abiotic Stress	2.99	0.03459
C2 domain-containing protein	AT5G55530	Abiotic Stress	2.12	0.00912
CP12 domain-containing protein 3	AT1G76560	Abiotic stress	2.04	0.00142
wound-responsive protein	AT4G28240	Abiotic Stress	2.03	0.03925
protein LOW PHOSPHATE ROOT2	AT1G71040	Abiotic Stress	2.01	0.00969
uncharacterized protein	AT4G33985	Abiotic stress	-2.12	0.00088
dehydrin ERD14	AT1G76180	Abiotic Stress	-2.13	0.01161
ABI five binding protein 2	AT1G13740	Abiotic stress	-2.23	0.00311
dehydration-induced protein ERD15	AT2G41430	Abiotic Stress	-2.25	0.00068
methyltransferase PMT3	AT4G14360	Abiotic Stress	-2.27	0.00121
germin-like protein subfamily 3 member 1	AT1G72610	Abiotic Stress	-2.27	0.00368
Hydrophobic protein RCI2A	AT3G05880	Abiotic Stress	-2.68	0.04527
chaperone DnaJ-domain containing protein	AT5G21430	Abiotic Stress	-2.73	0.04566
Hydrophobic protein RCI2B	AT3G05890	Abiotic Stress	-2.87	0.00061
protein dehydration-INDUCED 19-5	AT4G02200	Abiotic Stress	-2.96	0.00936
Heat shock protein 81-3	AT2G35880	Abiotic stress	-2.97	0.00495
methyltransferase PMT14	AT4G18030	Abiotic Stress	-3.08	0.00062
calmodulin binding protein 25	AT2G41010	Abiotic Stress	-3.10	0.01235
heat shock protein class V 15.4	AT4G21870	Abiotic Stress	-3.30	0.02098
methyltransferase PMT8	AT1G04430	Abiotic Stress	-4.04	8.1E-05
tetratricopeptide repeat-containing protein	AT2G47440	Abiotic Stress	-4.96	0.01565
dehydrin COR47	AT1G20440	Abiotic Stress	-5.56	2.4E-05
low-temperature-responsive protein 78/desiccation-responsive protein 29A	AT5G52310	Abiotic Stress	-5.59	0.00351
uncharacterized protein	AT1G51090	Abiotic stress	-6.29	0.00013
ERD4 protein	AT1G11960	Abiotic Stress	-7.68	6.2E-07
dehydrin ERD10	AT1G20450	Abiotic stress	-8.95	1.4E-05
cold-regulated protein 15b	AT2G42530	Abiotic Stress	-12.04	0.00508
abscisic acid 8'-hydroxylase 3	AT5G45340	Abiotic stress	-19.37	7E-07
cold-regulated protein 15a	AT2G42540	Abiotic Stress	-51.28	2.5E-05
peptin methylesterase 17 (PME17)	AT2G45220	Cell Wall	162.26	0.00415
extensin 3	AT1G21310	Cell Wall	29.98	9.6E-05
extensin 4	AT1G76930	Cell Wall	27.64	0.00817
caffeoyl-CoA 3-O-methyltransferase	AT1G67980	Cell wall	24.75	3.1E-06
FAD-binding and BBE domain-containing protein	AT5G44380	Cell wall	17.40	0.00149
peptin methylesterase 20 (PME20)	AT2G47550	Cell Wall	15.50	0.01654
mannose-6-phosphate isomerase	AT1G67070	Cell Wall	15.00	5.2E-05
UDP-arabinose 4-epimerase 4	AT5G44480	Cell Wall	9.79	8.2E-05
peptidoglycan-binding LysM domain-containing protein	AT5G62150	Cell Wall	8.06	0.00015
polygalacturonase /pectinase	AT2G43870	Cell Wall	7.54	0.02291
hydroxyproline-rich glycoprotein family protein	AT5G51680	Cell Wall	7.18	0.03499
fucosyltransferase 6	AT1G14080	Cell Wall	6.65	0.0005
Proline-rich extensin-like family protein	AT2G43150	Cell Wall	6.55	0.01362
caffeic acid/5-hydroxyferulic acid O-methyltransferase	AT5G54160	Cell wall	5.49	0.00022
cinnamyl alcohol dehydrogenase 5	AT4G34230	Cell wall	5.07	0.02014
expansin A1	AT1G69530	Cell Wall	4.97	8.2E-05
trans-cinnamate 4-monooxygenase	AT2G30490	Cell wall	4.51	0.00578
UDP-arabinose 4-epimerase 3	AT4G20460	Cell Wall	3.81	0.00111
glucuronidase 2	AT5G07830	Cell wall	3.71	0.00023
cinnamyl-alcohol dehydrogenase	AT1G72680	Cell wall	3.29	0.01779
ferulic acid 5-hydroxylase 1	AT4G36220	Cell wall	3.15	0.00019
Proline-rich extensin-like family protein	AT2G24980	Cell wall	2.96	0.00694
pectin lyase-like superfamily protein	AT1G02460	Cell Wall	2.81	0.00512
hydroxycinnamoyl-CoA shikimate/quinate hydroxycinnamoyl transferase	AT5G48930	Cell wall	2.63	0.00545
cinnamoyl coa reductase 1	AT1G15950	Cell wall	2.36	0.00103
Proline-rich extensin-like family protein	AT1G23720	Cell wall	2.19	0.0252
caffeoyl-CoA O-methyltransferase	AT4G34050	Cell wall	2.11	0.04725
lectin-like protein	AT5G03350	Cell wall	2.02	0.02058
arabinose 5-phosphate isomerase	AT3G54690	Cell Wall	-2.03	0.0002
pectin lyase-like superfamily protein	AT4G33440	Cell Wall	-2.05	0.00747
pectinacetylesterase family protein	AT3G62060	Cell Wall	-2.07	0.01394
UDP-glucose 6-dehydrogenase 2	AT5G39320	Cell Wall	-2.10	0.01888

pectate lyase 1	AT1G04680	Cell Wall	-2.11	0.01447
pectinacetylesterase family protein	AT3G05910	Cell Wall	-2.12	0.04804
uncharacterized protein	AT3G08600	Cell wall	-2.15	0.00065
LysM domain-containing GPI-anchored protein 2	AT1G77630	Cell wall	-2.21	0.00183
uncharacterized protein	AT2G25800	Cell wall	-2.24	0.00371
uncharacterized protein	AT5G01590	Cell wall	-2.24	0.00838
glycosyl hydrolase 9B7	AT1G75680	Cell wall	-2.25	0.01699
UDP-D-glucuronate 4-epimerase 6	AT3G23820	Cell Wall	-2.36	0.02748
expansin A5	AT3G29030	Cell Wall	-2.43	0.00013
FASCICLIN-like arabinogalactan protein 8	AT2G45470	Cell Wall	-2.43	0.00017
pectinesterase	AT3G43270	Cell Wall	-2.43	0.03004
GDSL esterase/lipase	AT1G28600	Cell wall	-2.55	0.00065
UDP-glucose 6-dehydrogenase 1	AT3G29360	Cell Wall	-2.57	0.00557
polygalacturonase	AT4G23500	Cell Wall	-2.62	0.03853
arabinogalactan protein 12	AT3G13520	Cell Wall	-2.63	0.02472
cinnamoyl-CoA reductase like protein	AT4G30470	Cell wall	-2.64	0.01715
GDSL esterase/lipase	AT1G29670	Cell wall	-2.66	0.00056
mannan synthase 7	AT2G35650	Cell Wall	-2.70	3.8E-05
extensin-like protein	AT1G12090	Cell wall	-2.72	0.00037
mannose-6-phosphate isomerase	AT3G02570	Cell Wall	-2.73	0.01083
O-Glycosyl hydrolases family 17 protein	AT3G55430	Cell wall	-2.77	0.04605
beta-D-xylosidase 5	AT3G19620	Cell Wall	-2.85	0.00245
GDSL esterase/lipase	AT3G16370	Cell wall	-2.93	0.01585
peptidoglycan-binding LysM domain-containing protein	AT5G23130	Cell wall	-3.00	1.7E-06
leucine-rich repeat extensin-like protein 5	AT4G18670	Cell Wall	-3.05	0.00433
fasciclin-like arabinogalactan protein 7	AT2G04780	Cell Wall	-3.07	0.01666
pectin methylesterase 34 (PME34)	AT3G49220	Cell Wall	-3.12	8.6E-05
mannan synthase 3	AT1G23480	Cell Wall	-3.14	6.9E-06
polygalacturonase-like protein	AT3G06770	Cell Wall	-3.44	8.5E-05
cellulose synthase A5 (CESA5)	AT5G09870	Cell Wall	-3.73	0.01463
fasciclin-like arabinogalactan protein 2	AT4G12730	Cell Wall	-4.09	0.00405
UDP-D-glucuronate 4-epimerase 1	AT4G30440	Cell Wall	-4.09	2.6E-05
pectinacetylesterase family protein	AT5G45280	Cell Wall	-4.29	0.00231
Cellulose-synthase-like C12	AT4G07960	Cell Wall	-4.45	0.00131
GDSL esterase/lipase	AT5G14450	Cell wall	-4.49	0.00399
fasciclin-like arabinogalactan protein 9	AT1G03870	Cell Wall	-4.63	0.00739
FASCICLIN-like arabinogalactan protein 18 precursor	AT3G11700	Cell Wall	-4.67	7.4E-05
protein EXORDIUM like 3	AT5G51550	Cell wall	-5.11	0.00353
early nodulin-like protein 17	AT5G15350	Cell wall	-5.34	1.5E-06
xyloglucan glycosyltransferase 4 (Cellulose synthase-like C4)	AT3G28180	Cell Wall	-6.04	6.1E-05
arabinogalactan protein 21	AT1G55330	Cell Wall	-6.52	0.00167
arabinogalactan protein 20	AT3G61640	Cell Wall	-6.68	0.00019
leucine-rich repeat extensin-like protein 3	AT4G13340	Cell Wall	-7.49	0.00182
Lysine-rich arabinogalactan protein 18	AT4G37450	Cell Wall	-7.70	5.7E-05
peroxidase 31	AT3G28200	Cell wall	-8.12	3.4E-07
arabinogalactan protein 1	AT5G64310	Cell Wall	-8.43	0.00078
fasciclin-like arabinogalactan protein 13	AT5G44130	Cell Wall	-8.84	0.00028
Lysine-rich arabinogalactan protein	AT2G23130	Cell Wall	-10.06	0.00023
uncharacterized protein	AT4G35320	Cell wall	-11.01	7.4E-07
uncharacterized protein	AT2G33570	Cell wall	-11.37	3.7E-06
expansin-like A2 (EXLA2)	AT4G38400	Cell Wall	-12.12	1.7E-05
pectinesterase 25	AT3G10720	Cell Wall	-13.50	5.5E-07
expansin-like A3	AT3G45960	Cell Wall	-27.84	0.00022
expansin-like A1	AT3G45970	Cell Wall	-53.94	0.00017
feruloyl CoA ortho-hydroxylase 1	AT3G13610	Cell cycle	34.02	3.3E-07
ankyrin repeat-containing protein	AT4G14390	Cell cycle	11.76	0.00414
ankyrin repeat family protein	AT5G54610	Cell cycle	10.53	0.00042
microtubule-associated protein 18	AT5G44610	Cell cycle	9.73	0.03853
centrin 2	AT4G37010	Cell cycle	9.61	0.00044
ankyrin repeat-containing protein	AT4G03450	Cell cycle	3.90	0.00801
DNAse I-like superfamily protein	AT2G37440	Cell cycle	3.67	0.00033
ankyrin repeat-containing protein	AT1G10340	Cell cycle	3.11	0.00421
protein accelerated cell death 6	AT4G14400	Cell cycle	2.80	0.00104
ankyrin repeat-containing protein	AT4G03460	Cell cycle	2.74	0.02552
Ca-2+ dependent nuclease	AT3G56170	Cell cycle	2.69	0.00113
autophagy-related protein 8e	AT2G45170	Cell cycle	2.41	0.00858
actin depolymerizing factor 9	AT4G34970	Cell cycle	2.35	0.00136
DNAse I-like superfamily protein	AT3G21530	Cell cycle	2.31	0.00308
endonuclease/exonuclease/phosphatase family protein	AT4G36050	Cell cycle	2.27	0.00295
ankyrin repeat-containing protein	AT3G04140	Cell cycle	2.22	0.04804
actin-related protein 8	AT5G56180	Cell cycle	2.19	0.0015

MA3 domain-containing protein	AT5G63190	Cell cycle	2.14	0.00234
cyclin-dependent kinase inhibitor 2	AT3G50630	Cell cycle	2.05	0.0264
cyclin-dependent kinase inhibitor 4	AT2G32710	Cell cycle	2.00	0.00846
FIBRILLIN 4	AT3G23400	Cell cycle	-2.00	0.00011
unknown protein (At1g03420)	AT1G03420	Cell cycle	-2.01	0.00022
cell division protein ftsZ-like protein 1	AT5G55280	Cell cycle	-2.02	0.01059
Tubulin/FtsZ family protein	AT3G52750	Cell cycle	-2.03	0.03451
protein ABIL1	AT2G46225	Cell cycle	-2.04	0.0137
protein BRCA2-like B	AT5G01630	Cell cycle	-2.04	0.04369
histone H2B	AT2G37470	Cell cycle	-2.08	0.01198
armadillo repeat-containing kinesin-like protein 2	AT1G01950	Cell cycle	-2.14	4.3E-05
mitotic spindle checkpoint protein MAD2	AT3G25980	Cell cycle	-2.14	0.00458
cyclin p1;1	AT3G63120	Cell cycle	-2.17	0.0139
fimbrin 1	AT4G26700	Cell cycle	-2.18	0.04922
microtubule-associated protein, RP/EB family member 1B	AT5G62500	Cell cycle	-2.25	0.00533
proline-rich family protein	AT5G07020	Cell cycle	-2.35	0.00075
actin binding Calponin homology domain-containing protein	AT5G55400	Cell cycle	-2.40	0.03016
transducin/WD40 domain-containing protein	AT3G51930	Cell cycle	-2.49	0.0016
3-methyladenine glycosylase I	AT5G44680	Cell cycle	-2.54	0.03403
holliday junction resolvase	AT1G12244	Cell cycle	-2.55	0.00694
microtubule end binding protein EB1A	AT3G47690	Cell cycle	-2.61	0.0007
Ankyrin repeat family protein	AT1G14480	Cell cycle	-2.88	0.04369
ankyrin repeat family protein	AT3G24530	Cell cycle	-2.99	9E-06
histone H3	AT5G10390	Cell cycle	-3.20	0.00772
Ankyrin repeat family protein	AT5G54710	Cell cycle	-3.73	0.00174
cyclin-A1-2	AT1G77390	Cell cycle	-4.57	2.1E-07
copia-like retrotransposon	AT3G28160	Cell cycle	-4.61	0.00059
ankyrin repeat family protein	AT1G11740	Cell cycle	-4.64	0.02953
stress-induced protein KIN2	AT5G15970	Cell cycle	-4.83	0.01231
tetratricopeptide repeat domain-containing protein	AT3G27960	Cell cycle	-5.10	7.3E-05
Ankyrin repeat family protein	AT2G24600	Cell cycle	-8.01	3.9E-05
CLAVATA3/ESR (CLE)-related protein 2	AT1G63245	Development	8.47	0.00018
histone-lysine N-methyltransferase MEDEA	AT1G02580	Development	6.18	3.9E-06
major facilitator protein	AT2G39210	Development	4.62	0.0003
nodulin MtN21 /EamA-like transporter family protein	AT5G40240	Development	4.41	0.00085
protein CLAVATA3/ESR-related 21	AT5G64800	Development	4.37	3.7E-05
nodulin MtN21-like transporter UMAMIT38	AT4G15540	Development	3.36	0.00189
nodulin MtN21 /EamA-like transporter family protein	AT5G40230	Development	3.34	0.00094
uncharacterized protein	AT1G74940	Development	3.27	3.2E-05
cysteine/histidine-rich C1 domain-containing protein	AT5G43520	Development	3.25	0.03968
dormancy/auxin associated protein	AT1G56220	Development	3.04	0.00016
protein seedling plastid development 1	AT3G10420	Development	2.98	0.02291
ROTUNDIFOLIA like 17	AT1G13245	Development	2.77	0.0242
uncharacterized protein	AT4G17670	Development	2.51	9.4E-05
dormancy-associated protein-like 1	AT1G28330	Development	2.44	0.03312
MEI2-like protein 5	AT1G29400	Development	2.36	0.01638
gigantea protein (GI)	AT1G22770	Development	2.32	0.00454
nodulin MtN21-like transporter family protein UMAMIT45	AT3G28100	Development	2.23	0.04605
NO-associated protein 1	AT3G47450	Development	-2.02	0.02295
agamous-like MADS-box protein AGL15	AT5G13790	Development	-2.05	0.00262
Embryo-specific protein 3, (ATS3)	AT2G41475	Development	-2.07	0.00086
phytosulfokines 5 precursor	AT5G65870	Development	-2.14	0.01638
late embryogenesis abundant 3-like protein	AT1G02820	Development	-2.38	0.00036
antitermination NusB domain-containing protein	AT4G26370	Development	-2.41	0.01901
MAC/Perforin domain-containing protein	AT1G29690	Development	-2.46	0.00768
methyltransferase	AT5G10830	Development	-2.47	0.03694
nodulin MtN21 /EamA-like transporter family protein	AT3G45870	Development	-2.56	0.0148
maternal effect embryo arrest 18 protein	AT2G34090	Development	-2.56	0.00899
squamosa promoter-binding-like protein 13	AT5G50570	Development	-3.28	0.01014
bidirectional sugar transporter SWEET4	AT3G28007	Development	-3.44	0.01313
far-red-elongated hypocotyl1-like protein	AT5G02200	Development	-3.61	6.9E-05
HAD superfamily, subfamily IIIB acid phosphatase	AT5G44020	Development	-3.74	0.0002
leucine-rich repeat receptor-like protein CLAVATA2	AT1G65380	Development	-3.80	0.0011
desiccation-related protein LEA14	AT1G01470	Development	-4.01	0.00262
uncharacterized protein	AT4G30090	Development	-4.37	3.9E-05
tetraspanin10	AT1G63260	Development	-4.85	0.01051
senescence/dehydration related protein	AT2G17840	Development	-5.06	2.7E-05
N-MYC downregulated-like 3 protein	AT2G19620	Development	-6.13	3.5E-06
senescence/dehydration-associated protein	AT4G35985	Development	-6.81	0.00106
protein exordium like 5	AT2G17230	Development	-9.56	1.1E-06
NAC transcription factor protein family	AT1G69490	DNA binding and transcriptio	14.78	0.03376

C2H2-type zinc finger-containing protein	AT2G28710	DNA binding and transcriptio	11.04	0.00953
cysteine/histidine-rich C1 domain-containing protein	AT2G42060	DNA binding and transcriptio	10.16	0.00024
myb domain protein 122	AT1G74080	DNA binding and transcriptio	9.45	0.00044
myb family transcription factor	AT1G71030	DNA binding and transcriptio	8.15	2.7E-05
zinc finger CCCH domain-containing protein 49	AT4G29190	DNA binding and transcriptio	5.94	4E-07
WRKY transcription factor 63	AT1G66600	DNA binding and transcriptio	5.67	0.00037
LOB domain-containing protein 21	AT3G11090	DNA binding and transcriptio	5.30	0.0002
myb domain protein 45	AT3G48920	DNA binding and transcriptio	5.00	0.00026
PLATZ transcription factor family protein	AT1G21000	DNA binding and transcriptio	3.77	0.00047
transcription factor	AT2G02060	DNA binding and transcriptio	3.69	0.03419
PLATZ transcription factor domain-containing protein	AT1G32700	DNA binding and transcriptio	3.54	1E-05
PLATZ transcription factor family protein	AT1G76590	DNA binding and transcriptio	3.52	0.01691
Calcium-dependent lipid-binding (CaLB domain) family protein	AT1G70810	DNA binding and transcriptio	3.47	8.2E-05
homeobox protein BEL1-like protein	AT5G41410	DNA binding and transcriptio	3.47	0.00043
cyclic DOF factor 3	AT3G47500	DNA binding and transcriptio	3.31	0.01419
AT-hook motif nuclear-localized protein 1	AT4G12080	DNA binding and transcriptio	3.26	0.03734
homeobox-leucine zipper protein HAT9	AT2G22800	DNA binding and transcriptio	3.25	0.00323
myb domain protein 13	AT1G06180	DNA binding and transcriptio	3.18	0.00066
myb domain protein 85	AT4G22680	DNA binding and transcriptio	3.06	0.00036
homeobox protein 23	AT5G39760	DNA binding and transcriptio	3.05	0.00516
homeobox-leucine zipper protein ATHB-21	AT2G18550	DNA binding and transcriptio	2.80	0.01646
basic leucine zipper 9	AT5G24800	DNA binding and transcriptio	2.79	0.00776
zinc finger protein CONSTANS-LIKE 7	AT1G73870	DNA binding and transcriptio	2.76	0.00237
plant regulator RWP-RK family protein	AT4G35270	DNA binding and transcriptio	2.69	0.0071
transcription factor MYB3	AT1G22640	DNA binding and transcriptio	2.59	0.00017
transcription factor MYB86	AT5G26660	DNA binding and transcriptio	2.58	0.02364
GATA transcription factor 27	AT5G47140	DNA binding and transcriptio	2.54	0.00014
transcription factor HY5-like protein	AT3G17609	DNA binding and transcriptio	2.52	0.00319
cycling DOF factor 2	AT5G39660	DNA binding and transcriptio	2.50	0.0004
E2F/DP family winged-helix DNA-binding domain-containing protein	AT4G18870	DNA binding and transcriptio	2.49	0.00879
transcription factor bHLH148	AT3G06590	DNA binding and transcriptio	2.35	0.00883
transcription factor TGA7	AT1G77920	DNA binding and transcriptio	2.32	0.0421
transcription factor TGA1	AT5G65210	DNA binding and transcriptio	2.32	0.00099
SNF2 , helicase and zinc-finger domain-containing protein	AT1G11100	DNA binding and transcriptio	2.29	0.01386
AP2/B3-like transcriptional factor family protein	AT3G11580	DNA binding and transcriptio	2.29	0.03208
homeobox-leucine zipper protein ATHB-4	AT2G44910	DNA binding and transcriptio	2.26	0.00076
phospholipid-transporting ATPase 9	AT1G68710	DNA binding and transcriptio	2.24	0.01251
transcription factor HY5	AT5G11260	DNA binding and transcriptio	2.19	0.02271
BEL1-like homeodomain 10	AT1G19700	DNA binding and transcriptio	2.18	0.00091
RNA recognition motif-containing protein	AT4G17720	DNA binding and transcriptio	2.14	0.00183
protein indeterminate(ID)-domain 11	AT3G13810	DNA binding and transcriptio	2.12	0.0417
BEL1-like homeodomain 3	AT1G75410	DNA binding and transcriptio	2.06	7.4E-05
oxidation-related zinc Finger 1	AT2G19810	DNA binding and transcriptio	2.05	0.01949
glycine-rich RNA-binding protein 7	AT2G21660	DNA binding and transcriptio	2.05	0.00944
H/ACA ribonucleoprotein complex, subunit Gar1/Naf1 protein	AT5G18180	DNA binding and transcriptio	2.03	0.04645
transcription factor bHLH61	AT5G10570	DNA binding and transcriptio	2.02	0.00341
protein argonaute 7 (protein ZIPPY)	AT1G69440	DNA binding and transcriptio	2.02	0.00241
RING/FYVE/PHD zinc finger-containing protein	AT3G43230	DNA binding and transcriptio	2.01	0.03929
chloroplast RNA-binding protein 31B	AT5G50250	DNA binding and transcriptio	-2.00	0.00279
NOL1/NOP2/sun family protein / antitermination NusB domain-containing protein	AT3G13180	DNA binding and transcriptio	-2.02	0.00433
Nucleic acid-binding, OB-fold-like protein	AT3G59980	DNA binding and transcriptio	-2.03	0.00056
DNA-binding storekeeper protein-related transcriptional regulator	AT4G25210	DNA binding and transcriptio	-2.04	0.02412
TRANS-ACTING SIRNA3	AT3G17185	DNA binding and transcriptio	-2.05	0.03012
RNA-binding (RRM/RBD/RNP motifs) family protein	AT2G46780	DNA binding and transcriptio	-2.11	6.3E-06
transcription factor TCP7	AT5G23280	DNA binding and transcriptio	-2.22	4.2E-05
maternally expressed PAB C-terminal protein	AT3G19350	DNA binding and transcriptio	-2.24	0.01108
PLATZ transcription factor family protein	AT5G46710	DNA binding and transcriptio	-2.27	0.00462
RNA recognition motif-containing protein	AT4G09040	DNA binding and transcriptio	-2.29	0.0036
RING/FYVE/PHD zinc finger-containing protein	AT1G77250	DNA binding and transcriptio	-2.29	0.02472
myb domain protein 70	AT2G23290	DNA binding and transcriptio	-2.30	0.02984
Rossmann-fold NAD(P)-binding domain-containing protein	AT4G35250	DNA binding and transcriptio	-2.31	0.0254
C2H2 and C2HC zinc finger-containing protein	AT4G17810	DNA binding and transcriptio	-2.32	0.00475
myb domain protein 76	AT5G07700	DNA binding and transcriptio	-2.35	0.01161
Dof zinc finger protein DOF5.6	AT5G62940	DNA binding and transcriptio	-2.38	0.00118
ribonuclease Z	AT2G04530	DNA binding and transcriptio	-2.39	0.00077
ribonuclease III-like protein	AT1G55140	DNA binding and transcriptio	-2.41	0.0128
uncharacterized protein	AT3G20300	DNA binding and transcriptio	-2.42	0.0296
basic helix-loop-helix domain-containing protein	AT2G42380	DNA binding and transcriptio	-2.50	0.01251
RNA recognition motif-containing protein	AT1G67950	DNA binding and transcriptio	-2.50	0.00356
CIA2-like transcription factor	AT4G25990	DNA binding and transcriptio	-2.50	9.9E-05
myb family transcription factor	AT3G16350	DNA binding and transcriptio	-2.51	0.00266

RNA methyltransferase	AT5G10620	DNA binding and transcriptio	-2.58	0.01223
ethylene-responsive transcription factor ERF061	AT1G64380	DNA binding and transcriptio	-2.59	0.02166
ribosome-binding factor A	AT4G34730	DNA binding and transcriptio	-2.66	0.00162
bZIP protein	AT5G04840	DNA binding and transcriptio	-2.89	0.00036
LOB domain-containing protein 38	AT3G49940	DNA binding and transcriptio	-3.00	0.02464
transcription factor BHLH32	AT3G25710	DNA binding and transcriptio	-3.06	0.01545
two-component response regulator ARR15	AT1G74890	DNA binding and transcriptio	-3.09	0.04131
activation-tagged BRI1 suppressor 1-interacting factor 1	AT3G05800	DNA binding and transcriptio	-3.15	0.015
PLATZ transcription factor family protein	AT1G43000	DNA binding and transcriptio	-3.22	0.00347
transcription factor jumonji family protein / zinc finger (C5HC2 type) family protein	AT5G46910	DNA binding and transcriptio	-3.24	0.00017
protein AGAMOUS-like 87	AT1G22590	DNA binding and transcriptio	-3.51	0.00854
homeodomain-like transcriptional regulator	AT5G58900	DNA binding and transcriptio	-3.91	0.00595
zinc finger transcription factor BZS1	AT4G39070	DNA binding and transcriptio	-3.97	0.00343
Myb transcription factor	AT5G62470	DNA binding and transcriptio	-3.99	0.00063
RNA recognition motif-containing protein	AT1G22330	DNA binding and transcriptio	-4.10	1.3E-06
homeobox-leucine zipper protein HAT1	AT4G17460	DNA binding and transcriptio	-4.31	0.00094
transcription factor bHLH96	AT1G72210	DNA binding and transcriptio	-4.73	0.00052
myb domain protein 77	AT3G50060	DNA binding and transcriptio	-4.78	2E-05
two-component response regulator ARR7	AT1G19050	DNA binding and transcriptio	-4.81	0.00747
RNA recognition motif-containing protein	AT1G03457	DNA binding and transcriptio	-5.48	0.00125
two-component response regulator ARR6	AT5G62920	DNA binding and transcriptio	-5.49	0.00065
heat shock transcription factor A3	AT5G03720	DNA binding and transcriptio	-6.31	2E-05
GATA transcription factor 8	AT3G54810	DNA binding and transcriptio	-6.85	2.3E-08
U-box domain-containing protein	AT1G66160	DNA binding and transcriptio	-7.97	0.00075
transcription factor bHLH137	AT5G50915	DNA binding and transcriptio	-11.99	9.9E-07
dehydration-responsive element-binding protein 1F	AT1G12610	DNA binding and transcriptio	-22.43	0.0244
ethylene-responsive transcription factor ERF019	AT1G22810	DNA binding and transcriptio	-51.21	3E-06
ethylene-responsive transcription factor ERF012	AT1G21910	DNA binding and transcriptio	-53.34	1.4E-05
carboxylesterase 6	AT1G68620	Metabolism	44.43	0.01129
methionine sulfoxide reductase B8	AT4G21840	Metabolism	38.32	0.00042
flavonol synthase 5	AT5G63600	Metabolism	16.36	0.00433
branched-chain-amino-acid aminotransferase 2	AT1G10070	Metabolism	16.25	0.00018
bifunctional nitrilase/nitrile hydratase NIT4	AT5G22300	Metabolism	14.68	0.03112
HXXXD-type acyl-transferase-like protein	AT5G42830	Metabolism	13.64	4.8E-05
aldehyde oxidase 1	AT5G20960	Metabolism	12.31	1.3E-06
2-oxoglutarate (2OG) and Fe(II)-dependent oxygenase superfamily protein	AT2G36690	Metabolism	11.15	0.00074
glutamate decarboxylase 1	AT5G17330	Metabolism	10.28	0.00015
beta-fructofuranosidase, insoluble isoenzyme CWINV6	AT5G11920	Metabolism	9.91	0.01043
beta-fructofuranosidase, insoluble isoenzyme CWINV1	AT3G13790	Metabolism	9.63	8.3E-05
Inositol monophosphatase family protein	AT5G09290	Metabolism	9.21	0.00039
glutamine synthetase 1;1	AT5G37600	Metabolism	8.79	1.2E-07
Terpenoid cyclases family protein	AT1G66960	Metabolism	8.08	0.00038
glycosyl hydrolase 9A2	AT1G65610	Metabolism	6.81	0.00673
glutamine amidotransferase	AT1G15040	Metabolism	6.72	0.03368
long-chain acyl-CoA synthetase	AT1G64400	Metabolism	6.58	2.5E-05
UDP-glycosyltransferase 76E4	AT3G46690	Metabolism	6.42	0.00086
phosphoglycerate mutase-like protein	AT1G09935	Metabolism	6.02	0.0004
lipid-transfer protein/seed storage	AT3G22620	Metabolism	5.99	0.02692
GDSL esterase/lipase 4	AT3G14225	Metabolism	5.80	0.00059
tryptophan N-monooxygenase 1	AT4G39950	Metabolism	5.37	0.00355
O-methyltransferase-like protein	AT1G33030	Metabolism	5.23	0.00044
alpha 1,4-glycosyltransferase family protein	AT5G01250	Metabolism	5.12	0.00179
tc|GB|AC007887.9|AAF79366.1 F15O4.44	AT1G35513	Metabolism	5.01	0.00054
beta-galactosidase 4	AT5G56870	Metabolism	4.90	0.00031
GDSL esterase/lipase	AT5G03610	Metabolism	4.59	0.00353
nudix hydrolase 6	AT2G04450	Metabolism	4.44	0.00036
beta glucosidase 17	AT2G44480	Metabolism	4.15	0.01614
pyruvate kinase	AT5G63680	Metabolism	4.14	1.3E-06
glucuronidase 1	AT5G61250	Metabolism	4.13	1.5E-05
flavodoxin-like quinone reductase 1	AT5G54500	Metabolism	3.95	5.7E-05
glucuronosyl transferase-like protein	AT3G46700	Metabolism	3.89	0.03295
NAD(P)-binding Rossmann-fold superfamily protein	AT5G52810	Metabolism	3.89	6.2E-05
glycolate oxidase	AT4G18360	Metabolism	3.63	0.00318
glutamine synthetase 1;4	AT5G16570	Metabolism	3.62	0.01467
phospholipase A1-Igamma2	AT2G30550	Metabolism	3.27	0.0001
cytochrome P450, family 706, subfamily A, polypeptide 4	AT4G12300	Metabolism	3.22	0.00178
Sphingosine-1-phosphate lyase	AT1G27980	Metabolism	3.21	0.00011
copper amine oxidase	AT3G43670	Metabolism	3.20	0.00279
Long-chain-alcohol oxidase FAO4B	AT4G28570	Metabolism	3.18	0.00236
calcium-independent phospholipase A	AT4G19860	Metabolism	3.11	2.8E-05
indole-3-glycerol phosphate synthase	AT2G04400	Metabolism	3.11	0.00739

D-3-phosphoglycerate dehydrogenase	AT4G34200	Metabolism	3.09	9.8E-05
lysophospholipase 2	AT1G52760	Metabolism	3.01	1.9E-05
anthranilate synthase component I-1	AT5G05730	Metabolism	3.00	0.04131
nudix hydrolase 5	AT2G04430	Metabolism	2.75	8.8E-06
pfkB-like carbohydrate kinase family protein	AT5G43910	Metabolism	2.69	0.00049
pyruvate decarboxylase-2	AT5G54960	Metabolism	2.66	0.03268
acyl activating enzyme 5	AT5G16370	Metabolism	2.65	0.00702
adenine nucleotide alpha hydrolases-domain containing protein kinase	AT1G77280	Metabolism	2.61	0.00636
P-loop containing nucleoside triphosphate hydrolases superfamily protein	AT1G04280	Metabolism	2.53	0.00034
HXXXD-type acyl-transferase family protein	AT1G28680	Metabolism	2.48	0.00322
acyl-coenzyme A oxidase 4	AT3G51840	Metabolism	2.47	0.00362
aldose 1-epimerase	AT3G47800	Metabolism	2.46	0.00425
phosphoglycerate mutase family protein	AT3G60450	Metabolism	2.45	0.00048
callose synthase 1	AT1G05570	Metabolism	2.43	0.0292
copper amine oxidase 1	AT1G62810	Metabolism	2.42	0.00152
strigolactone esterase D14	AT3G03990	Metabolism	2.40	0.00454
alkaline/neutral invertase CINV1	AT1G35580	Metabolism	2.38	0.00138
methyltransferase-like protein	AT1G69520	Metabolism	2.37	0.00027
beta-1,3-galactosyltransferase 2	AT1G05170	Metabolism	2.37	0.00151
GDSL esterase/lipase	AT1G28580	Metabolism	2.36	0.01888
melibiase family protein	AT3G56310	Metabolism	2.34	0.00377
EPSP synthase	AT2G45300	Metabolism	2.30	0.0039
long-chain acyl-CoA synthetase 7	AT5G27600	Metabolism	2.29	0.03762
aldose 1-epimerase family protein	AT4G25900	Metabolism	2.25	0.00303
Hydrolases superfamily protein	AT4G00500	Metabolism	2.23	0.00052
glutathione S-conjugate transporting ATPase	AT1G30400	Metabolism	2.21	0.01174
carboxylesterase 2	AT1G47480	Metabolism	2.19	0.01247
SAL3 phosphatase	AT5G63990	Metabolism	2.17	0.00193
enoyl-CoA hydratase 2	AT1G76150	Metabolism	2.16	0.00223
cysteine synthase C1	AT3G61440	Metabolism	2.13	0.02206
aldehyde dehydrogenase 2B4	AT3G48000	Metabolism	2.12	0.0203
digalactosyldiacylglycerol synthase 1	AT3G11670	Metabolism	2.11	0.00283
adenylate kinase	AT2G39270	Metabolism	2.09	0.0019
stearoyl-acyl-carrier-protein desaturase	AT5G16230	Metabolism	2.04	0.01965
UDP-glycosyltransferase-like protein	AT2G18560	Metabolism	2.03	0.03463
Cyclopropane-fatty-acyl-phospholipid synthase	AT3G23530	Metabolism	2.02	0.03674
Dihydroxyacetone kinase	AT1G48430	Metabolism	2.01	0.0211
UDP-glycosyltransferase 72B1	AT4G01070	Metabolism	2.00	0.03348
DNAse I-like superfamily protein	AT1G71710	Metabolism	-2.00	0.02972
acyl carrier protein 4	AT4G25050	Metabolism	-2.01	0.03268
glycine dehydrogenase	AT4G33010	Metabolism	-2.02	0.0292
pseudouridine synthase family protein	AT5G14460	Metabolism	-2.02	0.01945
dihydrolipoyl dehydrogenase	AT4G16155	Metabolism	-2.03	0.00091
aminotransferase	AT1G77670	Metabolism	-2.03	2.8E-05
myrosinase 1	AT5G26000	Metabolism	-2.05	0.00813
cytokinin riboside 5'-monophosphate phosphoribohydrolase LOG7	AT5G06300	Metabolism	-2.07	0.01345
Pseudouridine synthase family protein	AT3G43340	Metabolism	-2.07	0.03647
GDSL esterase/lipase	AT1G09390	Metabolism	-2.09	0.04843
photosystem I light harvesting complex protein 5	AT1G45474	Metabolism	-2.10	0.00727
2Fe-2S iron-sulfur cluster binding domain-containing protein	AT4G32590	Metabolism	-2.10	0.04051
3-ketoacyl-CoA synthase 11	AT2G26640	Metabolism	-2.10	0.00385
myo-inositol monophosphatase like 1	AT1G31190	Metabolism	-2.14	0.0421
adenosine kinase 1	AT3G09820	Metabolism	-2.14	0.00205
3-ketoacyl-CoA synthase 9	AT2G16280	Metabolism	-2.15	7.1E-05
GDSL esterase/lipase LIP-4	AT1G56670	Metabolism	-2.15	0.0001
fatty acid hydroxylase 2	AT4G20870	Metabolism	-2.15	0.02146
Enoyl-ACP reductase 1	AT2G30200	Metabolism	-2.16	0.04961
Enoyl-ACP reductase 1	AT2G05990	Metabolism	-2.18	0.01157
N2,N2-dimethylguanosine tRNA methyltransferase	AT3G56330	Metabolism	-2.18	0.00182
glucose-1-phosphate adenylyltransferase small subunit	AT5G48300	Metabolism	-2.19	0.01174
GDSL esterase/lipase	AT5G45920	Metabolism	-2.21	0.0296
bifunctional aspartokinase/homoserine dehydrogenase 1	AT1G31230	Metabolism	-2.21	0.00137
mevalonate kinase	AT5G27450	Metabolism	-2.22	0.02668
glycoside hydrolase	AT3G20440	Metabolism	-2.24	0.0276
1,4-alpha-glucan branching enzyme	AT5G03650	Metabolism	-2.25	0.00887
glycosyltransferase 18	AT5G62220	Metabolism	-2.26	0.01682
low psii accumulation2 protein	AT5G51545	Metabolism	-2.26	0.00127
branched-chain-amino-acid aminotransferase 3	AT3G49680	Metabolism	-2.26	0.00271
tRNA pseudouridine synthase	AT2G30320	Metabolism	-2.27	0.00236
1-(5-phosphoribosyl)-5-	AT2G36230	Metabolism	-2.28	0.02082
delta1-pyrroline-5-carboxylate synthase 1	AT2G39800	Metabolism	-2.29	0.00279

glutamate-1-semialdehyde 2,1-aminomutase 2	AT3G48730	Metabolism	-2.30	0.00069
pfkB-like carbohydrate kinase family protein	AT1G66430	Metabolism	-2.31	0.00198
core-2/I-branching beta-1,6-N-acetylglucosaminyltransferase family protein	AT3G15350	Metabolism	-2.37	0.00063
ferredoxin-like protein	AT1G02180	Metabolism	-2.40	0.04051
photosystem II reaction center PsbP family protein	AT1G76450	Metabolism	-2.42	0.00062
1-deoxy-D-xylulose 5-phosphate reductoisomerase	AT5G62790	Metabolism	-2.44	0.00158
aldose 1-epimerase family protein	AT5G66530	Metabolism	-2.45	0.00148
phosphoglycerate mutase family protein	AT5G62840	Metabolism	-2.48	0.0016
fatty acid desaturase 7	AT3G11170	Metabolism	-2.49	0.00167
glucan endo-1,3-beta-glucosidase 11	AT1G32860	Metabolism	-2.52	2.5E-05
NAD(P)-linked oxidoreductase superfamily protein	AT1G04420	Metabolism	-2.55	0.00454
esterase/lipase/thioesterase family protein	AT3G50790	Metabolism	-2.61	0.00027
3-hydroxyacyl-ACP dehydratase	AT2G22230	Metabolism	-2.62	0.00015
PsbP domain-containing protein 5	AT5G11450	Metabolism	-2.64	0.00065
bifunctional sn-glycerol-3-phosphate 2-O-acyltransferase/phosphatase	AT4G00400	Metabolism	-2.65	0.0016
chloroplast thylakoid lumen protein	AT4G02530	Metabolism	-2.71	0.00035
2-C-methyl-D-erythritol 4-phosphate cytidylyltransferase	AT2G02500	Metabolism	-2.72	0.00135
fructokinase-like 1	AT3G54090	Metabolism	-2.78	0.00928
3-ketoacyl-CoA synthase 20	AT5G43760	Metabolism	-2.83	0.00348
cysteine synthase 26	AT3G03630	Metabolism	-2.83	0.00226
nudix hydrolase 7	AT4G12720	Metabolism	-2.84	2.1E-05
cell wall / vacuolar inhibitor of fructosidase 2	AT5G64620	Metabolism	-2.84	0.00027
tropinone reductase-like protein	AT2G29290	Metabolism	-2.85	0.03212
FAD/NAD(P)-binding oxidoreductase domain-containing protein	AT1G57770	Metabolism	-2.91	9.5E-05
lipase domain-containing protein	AT5G50890	Metabolism	-2.91	0.00021
Monogalactosyldiacylglycerol synthase 1	AT4G31780	Metabolism	-2.92	0.01763
trehalose-phosphate phosphatase H	AT4G39770	Metabolism	-2.92	0.00149
3-ketoacyl-CoA synthase 3	AT1G07720	Metabolism	-3.00	0.00916
phosphoenolpyruvate carboxylase family protein	AT1G21440	Metabolism	-3.05	0.02448
fatty acyl-CoA reductase 1	AT5G22500	Metabolism	-3.09	8.3E-05
nudix hydrolase 8	AT5G47240	Metabolism	-3.13	0.00035
isopropylmalate dehydrogenase 1	AT5G14200	Metabolism	-3.27	0.02984
fatty acid hydroxylase 1	AT2G34770	Metabolism	-3.30	9.3E-06
fatty-acid-binding protein 3	AT1G53520	Metabolism	-3.33	0.02275
arabinogalactan protein 41	AT5G24105	Metabolism	-3.38	0.02158
adenine phosphoribosyl transferase 3	AT4G22570	Metabolism	-3.46	0.00353
galacturonosyltransferase-like 6	AT4G02130	Metabolism	-3.60	0.00032
flavin-containing monooxygenase FMO GS-OX3	AT1G62560	Metabolism	-3.65	0.00204
UDP-glycosyltransferase family protein	AT5G04480	Metabolism	-3.77	0.00011
sodium/metabolite cotransporter BASS5	AT4G12030	Metabolism	-3.77	0.03072
isoprenylcysteine alpha-carbonyl methylesterase ICMEL2	AT3G02410	Metabolism	-3.81	0.04764
carboxyvinyl-carboxyphosphonate phosphorylmutase	AT1G77060	Metabolism	-3.81	2.6E-06
nucleotide-diphospho-sugar transferase	AT1G64980	Metabolism	-3.92	3.9E-05
Type I inositol-1,4,5-trisphosphate 5-phosphatase 2	AT4G18010	Metabolism	-3.93	0.00677
hexokinase-like 1	AT1G50460	Metabolism	-4.01	0.00017
HXXXD-type acyl-transferase-like protein	AT2G39980	Metabolism	-4.02	0.00025
galacturonosyltransferase-like 8	AT1G24170	Metabolism	-4.45	3.3E-07
galacturonosyltransferase 15	AT3G58790	Metabolism	-4.53	7.9E-06
RNA-directed DNA methylase	AT1G13790	Metabolism	-4.54	0.00562
Chalcone-flavanone isomerase family protein	AT5G05270	Metabolism	-4.60	0.00537
GDSL esterase/lipase	AT4G28780	Metabolism	-4.61	0.04804
isopropylmalate isomerase 1	AT3G58990	Metabolism	-4.66	0.04922
S-adenosylmethionine synthase 4	AT3G17390	Metabolism	-4.81	3.1E-05
methylthioalkylmalate synthase 1	AT5G23010	Metabolism	-4.82	0.02508
fatty acid desaturase 8	AT5G05580	Metabolism	-4.83	0.01022
3-ketoacyl-CoA synthase 4	AT1G19440	Metabolism	-4.91	2.9E-05
plant glycogenin-like starch initiation protein 7	AT2G35710	Metabolism	-4.97	7.9E-07
oxidoreductase	AT3G60290	Metabolism	-5.05	3.8E-05
trehalose-phosphate phosphatase-like protein	AT5G51460	Metabolism	-5.12	5.1E-08
isopropylmalate isomerase 2	AT2G43100	Metabolism	-5.55	0.00731
O-glycosyl hydrolases family 17 protein	AT1G64760	Metabolism	-6.83	1.1E-06
phosphatidylinositol:ceramide inositolphosphotransferase 1	AT3G54020	Metabolism	-6.83	2.3E-06
3-ketoacyl-CoA synthase 1	AT1G01120	Metabolism	-6.84	9E-06
galacturonosyltransferase-like 9	AT1G70090	Metabolism	-7.01	3.2E-09
glutathione S-transferase TAU 20	AT1G78370	Metabolism	-7.34	0.00064
flavin-containing monooxygenase FMO GS-OX1	AT1G65860	Metabolism	-7.61	0.00039
methionine sulfoxide reductase B6	AT4G04840	Metabolism	-7.84	0.00104
acyl-CoA sterol acyl transferase 1	AT3G51970	Metabolism	-8.12	0.00029
inositol oxygenase 1	AT1G14520	Metabolism	-10.61	5.4E-08
cytidine/deoxycytidylate deaminase-like protein	AT4G29610	Metabolism	-16.05	3.1E-05
sn-glycerol-3-phosphate 2-O-acyltransferase	AT4G01950	Metabolism	-21.69	2.1E-07

aspartyl protease family protein	AT5G48430	Protein synthesis, modificatio	75.86	3.9E-05
AAA-type ATPase family protein	AT3G28510	Protein synthesis, modificatio	43.75	0.0425
aspartyl protease family protein	AT1G44130	Protein synthesis, modificatio	27.84	8.2E-07
aspartyl protease family protein	AT3G51330	Protein synthesis, modificatio	10.80	2E-05
E3 ubiquitin-protein ligase ATL41	AT2G42360	Protein synthesis, modificatio	10.53	0.00011
U-box domain-containing protein 36	AT3G61390	Protein synthesis, modificatio	10.46	7.3E-08
matrix metalloproteinase	AT1G70170	Protein synthesis, modificatio	10.45	0.00042
chaperone protein dnaJ 11	AT4G36040	Protein synthesis, modificatio	10.05	0.00066
exocyst subunit exo70 family protein H2	AT2G39380	Protein synthesis, modificatio	9.92	0.00028
Subtilase 3.5	AT1G32940	Protein synthesis, modificatio	6.15	5.8E-06
concanavalin A-like lectin kinase-like protein	AT3G45410	Protein synthesis, modificatio	6.07	0.00143
AAA-ATPase 1	AT5G40010	Protein synthesis, modificatio	6.03	0.00113
AAA-type ATPase family protein	AT5G57480	Protein synthesis, modificatio	5.66	0.00313
heat shock protein 70	AT5G02490	Protein synthesis, modificatio	5.54	0.04051
metacaspase 2	AT4G25110	Protein synthesis, modificatio	5.47	0.0001
F-box/kelch-repeat protein	AT2G44130	Protein synthesis, modificatio	5.46	0.00673
aspartyl protease family protein	AT5G10760	Protein synthesis, modificatio	5.08	0.00466
calcium-binding protein CML21	AT4G26470	Protein synthesis, modificatio	4.86	0.0006
ubiquitin-like protein	AT1G53980	Protein synthesis, modificatio	4.67	0.00916
vacuolar-processing enzyme gamma	AT4G32940	Protein synthesis, modificatio	4.61	8.5E-05
F-box/kelch-repeat protein	AT1G80440	Protein synthesis, modificatio	4.49	1.8E-05
RING/U-box domain-containing protein	AT1G63840	Protein synthesis, modificatio	3.95	0.0004
RING finger domain-containing protein	AT1G14200	Protein synthesis, modificatio	3.85	0.00019
RING-H2 finger protein ATL70	AT2G35910	Protein synthesis, modificatio	3.70	0.00014
C3HC4-type RING finger-containing protein	AT1G08050	Protein synthesis, modificatio	3.60	0.03694
ARM repeat superfamily protein	AT5G67340	Protein synthesis, modificatio	3.53	0.00035
uncharacterized protein	AT3G01175	Protein synthesis, modificatio	3.45	0.00049
Protein kinase superfamily protein	AT1G66460	Protein synthesis, modificatio	3.42	0.01006
F-box domain-containing protein	AT5G18780	Protein synthesis, modificatio	3.41	9.6E-06
RING/U-box domain-containing protein	AT1G26800	Protein synthesis, modificatio	3.26	0.01067
P-loop containing nucleoside triphosphate hydrolases superfamily protein	AT5G17760	Protein synthesis, modificatio	3.22	0.00057
F-box/kelch-repeat protein	AT1G15670	Protein synthesis, modificatio	3.18	0.0184
Unknown	AT1G18670	Protein synthesis, modificatio	3.12	0.01018
RING-H2 finger protein	AT2G42350	Protein synthesis, modificatio	3.03	0.0316
RNI-like superfamily protein	AT4G30640	Protein synthesis, modificatio	3.00	0.00099
adenine nucleotide alpha hydrolases-domain containing protein kinase	AT1G21590	Protein synthesis, modificatio	2.97	0.00541
autophagy-related protein 8a	AT4G21980	Protein synthesis, modificatio	2.96	0.00833
uncharacterized protein	AT1G21670	Protein synthesis, modificatio	2.96	0.00677
beta-1,3-galactosyltransferase 7	AT1G77810	Protein synthesis, modificatio	2.92	1.2E-05
flavin-binding, kelch repeat, f box 1	AT1G68050	Protein synthesis, modificatio	2.87	0.00528
RING/U-box domain-containing protein	AT4G26580	Protein synthesis, modificatio	2.78	0.01264
RING-H2 finger protein ATL79	AT5G47610	Protein synthesis, modificatio	2.76	0.00124
F-box stress induced protein 2	AT4G21510	Protein synthesis, modificatio	2.63	4.4E-05
RING/U-box domain-containing protein	AT5G55970	Protein synthesis, modificatio	2.61	0.00077
autophagy substrate NBR1	AT4G24690	Protein synthesis, modificatio	2.61	0.00066
F-box/kelch-repeat protein	AT3G23880	Protein synthesis, modificatio	2.60	0.00024
E3 ubiquitin-protein ligase RING1	AT5G10380	Protein synthesis, modificatio	2.59	9.4E-05
Polyubiquitin	AT4G05320	Protein synthesis, modificatio	2.59	0.00805
F-box/kelch-repeat protein	AT1G23390	Protein synthesis, modificatio	2.58	0.00013
RING/U-box domain-containing protein	AT3G47160	Protein synthesis, modificatio	2.57	0.00301
transmembrane Fragile-X-F-associated protein	AT1G68820	Protein synthesis, modificatio	2.56	0.04843
histone H3 K4-specific methyltransferase SET7/9 family protein	AT1G77660	Protein synthesis, modificatio	2.55	0.00681
RING/FYVE/PHD zinc finger-containing protein	AT2G37950	Protein synthesis, modificatio	2.54	0.00172
F-box/kelch-repeat protein	AT1G51550	Protein synthesis, modificatio	2.50	0.00015
cathepsin B-like cysteine protease	AT4G01610	Protein synthesis, modificatio	2.48	8.2E-05
peptidase C15, pyroglutamyl peptidase I-like protein	AT1G23440	Protein synthesis, modificatio	2.46	0.00203
beta-endo-N-acetyglucosaminidase	AT5G05460	Protein synthesis, modificatio	2.43	0.00199
DWD motif protein	AT3G45620	Protein synthesis, modificatio	2.42	7.6E-05
RING/U-box superfamily protein	AT5G24870	Protein synthesis, modificatio	2.39	0.00312
RING/U-box domain-containing protein	AT3G06330	Protein synthesis, modificatio	2.35	0.00084
F-box/kelch-repeat protein	AT3G24760	Protein synthesis, modificatio	2.34	0.00293
F-box protein	AT3G07870	Protein synthesis, modificatio	2.32	0.00026
papain family cysteine protease	AT4G16190	Protein synthesis, modificatio	2.27	0.00014
RING/U-box domain-containing protein	AT1G24440	Protein synthesis, modificatio	2.26	0.00628
E3 ubiquitin-protein ligase ARI5	AT1G05890	Protein synthesis, modificatio	2.26	0.02279
cysteine proteinase inhibitor 7	AT5G05110	Protein synthesis, modificatio	2.25	0.01989
histone deacetylase 8	AT1G08460	Protein synthesis, modificatio	2.24	0.00023
NEP1-interacting protein 2	AT2G17730	Protein synthesis, modificatio	2.24	0.01937
ADP-ribosylation factor B1B	AT5G17060	Protein synthesis, modificatio	2.23	2.5E-05
RING/U-box domain-containing protein	AT4G19670	Protein synthesis, modificatio	2.20	0.00673
aspartyl protease family protein	AT3G02740	Protein synthesis, modificatio	2.18	0.00252

PPPDE thiol peptidase family protein	AT3G07090	Protein synthesis, modificatio	2.13	0.03463
E3 ubiquitin-protein ligase ARI12	AT1G05880	Protein synthesis, modificatio	2.11	0.01157
RWD domain-containing protein	AT3G60300	Protein synthesis, modificatio	2.09	0.0006
ubiquitin receptor protein DSK2B	AT2G17200	Protein synthesis, modificatio	2.07	0.00801
ubiquitin-associated (UBA)/TS-N domain-containing protein	AT2G12550	Protein synthesis, modificatio	2.07	0.00019
C3H2C3-type RING E3 Ub ligase	AT4G23450	Protein synthesis, modificatio	2.06	0.00801
autophagy-related protein 8f	AT4G16520	Protein synthesis, modificatio	2.05	0.00358
RING-finger domain-containing protein	AT4G31450	Protein synthesis, modificatio	2.04	0.00586
peptidyl-prolyl cis-trans isomerase	AT4G17070	Protein synthesis, modificatio	2.04	0.01182
ubiquitin-like protein ATG12A	AT1G54210	Protein synthesis, modificatio	2.03	0.02066
RING/U-box domain-containing protein	AT1G55530	Protein synthesis, modificatio	2.03	0.01553
F-box/kelch-repeat protein OR23	AT4G03030	Protein synthesis, modificatio	2.00	0.00213
F-box protein 7	AT1G21760	Protein synthesis, modificatio	2.00	0.00784
RNI-like superfamily protein	AT5G07670	Protein synthesis, modificatio	2.00	0.04961
aspartyl protease family protein	AT3G52500	Protein synthesis, modificatio	-2.00	0.0059
uncharacterized protein	AT1G67700	Protein synthesis, modificatio	-2.00	0.04764
peptidyl-prolyl cis-trans isomerase CYP20-2	AT5G13120	Protein synthesis, modificatio	-2.00	0.0012
Matrixin family protein	AT1G59970	Protein synthesis, modificatio	-2.02	0.00154
plant organelle RNA recognition domain-containing protein	AT3G58520	Protein synthesis, modificatio	-2.03	0.00017
Initiation factor eIF-4 gamma, MA3	AT4G30680	Protein synthesis, modificatio	-2.03	0.02976
Co-chaperone GrpE family protein	AT1G36390	Protein synthesis, modificatio	-2.05	0.00998
casein kinase I-like 12	AT5G57015	Protein synthesis, modificatio	-2.05	0.00017
molecular chaperone Hsp40/DnaJ family protein	AT3G17830	Protein synthesis, modificatio	-2.06	0.03976
30S ribosomal protein S5	AT2G33800	Protein synthesis, modificatio	-2.09	0.01791
cysteine proteinase-like protein	AT3G02070	Protein synthesis, modificatio	-2.12	0.00203
50S ribosomal protein L13	AT1G78630	Protein synthesis, modificatio	-2.13	0.03284
RNA pseudourine synthase 6	AT4G21770	Protein synthesis, modificatio	-2.14	0.04961
ubiquitin-specific protease family C19-related protein	AT1G16860	Protein synthesis, modificatio	-2.15	0.00018
30S ribosomal protein S10	AT3G13120	Protein synthesis, modificatio	-2.18	0.00928
50S ribosomal protein L3-1	AT2G43030	Protein synthesis, modificatio	-2.18	0.00454
30S ribosomal protein 3-1	AT1G68590	Protein synthesis, modificatio	-2.19	0.02744
50S ribosomal protein L28	AT2G33450	Protein synthesis, modificatio	-2.22	0.02223
protein phosphatase 2C 58	AT4G28400	Protein synthesis, modificatio	-2.23	0.0186
protein disulfide-isomerase LQY1	AT1G75690	Protein synthesis, modificatio	-2.24	0.03929
translation elongation factor EF1B/ribosomal protein S6 family protein	AT1G64510	Protein synthesis, modificatio	-2.27	0.00479
photosystem II stability/assembly factor HCF136	AT5G23120	Protein synthesis, modificatio	-2.29	0.01219
50S ribosomal protein L5	AT4G01310	Protein synthesis, modificatio	-2.30	0.01573
ribosomal protein L35	AT2G24090	Protein synthesis, modificatio	-2.31	0.01755
peptidyl-prolyl cis-trans isomerase FKBP16-3	AT2G43560	Protein synthesis, modificatio	-2.32	0.00071
FKBP-type peptidyl-prolyl cis-trans isomerase 5	AT1G18170	Protein synthesis, modificatio	-2.35	0.00031
50S ribosomal protein L34	AT1G29070	Protein synthesis, modificatio	-2.42	0.00466
peptidyl-tRNA hydrolase family protein	AT5G38290	Protein synthesis, modificatio	-2.43	0.01317
chaperonin-60 alpha	AT2G28000	Protein synthesis, modificatio	-2.47	0.00809
RING/U-box domain-containing protein	AT1G67856	Protein synthesis, modificatio	-2.48	0.00475
plastid ribosomal protein S21	AT3G27160	Protein synthesis, modificatio	-2.55	0.02436
peptidyl-prolyl cis-trans isomerase FKBP17-3	AT1G73655	Protein synthesis, modificatio	-2.56	0.00788
50S ribosomal protein L21	AT1G35680	Protein synthesis, modificatio	-2.61	0.00365
FtsH extracellular protease	AT4G23940	Protein synthesis, modificatio	-2.63	0.00706
F-box protein	AT3G47030	Protein synthesis, modificatio	-2.71	0.03702
S2P-like metalloprotease	AT5G05740	Protein synthesis, modificatio	-2.75	0.00866
FKBP-type peptidyl-prolyl cis-trans isomerase 3	AT5G45680	Protein synthesis, modificatio	-2.75	0.00088
ER lumen protein retaining receptor-like protein	AT1G19970	Protein synthesis, modificatio	-2.77	0.00101
aspartyl protease family protein	AT3G54400	Protein synthesis, modificatio	-2.80	8.7E-05
Hypersensitive-induced response protein 2	AT1G69840	Protein synthesis, modificatio	-2.94	0.00508
50S ribosomal protein L19-2	AT5G47190	Protein synthesis, modificatio	-2.94	0.00219
F-box protein	AT1G78100	Protein synthesis, modificatio	-2.95	0.00433
RING/U-box domain-containing protein	AT1G45180	Protein synthesis, modificatio	-3.29	3.3E-06
50S ribosomal protein related protein	AT5G16200	Protein synthesis, modificatio	-3.34	0.00322
U-box domain-containing protein 6	AT1G24330	Protein synthesis, modificatio	-3.38	0.00797
peptidyl-prolyl cis-trans isomerase CYP37	AT3G15520	Protein synthesis, modificatio	-3.39	0.03176
U-box domain-containing protein 31	AT5G65920	Protein synthesis, modificatio	-3.66	0.0019
F-box/RNI-like superfamily protein	AT3G03030	Protein synthesis, modificatio	-3.78	0.0234
membrane-anchored ubiquitin-fold protein 4	AT3G26980	Protein synthesis, modificatio	-4.06	0.00045
RING-H2 finger protein ATL80	AT1G20823	Protein synthesis, modificatio	-4.13	0.00242
serine carboxypeptidase-like 34	AT5G23210	Protein synthesis, modificatio	-4.14	0.0421
RING-H2 finger protein ATL17	AT4G15975	Protein synthesis, modificatio	-4.49	0.00706
phenazine biosynthesis PhzC/PhzF family protein	AT4G02850	Protein synthesis, modificatio	-4.87	0.00094
ARM repeat superfamily protein	AT1G23030	Protein synthesis, modificatio	-5.22	0.00022
aspartyl protease family protein	AT1G66180	Protein synthesis, modificatio	-5.26	7.6E-06
serine protease inhibitor, Kazal-type family protein	AT4G01575	Protein synthesis, modificatio	-6.66	0.00023
chaperone DnaJ-domain containing protein	AT1G72416	Protein synthesis, modificatio	-7.84	7.1E-07

uncharacterized protein	AT3G59310	Protein synthesis, modificatio	-10.88	5.3E-08
RHOMBOID-like protein 7	AT4G23070	Protein synthesis, modificatio	-13.74	0.018
aspartyl protease family protein	AT4G16563	Protein synthesis, modificatio	-31.68	0.00131
FLG22-induced receptor-like kinase 1 (FRK1)	AT2G19190	Signaling	170.99	1.1E-05
LRR receptor-like protein kinase	AT1G51890	Signaling	34.47	8E-07
protein kinase-like protein	AT3G46280	Signaling	24.60	2.1E-05
calcium-transporting ATPase 12	AT3G63380	Signaling	16.22	3.5E-06
glutamate receptor 1.3	AT5G48410	Signaling	15.60	0.04329
Lectin-domain containing receptor kinase A4.2	AT5G01550	Signaling	14.11	0.00179
cysteine-rich receptor-like protein kinase 7	AT4G23150	Signaling	14.02	0.00023
concanavalin A-like lectin kinase-like protein	AT5G06740	Signaling	13.96	0.00012
glutamate receptor 2.5	AT5G11210	Signaling	10.97	0.0001
glutamate receptor 2.9	AT2G29100	Signaling	10.89	8.7E-07
leucine-rich repeat protein kinase	AT1G51800	Signaling	10.57	7.4E-05
glutamate receptor 1.2	AT5G48400	Signaling	9.92	9E-06
PAR1 protein	AT5G52390	Signaling	9.81	0.00211
LRR receptor-like serine/threonine-protein kinase	AT1G51860	Signaling	8.99	2.2E-06
Receptor-like protein kinase-related family protein	AT3G22060	Signaling	8.67	9E-06
receptor like protein 20	AT2G25440	Signaling	8.57	0.00586
cysteine-rich receptor-like protein kinase 20	AT4G23280	Signaling	8.10	1.2E-06
cysteine-rich receptor-like protein kinase 36	AT4G04490	Signaling	7.80	4.3E-06
Leucine-rich repeat protein kinase family protein	AT1G51850	Signaling	7.77	1.1E-05
Leucine-rich repeat transmembrane protein kinase	AT1G56120	Signaling	7.43	0.00012
leucine-rich repeat protein kinase-like protein	AT1G51790	Signaling	7.41	2.7E-08
G-type lectin S-receptor-like serine/threonine-protein kinase	AT1G61480	Signaling	7.28	2.1E-05
purple acid phosphatase 17	AT3G17790	Signaling	6.93	0.01969
cysteine-rich receptor-like protein kinase 37	AT4G04500	Signaling	6.93	2.8E-06
Protein kinase family protein	AT5G38250	Signaling	6.36	0.00024
cysteine-rich receptor-like protein kinase 13	AT4G23210	Signaling	6.29	3.7E-06
inactive leucine-rich repeat receptor-like protein kinase	AT1G66830	Signaling	5.64	0.00063
for hypothetical protein, clone: RAFL21-67-K19	AT1G29715	Signaling	4.91	0.00021
concanavalin A-like lectin protein kinase family protein	AT3G08870	Signaling	4.82	0.00209
G-type lectin S-receptor-like serine/threonine-protein kinase	AT1G67520	Signaling	4.78	0.00084
receptor-like protein kinase	AT1G72540	Signaling	4.53	6E-06
leucine-rich repeat protein kinase family protein	AT5G59680	Signaling	4.52	0.00298
Cysteine/Histidine-rich C1 domain family protein	AT2G21850	Signaling	4.41	0.00091
plasmodesmata-located protein 5	AT1G70690	Signaling	4.29	0.0001
cysteine-rich receptor-like protein kinase 38	AT4G04510	Signaling	4.23	0.00462
concanavalin A-like lectin kinase-like protein	AT3G45330	Signaling	4.01	0.00022
calcium-binding protein CML43	AT5G44460	Signaling	3.98	0.001
Purple acid phosphatases superfamily protein	AT1G13750	Signaling	3.89	0.00129
G-type lectin S-receptor-like serine/threonine-protein kinase	AT1G61550	Signaling	3.85	0.00023
Lectin-domain containing receptor kinase A4.3	AT5G01560	Signaling	3.81	0.01394
WD40 domain-containing protein	AT5G42010	Signaling	3.71	1.2E-05
SEC14 family protein	AT4G35750	Signaling	3.65	2.4E-05
cysteine-rich receptor-like protein kinase 6	AT4G23140	Signaling	3.59	0.02368
protein kinase family protein	AT4G11890	Signaling	3.47	0.0003
serine/threonine-protein kinase WNK11	AT5G55560	Signaling	3.44	0.00437
protein kinase family protein	AT4G21366	Signaling	3.44	0.00012
SEC7-like guanine nucleotide exchange family protein	AT4G35380	Signaling	3.21	0.01051
leucine-rich repeat protein kinase-like protein	AT4G39270	Signaling	3.20	0.0035
cysteine-rich receptor-like protein kinase 22	AT4G23300	Signaling	3.20	2.9E-05
Wall-associated receptor kinase	AT5G53110	Signaling	3.11	0.00132
inactive receptor kinase	AT5G53320	Signaling	3.10	0.03387
glutamate receptor 1.4	AT3G07520	Signaling	3.02	0.0094
protein MATERNAL EFFECT EMBRYO ARREST 62	AT5G45800	Signaling	3.01	0.00194
protein kinase family protein	AT3G61960	Signaling	2.97	0.0002
serine/threonine protein kinase	AT1G66880	Signaling	2.97	9.4E-05
Cam-binding protein 60-like G	AT5G26920	Signaling	2.81	0.00329
protein kinase family protein	AT5G42440	Signaling	2.68	0.00033
serine/threonine protein kinase	AT1G66920	Signaling	2.67	0.01585
atypical dual-specificity phosphatase 4	AT4G03960	Signaling	2.60	0.00012
protein kinase	AT1G48490	Signaling	2.59	2.4E-05
leucine-rich repeat protein kinase-like protein	AT5G07150	Signaling	2.55	0.00185
GTP-binding protein Obg/CgtA	AT1G07620	Signaling	2.53	0.00028
cysteine/histidine-rich C1 domain-containing protein	AT2G19650	Signaling	2.50	0.01463
receptor-like protein kinase ANXUR1	AT3G04690	Signaling	2.47	0.00108
calcineurin B-like protein 6	AT4G16350	Signaling	2.46	6E-05
cysteine-rich receptor-like protein kinase 3	AT1G70530	Signaling	2.40	0.001
uncharacterized protein	AT4G16670	Signaling	2.40	0.01824
receptor serine/threonine kinase	AT1G70250	Signaling	2.38	0.00784

calmodulin-binding receptor-like cytoplasmic kinase 1	AT5G58940	Signaling	2.32	0.01198
CBL-interacting protein kinase 5	AT5G10930	Signaling	2.32	0.01917
RabGAP/TBC domain-containing protein	AT5G54780	Signaling	2.29	0.00504
uncharacterized Rho GTPase-activating protein	AT5G61530	Signaling	2.25	6.7E-05
CBL-interacting serine/threonine-protein kinase 21	AT5G57630	Signaling	2.21	0.00615
G-type lectin S-receptor-like serine/threonine-protein kinase	AT5G35370	Signaling	2.18	0.03208
CBL-interacting serine/threonine-protein kinase 23	AT1G30270	Signaling	2.17	2.2E-05
U-box domain-containing protein kinase family protein	AT5G65500	Signaling	2.17	0.01876
mitogen-activated protein kinase 1	AT1G10210	Signaling	2.17	0.00805
wall-associated receptor kinase-like 8	AT1G16260	Signaling	2.16	0.0371
protein activator of spomin::LUC2	AT3G12890	Signaling	2.11	0.0042
protein kinase family protein	AT2G28940	Signaling	2.08	0.00154
leucine-rich repeat (LRR) family protein	AT1G13910	Signaling	2.06	0.00628
Histone H3 K4-specific methyltransferase SET7/9 family protein	AT4G17080	Signaling	2.01	0.04408
LRR receptor-like serine/threonine-protein kinase	AT1G67720	Signaling	-2.00	0.0215
protein phosphatase 2C 63	AT4G33920	Signaling	-2.02	0.00394
receptor-like protein kinase	AT2G39360	Signaling	-2.02	0.0103
protein kinase family protein	AT3G58690	Signaling	-2.04	0.00295
Protein kinase protein with tetratricopeptide repeat domain	AT1G63500	Signaling	-2.07	0.00259
LRR receptor-like serine/threonine-protein kinase	AT3G47570	Signaling	-2.07	0.0002
LRR receptor-like serine/threonine-protein kinase	AT2G16250	Signaling	-2.08	0.00973
CBL-interacting protein kinase	AT4G14580	Signaling	-2.10	0.02138
concanavalin A-like lectin kinase-like protein	AT3G45430	Signaling	-2.11	0.00202
protein phosphatase 2C 48	AT3G55050	Signaling	-2.11	0.0034
protein kinase family protein	AT5G40540	Signaling	-2.12	0.00072
mitogen-activated protein kinase 13	AT1G07880	Signaling	-2.16	0.01775
Rac-like GTP-binding protein ARAC9	AT2G44690	Signaling	-2.18	0.0027
Ras-related protein RABA6b	AT1G18200	Signaling	-2.20	1.7E-05
serine/threonine-protein kinase RLCKVII	AT1G07870	Signaling	-2.21	0.00177
Rac-like GTP-binding protein ARAC5	AT1G75840	Signaling	-2.22	0.00092
cysteine-rich receptor-like protein kinase 42	AT5G40380	Signaling	-2.23	2.4E-07
Leucine-rich repeat protein kinase family protein	AT4G23740	Signaling	-2.27	0.00722
GTP-binding protein	AT4G02790	Signaling	-2.28	0.00566
protein TIC 62	AT3G18890	Signaling	-2.29	0.02953
LysM-containing receptor-like kinase	AT2G33580	Signaling	-2.29	0.00129
PRA1 family protein B6	AT5G07110	Signaling	-2.29	0.0167
kinase interacting (KIP1-like) protein	AT1G03470	Signaling	-2.32	0.00854
LRR receptor-like serine/threonine-protein kinase FLS2	AT5G46330	Signaling	-2.33	0.01711
protein kinase family protein	AT3G57120	Signaling	-2.33	0.00706
proline-rich receptor-like protein kinase PERK15	AT1G52290	Signaling	-2.42	0.0009
RabGAP/TBC domain-containing protein	AT4G27100	Signaling	-2.43	0.00344
calmodulin-like protein MSS3	AT2G43290	Signaling	-2.45	0.00603
mitogen-activated protein kinase 3	AT3G45640	Signaling	-2.45	0.00994
leucine-rich receptor-like protein kinase	AT4G20940	Signaling	-2.45	0.00903
calcium dependent protein kinase 1	AT5G04870	Signaling	-2.47	1.9E-05
protein phosphatase 2C 47	AT3G51470	Signaling	-2.47	0.00089
protein IQ-domain 2	AT5G03040	Signaling	-2.48	1.2E-05
CBL-interacting protein kinase 2	AT5G07070	Signaling	-2.48	0.026
calmodulin 2	AT2G41110	Signaling	-2.49	0.0429
protein kinase family protein	AT1G70740	Signaling	-2.50	0.02162
copine-like protein BONZAI 1	AT5G61900	Signaling	-2.55	0.00025
purple acid phosphatase 3	AT1G14700	Signaling	-2.65	7.6E-08
CBL-interacting serine/threonine-protein kinase 15	AT5G01810	Signaling	-2.65	0.02909
remorin-like protein	AT1G45207	Signaling	-2.65	0.02348
calmodulin binding protein-like protein	AT5G62570	Signaling	-2.66	0.00094
G-type lectin S-receptor-like serine/threonine-protein kinase	AT1G61460	Signaling	-2.66	0.0215
GTP1/OBG family protein	AT5G18570	Signaling	-2.73	0.00433
serine/threonine-protein kinase	AT1G01540	Signaling	-2.75	0.00207
plasmodesmata-located protein 3	AT2G33330	Signaling	-2.76	0.01727
HAD superfamily, subfamily IIIB acid phosphatase	AT1G04040	Signaling	-2.77	0.04605
protein IQ-domain 23	AT5G62070	Signaling	-2.77	4.9E-06
leucine-rich receptor-like protein kinase family protein	AT2G25790	Signaling	-2.77	0.00021
protein kinase family protein	AT1G33770	Signaling	-2.93	0.00329
phosphatidylinositol-4-phosphate 5-kinase 6	AT3G07960	Signaling	-2.93	0.00057
leucine-rich repeat protein 1	AT5G16590	Signaling	-2.94	9.4E-05
CBL-interacting serine/threonine-protein kinase 9	AT1G01140	Signaling	-2.95	0.02512
leucine-rich receptor-like protein kinase	AT1G72180	Signaling	-2.96	0.00566
GTP-binding protein	AT1G56050	Signaling	-2.99	0.00027
cysteine-rich receptor-like protein kinase 10	AT4G23180	Signaling	-3.05	0.00014
calcium-binding endonuclease/exonuclease/phosphatase family protein	AT1G02270	Signaling	-3.10	0.00012
mechanosensitive channel of small conductance-like 6	AT1G78610	Signaling	-3.16	0.00016

protein phosphatase 2C 5	AT1G09160	Signaling	-3.17	0.00012
mitogen-activated protein kinase kinase 6	AT5G56580	Signaling	-3.23	0.00578
calcium-binding protein CML40	AT3G01830	Signaling	-3.64	0.04527
BTB/POZ domain-containing protein DOT3	AT5G10250	Signaling	-3.73	0.01941
leucine-rich repeat protein kinase-like protein	AT3G23750	Signaling	-3.74	0.00091
leucine-rich repeat (LRR) family protein	AT1G33610	Signaling	-3.78	0.00249
protein kinase-like protein	AT5G61570	Signaling	-3.79	0.0059
calcium-dependent lipid-binding domain-containing protein	AT4G34150	Signaling	-4.07	0.00038
cysteine-rich receptor-like protein kinase 29	AT4G21410	Signaling	-4.10	0.00018
protein phosphatase 2C 25	AT2G30020	Signaling	-4.16	0.04961
phosphoglucan phosphatase LSF2	AT3G10940	Signaling	-4.24	0.00076
Remorin family protein	AT2G02170	Signaling	-4.33	0.0001
protein phosphatase 2C-like protein	AT2G05050	Signaling	-4.34	0.00706
leucine-rich repeat protein kinase family protein	AT1G51805	Signaling	-4.36	0.00197
serine/threonine kinase	AT2G31010	Signaling	-4.48	0.0001
phosphoinositide phospholipase C 7	AT3G55940	Signaling	-4.69	0.00054
transducin/WD40 domain-containing protein	AT5G53500	Signaling	-4.74	0.00082
protein EXORDIUM like 2	AT5G64260	Signaling	-4.80	0.00189
protein phosphatase 2C 12	AT1G47380	Signaling	-4.85	6.2E-05
Remorin family protein	AT5G23750	Signaling	-4.97	7.2E-05
serine/threonine kinase	AT1G74330	Signaling	-5.14	4.2E-06
calcium-dependent protein kinase 32	AT3G57530	Signaling	-5.47	1.1E-05
calcium-dependent protein kinase 28	AT5G66210	Signaling	-5.77	4.3E-06
cysteine-rich receptor-like protein kinase 41	AT4G00970	Signaling	-6.31	0.00027
PTI1-like tyrosine-protein kinase 3	AT3G59350	Signaling	-6.71	2.3E-06
calcium-binding protein CML24	AT5G37770	Signaling	-9.56	0.01553
Calmodulin-binding protein	AT4G31000	Signaling	-11.03	0.00022
phosphate-responsive 1 family protein	AT4G08950	Signaling	-28.19	1.1E-07
cation/H(+) antiporter 17	AT4G23700	Transport	36.72	4.9E-07
uncharacterized protein	AT2G39530	Transport	32.65	9.7E-07
amino acid transporter 1	AT4G21120	Transport	22.99	0.00038
phosphate transporter 3;2	AT3G48850	Transport	13.84	0.00059
MATE efflux family protein	AT2G04100	Transport	9.99	2.2E-05
ADP/ATP carrier 3 protein	AT4G28390	Transport	8.59	0.0002
glucose-6-phosphate/phosphate translocator 2	AT1G61800	Transport	8.52	0.0258
ABC transporter-like protein	AT3G21080	Transport	8.14	0.00528
exocyst subunit exo70 family protein H1	AT3G55150	Transport	7.30	3.4E-05
urea-proton symporter DUR3	AT5G45380	Transport	7.19	7E-06
ALA-interacting subunit 5	AT1G79450	Transport	6.71	0.02708
cation/H(+) antiporter 16	AT1G64170	Transport	6.71	5.7E-05
calcium exchanger 7	AT5G17860	Transport	6.16	0.00265
copper transport family protein	AT5G52720	Transport	6.15	0.00632
MATE efflux family protein	AT2G04070	Transport	5.73	0.0013
cation/H(+) antiporter 2	AT1G79400	Transport	5.41	0.01921
sugar transport protein 13	AT5G26340	Transport	4.34	0.00038
inositol transporter 2	AT1G30220	Transport	4.01	0.00029
copper transport protein	AT4G05030	Transport	3.98	0.00231
proton pump interactor 2	AT3G15340	Transport	3.83	0.00175
multidrug resistance-associated protein 8	AT3G13090	Transport	3.77	0.005
ABC transporter C family member 7	AT3G13100	Transport	3.62	3.2E-06
potassium transporter 6	AT1G70300	Transport	3.32	0.00012
ABC transporter G family member 18	AT3G55110	Transport	3.23	0.00021
vesicle-associated protein 3-1	AT2G23830	Transport	3.19	0.04527
Sec14p-like phosphatidylinositol transfer family protein	AT1G75170	Transport	3.18	0.03088
aromatic and neutral transporter 1	AT3G11900	Transport	3.14	0.00487
peptide/nitrate transporter	AT5G14940	Transport	3.08	0.01018
Lysine histidine transporter 1	AT5G40780	Transport	3.05	0.00391
ABC transporter A family member 7	AT3G47780	Transport	2.90	0.03611
sec14p-like phosphatidylinositol transfer protein	AT4G36640	Transport	2.87	0.00099
TRAF-like family protein	AT5G26280	Transport	2.86	0.0103
phospholipid-transporting ATPase 12	AT1G26130	Transport	2.82	0.00018
clathrin assembly protein	AT5G35200	Transport	2.76	4.1E-05
ABC transporter C family member 4	AT2G47800	Transport	2.71	0.00088
sugar transport protein 3	AT5G61520	Transport	2.56	0.00838
phospholipid-transporting ATPase 1	AT5G04930	Transport	2.51	0.00228
cation/H(+) antiporter 20	AT3G53720	Transport	2.50	0.00357
MATE efflux family protein	AT3G21690	Transport	2.39	0.02428
MATE efflux family protein	AT3G26590	Transport	2.39	0.00288
H(+)-ATPase 2	AT4G30190	Transport	2.38	0.00375
auxin efflux transmembrane transporter MDR4	AT2G47000	Transport	2.36	0.00899
transmembrane emp24 domain-containing protein	AT1G26690	Transport	2.29	0.00023

plasma-membrane choline transporter family protein	AT3G03700	Transport	2.23	0.00099
vesicle-associated protein 4-1	AT5G54110	Transport	2.17	0.0001
zinc transporter	AT3G08650	Transport	2.16	0.04408
sodium/metabolite cotransporter BASS6	AT4G22840	Transport	2.11	0.00046
transducin/WD40 domain-containing protein	AT3G62770	Transport	2.00	0.02572
aquaporin TIP2-1	AT3G16240	Transport	-2.00	0.00359
patellin-5	AT4G09160	Transport	-2.11	0.00218
nucleotide-sugar transporter	AT2G43240	Transport	-2.21	0.00113
K+ uptake permease 9	AT4G19960	Transport	-2.22	0.02388
ABC transporter G family member 27	AT3G52310	Transport	-2.24	0.0244
nucleotide-sugar transporter family protein	AT5G04160	Transport	-2.28	0.00024
exocyst subunit exo70 family protein B1	AT5G58430	Transport	-2.48	0.00381
golgi nucleotide sugar transporter 3	AT1G76340	Transport	-2.50	5.1E-05
SNARE associated Golgi family protein	AT1G12450	Transport	-2.56	2.8E-05
envelope ADP,ATP carrier protein	AT3G51870	Transport	-2.58	0.00021
peptide/nitrate transporter	AT2G40460	Transport	-2.60	0.04016
nucleotide-sugar transporter-like protein	AT1G21070	Transport	-2.60	0.02996
amino acid permease 5	AT1G44100	Transport	-2.74	0.00085
peptide/nitrate transporter	AT5G13400	Transport	-2.75	0.02106
sugar transporter ERD6	AT1G08930	Transport	-2.77	0.02162
peptide/nitrate transporter	AT1G33440	Transport	-2.79	5E-05
Sec14p-like phosphatidylinositol transfer protein patellin-4	AT1G30690	Transport	-2.79	0.00072
peptide/nitrate transporter	AT1G22550	Transport	-2.91	0.00706
aluminum-activated, malate transporter 12	AT4G17970	Transport	-2.91	0.01419
nucleotide-sugar transporter family protein	AT4G18205	Transport	-2.93	0.01561
MATE efflux family protein	AT1G15150	Transport	-3.03	0.0057
peptide/nitrate transporter	AT1G22570	Transport	-3.07	0.02022
phosphate transporter PHO1-8	AT1G35350	Transport	-3.10	3.1E-05
hydrolase-like protein	AT5G17670	Transport	-3.11	0.01276
aquaporin TIP1-1	AT2G36830	Transport	-3.39	0.01051
sec14p-like phosphatidylinositol transfer-like protein	AT5G56160	Transport	-3.87	0.00015
autoinhibited Ca2+-ATPase 1	AT1G27770	Transport	-3.87	0.01707
Sodium/calcium exchanger family protein	AT5G17850	Transport	-4.07	0.00114
purine permease 14	AT1G19770	Transport	-4.14	0.00017
phosphate transporter PHO1-7	AT1G26730	Transport	-4.33	0.00167
sugar transporter ERD6-like 4	AT1G19450	Transport	-4.36	0.0003
EXS (ERD1/XPR1/SYG1) family protein	AT2G03240	Transport	-4.92	0.00388
exocyst subunit exo70 family protein H7	AT5G59730	Transport	-5.35	0.00965
cyclic nucleotide-gated channel 14	AT2G24610	Transport	-5.45	6.4E-05
glucosinolate transporter 1	AT3G47960	Transport	-5.79	5.7E-06
organic cation/carnitine transporter 6	AT1G16370	Transport	-11.05	0.01366
MATE efflux family protein	AT1G61890	Transport	-15.97	0.00057
MATE efflux family protein	AT5G52050	Transport	-18.91	0.0003
FAD-binding Berberine family protein	AT1G26390	Unknown	143.52	0.00019
membrane lipoprotein	AT3G18250	Unknown	66.29	0.00127
germin-like protein subfamily 1 member 8	AT4G14630	Unknown	62.13	0.00122
uncharacterized protein	AT1G13520	Unknown	54.94	0.02949
cytochrome P450, family 82, subfamily C, polypeptide 2	AT4G31970	Unknown	41.65	0.00022
FAD-binding Berberine family protein	AT1G30700	Unknown	35.33	0.00722
uncharacterized protein	AT2G39518	Unknown	33.98	0.00035
SPFH/Band 7/PHB domain-containing membrane-associated protein	AT5G25260	Unknown	30.54	3.2E-07
uncharacterized protein	AT5G44575	Unknown	27.90	9.2E-06
uncharacterized protein	AT3G55790	Unknown	24.68	1.1E-05
uncharacterized protein	AT1G36640	Unknown	20.38	2.7E-06
uncharacterized protein	AT3G19615	Unknown	20.16	0.01394
plant invertase/pectin methylesterase inhibitor domain-containing protein	AT5G46960	Unknown	18.44	2.2E-05
uncharacterized protein	AT3G13950	Unknown	17.59	0.00029
VQ motif-containing protein	AT4G20000	Unknown	17.23	4.7E-05
Adenine nucleotide alpha hydrolases-like superfamily protein	AT5G47740	Unknown	17.20	7.4E-05
Cupredoxin superfamily protein	AT2G15780	Unknown	16.93	0.00287
uncharacterized protein	AT1G53625	Unknown	16.08	0.00044
uncharacterized protein	AT5G37840	Unknown	15.69	6.4E-05
cytochrome P450, family 82, subfamily C, polypeptide 3	AT4G31950	Unknown	15.32	0.00374
pollen Ole e 1 allergen and extensin family protein	AT4G17215	Unknown	14.95	4.8E-06
uncharacterized protein	AT1G36622	Unknown	14.90	0.00029
PLAC8 family protein	AT1G68630	Unknown	14.84	8.9E-07
AAA-type ATPase family protein	AT3G28540	Unknown	14.58	0.00099
late embryogenesis abundant (LEA) hydroxyproline-rich glycoprotein	AT1G65690	Unknown	14.36	6.4E-06
uncharacterized protein	AT5G44585	Unknown	13.00	0.00014
uncharacterized protein	AT2G45360	Unknown	12.66	0.00574
beta glucosidase 46	AT1G61820	Unknown	12.63	0.03192

uncharacterized protein	AT3G26440	Unknown	12.56	1.6E-07
uncharacterized protein	AT3G15536	Unknown	10.70	0.01415
uncharacterized protein	AT1G65481	Unknown	10.43	5.9E-06
Unknown	AT5G55420	Unknown	10.33	0.00334
uncharacterized protein	AT1G13470	Unknown	10.11	0.00343
heavy-metal-associated domain-containing protein	AT5G26690	Unknown	9.42	0.00061
plant invertase/pectin methylesterase inhibitor domain-containing protein	AT1G62760	Unknown	9.33	0.00131
uncharacterized protein	AT1G65500	Unknown	9.26	0.0023
invertase/pectin methylesterase inhibitor family protein	AT5G46950	Unknown	8.84	0.00197
uncharacterized protein	AT1G15385	Unknown	8.69	0.00042
uncharacterized protein	AT4G11655	Unknown	8.67	3.7E-05
uncharacterized protein	AT5G44572	Unknown	8.26	3E-06
uncharacterized protein	AT2G21185	Unknown	8.16	5.2E-06
DUF679 domain membrane protein 1	AT3G21520	Unknown	7.635	0.03765
for hypothetical protein, clone: RAFL22-67-I17	AT5G01732	Unknown	7.41	9.9E-06
uncharacterized protein	AT3G14280	Unknown	7.02	0.0003
heat stress transcription factor B-1	AT4G36990	Unknown	6.98	3.4E-05
uncharacterized protein	AT4G28460	Unknown	6.78	0.025
O-Glycosyl hydrolases family 17 protein	AT5G64790	Unknown	6.78	0.00183
FAD-binding and BBE domain-containing protein	AT1G26380	Unknown	6.716	0.00105
cytochrome P450 71B6	AT2G24180	Unknown	6.66	8.7E-05
serine-rich protein-like protein	AT3G56500	Unknown	6.64	0.00875
pollen Ole e 1 allergen and extensin family protein	AT2G40113	Unknown	6.43	0.00504
glycosyl hydrolase family protein 17	AT5G63225	Unknown	6.12	6.9E-05
uncharacterized protein	AT5G41761	Unknown	6.026	0.00016
uncharacterized protein	AT1G53620	Unknown	6.01	2E-06
microRNA miR163 type 1 precursor, complete sequence; alternatively spliced	AT1G66725	Unknown	5.727	0.03295
cytochrome P450 71B2	AT1G13080	Unknown	5.72	8.4E-08
alpha/beta-Hydrolases superfamily protein	AT1G08310	Unknown	5.57	0.03659
uncharacterized protein	AT1G10140	Unknown	5.50	0.00677
UDP-glycosyltransferase 86A1	AT2G36970	Unknown	5.48	5.2E-05
germin-like protein 6	AT5G39100	Unknown	5.47	0.01524
cytochrome P450 monooxygenase	AT3G26220	Unknown	5.47	0.00012
polynucleotidyl transferase, ribonuclease H-like superfamily protein	AT3G12470	Unknown	5.42	0.01022
carbohydrate-binding X8 domain-containing protein	AT2G03505	Unknown	5.32	0.00154
uncharacterized protein	AT1G65845	Unknown	5.137	2.6E-05
cryptdin-related protein	AT1G51915	Unknown	5.13	0.00091
cytochrome P450, family 714, subfamily A, polypeptide 1	AT5G24910	Unknown	5.06	0.01202
uncharacterized protein	AT4G25070	Unknown	5.04	0.00011
cysteine/histidine-rich C1 domain-containing protein	AT2G44370	Unknown	4.994	0.00165
SRPBCC ligand-binding domain-containing protein	AT4G32870	Unknown	4.96	0.0022
uncharacterized protein	AT3G61198	Unknown	4.93	0.00123
uncharacterized protein	AT1G51913	Unknown	4.93	0.00429
Wound-responsive family protein	AT4G05070	Unknown	4.88	2.7E-05
uncharacterized protein	AT3G57950	Unknown	4.81	0.00253
uncharacterized protein	AT1G64405	Unknown	4.724	0.0007
uncharacerized protein	AT4G36988	Unknown	4.63	0.00273
uncharacterized protein	AT1G13480	Unknown	4.63	5.6E-05
O-acyltransferase (WSD1-like) family protein	AT5G12420	Unknown	4.454	0.00285
VQ motif-containing protein	AT4G15120	Unknown	4.34	0.00241
D-3-phosphoglycerate dehydrogenase	AT1G17745	Unknown	4.34	5.4E-05
uncharacterized protein	AT1G13550	Unknown	4.328	0.00011
late embryogenesis abundant hydroxyproline-rich glycoprotein	AT2G46150	Unknown	4.324	0.00019
uncharacterized protein	AT3G19660	Unknown	4.206	5.4E-05
copper amine oxidase family protein	AT4G12290	Unknown	4.19	9.9E-05
DnaJ domain-containing protein	AT3G08970	Unknown	4.166	0.00018
uncharacterized protein	AT4G19970	Unknown	4.16	0.00019
gunnii alcohol dehydrogenase-like protein	AT1G09480	Unknown	4.13	0.01088
uncharacterized protein	AT4G37900	Unknown	4.11	0.01129
uncharacterized protein	AT3G52480	Unknown	4.063	0.00042
uncharacterized protein	AT5G03230	Unknown	4.02	4E-05
calcium-dependent lipid-binding domain-containing protein	AT3G55470	Unknown	3.992	0.00111
uncharacterized protein	AT3G13435	Unknown	3.99	0.00681
Methyltransferase-related protein	AT5G58375	Unknown	3.99	6.4E-06
uncharacterized protein	AT1G13990	Unknown	3.97	0.00149
cytochrome P450 71B36	AT3G26320	Unknown	3.907	0.00957
uncharacterized protein	AT4G34630	Unknown	3.90	2E-05
uncharacterized protein	AT4G19370	Unknown	3.90	0.00248
uncharacterized protein	AT1G22890	Unknown	3.9	0.00193
calcium-dependent lipid-binding domain-containing protein	AT1G23140	Unknown	3.89	0.00024
core-2/I-branching beta-1,6-N-acetylglucosaminyltransferase family protein	AT1G68390	Unknown	3.87	0.00347

uncharacterized protein	AT5G50660	Unknown	3.849	0.04685
UDP-glycosyltransferase 87A2	AT2G30140	Unknown	3.847	0.01682
uncharacterized protein	AT1G49470	Unknown	3.82	0.01844
cytochrome P450 71B24	AT3G26230	Unknown	3.72	0.00098
cytochrome P450, family 81, subfamily H, polypeptide 1	AT4G37310	Unknown	3.70	7.1E-05
uncharacterized protein	AT5G12930	Unknown	3.676	0.00153
SOUL heme-binding protein	AT1G78460	Unknown	3.627	0.00809
uncharacterized protein	AT3G15760	Unknown	3.60	0.00037
uncharacterized protein	AT1G13530	Unknown	3.55	0.00077
phosphorylase superfamily protein	AT4G28940	Unknown	3.54	0.00089
Myosin heavy chain-related protein	AT4G40020	Unknown	3.528	0.00056
uncharacterized protein	AT1G16500	Unknown	3.525	0.00093
uncharacterized protein	AT2G17710	Unknown	3.516	0.03948
Mo25 family protein	AT2G03410	Unknown	3.49	0.0064
uncharacterized protein	AT5G02020	Unknown	3.44	0.02352
polyadenylate-binding protein-interacting protein 2	AT4G14270	Unknown	3.439	1.2E-05
uncharacterized protein	AT1G67920	Unknown	3.438	0.00965
uncharacterized protein	AT5G22270	Unknown	3.43	0.00241
pyridoxal phosphate phosphatase-related protein	AT1G17710	Unknown	3.43	0.0268
cytochrome P450 89A2	AT1G64900	Unknown	3.41	0.0001
uncharacterized protein	AT5G40720	Unknown	3.41	0.0425
Endosomal targeting BRO1-like domain-containing protein	AT1G13310	Unknown	3.40	0.00091
TRAF-like family protein	AT3G46190	Unknown	3.40	7E-05
uncharacterized protein	AT3G04700	Unknown	3.316	0.00026
uncharacterized protein	AT1G13360	Unknown	3.315	0.00018
cysteine/histidine-rich C1 domain-containing protein	AT2G27660	Unknown	3.311	0.00578
uncharacterized protein	AT1G71910	Unknown	3.306	0.00685
Exostosin family protein	AT5G25820	Unknown	3.30	0.00347
NAD(P)-binding Rossmann-fold superfamily protein	AT3G01980	Unknown	3.28	0.03264
cytochrome P450, family 704, subfamily A, polypeptide 2	AT2G45510	Unknown	3.271	0.00013
cytochrome P450, family 705, subfamily A, polypeptide 33	AT3G20960	Unknown	3.235	0.01646
uncharacterized protein	AT4G26990	Unknown	3.233	0.00825
Ninja-family protein AFP3	AT3G29575	Unknown	3.22	0.01227
formin-like protein 16	AT5G07770	Unknown	3.17	0.00155
RNI-like superfamily protein	AT5G45500	Unknown	3.152	0.00035
uncharacterized protein	AT1G15790	Unknown	3.144	0.00118
cytochrome P450 71B26	AT3G26290	Unknown	3.137	0.00153
uncharacterized protein	AT3G21710	Unknown	3.122	0.01174
uncharacterized protein	AT3G29240	Unknown	3.09	0.0394
uncharacterized protein	AT3G15534	Unknown	3.07	0.01194
late embryogenesis abundant hydroxyproline-rich glycoprotein	AT4G23610	Unknown	3.056	0.01178
uncharacterized protein	AT4G30230	Unknown	3.05	0.01043
uncharacterized protein	AT1G71970	Unknown	3.045	0.00278
uncharacterized protein	AT1G07090	Unknown	3.033	0.00466
uncharacterized protein	AT1G17744	Unknown	3.032	0.04131
uncharacterized protein	AT2G28570	Unknown	3.022	0.004
late embryogenesis abundant hydroxyproline-rich glycoprotein	AT4G35170	Unknown	3.01	1.1E-06
HIPL2 protein	AT5G62630	Unknown	3.001	0.0008
uncharacterized protein	AT3G03870	Unknown	2.991	0.00205
uncharacterized protein	AT4G23885	Unknown	2.987	1.2E-06
PB1_UP2 domain-containing protein	AT3G26510	Unknown	2.957	0.0016
ribosomal protein L18ae family protein	AT4G26060	Unknown	2.941	0.00014
uncharacterized protein	AT2G31160	Unknown	2.926	0.005
cytochrome P450, family 87, subfamily A, polypeptide 6	AT2G12190	Unknown	2.92	0.00875
uncharacterized protein	AT1G70420	Unknown	2.91	0.0071
uncharacterized protein	AT1G52855	Unknown	2.905	0.00205
Trm112p-like protein	AT1G78190	Unknown	2.903	0.03539
lysophospholipase 1-like protein	AT2G39410	Unknown	2.89	0.00105
cytochrome P450, family 89, subfamily A, polypeptide 5	AT1G64950	Unknown	2.86	0.0032
TRAM, LAG1 and CLN8 (TLC) lipid-sensing domain containing protein	AT3G27270	Unknown	2.86	0.0031
uncharacterized protein	AT1G10410	Unknown	2.85	0.00015
cytochrome P450 71A22	AT3G48310	Unknown	2.833	0.00105
aldehyde dehydrogenase 2C4	AT3G24503	Unknown	2.818	0.00026
Flavin-binding monooxygenase family protein	AT1G12160	Unknown	2.81	0.02604
uncharacterized protein	AT1G21050	Unknown	2.797	8.1E-05
uncharacterized protein	AT2G10560	Unknown	2.784	8.4E-05
calcium-dependent lipid-binding domain-containing protein	AT2G01540	Unknown	2.759	0.00981
protein DJ-1-like A	AT3G14990	Unknown	2.733	0.0101
uncharacterized protein	AT1G68650	Unknown	2.733	0.00408
Exostosin family protein	AT5G11610	Unknown	2.705	0.00219
uncharacterized protein	AT5G01610	Unknown	2.692	0.00025

uncharacterized protein	AT5G47580	Unknown	2.69	0.00015
HXXXD-type acyl-transferase-like protein	AT4G15400	Unknown	2.689	0.03168
leucine-rich repeat-containing protein	AT1G15740	Unknown	2.686	0.00131
uncharacterized protein	AT4G39610	Unknown	2.658	0.00466
uncharacterized protein	AT1G10690	Unknown	2.653	0.00735
cyclic phosphodiesterase	AT4G18930	Unknown	2.647	0.00296
uncharacterized protein	AT3G61280	Unknown	2.638	0.02608
carboxyesterase 20	AT5G62180	Unknown	2.631	0.00259
uncharacterized protein	AT1G70160	Unknown	2.595	9.4E-05
uncharacterized protein	AT1G24145	Unknown	2.583	0.01807
heme oxygenase-like, multi-helical protein	AT3G16990	Unknown	2.573	1.6E-05
Agenet domain-containing protein	AT5G52070	Unknown	2.571	0.00172
Dihydrolipoamide succinyltransferase	AT4G26910	Unknown	2.57	0.005
ARM repeat superfamily protein	AT3G03440	Unknown	2.538	0.00195
uncharacterized protein	AT1G08180	Unknown	2.526	0.00019
Flotillin-like protein 1	AT5G25250	Unknown	2.517	0.04843
chaperone protein dnaJ 20	AT4G13830	Unknown	2.516	0.001
uncharacterized protein	AT4G33980	Unknown	2.506	0.0306
pseudogene of disease resistance protein	AT3G03855	Unknown	2.5	0.00137
germin-like protein subfamily 2 member 5	AT5G26700	Unknown	2.479	0.00014
uncharacterized protein	AT3G13432	Unknown	2.467	0.00146
uncharacterized protein	AT2G27830	Unknown	2.39	0.02388
uncharacterized protein	AT2G15695	Unknown	2.391	0.00064
uncharacterized protein	AT1G12320	Unknown	2.385	0.00895
uncharacterized protein	AT5G44574	Unknown	2.381	0.00066
ARM repeat superfamily protein	AT3G51980	Unknown	2.356	0.00246
tc|Rep: Adagio protein 3 - Arabidopsis thaliana (Mouse-ear cress), partial (10%)	AT5G23410	Unknown	2.349	0.03901
Rossmann-fold NAD(P)-binding domain-containing protein	AT4G09750	Unknown	2.349	0.02235
uncharacterized protein	AT1G29240	Unknown	2.347	0.00362
ACT domain-containing protein	AT2G39570	Unknown	2.341	0.00545
alpha/beta-Hydrolases superfamily protein	AT3G51000	Unknown	2.34	0.00062
SART-1 family protein	AT3G14700	Unknown	2.321	0.00193
uncharacterized protein	AT4G11860	Unknown	2.309	0.0071
uncharacterized protein	AT3G24927	Unknown	2.297	0.00357
cytochrome P450 71B20	AT3G26180	Unknown	2.295	0.03773
uncharacterized protein	AT3G22240	Unknown	2.294	0.03407
uncharacterized protein	AT3G48640	Unknown	2.269	0.00083
TRAM, LAG1 and CLN8 (TLC) lipid-sensing domain containing protein	AT4G19645	Unknown	2.268	0.03698
uncharacterized protein	AT1G67850	Unknown	2.256	0.0013
uncharacterized protein	AT3G22235	Unknown	2.255	0.03734
uncharacterized protein	AT3G27880	Unknown	2.254	0.00903
alpha/beta-Hydrolases superfamily protein	AT1G78210	Unknown	2.253	0.00039
uncharacterized protein	AT3G10250	Unknown	2.252	0.00013
prolyl-4 hydroxylase 2	AT3G06300	Unknown	2.247	0.00382
uncharacterized protein	AT1G65985	Unknown	2.242	0.00215
uncharacterized protein	AT4G11350	Unknown	2.238	0.01341
CCT motif family protein	AT4G27900	Unknown	2.238	0.02194
uncharacterized protein	AT1G03290	Unknown	2.237	0.00035
clone 7553 sequence	AT1G67328	Unknown	2.232	0.00291
uncharacterized protein	AT5G57910	Unknown	2.225	0.03861
uncharacterized protein	AT1G01730	Unknown	2.224	0.00025
cytochrome P450, family 81, subfamily D, polypeptide 5	AT4G37320	Unknown	2.22	3.5E-05
cytochrome P450, family 76, subfamily C, polypeptide 5	AT1G33730	Unknown	2.211	0.00193
reticulon-like protein B9	AT3G18260	Unknown	2.209	0.02612
Yippee family zinc-binding protein	AT2G40110	Unknown	2.207	0.00821
Phenazine biosynthesis PhzC/PhzF protein	AT1G03210	Unknown	2.194	0.02444
cytochrome P450 71B4	AT3G26280	Unknown	2.176	0.0014
uncharacterized protein	AT1G75810	Unknown	2.175	0.04487
amidase family protein	AT5G07360	Unknown	2.172	0.02452
kinectin-related protein	AT5G66250	Unknown	2.171	0.00661
camphor resistance CrcB-like protein	AT2G41705	Unknown	2.162	0.00036
for hypothetical protein, clone: RAFL21-14-O08	AT3G26165	Unknown	2.159	0.00159
tetratricopeptide repeat-containing protein-like protein	AT1G07280	Unknown	2.158	0.03208
uncharacterized protein	AT2G40390	Unknown	2.157	0.00694
gb|AYBKB64TR pooled cDNA populations Arabidopsis thaliana cDNA, sequence	AT1G34315	Unknown	2.155	0.02736
uncharacterized protein	AT5G10946	Unknown	2.155	0.00079
VQ motif-containing protein	AT4G39720	Unknown	2.15	0.0011
uncharacterized protein	AT5G53030	Unknown	2.135	0.00074
uncharacterized protein	AT4G14620	Unknown	2.121	0.018
uncharacterized protein	AT1G65720	Unknown	2.114	0.0152
CFIM-25-like protein	AT4G29820	Unknown	2.111	0.01459

germin-like protein subfamily 1 member 18	AT5G39160	Unknown	2.105	0.00204
for hypothetical protein, partial cds, clone: RAFL14-17-D06	AT2G31585	Unknown	2.103	0.00125
uncharacterized protein	AT4G09830	Unknown	2.098	0.00516
uncharacterized protein	AT2G03310	Unknown	2.096	0.03188
cytochrome P450 71B11	AT5G25120	Unknown	2.093	0.01642
Mediator complex, subunit Med10	AT1G26665	Unknown	2.066	0.00557
uncharacterized protein	AT5G19860	Unknown	2.048	0.00772
tudor-like RNA-binding protein	AT4G32440	Unknown	2.045	4.5E-05
cytochrome c oxidase 19-1	AT1G66590	Unknown	2.044	0.00065
Aluminum activated malate transporter family protein	AT1G25480	Unknown	2.041	0.00026
uncharacterized protein	AT1G62840	Unknown	2.041	0.03762
uncharacterized protein	AT1G53035	Unknown	2.039	0.0117
uncharacterized protein	AT5G59400	Unknown	2.023	0.03056
wound-responsive protein	AT1G19660	Unknown	2.02	0.00328
uncharacterized protein	AT5G45470	Unknown	2.02	0.01723
uncharacterized protein	AT1G22250	Unknown	2.005	0.00479
uncharacterized protein	AT1G13540	Unknown	2.002	0.00315
uncharacterized protein	AT1G14770	Unknown	-2.00	0.02728
uncharacterized protein	AT5G66740	Unknown	-2.00	0.00216
transmembrane protein 97	AT1G05210	Unknown	-2.00	0.00379
uncharacterized protein	AT4G36105	Unknown	-2.021	0.0011
uncharacterized protein	AT5G63040	Unknown	-2.024	0.00365
uncharacterized protein	AT4G28590	Unknown	-2.028	0.01771
Restriction endonuclease, type II-like protein	AT1G67660	Unknown	-2.029	0.03164
uncharacterized protein	AT3G50340	Unknown	-2.03	0.00165
dynamin-like protein ARC5	AT3G19720	Unknown	-2.037	0.01096
Patched family protein	AT1G42470	Unknown	-2.045	0.00862
Vps4 regulator of MVB pathway	AT2G19710	Unknown	-2.047	0.01852
uncharacterized protein	AT1G65295	Unknown	-2.055	0.00512
uncharacterized protein	AT4G39900	Unknown	-2.061	0.03204
uncharacterized protein	AT2G34670	Unknown	-2.073	0.01537
P-loop containing nucleoside triphosphate hydrolase family protein	AT4G34420	Unknown	-2.074	0.00036
thylakoid lumenal protein-17.4	AT5G53490	Unknown	-2.09	0.03423
uncharacterized protein	AT3G51220	Unknown	-2.092	0.01272
tetrapyrrole (corrin/porphyrin)methylase	AT1G45110	Unknown	-2.097	0.03662
haloacid dehalogenase-like hydrolase domain-containing protein	AT3G58830	Unknown	-2.098	0.00326
uncharacterized protein	AT5G41100	Unknown	-2.106	0.00458
uncharacterized protein	AT2G25250	Unknown	-2.109	0.01807
Uroporphyrinogen decarboxylase	AT2G40490	Unknown	-2.114	0.00097
cold regulated 413 plasma membrane 1	AT2G15970	Unknown	-2.123	0.01598
Rhodanese/Cell cycle control phosphatase superfamily protein	AT3G25480	Unknown	-2.125	0.00574
uncharacterized protein	AT5G57000	Unknown	-2.126	0.00308
DNAJ heat shock N-terminal domain-containing protein	AT2G17880	Unknown	-2.127	0.03208
Unknown	AT3G53010	Unknown	-2.134	0.0002
uncharacterized protein	AT2G17300	Unknown	-2.136	0.02259
uncharacterized protein	AT2G30990	Unknown	-2.139	0.013
uncharacterized protein	AT2G44640	Unknown	-2.157	0.00379
uncharacterized protein	AT5G19260	Unknown	-2.165	0.04843
Uroporphyrinogen-III synthase	AT2G26540	Unknown	-2.172	0.02885
mesophyll-cell RNAi library line 7-like protein	AT2G31840	Unknown	-2.175	0.01358
uncharacterized protein	AT3G17330	Unknown	-2.18	0.0256
beta glucosidase 40	AT1G26560	Unknown	-2.18	0.0276
transacting siRNA generating locus	AT1G63130	Unknown	-2.186	0.0258
fringe-related protein	AT4G00300	Unknown	-2.19	0.02712
uncharacterized protein	AT5G13100	Unknown	-2.192	0.00315
uncharacterized protein	AT3G29280	Unknown	-2.197	0.03682
uncharacterized protein	AT1G57680	Unknown	-2.206	0.03607
tc|Rep: F22C12.9 - Arabidopsis thaliana (Mouse-ear cress), partial (56%)	AT1G64150	Unknown	-2.218	0.04329
uncharacterized protein	AT1G03730	Unknown	-2.227	0.00011
chromosome transmission fidelity protein 8 domain-containing protein	AT5G52220	Unknown	-2.234	0.01182
uncharacterized protein	AT5G22340	Unknown	-2.259	0.02178
uncharacterized protein	AT3G47510	Unknown	-2.271	0.02307
uncharacterized protein	AT2G35470	Unknown	-2.273	0.00116
uncharacterized protein	AT5G03670	Unknown	-2.274	0.02488
armadillo/beta-catenin-like repeat-containing protein	AT5G50900	Unknown	-2.274	0.0069
uncharacterized protein	AT1G69760	Unknown	-2.278	2.1E-05
protein LUTEIN DEFICIENT 5	AT1G31800	Unknown	-2.28	0.00891
uncharacterized protein	AT2G42110	Unknown	-2.28	0.02877
uncharacterized protein	AT1G49840	Unknown	-2.288	0.00578
branched-chain-amino-acid aminotransferase-like protein 1	AT3G05190	Unknown	-2.296	0.00285
thylakoid lumenal protein 17.9	AT4G24930	Unknown	-2.308	0.01488

uncharacterized protein	AT4G22830	Unknown	-2.323	0.00046
beta carbonic anhydrase 4	AT1G70410	Unknown	-2.323	0.00036
uncharacterized protein	AT4G15790	Unknown	-2.371	0.01739
uncharacterized protein	AT3G15480	Unknown	-2.374	2E-05
uncharacterized protein	AT2G43340	Unknown	-2.382	0.0238
Rossmann-fold NAD(P)-binding domain-containing protein	AT4G20760	Unknown	-2.384	0.04447
alpha-L-fucosidase 2	AT4G34260	Unknown	-2.403	0.00046
uncharacterized protein	AT3G08030	Unknown	-2.403	0.02142
uncharacterized protein	AT5G26770	Unknown	-2.405	0.00784
uncharacterized protein	AT5G42110	Unknown	-2.419	0.00221
uncharacterized protein	AT3G28760	Unknown	-2.424	0.01288
uncharacterized protein	AT1G68430	Unknown	-2.426	0.01415
uncharacterized protein	AT5G48470	Unknown	-2.446	0.00582
D-mannose binding lectin protein with Apple-like carbohydrate-binding domain	AT1G78820	Unknown	-2.447	0.0004
uncharacterized protein	AT3G50120	Unknown	-2.455	0.00282
cytochrome c biogenesis protein CCS1	AT1G49380	Unknown	-2.469	0.01921
alpha/beta-hydrolase-like protein	AT1G29840	Unknown	-2.478	1.8E-05
uncharacterized protein	AT5G43950	Unknown	-2.483	0.01063
uncharacterized protein	AT2G33400	Unknown	-2.489	0.04883
P-loop containing nucleoside triphosphate hydrolases superfamily protein	AT5G52882	Unknown	-2.495	0.0429
GTP-binding protein Era	AT5G66470	Unknown	-2.496	0.00755
uncharacterized protein	AT4G24175	Unknown	-2.504	0.00013
cytokine-induced anti-apoptosis inhibitor 1, Fe-S biogenesis	AT5G18400	Unknown	-2.523	0.04645
NHL domain-containing protein	AT1G70280	Unknown	-2.534	0.02752
uncharacterized protein	AT5G43880	Unknown	-2.567	0.00038
uncharacterized protein	AT2G29180	Unknown	-2.571	0.0425
tetratricopeptide repeat-containing protein	AT3G05625	Unknown	-2.576	0.00586
tropinone reductase-like protein	AT2G29300	Unknown	-2.583	0.00187
uncharacterized protein	AT1G75860	Unknown	-2.59	0.00034
tetratricopeptide repeat domain-containing protein	AT3G47080	Unknown	-2.601	5.5E-05
uncharacterized protein	AT5G44005	Unknown	-2.605	0.03108
heptahelical transmembrane protein1	AT5G20270	Unknown	-2.625	0.04527
uncharacterized protein	AT5G22390	Unknown	-2.628	0.00764
alpha/beta-Hydrolases superfamily protein	AT1G13820	Unknown	-2.631	0.00029
uncharacterized protein	AT5G66675	Unknown	-2.639	0.00011
conserved peptide upstream open reading frame 32	AT1G73602	Unknown	-2.641	0.02524
CASP-like protein	AT2G35760	Unknown	-2.642	0.015
O-Glycosyl hydrolases family 17 protein	AT5G55180	Unknown	-2.643	0.00353
uncharacterized protein	AT1G16170	Unknown	-2.645	0.00483
methyltransferase	AT5G01710	Unknown	-2.648	0.00053
uncharacterized protein	AT1G10522	Unknown	-2.668	0.00907
uncharacterized protein	AT3G06150	Unknown	-2.687	0.00731
uncharacterized protein	AT4G21570	Unknown	-2.692	0.00998
PLAC8 family protein	AT1G11380	Unknown	-2.695	0.00018
protein PHLOEM protein 2-LIKE A2	AT4G19850	Unknown	-2.698	0.0312
uncharacterized protein	AT4G30996	Unknown	-2.707	1.1E-05
alpha/beta-Hydrolases superfamily protein	AT5G09430	Unknown	-2.713	0.00207
hydrolase, alpha/beta fold family protein	AT4G12830	Unknown	-2.717	0.0417
O-Glycosyl hydrolases family 17 protein	AT3G07320	Unknown	-2.744	0.01083
uncharacterized protein	AT5G46220	Unknown	-2.752	0.00359
uncharacterized protein	AT5G37360	Unknown	-2.754	7.5E-05
uncharacterized protein	AT3G13275	Unknown	-2.756	0.00021
LORELEI-LIKE-GPI-ANCHORED PROTEIN 1	AT5G56170	Unknown	-2.761	0.00479
uncharacterized protein	AT5G64850	Unknown	-2.767	1.1E-05
uncharacterized protein	AT1G01570	Unknown	-2.768	0.00045
uncharacterized protein	AT3G07460	Unknown	-2.769	6.9E-05
Stress responsive alpha-beta barrel domain protein	AT2G32500	Unknown	-2.783	0.01787
hydrolase, alpha/beta fold family protein	AT5G02970	Unknown	-2.811	0.00044
thylakoid lumenal protein 2	AT5G52970	Unknown	-2.815	0.00063
uncharacterized protein	AT4G29400	Unknown	-2.825	0.02584
protein MID1-complementing activity 2	AT2G17780	Unknown	-2.855	0.01719
uncharacterized protein	AT3G56810	Unknown	-2.867	0.04764
uncharacterized protein	AT1G02380	Unknown	-2.881	0.00574
uncharacterized protein	AT5G61412	Unknown	-2.891	0.00102
uncharacterized protein	AT2G28140	Unknown	-2.901	0.0203
uncharacterized protein	AT5G11070	Unknown	-2.921	0.00099
magnesium chelatase subunit I2	AT5G45930	Unknown	-2.936	0.00044
uncharacterized protein	AT3G06435	Unknown	-2.945	0.02243
uncharacterized protein	AT2G36410	Unknown	-2.954	8.1E-05
polyketide cyclase / dehydrase and lipid transport protein	AT4G01883	Unknown	-2.955	0.00355
myb domain protein 73	AT4G37260	Unknown	-2.958	0.00296

uncharacterized protein	AT5G37550	Unknown	-2.959	0.04804
uncharacterized protein	AT1G05540	Unknown	-2.966	0.00368
uncharacterized protein	AT4G09640	Unknown	-2.97	1.8E-05
early nodulin-like protein 2	AT4G27520	Unknown	-2.984	0.01695
uncharacterized protein	AT2G20724	Unknown	-2.992	0.00103
uncharacterized protein	AT5G27290	Unknown	-3.008	0.0238
uncharacterized protein	AT1G18740	Unknown	-3.03	0.00491
uncharacterized protein	AT5G62960	Unknown	-3.049	0.00019
uncharacterized protein	AT5G03390	Unknown	-3.053	0.00138
uncharacterized protein	AT3G07380	Unknown	-3.058	3.1E-05
uncharacterized protein	AT5G66800	Unknown	-3.067	0.01002
Rubber elongation factor protein	AT2G47780	Unknown	-3.071	0.00231
dihydrodipicolinate reductase 1	AT2G44040	Unknown	-3.106	0.00019
uncharacterized protein	AT4G17240	Unknown	-3.108	0.02086
uncharacterized protein	AT4G27350	Unknown	-3.113	0.00316
uncharacterized protein	AT2G39650	Unknown	-3.155	0.00117
uncharacterized protein	AT1G10020	Unknown	-3.16	0.00033
hydrolase	AT1G52510	Unknown	-3.178	0.0022
Unknown	AT5G01542	Unknown	-3.19	4.4E-05
uncharacterized protein	AT3G60990	Unknown	-3.238	3E-05
major facilitator protein	AT2G16990	Unknown	-3.259	0.00225
uncharacterized protein	AT3G01960	Unknown	-3.263	0.00314
methyltransferase	AT3G28460	Unknown	-3.301	0.0018
uncharacterized protein	AT1G18060	Unknown	-3.305	0.00524
Rossmann-fold NAD(P)-binding domain-containing protein	AT2G37540	Unknown	-3.317	0.00057
uncharacterized protein	AT4G20170	Unknown	-3.323	0.00024
formin-like protein 7	AT1G59910	Unknown	-3.326	4.8E-06
uncharacterized protein	AT4G18070	Unknown	-3.368	5.4E-05
uncharacterized protein	AT3G01430	Unknown	-3.382	0.00285
uncharacterized protein	AT1G27030	Unknown	-3.387	2.8E-05
uncharacterized protein	AT3G07470	Unknown	-3.443	3.7E-05
uncharacterized protein	AT5G44010	Unknown	-3.462	0.00017
uncharacterized protein	AT5G67370	Unknown	-3.479	0.02046
uncharacterized protein	AT1G69890	Unknown	-3.512	0.00063
uncharacterized protein	AT2G24330	Unknown	-3.516	4.8E-05
armadillo/beta-catenin-like repeats-containing protein	AT2G05810	Unknown	-3.532	0.01993
uncharacterized protein	AT1G29980	Unknown	-3.541	0.0062
FAD-binding and BBE domain-containing protein	AT4G20820	Unknown	-3.553	5.1E-05
early nodulin-like protein 18	AT1G08500	Unknown	-3.569	0.01006
transducin/WD40 repeat-like superfamily protein	AT1G78070	Unknown	-3.616	0.00011
alpha carbonic anhydrase 1	AT3G52720	Unknown	-3.617	5.9E-05
cysteine/histidine-rich C1 domain-containing protein	AT4G02540	Unknown	-3.626	8.5E-05
uncharacterized protein	AT1G09575	Unknown	-3.672	0.00768
uncharacterized protein	AT1G69430	Unknown	-3.702	0.0002
uncharacterized protein	AT2G35658	Unknown	-3.703	0.03607
COBRA-like protein 8	AT3G16860	Unknown	-3.725	0.04804
uncharacterized protein	AT3G54000	Unknown	-3.777	0.00907
CASP-like protein	AT2G38480	Unknown	-3.781	0.00303
Galactose-binding protein	AT1G22882	Unknown	-3.828	0.00174
cysteine/histidine-rich C1 domain-containing protein	AT2G23100	Unknown	-3.855	1.4E-06
uncharacterized protein	AT3G49720	Unknown	-3.942	5.8E-06
pollen Ole e 1 allergen and extensin family protein	AT3G26960	Unknown	-3.958	0.00204
uncharacterized protein	AT3G06890	Unknown	-3.964	0.00607
cytochrome P450, family 96, subfamily A, polypeptide 4	AT5G52320	Unknown	-4.059	0.04091
Unknown	AT1G74330	Unknown	-4.161	0.00047
Exostosin family protein	AT4G32790	Unknown	-4.266	1.8E-06
ATP sulfurylase	AT4G14680	Unknown	-4.307	0.02548
uncharacterized protein	AT1G79245	Unknown	-4.42	0.00027
uncharacterized protein	AT5G10750	Unknown	-4.456	0.00034
uncharacterized protein	AT2G12400	Unknown	-4.498	0.00134
uncharacterized protein	AT5G25240	Unknown	-4.677	0.01345
regulator of Vps4 activity protein	AT4G32350	Unknown	-4.712	0.0252
uncharacterized protein	AT5G36920	Unknown	-4.742	0.01313
uncharacterized protein	AT1G01130	Unknown	-4.789	0.00045
uncharacterized protein	AT5G57770	Unknown	-4.837	7.3E-05
uncharacterized protein	AT4G39840	Unknown	-4.874	0.00183
O-fucosyltransferase family protein	AT2G44500	Unknown	-4.884	0.00113
uncharacterized protein	AT1G61667	Unknown	-4.914	0.00105
uncharacterized protein	AT1G80120	Unknown	-4.976	0.03372
Actin cross-linking protein	AT1G69900	Unknown	-5.063	0.00024
beta-glucosidase 47	AT4G21760	Unknown	-5.083	0.00119

for hypothetical protein, clone: RAFL21-49-A06	AT5G36002	Unknown	-5.138	0.00092
major facilitator protein	AT4G27720	Unknown	-5.149	9.3E-06
uncharacterized protein	AT5G17350	Unknown	-5.154	0.00953
uncharacterized protein	AT4G02170	Unknown	-5.401	0.00112
uncharacterized protein	AT3G11420	Unknown	-5.429	2.2E-06
C1 domain-containing protein	AT5G03360	Unknown	-5.559	0.0006
uncharacterized protein	AT2G27402	Unknown	-5.616	4.8E-05
Regulator of Vps4 activity in the MVB pathway protein	AT4G35730	Unknown	-5.642	0.00054
uncharacterized protein	AT4G25830	Unknown	-5.701	2.9E-06
uncharacterized protein	AT1G22470	Unknown	-5.742	0.00566
NADPH--cytochrome P450 reductase 2	AT4G30210	Unknown	-5.838	5.7E-05
uncharacterized protein	AT3G06070	Unknown	-6.014	4.8E-05
copper amine oxidase	AT1G31690	Unknown	-6.17	0.00251
uncharacterized protein	AT2G36145	Unknown	-6.19	0.00049
uncharacterized protein	AT1G06475	Unknown	-6.34	4.4E-07
uncharacterized protein	AT2G32200	Unknown	-6.344	0.01618
Heavy metal transport/detoxification superfamily protein	AT5G05365	Unknown	-6.452	6.2E-06
uncharacterized protein	AT1G19380	Unknown	-6.47	0.00084
VQ motif-containing protein	AT2G22880	Unknown	-6.596	0.00462
uncharacterized protein	AT3G61920	Unknown	-6.601	0.00011
uncharacterized protein	AT1G56660	Unknown	-7.12	0.00504
uncharacterized protein	AT2G01300	Unknown	-7.153	0.02396
O-fucosyltransferase family protein	AT3G05320	Unknown	-7.554	2.1E-05
uncharacterized protein	AT3G19680	Unknown	-7.58	3.9E-09
uncharacterized protein	AT1G74450	Unknown	-7.628	0.00407
uncharacterized protein	AT3G14870	Unknown	-8.062	9.1E-06
uncharacterized protein	AT2G20835	Unknown	-8.29	0.00487
uncharacterized protein	AT2G34510	Unknown	-8.334	4.5E-06
Hs1pro-1 protein	AT3G55840	Unknown	-8.337	0.00395
uncharacterized protein	AT2G44230	Unknown	-8.493	0.00097
hydroxyproline-rich glycoprotein family protein	AT1G72790	Unknown	-9.147	3.8E-05
uncharacterized protein	AT5G56980	Unknown	-9.625	0.04408
uncharacterized protein	AT2G23690	Unknown	-9.771	2.2E-05
uncharacterized protein	AT5G38700	Unknown	-9.791	0.02672
uncharacterized protein	AT5G28610	Unknown	-11.36	0.00072
uncharacterized protein	AT4G37240	Unknown	-12.06	4.6E-06
Late embryogenesis abundant protein, group 6	AT2G23120	Unknown	-12.09	3.4E-08
uncharacterized protein	AT1G16850	Unknown	-12.31	0.00054
uncharacterized protein	AT5G03120	Unknown	-12.7	0.00022
late embryogenesis abundant hydroxyproline-rich glycoprotein	AT2G27080	Unknown	-12.71	5.8E-05
uncharacterized protein	AT4G29780	Unknown	-13.95	7.4E-05
uncharacterized protein	AT5G57760	Unknown	-19.1	2.5E-06
uncharacterized protein	AT1G50040	Unknown	-25.76	6.7E-07
uncharacterized protein	AT2G35290	Unknown	-37.1	3.8E-06

Supplementary Table S5. Comparative response of genes induced by *P. cucumerina* infection in wild type and *MIM396* plants

Description	Description	Go category	WT_Pcc vs. WT_Mock	MIM_Pcc vs. MIM_Mock
lipid-transfer protein/seed storage	AT4G12500	Biotic stress	13.70	29.87
chitinase	AT2G43620	Biotic stress	8.62	14.55
disease resistance protein RMG1	AT4G11170	Biotic stress	23.26	31.45
lipid-transfer protein/seed storage	AT4G12490	Biotic stress	1.00	85.79
beta-1,3-glucanase	AT4G16260	Biotic stress	1.00	15.07
lipid transfer protein (LTP)	AT3G22600	Biotic stress	43.71	75.44
carbonic anhydrase 2	AT2G28210	Biotic stress	1.00	8.15
PR-6 proteinase inhibitor family protein	AT2G38870	Biotic stress	6.84	9.11
NB-ARC domain-containing disease resistance protein	AT3G14470	Biotic stress	-	4.94
beta-1,3-glucanase 3	AT3G57240	Biotic stress	-	7.22
Tautomerase/MIF superfamily protein	AT3G51660	Biotic stress	-	5.71
receptor like protein 21	AT2G25470	Biotic stress	-	5.54
hydroxyproline-rich glycoprotein family protein	AT1G70985	Biotic stress	-	-6.02
flavin-dependent monooxygenase 1 (FMO1)	AT1G19250	Biotic stress	209.47	178.82
GDSL lipase 1	AT5G40990	Biotic stress	51.98	59.93
AGD2-like defense response protein 1	AT2G13810	Biotic stress	44.07	50.46
cytochrome P450 71A12	AT2G30750	Biotic stress	37.19	73.58
chitinase class 4-like protein	AT2G43570	Biotic stress	32.95	27.95
PR-6 proteinase inhibitor family protein	AT5G43570	Biotic stress	32.39	27.73
protein AIG1 (AVRRPT2-Induced Gene 1)	AT1G33960	Biotic stress	22.05	23.93
cytochrome P450, family 71, subfamily A, polypeptide 13	AT2G30770	Biotic stress	17.03	30.17
chitinase A	AT5G24090	Biotic stress	16.76	19.00
Chitinase family protein	AT2G43590	Biotic stress	16.41	12.04
cysteine/histidine-rich C1 domain-containing protein	AT2G17740	Biotic stress	15.96	30.75
uncharacterized protein	AT5G57510	Biotic stress	13.28	-
lipid-transfer protein/seed storage	AT1G36150	Biotic stress	12.86	-
BON1-associated protein 2	AT2G45760	Biotic stress	11.94	-
chitinase	AT3G47540	Biotic stress	9.18	-
uncharacterized protein	AT5G24200	Biotic stress	8.88	12.59
disease resistance protein	AT5G66890	Biotic stress	8.64	7.30
FAD-binding and BBE domain-containing protein	AT1G26420	Biotic stress	8.63	19.94
basic chitinase B	AT3G12500	Biotic stress	8.39	5.04
TIR-NBS class of disease resistance protein	AT1G72890	Biotic stress	7.72	5.40
Isochorismate synthase 1	AT1G74710	Biotic stress	6.87	9.41
Toll-Interleukin-Resistance domain-containing protein	AT5G44920	Biotic stress	6.06	-
Disease resistance protein (TIR-NBS class)	AT1G17615	Biotic stress	6.03	5.53
UDP-dependent glycosyltransferase 76B1	AT3G11340	Biotic stress	5.71	5.99
downy mildew resistance 6 protein / oxidoreductase	AT5G24530	Biotic stress	5.45	6.19
C2 calcium/lipid-binding plant phosphoribosyltransferase	AT4G00700	Biotic stress	5.36	7.29
defensin-like protein 308	AT5G46871	Biotic stress	4.91	6.53
defensin-like protein 309	AT5G46874	Biotic stress	4.45	5.41
carboxyesterase 17	AT5G16080	Biotic stress	4.41	5.13
LURP1 protein	AT2G14560	Biotic stress	4.40	6.73
uncharacterized protein	AT5G64510	Biotic stress	4.39	5.87
RPW8 domain-containing powdery mildew resistance protein	AT3G26470	Biotic stress	4.16	5.46
uncharacterized protein	AT5G45410	Biotic stress	3.91	3.36
pathogenesis-related protein 1 domain-containing protein	AT4G07820	Biotic stress	3.84	4.34
cytochrome P450 71B23	AT3G26210	Biotic stress	3.67	9.20
LRR and NB-ARC domain-containing disease resistance protein	AT3G14460	Biotic stress	3.59	4.46
receptor like protein 50	AT4G13920	Biotic stress	3.42	-
MLO-like protein 3	AT3G45290	Biotic stress	3.36	4.93
avirulence induced protein	AT1G33950	Biotic stress	3.29	3.43
phospholipase-like protein (PEARLI 4) family	AT2G16900	Biotic stress	3.27	4.05
uncharacterized protein	AT4G29110	Biotic stress	3.21	4.26
VQ motif-containing protein	AT2G41180	Biotic stress	2.95	2.91
beta glucosidase 10	AT4G27830	Biotic stress	2.95	3.31
4-coumarate--CoA ligase 1	AT1G51680	Biotic stress	2.90	3.37
alcohol dehydrogenase-like 6	AT5G24760	Biotic stress	2.77	2.57
Bax inhibitor-1 family protein	AT5G47130	Biotic stress	2.75	3.27
methyl esterase 1	AT2G23620	Biotic stress	2.69	2.56
CC-NBS-LRR class disease resistance protein	AT1G12290	Biotic stress	2.66	3.26
haloacid dehalogenase-like hydrolase domain-containing protein	AT2G41250	Biotic stress	2.65	-

cytochrome P450 98A3	AT2G40890	Biotic stress	2.61	2.71
leucine-rich repeat-containing protein	AT5G45510	Biotic stress	2.53	3.53
defensin-like protein 36	AT1G69828	Biotic stress	2.43	-
calreticulin-3	AT1G08450	Biotic stress	2.40	3.23
FAD/NAD(P)-binding oxidoreductase family protein	AT4G38540	Biotic stress	2.40	3.67
TIR-NBS-LRR class disease resistance protein	AT5G51630	Biotic stress	2.35	2.45
NB-ARC domain-containing disease resistance protein	AT4G27220	Biotic stress	2.35	-
glycine-rich family protein	AT4G27850	Biotic stress	2.23	2.02
prolyl 4-hydroxylase 5	AT2G17720	Biotic stress	2.16	2.89
TIR-NBS-LRR class disease resistance protein	AT5G45000	Biotic stress	2.14	5.49
protein PHLOEM protein 2-LIKE A8	AT5G45070	Biotic stress	2.13	-
cysteine-rich receptor-like protein kinase 18	AT4G23260	Biotic stress	1.83	2.66
TIR-NBS-LRR class disease resistance protein	AT1G63860	Biotic stress	-1.32	-2.25
defensin-like protein 302	AT3G13403	Biotic stress	-1.87	-2.29
TIR-NBS-LRR class disease resistance protein	AT1G63750	Biotic stress	-1.93	-2.48
TIR-NBS-LRR class disease resistance protein	AT5G41740	Biotic stress	-2.00	-2.27
leucine-rich repeat (LRR) family protein	AT1G33590	Biotic stress	-2.13	-
TIR-NBS-LRR class disease resistance protein	AT5G58120	Biotic stress	-2.21	-2.36
chloroplast stem-loop binding protein	AT1G09340	Biotic stress	-2.32	-
TIR-NBS class of disease resistance protein	AT1G66090	Biotic stress	-2.32	-2.25
TIR-NBS-LRR class disease resistance protein	AT1G56540	Biotic stress	-2.32	-2.44
vegetative storage protein 2	AT5G24770	Biotic stress	-2.33	-
glycine-rich protein	AT5G61660	Biotic stress	-2.33	-2.00
respiratory burst oxidase-D	AT5G47910	Biotic stress	-2.36	-
lipid-transfer protein/seed storage	AT2G27130	Biotic stress	-2.40	-2.60
glycine-rich protein	AT4G29020	Biotic stress	-2.49	-
carbonic anhydrase 1	AT3G01500	Biotic stress	-2.52	-
TIR-NBS-LRR class disease resistance protein	AT4G19520	Biotic stress	-2.57	-2.35
TIR-NBS-LRR class disease resistance protein	AT4G19530	Biotic stress	-2.74	-2.99
uncharacterized protein	AT1G61260	Biotic stress	-2.82	-2.95
TIR-NBS-LRR class disease resistance protein	AT3G44630	Biotic stress	-2.85	-2.55
PLAT-plant-stress domain-containing protein	AT2G22170	Biotic stress	-2.99	-3.25
MLO-like protein 4	AT1G11000	Biotic stress	-3.02	-3.83
glycine-rich protein	AT1G07135	Biotic stress	-3.09	-5.28
TIR-NBS-LRR class disease resistance protein	AT5G41750	Biotic stress	-3.14	-2.12
lipid-transfer protein/seed storage	AT3G43720	Biotic stress	-3.22	-2.85
Thaumatin-like protein	AT1G18250	Biotic stress	-3.28	-
Non-specific lipid-transfer protein-like protein	AT2G13820	Biotic stress	-3.32	-2.62
uncharacterized protein	AT4G24275	Biotic stress	-3.44	-4.91
uncharacterized protein	AT1G58420	Biotic stress	-3.46	-3.78
RPM1-interacting protein 4 (RIN4) family protein	AT4G35655	Biotic stress	-3.66	-3.79
Non-specific lipid-transfer protein-like protein	AT5G64080	Biotic stress	-3.74	-2.89
MLO-like protein 8	AT2G17480	Biotic stress	-4.23	-4.64
phytochelatin synthase 1	AT5G44070	Biotic stress	-4.34	-3.55
TIR-NBS-LRR class disease resistance protein	AT5G46510	Biotic stress	-4.65	-4.43
TIR class disease resistance protein	AT1G61100	Biotic stress	-4.75	-5.09
ferrochelatase 1	AT5G26030	Biotic stress	-4.96	-4.27
uncharacterized protein	AT3G57450	Biotic stress	-4.98	-6.83
lipid-transfer protein/seed storage	AT1G73550	Biotic stress	-5.01	-6.56
Toll-Interleukin-Resistance domain-containing protein	AT1G57850	Biotic stress	-5.06	-6.15
dicarboxylate carrier 2	AT4G24570	Biotic stress	-5.08	-13.73
pathogenesis-related thaumatin-like protein	AT1G19320	Biotic stress	-6.01	-
Pto-interacting 1-4	AT2G47060	Biotic stress	-6.76	-7.46
glycine-rich cell wall protein-like protein	AT4G18280	Biotic stress	-7.01	-12.04
TIR-NBS-LRR class disease resistance protein	AT5G22690	Biotic stress	-7.47	-7.33
CBS domain-containing protein	AT5G53750	Biotic stress	-10.27	-12.54
RPM1-interacting protein 4-like protein	AT2G17660	Biotic stress	-12.03	-21.94
pathogenesis-related thaumatin family protein	AT4G36010	Biotic stress	-13.27	-15.87
disease resistance-like protein/LRR domain-containing protein	AT2G34930	Biotic stress	-20.26	-31.34
glycine-rich protein	AT5G28630	Biotic stress	-23.90	-18.15
cytochrome P450, family 94, subfamily C, polypeptide 1	AT2G27690	Biotic stress	-25.21	-34.89
cytochrome P450, family 94, subfamily B, polypeptide 1	AT5G63450	Biotic stress	-31.68	-28.26
COBRA-like protein 5	AT5G60950	Biotic stress	-	4.35
lipase class 3 family protein / disease resistance protein-related protein (EDS1B)	AT3G48080	Biotic stress	-	3.34
chitinase	AT1G02360	Biotic stress	-	3.19
alpha dioxygenase	AT1G73680	Biotic stress	-	3.18
Chitinase family protein	AT4G01700	Biotic stress	-	3.13
MLO-like protein	AT2G39200	Biotic stress	-	2.94
flavin monooxygenase.	AT1G12200	Biotic stress	-	2.64
receptor like protein 2	AT1G17240	Biotic stress	-	2.61
receptor like protein 43	AT3G28890	Biotic stress	-	2.49

BAX inhibitor 1	AT5G47120	Biotic stress	-	2.46
LRR receptor-like serine/threonine-protein kinase	AT1G74360	Biotic stress	-	2.30
disease resistance protein	AT4G14610	Biotic stress	-	2.16
late embryogenesis abundant hydroxyproline-rich glycoprotein	AT3G44220	Biotic stress	-	-2.06
elicitor peptide 4	AT5G09980	Biotic stress	-	-2.12
pathogenesis-related thaumatin family protein	AT4G36000	Biotic stress	-	-2.23
carboxyesterase 5	AT1G49660	Biotic stress	-	-2.32
alkenal/one oxidoreductase	AT1G23740	Biotic stress	-	-2.35
glucan endo-1,3-beta-glucosidase 10	AT5G42100	Biotic stress	-	-2.36
hydroxyproline-rich glycoprotein family protein	AT4G25620	Biotic stress	-	-2.48
pathogenesis-related protein	AT4G25780	Biotic stress	-	-3.44
glycine-rich protein	AT3G04640	Biotic stress	-	-3.65
			-	-
thioredoxin-dependent peroxidase 2	AT1G65970	Oxidative stress	56.04	1.00
oxidoreductase, 2OG-Fe(II) oxygenase family protein	AT4G10500	Oxidative stress	1.00	17.39
glutathione S-transferase TAU 12	AT1G69920	Oxidative stress	1.00	5.08
peroxidase 4	AT1G14540	Oxidative stress	1.00	5.02
glutathione S-transferase TAU 10	AT1G74590	Oxidative stress	22.36	32.75
Thioredoxin superfamily protein	AT5G38900	Oxidative stress	21.93	32.83
glutathione S-transferase 6	AT1G02930	Oxidative stress	1.00	7.26
apoplastic peroxidase Prx37	AT4G08770	Oxidative stress	27.27	32.41
Glutathione S-transferase family protein	AT5G44990	Oxidative stress	6.36	9.24
L-ascorbate oxidase	AT4G39830	Oxidative stress	-	2.14
catalase 3	AT1G20620	Oxidative stress	2.44	2.71
peroxidase 5	AT1G14550	Oxidative stress	37.46	23.52
PLAC8 family protein	AT1G52200	Oxidative stress	-	5.41
glutathione S-transferase tau 3	AT2G29470	Oxidative stress	32.47	27.15
peroxidase 52	AT5G05340	Oxidative stress	27.20	24.36
peroxidase	AT5G06730	Oxidative stress	16.52	14.84
peroxidase 38	AT4G08780	Oxidative stress	10.00	-
glutathione S-transferase TAU 11	AT1G69930	Oxidative stress	9.08	8.50
peroxidase 50	AT4G37520	Oxidative stress	8.68	11.29
glutathione transferase lambda 1	AT5G02780	Oxidative stress	8.41	12.28
Regulator of Vps4 activity in the MVB pathway protein	AT1G13340	Oxidative stress	6.37	8.39
peroxidase	AT5G19880	Oxidative stress	6.18	7.90
glutaredoxin ATGRXS13	AT1G03850	Oxidative stress	5.36	5.97
peroxidase 71	AT5G64120	Oxidative stress	4.71	8.18
chlorophyll(ide) b reductase NYC1	AT4G13250	Oxidative stress	4.55	-
glutathione S-transferase F2	AT4G02520	Oxidative stress	3.81	5.63
glutaredoxin-like protein	AT1G64500	Oxidative stress	3.48	3.06
glutathione peroxidase GPx	AT2G31570	Oxidative stress	2.84	-
glutathione S-transferase zeta-class 1	AT2G02390	Oxidative stress	2.49	2.85
aconitate hydratase 3	AT2G05710	Oxidative stress	2.46	2.51
membrane-associated progesterone binding protein 3	AT3G48890	Oxidative stress	2.42	2.75
glucose-6-phosphate dehydrogenase 4	AT1G09420	Oxidative stress	2.41	2.81
Aldolase-type TIM barrel family protein	AT5G13420	Oxidative stress	2.39	2.52
glutathione S-transferase	AT1G65820	Oxidative stress	2.37	2.72
aconitase 2	AT4G26970	Oxidative stress	2.28	2.59
protein reduce transmission through pollen	AT1G60420	Oxidative stress	2.20	2.31
Pyridoxamine 5'-phosphate oxidase family protein	AT2G04690	Oxidative stress	2.19	2.26
glutaredoxin-C1	AT5G63030	Oxidative stress	2.14	2.39
glutaredoxin-C3	AT1G77370	Oxidative stress	2.14	2.34
GroES-like zinc-binding alcohol dehydrogenase family protein	AT5G63620	Oxidative stress	2.13	1.82
Thioredoxin	AT5G39950	Oxidative stress	2.06	-
Pyridoxamine 5'-phosphate oxidase family protein	AT2G04690	Oxidative stress	2.05	2.14
atypical CYS HIS rich thioredoxin 4	AT1G08570	Oxidative stress	2.05	2.11
Thioredoxin H-type 2 - Arabidopsis thaliana (Mouse-ear cress), partial (74%)	AT5G39950	Oxidative stress	1.94	2.07
NADPH--cytochrome P450 reductase 1	AT4G24520	Oxidative stress	1.94	1.99
uncharacterized protein	AT3G14430	Oxidative stress	1.89	2.04
thioredoxin-like protein	AT1G21350	Oxidative stress	-1.93	-2.01
SOUL heme-binding protein	AT5G20140	Oxidative stress	-1.96	-2.00
thioredoxin F-type 1	AT3G02730	Oxidative stress	-1.99	-
2-Cysteine peroxiredoxin	AT5G06290	Oxidative stress	-2.08	-
Glutaredoxin family protein	AT5G01420	Oxidative stress	-2.11	-
monothiol glutaredoxin-S2	AT5G18600	Oxidative stress	-2.12	-3.14
Thioredoxin superfamily protein	AT2G37240	Oxidative stress	-2.24	-2.55
Fe superoxide dismutase 2	AT5G51100	Oxidative stress	-2.39	-2.28
tRNA dihydrouridine synthase	AT3G63510	Oxidative stress	-2.39	-2.74
glutathione transferase lambda 2	AT3G55040	Oxidative stress	-2.49	-2.31
thioredoxin-like 2-2	AT4G29670	Oxidative stress	-2.59	-2.62
2-Cys peroxiredoxin BAS1	AT3G11630	Oxidative stress	-2.76	-2.60

laccase 8	AT5G01040	Oxidative stress	-3.21	-2.62
glutaredoxin-C12	AT2G47870	Oxidative stress	-3.23	-3.12
monothiol glutaredoxin-S1	AT1G03020	Oxidative stress	-3.36	-
glyoxylate reductase 2	AT1G17650	Oxidative stress	-3.50	-3.34
monothiol glutaredoxin-S9	AT2G30540	Oxidative stress	-3.69	-3.97
peroxiredoxin Q	AT3G26060	Oxidative stress	-4.09	-
monothiol glutaredoxin-S4	AT4G15680	Oxidative stress	-4.31	-5.92
monothiol glutaredoxin-S8	AT4G15660	Oxidative stress	-4.54	-4.79
glutathione S-transferase F11	AT3G03190	Oxidative stress	-4.59	-4.41
glutaredoxin-like protein	AT2G41330	Oxidative stress	-5.40	-7.70
monothiol glutaredoxin-S11	AT1G06830	Oxidative stress	-6.74	-3.63
serine-type endopeptidase inhibitor	AT1G72060	Oxidative stress	-	3.13
Homeobox even-skipped homolog protein 1 (EVX-1). - Gallus gallus, partial (9%)	AT4G08390	Oxidative stress	-	2.35
L-ascorbate peroxidase 1	AT1G07890	Oxidative stress	-	2.14
6-phosphogluconate dehydrogenase, decarboxylating 3	AT3G02360	Oxidative stress	-	2.05
glutaredoxin-C5	AT4G28730	Oxidative stress	-	-2.49
monothiol glutaredoxin-S7	AT4G15670	Oxidative stress	-	-4.40
NAD(P)-binding Rossmann-fold-containing protein	AT1G32220	Oxidative stress	-3.61	-3.56
			-	-
pectin methylesterase 17 (PME17)	AT2G45220	Cell Wall	93.89	162.26
hydroxyproline-rich glycoprotein family protein	AT5G51680	Cell Wall	1.00	7.18
pectin methylesterase 20 (PME20)	AT2G47550	Cell Wall	25.95	15.50
caffeoyl-CoA 3-O-methyltransferase	AT1G67980	Cell wall	15.66	24.75
expansin-like A1	AT3G45970	Cell Wall	-43.15	-53.94
caffeic acid/5-hydroxyferulic acid O-methyltransferase	AT5G54160	Cell wall	1.00	5.49
extensin 3	AT1G21310	Cell Wall	37.37	29.98
peptidoglycan-binding LysM domain-containing protein	AT5G62150	Cell Wall	5.24	8.06
UDP-D-glucuronate 4-epimerase 1	AT4G30440	Cell Wall	-3.70	-4.09
cinnamyl alcohol dehydrogenase 5	AT4G34230	Cell wall	-	5.07
extensin 4	AT1G76930	Cell Wall	31.53	27.64
mannose-6-phosphate isomerase	AT1G67070	Cell Wall	18.17	15.00
FAD-binding and BBE domain-containing protein	AT5G44380	Cell wall	12.32	17.40
polygalacturonase /pectinase	AT2G43870	Cell Wall	10.60	7.54
UDP-arabinose 4-epimerase 4	AT5G44480	Cell Wall	8.68	9.79
Proline-rich extensin-like family protein	AT2G43150	Cell Wall	7.70	6.55
fucosyltransferase 6	AT1G14080	Cell Wall	6.02	6.65
peroxidase 34	AT3G49120	Cell wall	4.36	-
expansin A1	AT1G69530	Cell Wall	4.29	4.97
trans-cinnamate 4-monooxygenase	AT2G30490	Cell wall	4.17	4.51
Proline-rich extensin-like family protein	AT2G24980	Cell wall	3.93	2.96
cinnamyl-alcohol dehydrogenase	AT1G72680	Cell wall	3.52	3.29
UDP-arabinose 4-epimerase 3	AT4G20460	Cell Wall	3.24	3.81
Proline-rich extensin-like family protein	AT4G08410	Cell wall	3.18	-
lectin-like protein	AT5G03350	Cell wall	3.16	2.02
xyloglucan endotransglucosylase/hydrolase protein 29	AT4G18990	Cell wall	3.15	-
Exostosin family protein	AT3G42180	Cell wall	2.93	-
ferulic acid 5-hydroxylase 1	AT4G36220	Cell wall	2.86	3.15
glucuronidase 2	AT5G07830	Cell wall	2.82	3.71
Proline-rich extensin-like family protein	AT1G23720	Cell wall	2.72	2.19
uncharacterized protein	AT5G24460	Cell wall	2.05	1.79
cinnamoyl coa reductase 1	AT1G15950	Cell wall	1.99	2.36
GDSL esterase/lipase	AT1G28600	Cell wall	-1.89	-2.55
uncharacterized protein	AT3G08600	Cell wall	-1.95	-2.15
uncharacterized protein	AT2G25800	Cell wall	-1.97	-2.24
LysM domain-containing GPI-anchored protein 2	AT1G77630	Cell wall	-2.00	-2.21
pectinacetylesterase family protein	AT3G62060	Cell Wall	-2.01	-2.07
uncharacterized protein	AT3G13674	Cell wall	-2.01	-
arabinose 5-phosphate isomerase	AT3G54690	Cell Wall	-2.07	-2.03
mannan synthase 7	AT2G35650	Cell Wall	-2.09	-2.70
cinnamoyl-CoA reductase	AT1G80820	Cell wall	-2.16	-1.62
polygalacturonase-like protein	AT3G06770	Cell Wall	-2.24	-3.44
UDP-D-glucuronate 4-epimerase 6	AT3G23820	Cell Wall	-2.25	-2.36
glycosyl hydrolase 9B7	AT1G75680	Cell wall	-2.26	-2.25
pectin lyase-like superfamily protein	AT3G09540	Cell Wall	-2.30	-1.94
UDP-glucose 6-dehydrogenase 2	AT5G39320	Cell Wall	-2.31	-2.10
uncharacterized protein	AT5G01590	Cell wall	-2.36	-2.24
leucine-rich repeat-containing protein	AT1G33600	Cell Wall	-2.38	-1.77
beta-D-xylosidase 5	AT3G19620	Cell Wall	-2.39	-2.85
leucine-rich repeat extensin-like protein 5	AT4G18670	Cell Wall	-2.39	-3.05
FASCICLIN-like arabinogalactan protein 8	AT2G45470	Cell Wall	-2.48	-2.43
pectin methylesterase 34 (PME34)	AT3G49220	Cell Wall	-2.49	-3.12

pectinesterase	AT3G43270	Cell Wall	-2.56	-2.43
pectate lyase 1	AT1G04680	Cell Wall	-2.60	-2.11
peptidoglycan-binding LysM domain-containing protein	AT5G23130	Cell wall	-2.65	-3.00
pectinacetylesterase family protein	AT5G23870	Cell wall	-2.65	-
extensin-like protein	AT1G12090	Cell wall	-2.73	-2.72
expansin A5	AT3G29030	Cell Wall	-2.80	-2.43
UDP-glucose 6-dehydrogenase 1	AT3G29360	Cell Wall	-2.83	-2.57
mannan synthase 3	AT1G23480	Cell Wall	-2.84	-3.14
mannose-6-phosphate isomerase	AT3G02570	Cell Wall	-2.96	-2.73
pectinacetylesterase family protein	AT5G23870	Cell wall	-3.03	-
GDSL esterase/lipase	AT1G29670	Cell wall	-3.25	-2.66
cellulose synthase A5 (CESA5)	AT5G09870	Cell Wall	-3.51	-3.73
Alpha-expansin precursor	AT3G29030	Cell Wall	-3.73	-3.42
fasciclin-like arabinogalactan protein 7	AT2G04780	Cell Wall	-3.96	-3.07
fasciclin-like arabinogalactan protein 2	AT4G12730	Cell Wall	-3.98	-4.09
FASCICLIN-like arabinogalactan protein 18 precursor	AT3G11700	Cell Wall	-4.02	-4.67
pectinacetylesterase family protein	AT5G45280	Cell Wall	-4.14	-4.29
fasciclin-like arabinogalactan protein 9	AT1G03870	Cell Wall	-4.20	-4.63
GDSL esterase/lipase	AT5G14450	Cell wall	-4.25	-4.49
curculin-like (mannose-binding) lectin family protein	AT1G78850	Cell wall	-4.32	-
xyloglucan glycosyltransferase 4 (Cellulose synthase-like C4)	AT3G28180	Cell Wall	-4.41	-6.04
esterase/lipase/thioesterase family protein	AT5G22460	Cell wall	-4.54	-
early nodulin-like protein 17	AT5G15350	Cell wall	-4.86	-5.34
SKU5-like 5 protein	AT1G76160	Cell wall	-4.95	-
protein EXORDIUM like 3	AT5G51550	Cell wall	-5.22	-5.11
arabinogalactan protein 20	AT3G61640	Cell Wall	-5.26	-6.68
peroxidase 31	AT3G28200	Cell wall	-6.10	-8.12
Lysine-rich arabinogalactan protein	AT2G23130	Cell Wall	-6.28	-7.42
arabinogalactan protein 21	AT1G55330	Cell Wall	-6.91	-6.52
leucine-rich repeat extensin-like protein 3	AT4G13340	Cell Wall	-7.05	-7.49
arabinogalactan protein 1	AT5G64310	Cell Wall	-7.24	-8.43
Lysine-rich arabinogalactan protein	AT2G23130	Cell Wall	-8.96	-10.06
uncharacterized protein	AT2G33570	Cell wall	-9.24	-11.37
Lysine-rich arabinogalactan protein 18	AT4G37450	Cell Wall	-9.33	-7.70
expansin-like A2 (EXLA2)	AT4G38400	Cell Wall	-9.45	-12.12
pectinesterase 25	AT3G10720	Cell Wall	-10.85	-13.50
uncharacterized protein	AT4G35320	Cell wall	-10.91	-11.01
fasciclin-like arabinogalactan protein 13	AT5G44130	Cell Wall	-11.40	-8.84
expansin-like A3	AT3G45960	Cell Wall	-28.69	-27.84
pectin lyase-like superfamily protein	AT1G02460	Cell Wall	-	2.81
hydroxycinnamoyl-CoA shikimate/quinate hydroxycinnamoyl transferase	AT5G48930	Cell Wall	-	2.63
caffeoyl-CoA O-methyltransferase	AT4G34050	Cell wall	-	2.11
pectin lyase-like superfamily protein	AT4G33440	Cell Wall	-	-2.05
pectinacetylesterase family protein	AT3G05910	Cell Wall	-	-2.12
polygalacturonase	AT4G23500	Cell Wall	-	-2.62
arabinogalactan protein 12	AT3G13520	Cell Wall	-	-2.63
cinnamoyl-CoA reductase like protein	AT4G30470	Cell wall	-	-2.64
O-Glycosyl hydrolases family 17 protein	AT3G55430	Cell wall	-	-2.77
GDSL esterase/lipase	AT3G16370	Cell wall	-	-2.93
Cellulose-synthase-like C12	AT4G07960	Cell Wall	-	-4.45
ornithine N-delta-acetyltransferase	AT2G39030	Hormone	-	14.80
ethylene-responsive transcription factor ERF096	AT5G43410	Hormone	12.70	8.87
indole-3-acetic acid-amido synthetase GH3.3	AT2G23170	Hormone	9.10	4.20
aldehyde oxidase 1	AT5G20960	Hormone	8.09	12.14
1-aminocyclopropane-1-carboxylate oxidase-like protein 11	AT5G59530	Hormone	7.84	10.57
Mediator of ABA-regulated dormancy MARD1	AT3G63210	Hormone	5.61	4.71
SAUR-like auxin-responsive protein	AT4G38860	Hormone	4.15	2.89
aldo-keto reductase 5	AT1G60730	Hormone	4.07	5.47
2-oxoglutarate (2OG) and Fe(II)-dependent oxygenase-like protein	AT2G30840	Hormone	3.66	4.65
adenine nucleotide alpha hydrolases-like protein	AT1G09740	Hormone	3.59	4.13
BES1/BZR1 1	AT3G50750	Hormone	2.73	2.49
auxin-induced in root cultures protein 12	AT3G07390	Hormone	2.57	2.57
SAUR-like auxin-responsive protein	AT3G60690	Hormone	2.50	3.74
gibberellin receptor GID1L2	AT3G63010	Hormone	2.42	3.24
ABRE binding factor 4	AT3G19290	Hormone	2.39	2.48
jacalin-like lectin domain-containing protein	AT1G52100	Hormone	2.34	2.54
abscisic acid responsive element-binding factor 1	AT1G49720	Hormone	2.12	2.27
auxin signaling F-box 3 protein	AT1G12820	Hormone	2.11	2.05
gibberellin receptor GID1L3	AT5G27320	Hormone	2.08	2.04
dormancy/auxin associated protein	AT2G33830	Hormone	2.03	1.97
IAA-amino acid hydrolase ILR1-like 3	AT5G54140	Hormone	1.97	2.68

12-oxophytodienoate reductase-like protein 1	AT1G09400	Hormone	1.92	2.20
brassinosteroid-responsive RING-H2	AT3G61460	Hormone	1.63	2.69
gibberellin 2-beta-dioxygenase 2	AT1G30040	Hormone	-1.23	-2.56
O-fucosyltransferase-like protein	AT3G02250	Hormone	-1.87	-2.08
transcription factor TCP21	AT5G08330	Hormone	-1.93	-2.59
SAUR-like auxin-responsive family protein	AT4G34750	Hormone	-1.96	-2.22
ferredoxin C2	AT1G32550	Hormone	-2.09	-2.19
12-oxophytodienoate reductase-like protein 2B	AT1G18020	Hormone	-2.14	-2.79
Ent-kaur-16-ene synthase	AT1G79460	Hormone	-2.50	-2.27
epithiospecifier protein	AT1G54040	Hormone	-2.59	-2.72
delta(14)-sterol reductase	AT3G52940	Hormone	-2.65	-3.10
S-adenosyl-L-methionine-dependent methyltransferase	AT3G21950	Hormone	-2.73	-2.70
uncharacterized protein	AT5G40460	Hormone	-2.86	-2.68
cytochrome P450 90B1 (steroid 22-alpha-hydrolase CYP90B1)	AT3G50660	Hormone	-2.90	-3.86
basic helix-loop-helix domain-containing protein	AT2G31730	Hormone	-2.98	-3.05
arabinogalactan protein 15	AT5G11740	Hormone	-3.17	-2.46
gibberellin 2-oxidase 6	AT1G02400	Hormone	-3.32	-3.47
heptahelical transmembrane protein2	AT4G30850	Hormone	-4.08	-3.15
SAUR-like auxin-responsive protein family	AT1G72430	Hormone	-4.98	-5.73
O-fucosyltransferase family protein	AT5G01100	Hormone	-5.94	-4.45
allene oxide synthase	AT5G42650	Hormone	-6.25	-3.62
cold and ABA inducible protein kin1	AT5G15960	Hormone	-6.60	-5.39
lipoxygenase 4	AT1G72520	Hormone	-7.84	-7.95
Col-0 2-oxoglutarate-dependent dioxygenase (AOP2) pseudogene	AT4G03060	Hormone	-9.18	-6.67
O-fucosyltransferase family protein	AT5G65470	Hormone	-9.32	-12.97
1-aminocyclopropane-1-carboxylate synthase 11	AT4G08040	Hormone	-9.69	-9.27
SAUR-like auxin-responsive protein	AT3G09870	Hormone	-13.06	-18.55
ethylene-responsive transcription factor ERF098	AT3G23230	Hormone	-17.01	-14.94
multiprotein-bridging factor 1c	AT3G24500	Hormone	-	3.64
nitrile specifier protein 5	AT5G48180	Hormone	-	3.38
ethylene-responsive transcription factor ERF094	AT1G06160	Hormone	-	3.37
SAUR-like auxin-responsive protein	AT1G16510	Hormone	-	2.44
AT-hook protein of GA feedback 2	AT3G55560	Hormone	-	2.43
receptor-like protein kinase BRI1-like 3	AT3G13380	Hormone	-	2.26
12-oxophytodienoate reductase 1	AT1G76680	Hormone	-	-2.58
auxin-responsive protein	AT4G12980	Hormone	-	-2.75
abscisic acid 8'-hydroxylase 1	AT4G19230	Hormone	-	-2.78
SAUR-like auxin-responsive protein 9	AT4G34760	Hormone	-	-3.16
ethylene-responsive transcription factor 11	AT1G28370	Hormone	-	-5.59
SAUR-like auxin-responsive protein	AT4G12410	Hormone	-	-7.60
ethylene responsive element binding factor 6	AT4G17490	Hormone	-	-8.30
S-adenosyl-L-methionine-dependent methyltransferase	AT1G15125	Hormones	-	5.81
alternative oxidase 1D	AT1G32350	Hormones	34.21	-
1-aminocyclopropane-1-carboxylate synthase 2	AT1G01480	Hormones	13.43	-
indole-3-acetic acid-amido synthetase GH3.2	AT4G37390	Hormones	9.53	-
S-adenosyl-L-methionine-dependent methyltransferase-like protein	AT5G54400	Hormones	8.54	-
aluminum induced protein with YGL and LRDR motifs	AT4G27450	Hormones	3.30	-
SAUR-like auxin-responsive protein	AT2G28085	Hormones	3.02	-
ethylene-responsive transcription factor ERF060	AT4G39780	Hormones	2.95	2.94
IAA-amino acid hydrolase ILR1	AT3G02875	Hormones	2.80	-
ethylene-responsive transcription factor ERF070	AT1G71130	Hormones	2.10	2.19
SAUR-like auxin-responsive protein	AT4G36110	Hormones	2.02	1.63
UDP-glucosyl transferase 71B6	AT3G21780	Hormones	1.99	-
AP2-like ethylene-responsive transcription factor AIL5	AT5G57390	hormones	-1.80	-2.28
HVA22-like protein e	AT5G50720	Hormones	-2.17	-1.70
methyltransferase S-adenosyl-L-methionine-dependent methyltransferase	AT4G29590	Hormones	-2.37	-2.38
S-adenosyl-L-methionine-dependent methyltransferase-like protein	AT4G24805	Hormones	-2.38	-2.36
cytokinin oxidase/dehydrogenase 6	AT3G63440	Hormones	-2.52	-
ethylene-responsive transcription factor ERF058	AT1G22190	Hormones	-2.59	-4.07
GRAM domain-containing protein / ABA-responsive protein-related	AT5G23350	Hormones	-2.60	-
SAUR-like auxin-responsive protein	AT4G00880	Hormones	-2.74	-
brassinosteroid metabolic pathway protein BEN1	AT2G45400	hormones	-2.95	-3.79
auxin-responsive protein IAA19	AT3G15540	Hormones	-3.17	-
auxin-responsive protein IAA17	AT1G04250	Hormones	-3.60	-3.70
S-adenosylmethionine-dependent methyltransferase domain-containing protein	AT1G69523	Hormones	-4.22	-3.49
auxin-responsive protein IAA5	AT1G15580	Hormones	-4.44	-4.81
indole-3-acetic acid 6	AT1G52830	Hormones	-10.55	-17.27
ethylene-responsive transcription factor ERF043	AT4G32800	Hormones	-11.20	-13.07
ethylene-responsive transcription factor ERF013	AT1G77640	Hormones	-16.37	-12.92
ethylene-responsive transcription factor ERF022	AT1G33760	Hormones	-24.81	-48.29
ethylene-responsive transcription factor ERF109	AT4G34410	Hormones	-28.69	-

p-Value > 0,05 - Fold change 2

auxin response factor 16	AT4G30080	Hormones	-	2.06
auxin-responsive protein IAA7	AT3G23050	Hormones	-	-2.17
cytokinin riboside 5'-monophosphate phosphoribohydrolase LOG1	AT2G28305	Hormones	-	-2.78
ethylene-responsive transcription factor RAP2-10	AT4G36900	Hormones	-	-2.94
ethylene-responsive transcription factor ERF018	AT1G74930	Hormones	-	-3.35
ethylene-responsive transcription factor CRF2	AT4G23750	Hormones	-	-3.53
ethylene-responsive transcription factor ABR1	AT5G64750	Hormones	-	-11.08
			-	-
mediator of RNA polymerase II transcription subunit 37b	AT1G09080	Abiotic Stress	-	6.41
heat shock protein 90.1	AT5G52640	Abiotic Stress	-	6.36
Late embryogenesis abundant-related protein	AT1G54890	Abiotic stress	32.14	25.30
FAD-binding and BBE domain-containing protein	AT1G26410	Abiotic stress	28.31	36.41
germin-like protein subfamily 1 member 19	AT5G39180	Abiotic Stress	24.06	24.09
acireductone dioxygenase 3	AT2G26400	Abiotic stress	11.33	-
mediator of RNA polymerase II transcription subunit 37b	AT1G09080	Abiotic Stress	6.95	8.70
UDP-glycosyltransferase family protein	AT4G19460	Abiotic stress	4.45	4.42
polyketide cyclase/dehydrase and lipid transport superfamily protein	AT1G23120	Abiotic Stress	4.34	5.57
phosphopantothenoylcysteine decarboxylase	AT1G48605	Abiotic Stress	3.77	3.83
bifunctional nuclease in basal defense response 1	AT1G75380	Abiotic Stress	3.12	3.95
J-domain protein required for chloroplast accumulation response 1	AT1G75100	Abiotic stress	2.82	-
uncharacterized protein	AT2G24550	Abiotic stress	2.51	-
saposin B domain-containing protein	AT3G51730	Abiotic stress	2.17	-
AFG1-like ATPase family protein	AT4G30490	Abiotic stress	2.08	-
C2 domain-containing protein	AT5G55530	Abiotic Stress	2.00	2.12
CP12 domain-containing protein 3	AT1G76560	Abiotic stress	1.88	2.04
protein LOW PHOSPHATE ROOT2	AT1G71040	Abiotic Stress	1.83	2.01
ABI five binding protein 2	AT1G13740	Abiotic stress	-1.89	-2.23
uncharacterized protein	AT4G33985	Abiotic stress	-1.92	-2.12
methyltransferase PMT3	AT4G14360	Abiotic Stress	-2.04	-2.27
cold regulated 314 thylakoid membrane 2	AT1G29390	Abiotic stress	-2.05	-1.92
germin-like protein subfamily 3 member 1	AT1G72610	Abiotic Stress	-2.07	-2.27
glutathione S-transferase U17	AT1G10370	Abiotic stress	-2.19	-
BCL-2-associated athanogene 1	AT5G52060	Abiotic Stress	-2.21	-1.90
dehydration-induced protein ERD15	AT2G41430	Abiotic Stress	-2.34	-2.25
dehydrin ERD14	AT1G76180	Abiotic Stress	-2.51	-2.13
Heat shock protein 81-3	AT2G35880	Abiotic stress	-2.56	-2.97
methyltransferase PMT14	AT4G18030	Abiotic Stress	-2.75	-3.08
protein dehydration-INDUCED 19-5	AT4G02200	Abiotic Stress	-2.83	-2.96
uncharacterized protein	AT2G32240	Abiotic stress	-2.83	-
methyltransferase PMT8	AT1G04430	Abiotic Stress	-3.07	-4.04
fumarate hydratase 2	AT5G50950	Abiotic Stress	-3.08	-
glutathione S-transferase tau 26	AT1G17190	Abiotic Stress	-3.16	-
chaperone DnaJ-domain containing protein	AT5G21430	Abiotic Stress	-3.34	-2.73
uncharacterized protein	AT1G51090	Abiotic stress	-3.86	-6.29
dehydrin COR47	AT1G20440	Abiotic Stress	-4.80	-5.56
heat shock protein class V 15.4	AT4G21870	Abiotic Stress	-5.52	-3.30
ERD4 protein	AT1G11960	Abiotic Stress	-5.90	-7.68
low-temperature-responsive protein 78/desiccation-responsive protein 29A	AT5G52310	Abiotic Stress	-6.53	-5.59
dehydrin ERD10	AT1G20450	Abiotic stress	-7.76	-8.95
abscisic acid 8'-hydroxylase 3	AT5G45340	Abiotic stress	-8.50	-19.37
allene oxide cyclase 1	AT3G25760	Abiotic stress	-11.54	-
cold-regulated protein 15b	AT2G42530	Abiotic Stress	-14.86	-12.04
cold-regulated protein 15a	AT2G42540	Abiotic Stress	-49.46	-51.28
chaperone protein dnaJ 8	AT1G80920	Abiotic Stress	-	3.13
germin-like protein 5	AT1G09560	Abiotic Stress	-	2.99
wound-responsive protein	AT4G28240	Abiotic Stress	-	2.03
Hydrophobic protein RCI2A	AT3G05880	Abiotic Stress	-	-2.68
Hydrophobic protein RCI2B	AT3G05890	Abiotic Stress	-	-2.87
calmodulin binding protein 25	AT2G41010	Abiotic Stress	-	-3.10
tetratricopeptide repeat-containing protein	AT2G47440	Abiotic Stress	-	-4.96
			-	-
WRKY transcription factor 38	AT5G22570	DNA binding and transcriptio	16.78	1.00
WRKY transcription factor 62	AT5G01900	DNA binding and transcriptio	8.86	1.00
WRKY transcription factor 51	AT5G64810	DNA binding and transcriptio	9.38	1.00
WRKY transcription factor 50	AT5G26170	DNA binding and transcriptio	4.56	1.00
calcium-binding protein CML47	AT3G47480	Signaling	18.96	41.57
LRR receptor-like protein kinase	AT1G51890	Signaling	11.68	34.47
Lectin-domain containing receptor kinase A4.2	AT5G01550	Signaling	9.40	14.11
calcium-transporting ATPase 12	AT3G63380	Signaling	7.65	16.22
feruloyl CoA ortho-hydroxylase 1	AT3G13610	Cell cycle	35.21	34.02
microtubule-associated protein 18	AT5G44610	Cell cycle	11.75	9.73

ankyrin repeat-containing protein	AT4G14390	Cell cycle	7.17	11.76
centrin 2	AT4G37010	Cell cycle	6.28	9.61
ankyrin repeat family protein	AT5G54610	Cell cycle	6.24	10.53
DNAse I-like superfamily protein	AT2G37440	Cell cycle	2.76	3.67
MA3 domain-containing protein	AT5G63190	Cell cycle	2.40	2.14
actin-related protein 8	AT5G56180	Cell cycle	2.34	2.19
endonuclease/exonuclease/phosphatase family protein	AT4G36050	Cell cycle	2.24	2.27
Ca-2+ dependent nuclease	AT3G56170	Cell cycle	2.15	2.69
actin depolymerizing factor 9	AT4G34970	Cell cycle	2.08	2.35
cell division control protein 48-A	AT3G09840	Cell cycle	2.07	1.95
cyclin-dependent kinase inhibitor 4	AT2G32710	Cell cycle	2.04	2.00
protein accelerated cell death 6	AT4G14400	Cell cycle	2.04	2.80
membrin 11	AT2G36900	Cell cycle	2.03	1.94
actin 8	AT1G49240	Cell cycle	2.03	1.90
cyclin-dependent kinase inhibitor 2	AT3G50630	Cell cycle	2.01	2.05
DNAse I-like superfamily protein	AT3G21530	Cell cycle	2.00	2.31
armadillo repeat-containing kinesin-like protein 2	AT1G01950	Cell cycle	-1.68	-2.14
unknown protein (At1g03420)	AT1G03420	Cell cycle	-1.72	-2.01
FIBRILLIN 4	AT3G23400	Cell cycle	-1.84	-1.99
cell division protein ftsZ-like protein 1	AT5G55280	Cell cycle	-1.94	-2.02
microtubule-associated protein, RP/EB family member 1B	AT5G62500	Cell cycle	-1.95	-2.25
ankyrin repeat-containing protein	AT3G54070	Cell cycle	-2.05	-
protein ABIL1	AT2G46225	Cell cycle	-2.07	-2.04
uncharacterized protein	AT2G41830	Cell cycle	-2.08	-1.92
histone H3	AT1G09200	Cell cycle	-2.08	-1.81
cyclin-B1-4	AT2G26760	Cell cycle	-2.09	-1.89
Tubulin/FtsZ family protein	AT3G52750	Cell cycle	-2.12	-2.03
mitotic spindle checkpoint protein MAD2	AT3G25980	Cell cycle	-2.26	-2.14
microtubule end binding protein EB1A	AT3G47690	Cell cycle	-2.37	-2.61
actin binding Calponin homology domain-containing protein	AT5G55400	Cell cycle	-2.43	-2.40
proline-rich family protein	AT5G07020	Cell cycle	-2.47	-2.35
ankyrin repeat family protein	AT3G24530	Cell cycle	-2.57	-2.99
cyclin-B1-2	AT5G06150	Cell cycle	-2.60	-
phloem protein 2-A11	AT1G63090	Cell cycle	-2.61	-
transducin/WD40 domain-containing protein	AT3G51930	Cell cycle	-2.61	-2.49
ankyrin repeat domain-containing protein EMB506	AT5G40160	Cell cycle	-2.67	-
3-methyladenine glycosylase I	AT5G44680	Cell cycle	-2.70	-2.54
Actin binding Calponin homology (CH) domain-containing protein	AT5G48460	Cell cycle	-3.14	-
histone H3	AT5G10390	Cell cycle	-3.39	-3.20
copia-like retrotransposon	AT3G28160	Cell cycle	-3.55	-4.61
cyclin-A1-2	AT1G77390	Cell cycle	-3.91	-4.57
protein SCAR4	AT5G01730	Cell cycle	-4.65	-
tetratricopeptide repeat domain-containing protein	AT3G27960	Cell cycle	-4.99	-5.10
Ankyrin repeat family protein	AT5G54710	Cell cycle	-5.26	-3.73
stress-induced protein KIN2	AT5G15970	Cell cycle	-5.83	-4.83
Ankyrin repeat family protein	AT2G24600	Cell cycle	-7.33	-8.01
ankyrin repeat-containing protein	AT4G03450	Cell cycle	-	3.90
ankyrin repeat-containing protein	AT1G10340	Cell cycle	-	3.11
ankyrin repeat-containing protein	AT4G03460	Cell cycle	-	2.74
autophagy-related protein 8e	AT2G45170	Cell cycle	-	2.41
ankyrin repeat-containing protein	AT3G04140	Cell cycle	-	2.22
protein BRCA2-like B	AT5G01630	Cell cycle	-	-2.04
histone H2B	AT2G37470	Cell cycle	-	-2.08
cyclin p1;1	AT3G63120	Cell cycle	-	-2.17
fimbrin 1	AT4G26700	Cell cycle	-	-2.18
holliday junction resolvase	AT1G12244	Cell cycle	-	-2.55
Ankyrin repeat family protein	AT1G14480	Cell cycle	-	-2.88
ankyrin repeat family protein	AT1G11740	Cell cycle	-	-4.64
histone-lysine N-methyltransferase MEDEA	AT1G02580	Development	6.35	6.18
CLAVATA3/ESR (CLE)-related protein 2	AT1G63245	Development	6.07	8.47
cysteine/histidine-rich C1 domain-containing protein	AT5G43520	Development	4.42	3.25
nodulin MtN21 /EamA-like transporter family protein	AT5G40240	Development	3.95	4.41
protein CLAVATA3/ESR-related 21	AT5G64800	Development	3.81	4.37
nodulin MtN21 /EamA-like transporter family protein	AT5G40230	Development	3.67	3.34
protein seedling plastid development 1	AT3G10420	Development	3.56	2.98
major facilitator protein	AT2G39210	Development	3.10	4.62
dormancy/auxin associated protein	AT1G56220	Development	3.05	3.04
nodulin MtN21-like transporter UMAMIT38	AT4G15540	Development	2.86	3.36
uncharacterized protein	AT1G74940	Development	2.62	3.27
MEI2-like protein 5	AT1G29400	Development	2.46	2.36
nodulin MtN21-like transporter family protein UMAMIT45	AT3G28100	Development	2.34	2.23

Multiple acid move in and out transporter	AT3G28100	Development	2.16	2.09
agamous-like MADS-box protein AGL15	AT5G13790	Development	-2.00	-2.05
seed storage albumin 1	AT4G27140	Development	-2.06	-
Embryo-specific protein 3, (ATS3)	AT2G41475	Development	-2.12	-2.07
squamosa promoter-binding-like protein 2	AT5G43270	Development	-2.25	-
protodermal factor 1	AT2G42840	Development	-2.43	-
MAC/Perforin domain-containing protein	AT1G29690	Development	-2.43	-2.46
late embryogenesis abundant 3-like protein	AT1G02820	Development	-2.47	-2.38
methyltransferase	AT5G10830	Development	-2.73	-2.47
phytosulfokines 5 precursor	AT5G65870	Development	-2.77	-2.14
far-red-elongated hypocotyl1-like protein	AT5G02200	Development	-2.87	-3.61
HAD superfamily, subfamily IIIB acid phosphatase	AT5G44020	Development	-3.27	-3.74
protein FLOWERING LOCUS T	AT1G65480	Development	-3.28	-
uncharacterized protein	AT4G30090	Development	-3.91	-4.37
desiccation-related protein LEA14	AT1G01470	Development	-4.21	-4.01
leucine-rich repeat receptor-like protein CLAVATA2	AT1G65380	Development	-4.52	-3.80
senescence/dehydration related protein	AT2G17840	Development	-5.00	-5.06
protein ULTRAPETALA 1	AT4G28190	Development	-5.61	-
senescence/dehydration-associated protein	AT4G35985	Development	-6.15	-6.81
protein exordium like 5	AT2G17230	Development	-7.63	-9.56
protein exordium like 5	AT2G17230	Development	-9.25	-9.53
NAC transcription factor protein family	AT1G69490	Development	-	12.14
ROTUNDIFOLIA like 17	AT1G13245	Development	-	2.77
uncharacterized protein	AT4G17670	Development	-	2.51
dormancy-associated protein-like 1	AT1G28330	Development	-	2.44
gigantea protein (GI)	AT1G22770	Development	-	2.32
NO-associated protein 1	AT3G47450	Development	-	-2.02
antitermination NusB domain-containing protein	AT4G26370	Development	-	-2.41
nodulin MtN21 /EamA-like transporter family protein	AT3G45870	Development	-	-2.56
maternal effect embryo arrest 18 protein	AT2G34090	Development	-	-2.56
squamosa promoter-binding-like protein 13	AT5G50570	Development	-	-3.28
bidirectional sugar transporter SWEET4	AT3G28007	Development	-	-3.44
tetraspanin10	AT1G63260	Development	-	-4.85
N-MYC downregulated-like 3 protein	AT2G19620	Development	-	-6.13
C2H2-type zinc finger-containing protein	AT2G28710	DNA binding and transcriptio	-	11.04
NAC transcription factor protein family	AT1G69490	DNA binding and transcriptio	14.23	14.78
myb domain protein 122	AT1G74080	DNA binding and transcriptio	9.57	9.45
WRKY transcription factor 61	AT1G18860	DNA binding and transcriptio	8.93	-
cysteine/histidine-rich C1 domain-containing protein	AT2G42060	DNA binding and transcriptio	8.73	10.16
WRKY DNA-binding protein 31	AT4G22070	DNA binding and transcriptio	8.26	-
WRKY transcription factor 8	AT5G46350	DNA binding and transcriptio	7.19	-
myb family transcription factor	AT1G71030	DNA binding and transcriptio	6.40	8.15
WRKY transcription factor 71	AT1G29860	DNA binding and transcriptio	5.55	-
WRKY transcription factor 63	AT1G66600	DNA binding and transcriptio	5.48	5.67
WRKY transcription factor 72	AT5G15130	DNA binding and transcriptio	5.32	-
myb domain protein 13	AT1G06180	DNA binding and transcriptio	5.28	3.18
myb domain protein 45	AT3G48920	DNA binding and transcriptio	5.27	5.00
LOB domain-containing protein 21	AT3G11090	DNA binding and transcriptio	4.97	5.30
PLATZ transcription factor family protein	AT1G76590	DNA binding and transcriptio	4.89	3.52
WRKY transcription factor 41	AT4G11070	DNA binding and transcriptio	4.61	-
zinc finger CCCH domain-containing protein 49	AT4G29190	DNA binding and transcriptio	4.57	5.94
PLATZ transcription factor family protein	AT1G21000	DNA binding and transcriptio	3.96	3.77
LOB domain-containing protein 1	AT1G07900	DNA binding and transcriptio	3.45	-
homeobox-leucine zipper protein HAT9	AT2G22800	DNA binding and transcriptio	3.18	3.25
homeobox protein BEL1-like protein	AT5G41410	DNA binding and transcriptio	3.11	3.47
homeobox-leucine zipper protein ATHB-21	AT2G18550	DNA binding and transcriptio	2.95	2.80
myb domain protein 85	AT4G22680	DNA binding and transcriptio	2.93	3.06
transcription factor MYB3	AT1G22640	DNA binding and transcriptio	2.83	2.59
AP2/B3-like transcriptional factor family protein	AT3G11580	DNA binding and transcriptio	2.78	2.29
plant regulator RWP-RK family protein	AT4G35270	DNA binding and transcriptio	2.75	2.69
myb domain protein 78	AT5G49620	DNA binding and transcriptio	2.74	-
B3 DNA-binding domain transcription factor	AT2G36080	DNA binding and transcriptio	2.69	-
WRKY transcription factor 66	AT1G80590	DNA binding and transcriptio	2.59	-
E2F/DP family winged-helix DNA-binding domain-containing protein	AT4G18870	DNA binding and transcriptio	2.57	2.49
basic leucine zipper 9	AT5G24800	DNA binding and transcriptio	2.52	2.79
GATA transcription factor 27	AT5G47140	DNA binding and transcriptio	2.48	2.54
transcription factor HY5-like protein	AT3G17609	DNA binding and transcriptio	2.47	2.52
MEI2-like 2 protein	AT2G42890	DNA binding and transcriptio	2.45	-
transcription factor bHLH61	AT5G10570	DNA binding and transcriptio	2.43	2.02
cycling DOF factor 2	AT5G39660	DNA binding and transcriptio	2.43	2.50
transcription factor HY5	AT5G11260	DNA binding and transcriptio	2.41	2.19

phospholipid-transporting ATPase 9	AT1G68710	DNA binding and transcriptio	2.36	2.24
transcription factor bHLH66	AT2G24260	DNA binding and transcriptio	2.36	1.95
Calcium-dependent lipid-binding (CaLB domain) family protein	AT1G70810	DNA binding and transcriptio	2.31	3.47
SNF2 , helicase and zinc-finger domain-containing protein	AT1G11100	DNA binding and transcriptio	2.30	2.29
homeobox-leucine zipper protein ATHB-4	AT2G44910	DNA binding and transcriptio	2.27	2.26
PLATZ transcription factor domain-containing protein	AT1G32700	DNA binding and transcriptio	2.26	3.54
transcription factor bHLH148	AT3G06590	DNA binding and transcriptio	2.06	2.35
myb domain protein 14	AT2G31180	DNA binding and transcriptio	2.06	-
WRKY transcription factor 60	AT2G25000	DNA binding and transcriptio	2.05	-
oxidation-related zinc Finger 1	AT2G19810	DNA binding and transcriptio	2.01	2.05
protein argonaute 7 (protein ZIPPY)	AT1G69440	DNA binding and transcriptio	1.97	2.02
BEL1-like homeodomain 10	AT1G19700	DNA binding and transcriptio	1.94	2.18
BEL1-like homeodomain 3	AT1G75410	DNA binding and transcriptio	1.77	2.06
RNA-binding (RRM/RBD/RNP motifs) family protein	AT2G46780	DNA binding and transcriptio	-1.71	-2.11
transcription factor TCP7	AT5G23280	DNA binding and transcriptio	-1.82	-2.22
ribonuclease Z	AT2G04530	DNA binding and transcriptio	-1.88	-2.39
Nucleic acid-binding, OB-fold-like protein	AT3G59980	DNA binding and transcriptio	-1.92	-2.03
RNA recognition motif-containing protein	AT4G09040	DNA binding and transcriptio	-1.95	-2.29
NOL1/NOP2/sun family protein / antitermination NusB domain-containing protein	AT3G13180	DNA binding and transcriptio	-1.97	-2.02
myb family transcription factor	AT3G16350	DNA binding and transcriptio	-2.01	-2.51
chloroplast RNA-binding protein 33	AT3G52380	DNA binding and transcriptio	-2.02	-1.96
DNA-binding storekeeper protein-related transcriptional regulator	AT4G25210	DNA binding and transcriptio	-2.05	-2.04
chloroplast RNA-binding protein 31B	AT5G50250	DNA binding and transcriptio	-2.11	-2.00
Cyclin/Brf1-like TBP-binding protein	AT2G45100	DNA binding and transcriptio	-2.16	-
agamous-like MADS-box protein AGL3	AT2G03710	DNA binding and transcriptio	-2.19	-
WRKY DNA-binding protein 30	AT5G24110	DNA binding and transcriptio	-2.23	-
WUSCHEL-related homeobox 4	AT1G46480	DNA binding and transcriptio	-2.25	-
ribonuclease III-like protein	AT1G55140	DNA binding and transcriptio	-2.28	-2.41
WUSCHEL-related homeobox 2	AT5G59340	DNA binding and transcriptio	-2.32	-
myb domain protein 76	AT5G07700	DNA binding and transcriptio	-2.33	-2.35
RNA recognition motif-containing protein	AT1G67950	DNA binding and transcriptio	-2.34	-2.50
ribosome-binding factor A	AT4G34730	DNA binding and transcriptio	-2.40	-2.66
homeobox-leucine zipper protein ATHB-54	AT1G27045	DNA binding and transcriptio	-2.45	-
Rossmann-fold NAD(P)-binding domain-containing protein	AT4G35250	DNA binding and transcriptio	-2.49	-2.31
DREB subfamily A-4 of ERF/AP2 transcription factor	AT1G63040	DNA binding and transcriptio	-2.62	-
transcription factor TCP9	AT2G45680	DNA binding and transcriptio	-2.64	-
RNA methyltransferase	AT5G10620	DNA binding and transcriptio	-2.69	-2.58
ethylene-responsive transcription factor ERF061	AT1G64380	DNA binding and transcriptio	-2.79	-2.59
bZIP protein	AT5G04840	DNA binding and transcriptio	-2.80	-2.89
myb domain protein 77	AT3G50060	DNA binding and transcriptio	-2.84	-4.78
transcription factor bHLH96	AT1G72210	DNA binding and transcriptio	-2.88	-4.73
chloroplast stem-loop binding protein-41	AT3G63140	DNA binding and transcriptio	-2.91	-
protein AGAMOUS-like 87	AT1G22590	DNA binding and transcriptio	-3.12	-3.51
activation-tagged BRI1 suppressor 1-interacting factor 1	AT3G05800	DNA binding and transcriptio	-3.19	-3.15
homeodomain-like transcriptional regulator	AT5G58900	DNA binding and transcriptio	-3.25	-3.91
RNA recognition motif-containing protein	AT1G22330	DNA binding and transcriptio	-3.27	-4.10
RNA recognition motif-containing protein	AT1G03457	DNA binding and transcriptio	-3.46	-5.48
transcription factor jumonji family protein / zinc finger (C5HC2 type) family protein	AT5G46910	DNA binding and transcriptio	-3.47	-3.24
zinc finger transcription factor BZS1	AT4G39070	DNA binding and transcriptio	-3.53	-3.97
myb domain protein 50	AT1G57560	DNA binding and transcriptio	-3.65	-
two-component response regulator ARR15	AT1G74890	DNA binding and transcriptio	-3.86	-3.09
Myb transcription factor	AT5G62470	DNA binding and transcriptio	-3.98	-3.99
homeobox-leucine zipper protein HAT1	AT4G17460	DNA binding and transcriptio	-4.26	-4.31
two-component response regulator ARR6	AT5G62920	DNA binding and transcriptio	-4.52	-5.49
myb domain protein 29	AT5G07690	DNA binding and transcriptio	-4.76	-
two-component response regulator ARR7	AT1G19050	DNA binding and transcriptio	-4.93	-4.81
PLATZ transcription factor family protein	AT1G43000	DNA binding and transcriptio	-5.17	-3.22
GATA transcription factor 8	AT3G54810	DNA binding and transcriptio	-5.55	-6.85
heat shock transcription factor A3	AT5G03720	DNA binding and transcriptio	-6.79	-6.31
U-box domain-containing protein	AT1G66160	DNA binding and transcriptio	-7.60	-7.97
dual transcription unit and alternative splicing protein GLAUCE	AT1G65450	DNA binding and transcriptio	-8.17	-
transcription factor bHLH137	AT5G50915	DNA binding and transcriptio	-9.29	-11.99
ethylene-responsive transcription factor ERF012	AT1G21910	DNA binding and transcriptio	-57.97	-53.34
ethylene-responsive transcription factor ERF019	AT1G22810	DNA binding and transcriptio	-71.44	-51.21
transcription factor	AT2G02060	DNA binding and transcriptio	-	3.69
cyclic DOF factor 3	AT3G47500	DNA binding and transcriptio	-	3.31
AT-hook motif nuclear-localized protein 1	AT4G12080	DNA binding and transcriptio	-	3.26
homeobox protein 23	AT5G39760	DNA binding and transcriptio	-	3.05
zinc finger protein CONSTANS-LIKE 7	AT1G73870	DNA binding and transcriptio	-	2.76
transcription factor MYB86	AT5G26660	DNA binding and transcriptio	-	2.58
transcription factor TGA7	AT1G77920	DNA binding and transcriptio	-	2.32

transcription factor TGA1	AT5G65210	DNA binding and transcriptio	-	2.32
RNA recognition motif-containing protein	AT4G17720	DNA binding and transcriptio	-	2.14
protein indeterminate(ID)-domain 11	AT3G13810	DNA binding and transcriptio	-	2.12
glycine-rich RNA-binding protein 7	AT2G21660	DNA binding and transcriptio	-	2.05
H/ACA ribonucleoprotein complex, subunit Gar1/Naf1 protein	AT5G18180	DNA binding and transcriptio	-	2.03
RING/FYVE/PHD zinc finger-containing protein	AT3G43230	DNA binding and transcriptio	-	2.01
TRANS-ACTING SIRNA3	AT3G17185	DNA binding and transcriptio	-	-2.05
maternally expressed PAB C-terminal protein	AT3G19350	DNA binding and transcriptio	-	-2.24
PLATZ transcription factor family protein	AT5G46710	DNA binding and transcriptio	-	-2.27
RING/FYVE/PHD zinc finger-containing protein	AT1G77250	DNA binding and transcriptio	-	-2.29
myb domain protein 70	AT2G23290	DNA binding and transcriptio	-	-2.30
C2H2 and C2HC zinc finger-containing protein	AT4G17810	DNA binding and transcriptio	-	-2.32
Dof zinc finger protein DOF5.6	AT5G62940	DNA binding and transcriptio	-	-2.38
uncharacterized protein	AT3G20300	DNA binding and transcriptio	-	-2.42
basic helix-loop-helix domain-containing protein	AT2G42380	DNA binding and transcriptio	-	-2.50
CIA2-like transcription factor	AT4G25990	DNA binding and transcriptio	-	-2.50
LOB domain-containing protein 38	AT3G49940	DNA binding and transcriptio	-	-3.00
transcription factor BHLH32	AT3G25710	DNA binding and transcriptio	-	-3.06
dehydration-responsive element-binding protein 1F	AT1G12610	DNA binding and transcriptio	-	-22.43
photosystem I reaction center subunit psaK	AT1G30380	Light	-2.07	-
protein plastid transcriptionally active 16	AT3G46780	Light	-2.10	-
cofactor assembly, complex C (B6F)	AT5G36120	Light	-2.14	-
carboxylesterase 6	AT1G68620	Metabolism	42.79	44.43
methionine sulfoxide reductase B8	AT4G21840	Metabolism	22.15	38.32
Terpenoid cyclases family protein	AT1G66960	Metabolism	12.85	8.08
glutamate decarboxylase 1	AT5G17330	Metabolism	10.33	10.28
flavonol synthase 5	AT5G63600	Metabolism	9.95	16.36
aldehyde oxidase 1	AT5G20960	Metabolism	8.98	12.31
Inositol monophosphatase family protein	AT5G09290	Metabolism	8.70	9.21
HXXXD-type acyl-transferase-like protein	AT5G42830	Metabolism	8.34	13.64
branched-chain-amino-acid aminotransferase 2	AT1G10070	Metabolism	7.65	16.25
phosphoglycerate mutase-like protein	AT1G09935	Metabolism	7.11	6.02
lipid-transfer protein/seed storage	AT3G22620	Metabolism	6.91	5.99
beta-fructofuranosidase, insoluble isoenzyme CWINV1	AT3G13790	Metabolism	5.92	9.63
2-oxoglutarate (2OG) and Fe(II)-dependent oxygenase superfamily protein	AT2G36690	Metabolism	5.89	11.15
methionine gamma-lyase	AT1G64660	Metabolism	5.58	-
long-chain acyl-CoA synthetase	AT1G64400	Metabolism	5.38	6.58
glycosyl hydrolase 9A2	AT1G65610	Metabolism	5.31	6.81
GDSL esterase/lipase	AT5G03610	Metabolism	5.20	4.59
tryptophan N-monooxygenase 1	AT4G39950	Metabolism	5.15	5.37
alpha 1,4-glycosyltransferase family protein	AT5G01250	Metabolism	4.95	5.12
glutamine synthetase 1;1	AT5G37600	Metabolism	4.78	8.79
GDSL esterase/lipase 4	AT3G14225	Metabolism	4.68	5.80
beta-galactosidase 4	AT5G56870	Metabolism	4.57	4.90
glycolate oxidase	AT4G18360	Metabolism	3.72	3.63
glucuronidase 1	AT5G61250	Metabolism	3.66	4.13
UDP-glycosyltransferase 76E4	AT3G46690	Metabolism	3.57	6.42
pyruvate kinase	AT5G63680	Metabolism	3.54	4.14
flavodoxin-like quinone reductase 1	AT5G54500	Metabolism	3.54	3.95
phospholipase A1-Igamma2	AT2G30550	Metabolism	3.21	3.27
copper amine oxidase	AT3G43670	Metabolism	3.13	3.20
O-methyltransferase-like protein	AT1G33030	Metabolism	2.98	5.23
lysophospholipase 2	AT1G52760	Metabolism	2.91	3.01
D-3-phosphoglycerate dehydrogenase	AT4G34200	Metabolism	2.84	3.09
aldose 1-epimerase	AT3G47800	Metabolism	2.81	2.46
calcium-independent phospholipase A	AT4G19860	Metabolism	2.80	3.11
Long-chain-alcohol oxidase FAO4B	AT4G28570	Metabolism	2.80	3.18
nudix hydrolase 18	AT1G14860	Metabolism	2.79	-
Sphingosine-1-phosphate lyase	AT1G27980	Metabolism	2.78	3.21
cytochrome P450, family 706, subfamily A, polypeptide 4	AT4G12300	Metabolism	2.74	3.22
acyl-coenzyme A oxidase 4	AT3G51840	Metabolism	2.68	2.47
adenine nucleotide alpha hydrolases-domain containing protein kinase	AT1G77280	Metabolism	2.55	2.61
pfkB-like carbohydrate kinase family protein	AT5G43910	Metabolism	2.54	2.69
metallo-beta-lactamase family protein	AT4G33540	Metabolism	2.51	-
SAL3 phosphatase	AT5G63990	Metabolism	2.49	2.17
HXXXD-type acyl-transferase family protein	AT1G28680	Metabolism	2.44	2.48
copper amine oxidase 1	AT1G62810	Metabolism	2.37	2.49
UDP-glycosyltransferase-like protein	AT2G18560	Metabolism	2.37	2.03
strigolactone esterase D14	AT3G03990	Metabolism	2.36	2.40
trehalose-phosphate phosphatase D	AT1G35910	Metabolism	2.36	-
acyl activating enzyme 5	AT5G16370	Metabolism	2.35	2.65

tetraketide alpha-pyrone reductase 2-like protein	AT1G25460	Metabolism	2.33	-
copper amine oxidase 1	AT1G62810	Metabolism	2.30	2.42
metallo-beta-lactamase family protein	AT4G33540	Metabolism	2.30	-
GDSL esterase/lipase	AT1G28580	Metabolism	2.29	2.36
P-loop containing nucleoside triphosphate hydrolases superfamily protein	AT1G04280	Metabolism	2.25	2.53
GDSL esterase/lipase	AT2G38180	Metabolism	2.25	-
alkaline/neutral invertase CINV1	AT1G35580	Metabolism	2.25	2.38
phospholipase A(1) LCAT3	AT3G03310	Metabolism	2.19	-
melibiase family protein	AT3G56310	Metabolism	2.18	2.34
glutathione S-conjugate transporting ATPase	AT1G30400	Metabolism	2.17	2.21
Hydrolases superfamily protein	AT4G00500	Metabolism	2.16	2.23
Cyclopropane-fatty-acyl-phospholipid synthase	AT3G23530	Metabolism	2.16	2.02
aldehyde dehydrogenase 2B4	AT3G48000	Metabolism	2.14	2.12
NAD(P)-binding Rossmann-fold superfamily protein	AT5G52810	Metabolism	2.14	3.89
stearoyl-acyl-carrier-protein desaturase	AT5G16230	Metabolism	2.13	2.04
alpha-galactosidase 1	AT5G08380	Metabolism	2.08	1.83
EPSP synthase	AT2G45300	Metabolism	2.07	2.30
glycine-rich protein / oleosin	AT5G56100	Metabolism	2.07	-
Class-II DAHP synthetase family protein	AT1G22410	Metabolism	2.05	1.87
fatty acid binding protein 2	AT2G26310	Metabolism	2.04	1.92
UDP-glycosyltransferase 72B1	AT4G01070	Metabolism	2.04	1.99
digalactosyldiacylglycerol synthase 1	AT3G11670	Metabolism	2.04	2.11
beta-1,3-galactosyltransferase 2	AT1G05170	Metabolism	1.96	2.37
enoyl-CoA hydratase 2	AT1G76150	Metabolism	1.95	2.16
cysteine synthase C1	AT3G61440	Metabolism	1.92	2.13
adenylate kinase	AT2G39270	Metabolism	1.89	2.09
phosphoglycerate mutase family protein	AT3G60450	Metabolism	1.88	2.45
N2,N2-dimethylguanosine tRNA methyltransferase	AT3G56330	Metabolism	-1.80	-2.18
core-2/I-branching beta-1,6-N-acetylglucosaminyltransferase family protein	AT3G15350	Metabolism	-1.80	-2.37
3-ketoacyl-CoA synthase 9	AT2G16280	Metabolism	-1.93	-2.15
GDSL esterase/lipase LIP-4	AT1G56670	Metabolism	-1.96	-2.15
cytokinin riboside 5'-monophosphate phosphoribohydrolase LOG7	AT5G06300	Metabolism	-2.00	-2.07
adenosine kinase 1	AT3G09820	Metabolism	-2.01	-2.14
uncharacterized protein	AT2G46890	Metabolism	-2.02	-
P-loop containing nucleoside triphosphate hydrolases superfamily protein	AT3G01820	Metabolism	-2.03	-
acyl carrier protein 4	AT4G25050	Metabolism	-2.03	-2.01
3-ketoacyl-CoA synthase 5	AT1G25450	Metabolism	-2.04	-
tRNA pseudouridine synthase	AT2G30320	Metabolism	-2.06	-2.27
glutamine synthetase	AT5G35630	Metabolism	-2.06	-1.94
glutamate--glyoxylate aminotransferase 2	AT1G70580	Metabolism	-2.07	-
dihydrolipoyl dehydrogenase	AT4G16155	Metabolism	-2.09	-2.03
2Fe-2S iron-sulfur cluster binding domain-containing protein	AT4G32590	Metabolism	-2.10	-2.10
aminomethyltransferase	AT1G11860	Metabolism	-2.11	-
1-(5-phosphoribosyl)-5-	AT2G36230	Metabolism	-2.14	-2.28
isoleucyl-tRNA synthetase	AT5G49030	Metabolism	-2.14	-
phosphoglycerate kinase 1	AT3G12780	Metabolism	-2.15	-
bifunctional aspartokinase/homoserine dehydrogenase 1	AT1G31230	Metabolism	-2.15	-2.21
adenylate kinase family protein	AT5G35170	Metabolism	-2.16	-
NAD(P)H:plastoquinone dehydrogenase complex subunit O	AT1G74880	Metabolism	-2.17	-
glyceraldehyde-3-phosphate dehydrogenase (NADP+) (phosphorylating)	AT1G12900	Metabolism	-2.18	-
2Fe-2S iron-sulfur cluster binding domain-containing protein	AT4G32590	Metabolism	-2.18	-2.01
NAD(P)-linked oxidoreductase superfamily protein	AT2G27680	Metabolism	-2.18	-
mevalonate kinase	AT5G27450	Metabolism	-2.18	-2.22
esterase/lipase/thioesterase family protein	AT3G50790	Metabolism	-2.18	-2.61
pfkB-like carbohydrate kinase family protein	AT1G66430	Metabolism	-2.19	-2.31
3-ketoacyl-CoA synthase 10	AT2G26250	Metabolism	-2.19	-
aminotransferase	AT1G77670	Metabolism	-2.20	-2.03
Enoyl-ACP reductase 1	AT2G05990	Metabolism	-2.24	-2.18
ATP synthase protein I-related protein	AT2G31040	Metabolism	-2.24	-
branched-chain-amino-acid aminotransferase 3	AT3G49680	Metabolism	-2.28	-2.26
GDSL esterase/lipase	AT5G45920	Metabolism	-2.29	-2.21
myo-inositol-1-phosphate synthase	AT5G10170	Metabolism	-2.31	-
phosphoglycerate mutase family protein	AT5G62840	Metabolism	-2.31	-2.48
glycine dehydrogenase	AT4G33010	Metabolism	-2.32	-2.02
trehalose-phosphate phosphatase H	AT4G39770	Metabolism	-2.35	-2.92
myo-inositol monophosphatase like 1	AT1G31190	Metabolism	-2.35	-2.04
myo-inositol monophosphatase like 1	AT1G31190	Metabolism	-2.35	-2.14
photosystem II reaction center PsbP family protein	AT1G76450	Metabolism	-2.36	-2.42
2-phosphoglycolate phosphatase 1	AT5G36700	Metabolism	-2.39	-
fructokinase-like 1	AT3G54090	Metabolism	-2.40	-2.78
nucleotide-diphospho-sugar transferase	AT1G64980	Metabolism	-2.40	-3.92

1-deoxy-D-xylulose 5-phosphate reductoisomerase	AT5G62790	Metabolism	-2.42	-2.44
glycine dehydrogenase	AT4G33010	Metabolism	-2.43	-
low psii accumulation2 protein	AT5G51545	Metabolism	-2.46	-2.26
photosystem I light harvesting complex protein 5	AT1G45474	Metabolism	-2.47	-2.10
1,4-alpha-glucan branching enzyme	AT5G03650	Metabolism	-2.48	-2.25
glucan endo-1,3-beta-glucosidase 11	AT1G32860	Metabolism	-2.48	-2.52
glutamate-1-semialdehyde 2,1-aminomutase 2	AT3G48730	Metabolism	-2.49	-2.30
fructose-1,6-bisphosphatase	AT3G54050	Metabolism	-2.50	-
bifunctional sn-glycerol-3-phosphate 2-O-acyltransferase/phosphatase	AT4G00400	Metabolism	-2.50	-2.65
3-hydroxyacyl-ACP dehydratase	AT2G22230	Metabolism	-2.51	-2.62
alpha-galactosidase 2	AT5G08370	Metabolism	-2.52	-
NDH-dependent cyclic electron flow 1	AT3G16250	Metabolism	-2.52	-
PsbP domain-containing protein 5	AT5G11450	Metabolism	-2.57	-2.64
cysteine synthase 26	AT3G03630	Metabolism	-2.59	-2.83
HXXXD-type acyl-transferase-like protein	AT2G39980	Metabolism	-2.62	-4.02
glucose-1-phosphate adenylyltransferase small subunit	AT5G48300	Metabolism	-2.63	-2.19
NAD(P)-linked oxidoreductase superfamily protein	AT1G04420	Metabolism	-2.65	-2.55
phosphomethylpyrimidine synthase	AT2G29630	Metabolism	-2.65	-
aldose 1-epimerase family protein	AT5G66530	Metabolism	-2.67	-2.45
chloroplast thylakoid lumen protein	AT4G02530	Metabolism	-2.70	-2.71
2-C-methyl-D-erythritol 4-phosphate cytidylyltransferase	AT2G02500	Metabolism	-2.73	-2.72
carboxyvinyl-carboxyphosphonate phosphorylmutase	AT1G77060	Metabolism	-2.77	-3.81
lipase domain-containing protein	AT5G50890	Metabolism	-2.82	-2.91
fatty acyl-CoA reductase 1	AT5G22500	Metabolism	-2.87	-3.09
Monogalactosyldiacylglycerol synthase 1	AT4G31780	Metabolism	-2.89	-2.92
hexokinase-like 1	AT1G50460	Metabolism	-2.95	-4.01
tropinone reductase-like protein	AT2G29290	Metabolism	-3.00	-2.85
fatty acid desaturase 7	AT3G11170	Metabolism	-3.04	-2.49
fatty acid hydroxylase 1	AT2G34770	Metabolism	-3.08	-2.88
Flavonoid 3'-monooxygenase	AT5G07990	Metabolism	-3.13	-
alpha-L-fucosidase 2	AT4G34260	Metabolism	-3.13	-4.38
galacturonosyltransferase-like 6	AT4G02130	Metabolism	-3.15	-3.60
FAD/NAD(P)-binding oxidoreductase domain-containing protein	AT1G57770	Metabolism	-3.16	-2.91
adenine phosphoribosyl transferase 3	AT4G22570	Metabolism	-3.22	-3.46
galacturonosyltransferase 15	AT3G58790	Metabolism	-3.23	-4.53
3-ketoacyl-CoA synthase 20	AT5G43760	Metabolism	-3.24	-2.83
UDP-glycosyltransferase family protein	AT5G04480	Metabolism	-3.30	-3.77
3-isopropylmalate dehydrogenase 1	AT1G31180	Metabolism	-3.32	-
Type I inositol-1,4,5-trisphosphate 5-phosphatase 2	AT4G18010	Metabolism	-3.32	-3.93
fatty acid hydroxylase 1	AT2G34770	Metabolism	-3.37	-3.30
adenosylhomocysteinase 2	AT3G23810	Metabolism	-3.40	-
cell wall / vacuolar inhibitor of fructosidase 2	AT5G64620	Metabolism	-3.50	-2.84
phosphoenolpyruvate carboxylase family protein	AT1G21440	Metabolism	-3.53	-3.05
plant glycogenin-like starch initiation protein 7	AT2G35710	Metabolism	-3.54	-4.97
fatty-acid-binding protein 3	AT1G53520	Metabolism	-3.54	-3.33
Haloacid dehalogenase-like hydrolase (HAD) superfamily protein	AT3G48420	Metabolism	-3.70	-
fatty-acid-binding protein 3	AT1G53520	Metabolism	-3.84	-
galacturonosyltransferase-like 8	AT1G24170	Metabolism	-3.86	-4.45
RNA-directed DNA methylase	AT1G13790	Metabolism	-3.96	-4.54
trehalose-phosphate phosphatase-like protein	AT5G51460	Metabolism	-4.18	-5.12
Chalcone-flavanone isomerase family protein	AT5G05270	Metabolism	-4.48	-4.60
isoprenylcysteine alpha-carbonyl methylesterase ICMEL2	AT3G02410	Metabolism	-4.57	-3.81
3-ketoacyl-CoA synthase 4	AT1G19440	Metabolism	-4.58	-4.91
delta-9 desaturase-like 5 protein	AT1G06360	Metabolism	-4.61	-
fatty acid desaturase 8	AT5G05580	Metabolism	-4.64	-4.83
galacturonosyltransferase-like 9	AT1G70090	Metabolism	-4.92	-7.01
acyl-CoA sterol acyl transferase 1	AT3G51970	Metabolism	-5.21	-8.12
isopropylmalate dehydrogenase 1	AT5G14200	Metabolism	-5.23	-3.27
GDSL esterase/lipase	AT4G28780	Metabolism	-5.34	-4.61
cytochrome P450 83A1	AT4G13770	Metabolism	-5.40	-
flavin-containing monooxygenase FMO GS-OX3	AT1G62560	Metabolism	-5.43	-3.65
sodium/metabolite cotransporter BASS5	AT4G12030	Metabolism	-5.68	-3.77
oxidoreductase	AT3G60290	Metabolism	-5.78	-5.05
O-glycosyl hydrolases family 17 protein	AT1G64760	Metabolism	-6.02	-6.83
phosphatidylinositol:ceramide inositolphosphotransferase 1	AT3G54020	Metabolism	-6.10	-6.83
S-adenosylmethionine synthase 4	AT3G17390	Metabolism	-6.44	-4.81
isopropylmalate isomerase 1	AT3G58990	Metabolism	-6.95	-4.66
peptide methionine sulfoxide reductase B5	AT4G04830	Metabolism	-7.20	-
homocysteine S-methyltransferase 3	AT3G22740	Metabolism	-7.37	-
delta-9 acyl-lipid desaturase 1	AT1G06080	Metabolism	-8.05	-
chalcone synthase	AT5G13930	Metabolism	-8.41	-

3-ketoacyl-CoA synthase 1	AT1G01120	Metabolism	-8.42	-6.84
isopropylmalate isomerase 2	AT2G43100	Metabolism	-8.59	-5.55
inositol oxygenase 1	AT1G14520	Metabolism	-9.54	-10.61
methionine sulfoxide reductase B6	AT4G04840	Metabolism	-9.67	-7.84
methylthioalkylmalate synthase 1	AT5G23010	Metabolism	-10.00	-4.82
flavin-containing monooxygenase FMO GS-OX1	AT1G65860	Metabolism	-10.32	-7.61
glutathione S-transferase TAU 20	AT1G78370	Metabolism	-11.00	-7.34
cytidine/deoxycytidylate deaminase-like protein	AT4G29610	Metabolism	-11.80	-16.05
branched-chain aminotransferase4	AT3G19710	Metabolism	-16.76	-
sn-glycerol-3-phosphate 2-O-acyltransferase	AT4G01950	Metabolism	-17.58	-21.69
dihomomethionine N-hydroxylase	AT1G16410	Metabolism	-19.44	-
bifunctional nitrilase/nitrile hydratase NIT4	AT5G22300	Metabolism	-	14.68
beta-fructofuranosidase, insoluble isoenzyme CWINV6	AT5G11920	Metabolism	-	9.91
glutamine amidotransferase	AT1G15040	Metabolism	-	6.72
tc|GB|AC007887.9|AAF79366.1 F15O4.44	AT1G35513	Metabolism	-	5.01
nudix hydrolase 6	AT2G04450	Metabolism	-	4.44
beta glucosidase 17	AT2G44480	Metabolism	-	4.15
glucuronosyl transferase-like protein	AT3G46700	Metabolism	-	3.89
glutamine synthetase 1;4	AT5G16570	Metabolism	-	3.62
indole-3-glycerol phosphate synthase	AT2G04400	Metabolism	-	3.11
anthranilate synthase component I-1	AT5G05730	Metabolism	-	3.00
nudix hydrolase 5	AT2G04430	Metabolism	-	2.75
pyruvate decarboxylase-2	AT5G54960	Metabolism	-	2.66
callose synthase 1	AT1G05570	Metabolism	-	2.43
methyltransferase-like protein	AT1G69520	Metabolism	-	2.37
long-chain acyl-CoA synthetase 7	AT5G27600	Metabolism	-	2.29
aldose 1-epimerase family protein	AT4G25900	Metabolism	-	2.25
carboxylesterase 2	AT1G47480	Metabolism	-	2.19
Dihydroxyacetone kinase	AT1G48430	Metabolism	-	2.01
DNAse I-like superfamily protein	AT1G71710	Metabolism	-	-2.00
pseudouridine synthase family protein	AT5G14460	Metabolism	-	-2.02
myrosinase 1	AT5G26000	Metabolism	-	-2.05
Pseudouridine synthase family protein	AT3G43340	Metabolism	-	-2.07
GDSL esterase/lipase	AT1G09390	Metabolism	-	-2.09
3-ketoacyl-CoA synthase 11	AT2G26640	Metabolism	-	-2.10
fatty acid hydroxylase 2	AT4G20870	Metabolism	-	-2.15
Enoyl-ACP reductase 1	AT2G30200	Metabolism	-	-2.16
glycoside hydrolase	AT3G20440	Metabolism	-	-2.24
glycosyltransferase 18	AT5G62220	Metabolism	-	-2.26
delta1-pyrroline-5-carboxylate synthase 1	AT2G39800	Metabolism	-	-2.29
ferredoxin-like protein	AT1G02180	Metabolism	-	-2.40
nudix hydrolase 7	AT4G12720	Metabolism	-	-2.84
3-ketoacyl-CoA synthase 3	AT1G07720	Metabolism	-	-3.00
nudix hydrolase 8	AT5G47240	Metabolism	-	-3.13
arabinogalactan protein 41	AT5G24105	Metabolism	-	-3.38
aspartyl protease family protein	AT5G48430	Protein synthesis, modificatio	41.55	75.86
aspartyl protease family protein	AT1G44130	Protein synthesis, modificatio	27.44	27.84
U-box domain-containing protein 36	AT3G61390	Protein synthesis, modificatio	12.09	10.46
matrix metalloproteinase	AT1G70170	Protein synthesis, modificatio	9.96	10.45
C3H4 type zinc finger protein	AT5G49665	Protein synthesis, modificatio	7.64	-
exocyst subunit exo70 family protein H2	AT2G39380	Protein synthesis, modificatio	7.60	9.92
chaperone protein dnaJ 11	AT4G36040	Protein synthesis, modificatio	7.51	10.05
F-box/kelch-repeat protein	AT2G44130	Protein synthesis, modificatio	6.98	5.46
E3 ubiquitin-protein ligase ATL41	AT2G42360	Protein synthesis, modificatio	6.09	10.53
calcium-binding protein CML21	AT4G26470	Protein synthesis, modificatio	5.91	4.86
concanavalin A-like lectin kinase-like protein	AT3G45410	Protein synthesis, modificatio	5.71	6.07
F-box/kelch-repeat protein	AT1G80440	Protein synthesis, modificatio	5.49	4.49
vacuolar sorting receptor 6	AT1G30900	Protein synthesis, modificatio	5.35	-
aspartyl protease family protein	AT3G51330	Protein synthesis, modificatio	5.03	10.80
F-box protein	AT3G44326	Protein synthesis, modificatio	4.31	-
subtilase family protein	AT1G32950	Protein synthesis, modificatio	4.15	-
AAA-type ATPase family protein	AT5G57480	Protein synthesis, modificatio	4.00	5.66
AAA-ATPase 1	AT5G40010	Protein synthesis, modificatio	3.97	6.03
ubiquitin-like protein	AT1G53980	Protein synthesis, modificatio	3.90	4.67
aspartyl protease family protein	AT5G10760	Protein synthesis, modificatio	3.67	5.08
uncharacterized protein	AT3G01175	Protein synthesis, modificatio	3.50	3.45
ubiquitin 13	AT1G65350	Protein synthesis, modificatio	3.36	-
RING-H2 finger protein ATL79	AT5G47610	Protein synthesis, modificatio	3.09	2.76
P-loop containing nucleoside triphosphate hydrolases superfamily protein	AT5G17760	Protein synthesis, modificatio	3.01	3.22
RING-H2 finger protein ATL70	AT2G35910	Protein synthesis, modificatio	3.00	3.70
transmembrane Fragile-X-F-associated protein	AT1G68820	Protein synthesis, modificatio	2.97	2.56

RING/U-box domain-containing protein	AT4G26580	Protein synthesis, modificatio	2.89	2.78
RING/U-box domain-containing protein	AT5G55970	Protein synthesis, modificatio	2.87	2.61
adenine nucleotide alpha hydrolases-domain containing protein kinase	AT1G21590	Protein synthesis, modificatio	2.86	2.97
autophagy-related protein 8a	AT4G21980	Protein synthesis, modificatio	2.85	2.96
histone H3 K4-specific methyltransferase SET7/9 family protein	AT1G77660	Protein synthesis, modificatio	2.80	2.55
F-box stress induced protein 2	AT4G21510	Protein synthesis, modificatio	2.69	2.63
Unknown	AT1G18670	Protein synthesis, modificatio	2.67	3.12
vacuolar-processing enzyme gamma	AT4G32940	Protein synthesis, modificatio	2.66	4.61
F-box protein SKP2B	AT1G77000	Protein synthesis, modificatio	2.66	-
F-box domain-containing protein	AT5G18780	Protein synthesis, modificatio	2.59	3.41
autophagy substrate NBR1	AT4G24690	Protein synthesis, modificatio	2.59	2.61
RING/U-box superfamily protein	AT5G24870	Protein synthesis, modificatio	2.58	2.39
RING/U-box domain-containing protein	AT1G63840	Protein synthesis, modificatio	2.55	3.95
ubiquitin 4	AT5G20620	Protein synthesis, modificatio	2.53	-
vacuolar-processing enzyme gamma	AT4G32940	Protein synthesis, modificatio	2.53	4.96
F-box/kelch-repeat protein	AT3G23880	Protein synthesis, modificatio	2.51	2.60
RING/FYVE/PHD zinc finger-containing protein	AT2G37950	Protein synthesis, modificatio	2.50	2.54
uncharacterized protein	AT1G21670	Protein synthesis, modificatio	2.49	2.96
E3 ubiquitin-protein ligase ARI12	AT1G05880	Protein synthesis, modificatio	2.48	2.11
cathepsin B-like cysteine protease	AT4G01610	Protein synthesis, modificatio	2.46	2.48
E3 ubiquitin-protein ligase ARI5	AT1G05890	Protein synthesis, modificatio	2.44	2.18
E3 ubiquitin-protein ligase ARI5	AT1G05890	Protein synthesis, modificatio	2.44	2.26
RING/U-box domain-containing protein	AT1G24440	Protein synthesis, modificatio	2.41	2.26
aspartyl protease family protein	AT3G51360	Protein synthesis, modificatio	2.33	1.88
ARM repeat superfamily protein	AT5G67340	Protein synthesis, modificatio	2.33	3.53
F-box/kelch-repeat protein	AT1G51550	Protein synthesis, modificatio	2.33	2.50
subtilase 4.12	AT5G59090	Protein synthesis, modificatio	2.33	-
RING/U-box domain-containing protein	AT4G19670	Protein synthesis, modificatio	2.32	2.20
F-box/kelch-repeat protein	AT1G23390	Protein synthesis, modificatio	2.29	2.58
F-box/kelch-repeat protein	AT3G24760	Protein synthesis, modificatio	2.28	2.34
RING/U-box domain-containing protein	AT3G47160	Protein synthesis, modificatio	2.26	2.57
peptidase C15, pyroglutamyl peptidase I-like protein	AT1G23440	Protein synthesis, modificatio	2.22	2.46
atypical dual-specicity phosphatase	AT2G32960	Protein synthesis, modificatio	2.20	-
aspartyl protease family protein	AT3G02740	Protein synthesis, modificatio	2.20	2.18
beta-1,3-galactosyltransferase 7	AT1G77810	Protein synthesis, modificatio	2.18	2.92
F-box/RNI-like superfamily protein	AT5G67140	Protein synthesis, modificatio	2.15	-
RING/U-box domain-containing protein	AT3G06330	Protein synthesis, modificatio	2.10	2.35
C3H2C3-type RING E3 Ub ligase	AT4G23450	Protein synthesis, modificatio	2.08	2.06
histone deacetylase 8	AT1G08460	Protein synthesis, modificatio	2.07	2.24
ubiquitin-associated (UBA)/TS-N domain-containing protein	AT2G12550	Protein synthesis, modificatio	2.05	2.07
RNI-like superfamily protein	AT1G80570	Protein synthesis, modificatio	2.04	-
autophagy-related protein 8f	AT4G16520	Protein synthesis, modificatio	2.03	2.05
RNI-like superfamily protein	AT5G07670	Protein synthesis, modificatio	2.02	2.00
DWD motif protein	AT3G45620	Protein synthesis, modificatio	2.01	2.42
RING/U-box superfamily protein	AT1G49850	Protein synthesis, modificatio	2.01	1.80
F-box/kelch-repeat protein SKIP11	AT2G02870	Protein synthesis, modificatio	2.00	1.85
ubiquitin-like protein ATG12A	AT1G54210	Protein synthesis, modificatio	1.99	2.03
chaperone protein dnaJ 20	AT4G13830	Protein synthesis, modificatio	1.99	2.60
RWD domain-containing protein	AT3G60300	Protein synthesis, modificatio	1.98	2.09
Cysteine proteinase	AT4G16190	Protein synthesis, modificatio	1.95	2.18
F-box protein 7	AT1G21760	Protein synthesis, modificatio	1.95	1.99
F-box protein	AT3G07870	Protein synthesis, modificatio	1.95	2.32
RING-finger domain-containing protein	AT4G31450	Protein synthesis, modificatio	1.91	2.04
F-box/kelch-repeat protein OR23	AT4G03030	Protein synthesis, modificatio	1.85	2.00
ADP-ribosylation factor B1B	AT5G17060	Protein synthesis, modificatio	1.85	2.23
ubiquitin receptor protein DSK2B	AT2G17200	Protein synthesis, modificatio	1.84	2.07
papain family cysteine protease	AT4G16190	Protein synthesis, modificatio	1.83	2.27
plant organelle RNA recognition domain-containing protein	AT3G58520	Protein synthesis, modificatio	-1.88	-2.03
casein kinase I-like 12	AT5G57015	Protein synthesis, modificatio	-1.96	-2.05
uncharacterized protein	AT1G09070	Protein synthesis, modificatio	-2.00	-
Matrixin family protein	AT1G59970	Protein synthesis, modificatio	-2.00	-2.02
uncharacterized protein	AT1G67700	Protein synthesis, modificatio	-2.02	-2.00
Co-chaperone GrpE family protein	AT1G36390	Protein synthesis, modificatio	-2.03	-2.05
ubiquitin-specific protease family C19-related protein	AT1G16860	Protein synthesis, modificatio	-2.06	-2.15
50S ribosomal protein L13	AT1G78630	Protein synthesis, modificatio	-2.09	-2.13
ribosomal L18p/L5e family protein	AT5G27820	Protein synthesis, modificatio	-2.12	-
peptidyl-prolyl cis-trans isomerase CYP20-2	AT5G13120	Protein synthesis, modificatio	-2.13	-1.99
FKBP-type peptidyl-prolyl cis-trans isomerase 5	AT1G18170	Protein synthesis, modificatio	-2.14	-2.35
50S ribosomal protein L1	AT3G63490	Protein synthesis, modificatio	-2.19	-1.89
cysteine proteinase-like protein	AT3G02070	Protein synthesis, modificatio	-2.19	-2.12
translation elongation factor EF1B/ribosomal protein S6 family protein	AT1G64510	Protein synthesis, modificatio	-2.21	-2.27

p-Value > 0,05 - Fold change 2

ARM repeat superfamily protein	AT1G67530	Protein synthesis, modificatio	-2.23	-1.80
30S ribosomal protein S10-like	AT3G13120	Protein synthesis, modificatio	-2.23	-1.96
serine carboxypeptidase-like 25	AT3G02110	Protein synthesis, modificatio	-2.25	-
protein phosphatase 2C 58	AT4G28400	Protein synthesis, modificatio	-2.26	-2.23
peptidyl-tRNA hydrolase family protein	AT5G38290	Protein synthesis, modificatio	-2.27	-2.43
30S ribosomal protein S5	AT2G33800	Protein synthesis, modificatio	-2.28	-2.09
30S ribosomal protein S10	AT3G13120	Protein synthesis, modificatio	-2.28	-2.18
plastid-specific 50S ribosomal protein 6	AT5G17870	Protein synthesis, modificatio	-2.29	-
50S ribosomal protein L3-1	AT2G43030	Protein synthesis, modificatio	-2.30	-2.18
chaperonin-60 alpha	AT2G28000	Protein synthesis, modificatio	-2.33	-2.47
ribosomal protein L35	AT2G24090	Protein synthesis, modificatio	-2.35	-2.31
50S ribosomal protein L24	AT5G54600	Protein synthesis, modificatio	-2.37	-
30S ribosomal protein 3-1	AT1G68590	Protein synthesis, modificatio	-2.40	-2.19
ubiquitin-conjugating enzyme E2 20	AT1G50490	Protein synthesis, modificatio	-2.41	-
ER lumen protein retaining receptor-like protein	AT1G19970	Protein synthesis, modificatio	-2.44	-2.77
50S ribosomal protein L28	AT2G33450	Protein synthesis, modificatio	-2.45	-2.22
peptidyl-prolyl cis-trans isomerase FKBP16-3	AT2G43560	Protein synthesis, modificatio	-2.46	-2.32
50S ribosomal protein L5	AT4G01310	Protein synthesis, modificatio	-2.47	-2.30
FtsH extracellular protease	AT4G23940	Protein synthesis, modificatio	-2.51	-2.63
50S ribosomal protein related protein	AT5G16200	Protein synthesis, modificatio	-2.52	-3.34
30S ribosomal protein S20	AT3G15190	Protein synthesis, modificatio	-2.54	-
aspartyl protease family protein	AT3G54400	Protein synthesis, modificatio	-2.56	-2.80
photosystem II stability/assembly factor HCF136	AT5G23120	Protein synthesis, modificatio	-2.57	-2.29
protein disulfide-isomerase LQY1	AT1G75690	Protein synthesis, modificatio	-2.64	-2.24
peptidyl-prolyl cis-trans isomerase FKBP16-4	AT3G10060	Protein synthesis, modificatio	-2.64	-
S2P-like metalloprotease	AT5G05740	Protein synthesis, modificatio	-2.65	-2.75
FKBP-type peptidyl-prolyl cis-trans isomerase 3	AT5G45680	Protein synthesis, modificatio	-2.66	-2.75
F-box protein	AT1G78100	Protein synthesis, modificatio	-2.68	-2.95
50S ribosomal protein L34	AT1G29070	Protein synthesis, modificatio	-2.69	-2.42
50S ribosomal protein L21	AT1G35680	Protein synthesis, modificatio	-2.69	-2.61
plastid ribosomal protein S21	AT3G27160	Protein synthesis, modificatio	-2.70	-2.55
chaperonin 60 subunit beta 1	AT1G55490	Protein synthesis, modificatio	-2.80	-
peptidyl-prolyl cis-trans isomerase FKBP17-3	AT1G73655	Protein synthesis, modificatio	-2.83	-2.56
RING/U-box domain-containing protein	AT1G45180	Protein synthesis, modificatio	-2.84	-3.29
50S ribosomal protein L19-2	AT5G47190	Protein synthesis, modificatio	-2.94	-2.94
peptidyl-prolyl cis-trans isomerase CYP20-3	AT3G62030	Protein synthesis, modificatio	-2.95	-
Hypersensitive-induced response protein 2	AT1G69840	Protein synthesis, modificatio	-3.01	-2.94
aspartyl protease family protein	AT1G66180	Protein synthesis, modificatio	-3.34	-5.26
U-box domain-containing protein 31	AT5G65920	Protein synthesis, modificatio	-3.43	-3.66
peptidyl-prolyl cis-trans isomerase CYP37	AT3G15520	Protein synthesis, modificatio	-3.47	-3.39
ARM repeat superfamily protein	AT1G23030	Protein synthesis, modificatio	-3.52	-5.22
palmitoyl protein thioesterase family protein	AT5G47330	Protein synthesis, modificatio	-4.04	-
membrane-anchored ubiquitin-fold protein 4	AT3G26980	Protein synthesis, modificatio	-4.17	-4.06
phenazine biosynthesis PhzC/PhzF family protein	AT4G02850	Protein synthesis, modificatio	-4.79	-4.87
serine protease inhibitor, Kazal-type family protein	AT4G01575	Protein synthesis, modificatio	-4.80	-6.66
RING-H2 finger protein ATL17	AT4G15975	Protein synthesis, modificatio	-5.09	-4.49
uncharacterized protein	AT3G59310	Protein synthesis, modificatio	-7.78	-10.88
chaperone DnaJ-domain containing protein	AT1G72416	Protein synthesis, modificatio	-9.26	-7.84
aspartyl protease family protein	AT4G16563	Protein synthesis, modificatio	-42.42	-31.68
AAA-type ATPase family protein	AT3G28510	Protein synthesis, modificatio	-	43.75
Subtilase 3.5	AT1G32940	Protein synthesis, modificatio	-	6.15
heat shock protein 70	AT5G02490	Protein synthesis, modificatio	-	5.54
metacaspase 2	AT4G25110	Protein synthesis, modificatio	-	5.47
RING finger domain-containing protein	AT1G14200	Protein synthesis, modificatio	-	3.85
C3HC4-type RING finger-containing protein	AT1G08050	Protein synthesis, modificatio	-	3.60
Protein kinase superfamily protein	AT1G66460	Protein synthesis, modificatio	-	3.42
RING/U-box domain-containing protein	AT1G26800	Protein synthesis, modificatio	-	3.26
F-box/kelch-repeat protein	AT1G15670	Protein synthesis, modificatio	-	3.18
RING-H2 finger protein	AT2G42350	Protein synthesis, modificatio	-	3.03
RNI-like superfamily protein	AT4G30640	Protein synthesis, modificatio	-	3.00
flavin-binding, kelch repeat, f box 1	AT1G68050	Protein synthesis, modificatio	-	2.87
E3 ubiquitin-protein ligase RING1	AT5G10380	Protein synthesis, modificatio	-	2.59
Polyubiquitin	AT4G05320	Protein synthesis, modificatio	-	2.59
beta-endo-N-acetyglucosaminidase	AT5G05460	Protein synthesis, modificatio	-	2.43
cysteine proteinase inhibitor 7	AT5G05110	Protein synthesis, modificatio	-	2.25
NEP1-interacting protein 2	AT2G17730	Protein synthesis, modificatio	-	2.24
PPPDE thiol peptidase family protein	AT3G07090	Protein synthesis, modificatio	-	2.13
peptidyl-prolyl cis-trans isomerase	AT4G17070	Protein synthesis, modificatio	-	2.04
RING/U-box domain-containing protein	AT1G55530	Protein synthesis, modificatio	-	2.03
aspartyl protease family protein	AT3G52500	Protein synthesis, modificatio	-	-2.00
Initiation factor eIF-4 gamma, MA3	AT4G30680	Protein synthesis, modificatio	-	-2.03

molecular chaperone Hsp40/DnaJ family protein	AT3G17830	Protein synthesis, modificatio	-	-2.06
RNA pseudourine synthase 6	AT4G21770	Protein synthesis, modificatio	-	-2.14
RING/U-box domain-containing protein	AT1G67856	Protein synthesis, modificatio	-	-2.48
F-box protein	AT3G47030	Protein synthesis, modificatio	-	-2.71
U-box domain-containing protein 6	AT1G24330	Protein synthesis, modificatio	-	-3.38
F-box/RNI-like superfamily protein	AT3G03030	Protein synthesis, modificatio	-	-3.78
RING-H2 finger protein ATL80	AT1G20823	Protein synthesis, modificatio	-	-4.13
serine carboxypeptidase-like 34	AT5G23210	Protein synthesis, modificatio	-	-4.14
RHOMBOID-like protein 7	AT4G23070	Protein synthesis, modificatio	-	-13.74
cysteine-rich receptor-like protein kinase 7	AT4G23150	Signaling	6.50	14.02
FLG22-induced receptor-like kinase 1 (FRK1)	AT2G19190	Signaling	58.47	170.99
PAR1 protein	AT5G52390	Signaling	16.76	9.81
inorganic pyrophosphatase 1	AT1G73010	Signaling	13.41	-
protein kinase-like protein	AT3G46280	Signaling	11.85	24.60
concanavalin A-like lectin kinase-like protein	AT5G06740	Signaling	11.30	13.96
glutamate receptor 1.2	AT5G48400	Signaling	10.98	9.92
glutamate receptor 2.5	AT5G11210	Signaling	10.30	10.97
G-type lectin S-receptor-like serine/threonine-protein kinase	AT1G61480	Signaling	6.82	7.28
Receptor-like protein kinase-related family protein	AT3G22060	Signaling	6.14	8.67
LRR receptor-like serine/threonine-protein kinase	AT1G51860	Signaling	6.02	8.99
glutamate receptor 2.9	AT2G29100	Signaling	5.57	10.89
cysteine-rich receptor-like protein kinase 37	AT4G04500	Signaling	5.27	6.93
calcium-binding protein CML43	AT5G44460	Signaling	5.22	3.98
Protein kinase family protein	AT5G38250	Signaling	4.76	6.36
cysteine-rich receptor-like protein kinase 13	AT4G23210	Signaling	4.73	6.29
Leucine-rich repeat transmembrane protein kinase	AT1G56120	Signaling	4.70	7.43
for hypothetical protein, clone: RAFL21-67-K19	AT1G29715	Signaling	4.21	4.91
protein MATERNAL EFFECT EMBRYO ARREST 62	AT5G45800	Signaling	4.06	3.01
receptor-like protein kinase	AT1G72540	Signaling	4.05	4.53
inactive leucine-rich repeat receptor-like protein kinase	AT1G66830	Signaling	4.05	5.64
cysteine-rich receptor-like protein kinase 36	AT4G04490	Signaling	4.04	7.80
Cysteine/Histidine-rich C1 domain family protein	AT2G21850	Signaling	4.02	4.41
wall-associated receptor kinase-like 16	AT3G25490	Signaling	4.01	-
concanavalin A-like lectin protein kinase family protein	AT3G08870	Signaling	3.83	4.82
plasmodesmata-located protein 5	AT1G70690	Signaling	3.67	4.29
SEC7-like guanine nucleotide exchange family protein	AT4G35380	Signaling	3.63	3.21
G-type lectin S-receptor-like serine/threonine-protein kinase	AT1G61550	Signaling	3.56	3.85
SEC14 family protein	AT4G35750	Signaling	3.46	3.65
concanavalin A-like lectin kinase-like protein	AT3G45330	Signaling	3.36	4.01
inactive receptor kinase	AT5G53320	Signaling	3.27	3.10
cysteine-rich receptor-like protein kinase 20	AT4G23280	Signaling	3.15	8.10
glutamate receptor 1.4	AT3G07520	Signaling	3.02	3.02
atypical dual-specificity phosphatase 4	AT4G03960	Signaling	3.00	2.60
Wall-associated receptor kinase	AT5G53110	Signaling	2.99	3.11
G-type lectin S-receptor-like serine/threonine-protein kinase	AT1G67520	Signaling	2.92	4.78
SNF1-related kinase	AT2G25090	Signaling	2.92	-
leucine-rich repeat protein kinase-like protein	AT5G07150	Signaling	2.79	2.55
protein IQ-DOMAIN 14	AT2G43680	Signaling	2.76	-
cysteine-rich receptor-like protein kinase 22	AT4G23300	Signaling	2.73	3.20
protein kinase family protein	AT3G61960	Signaling	2.68	2.97
receptor-like protein kinase ANXUR1	AT3G04690	Signaling	2.59	2.47
CBL-interacting protein kinase 5	AT5G10930	Signaling	2.42	2.32
U-box domain-containing protein kinase family protein	AT5G65500	Signaling	2.38	2.17
leucine-rich repeat (LRR) family protein	AT1G13910	Signaling	2.31	2.06
Cam-binding protein 60-like G	AT5G26920	Signaling	2.30	2.81
Histone H3 K4-specific methyltransferase SET7/9 family protein	AT4G17080	Signaling	2.22	2.01
receptor serine/threonine kinase	AT1G70250	Signaling	2.21	2.38
protein kinase	AT1G48490	Signaling	2.17	2.59
concanavalin A-like lectin kinase-like protein	AT5G59260	Signaling	2.16	1.77
purple acid phosphatase 22	AT3G52820	Signaling	2.14	-
WD40 domain-containing protein	AT5G42010	Signaling	2.13	3.71
receptor-like protein kinase 1	AT5G60900	Signaling	2.10	-
mitogen-activated protein kinase 1	AT1G10210	Signaling	2.09	2.17
calcineurin B-like protein 6	AT4G16350	Signaling	2.08	2.46
protein kinase family protein	AT5G42440	Signaling	2.08	2.68
protein kinase family protein	AT4G21366	Signaling	2.07	3.44
CBL-interacting serine/threonine-protein kinase 23	AT1G30270	Signaling	2.00	2.17
RabGAP/TBC domain-containing protein	AT5G54780	Signaling	1.99	2.29
leucine-rich repeat protein kinase-like protein	AT1G51790	Signaling	1.94	7.41
uncharacterized Rho GTPase-activating protein	AT5G61530	Signaling	1.93	2.25
cysteine-rich receptor-like protein kinase 3	AT1G70530	Signaling	1.88	2.40

serine/threonine protein kinase	AT1G66880	Signaling	1.87	2.97
protein kinase family protein	AT2G28940	Signaling	1.85	2.08
protein activator of spomin::LUC2	AT3G12890	Signaling	1.83	2.11
protein phosphatase 2C 48	AT3G55050	Signaling	-1.79	-2.11
Rac-like GTP-binding protein ARAC9	AT2G44690	Signaling	-1.85	-2.18
LysM-containing receptor-like kinase	AT2G33580	Signaling	-1.96	-2.29
leucine-rich receptor-like protein kinase family protein	AT2G25790	Signaling	-1.99	-2.77
protein-tyrosine phosphatase	AT3G44620	Signaling	-2.00	-1.95
protein kinase family protein	AT2G25220	Signaling	-2.02	-1.93
ROP guanine nucleotide exchange factor 5	AT5G05940	Signaling	-2.02	-1.76
Rho GTPase activating protein with PAK-box/P21-Rho-binding domain	AT4G03100	Signaling	-2.03	-
calcium-dependent protein kinase 4	AT4G09570	Signaling	-2.04	-1.85
protein kinase family protein	AT3G57120	Signaling	-2.05	-2.33
plasmodesmata-located protein 7	AT5G37660	Signaling	-2.05	-
LRR receptor-like serine/threonine-protein kinase	AT3G47570	Signaling	-2.06	-2.07
Rac-like GTP-binding protein ARAC5	AT1G75840	Signaling	-2.07	-2.22
GTP-binding protein	AT4G02790	Signaling	-2.09	-2.28
Leucine-rich repeat protein kinase family protein	AT4G23740	Signaling	-2.13	-2.27
proline-rich receptor-like protein kinase PERK15	AT1G52290	Signaling	-2.17	-2.42
HAD superfamily, subfamily IIIB acid phosphatase	AT4G29270	Signaling	-2.21	-
photolyase/blue-light receptor 2	AT2G47590	Signaling	-2.24	-
RabGAP/TBC domain-containing protein	AT4G27100	Signaling	-2.24	-2.43
leucine-rich receptor-like protein kinase	AT4G20940	Signaling	-2.27	-2.45
protein kinase family protein	AT3G09830	Signaling	-2.29	-
protein kinase family protein	AT5G40540	Signaling	-2.29	-2.12
cysteine-rich receptor-like protein kinase 42	AT5G40380	Signaling	-2.30	-2.23
receptor-like protein kinase	AT2G39360	Signaling	-2.31	-2.02
calmodulin binding protein-like protein	AT5G62570	Signaling	-2.37	-2.66
calmodulin-binding protein	AT1G27460	Signaling	-2.37	-
CBL-interacting protein kinase 2	AT5G07070	Signaling	-2.38	-2.48
protein IQ-domain 2	AT5G03040	Signaling	-2.39	-2.48
Protein kinase protein with tetratricopeptide repeat domain	AT1G63500	Signaling	-2.41	-2.07
protein kinase family protein	AT1G33770	Signaling	-2.42	-2.93
mitogen-activated protein kinase 3	AT3G45640	Signaling	-2.45	-2.45
calcium-binding endonuclease/exonuclease/phosphatase family protein	AT1G02270	Signaling	-2.50	-3.10
mitogen-activated protein kinase 13	AT1G07880	Signaling	-2.53	-2.16
leucine-rich receptor-like protein kinase	AT1G72180	Signaling	-2.53	-2.96
protein phosphatase 2C 47	AT3G51470	Signaling	-2.54	-2.47
serine/threonine-protein kinase aurora-2	AT2G25880	Signaling	-2.55	-
protein kinase superfamily protein	AT3G61080	Signaling	-2.56	-
protein phosphatase 2C 5	AT1G09160	Signaling	-2.60	-3.17
protein TIC 62	AT3G18890	Signaling	-2.62	-2.29
serine/threonine-protein kinase	AT1G01540	Signaling	-2.64	-2.75
calmodulin 2	AT2G41110	Signaling	-2.66	-2.49
protein ralf-like 24	AT3G23805	Signaling	-2.67	-
leucine-rich repeat protein 1	AT5G16590	Signaling	-2.68	-2.94
GTP1/OBG family protein	AT5G18570	Signaling	-2.72	-2.73
copine-like protein BONZAI 1	AT5G61900	Signaling	-2.74	-2.55
GTP-binding protein	AT1G56050	Signaling	-2.78	-2.99
phosphatidylinositol-4-phosphate 5-kinase 6	AT3G07960	Signaling	-2.80	-2.93
calcium dependent protein kinase 1	AT5G04870	Signaling	-2.85	-2.47
mechanosensitive channel of small conductance-like 6	AT1G78610	Signaling	-2.90	-3.16
leucine-rich repeat protein kinase-like protein	AT3G23750	Signaling	-2.90	-3.74
leucine-rich repeat (LRR) family protein	AT1G33610	Signaling	-3.01	-3.78
protein kinase-like protein	AT5G61570	Signaling	-3.10	-3.79
cysteine-rich receptor-like protein kinase 10	AT4G23180	Signaling	-3.11	-3.05
protein IQ-domain 23	AT5G62070	Signaling	-3.12	-2.77
LRR receptor-like serine/threonine-protein kinase FLS2	AT5G46330	Signaling	-3.16	-2.33
HAD superfamily, subfamily IIIB acid phosphatase	AT1G04040	Signaling	-3.25	-2.77
calmodulin-like protein 11	AT3G22930	Signaling	-3.29	-
phosphoinositide phospholipase C 7	AT3G55940	Signaling	-3.44	-4.69
protein phosphatase 2C 12	AT1G47380	Signaling	-3.53	-4.85
serine/threonine kinase	AT2G31010	Signaling	-3.73	-4.48
RAB GTPase-like protein 7A	AT2G21880	Signaling	-3.81	-
mitogen-activated protein kinase kinase 6	AT5G56580	Signaling	-3.98	-3.23
transducin/WD40 domain-containing protein	AT5G53500	Signaling	-4.15	-4.74
calcium-dependent lipid-binding domain-containing protein	AT4G34150	Signaling	-4.16	-4.07
calcium-binding protein CML40	AT3G01830	Signaling	-4.20	-3.64
Remorin family protein	AT5G23750	Signaling	-4.36	-4.97
Remorin family protein	AT2G02170	Signaling	-4.40	-4.33
protein EXORDIUM like 2	AT5G64260	Signaling	-4.46	-4.80

leucine-rich repeat protein kinase family protein	AT1G51805	Signaling	-4.69	-
phosphoglucan phosphatase LSF2	AT3G10940	Signaling	-4.79	-4.24
serine/threonine kinase	AT1G74330	Signaling	-4.92	-5.14
receptor-like protein kinase THESEUS 1	AT5G54380	Signaling	-4.97	-
calcium-dependent protein kinase 32	AT3G57530	Signaling	-5.43	-5.47
cysteine-rich receptor-like protein kinase 29	AT4G21410	Signaling	-5.44	-4.10
calcium-dependent protein kinase 28	AT5G66210	Signaling	-5.64	-5.77
PTI1-like tyrosine-protein kinase 3	AT3G59350	Signaling	-5.87	-6.71
calcium-binding protein CML24	AT5G37770	Signaling	-5.90	-9.56
calcium-binding protein CML42	AT4G20780	Signaling	-5.92	-
leucine-rich repeat protein kinase family protein	AT1G51805	Signaling	-6.31	-4.36
cysteine-rich receptor-like protein kinase 41	AT4G00970	Signaling	-7.59	-6.31
Calmodulin-binding protein	AT4G31000	Signaling	-10.17	-11.03
phosphate-responsive 1 family protein	AT4G08950	Signaling	-20.16	-28.19
glutamate receptor 1.3	AT5G48410	Signaling	-	15.60
leucine-rich repeat protein kinase	AT1G51800	Signaling	-	10.57
receptor like protein 20	AT2G25440	Signaling	-	8.57
Leucine-rich repeat protein kinase family protein	AT1G51850	Signaling	-	7.77
purple acid phosphatase 17	AT3G17790	Signaling	-	6.93
leucine-rich repeat protein kinase family protein	AT5G59680	Signaling	-	4.52
cysteine-rich receptor-like protein kinase 38	AT4G04510	Signaling	-	4.23
Purple acid phosphatases superfamily protein	AT1G13750	Signaling	-	3.89
Lectin-domain containing receptor kinase A4.3	AT5G01560	Signaling	-	3.81
cysteine-rich receptor-like protein kinase 6	AT4G23140	Signaling	-	3.59
protein kinase family protein	AT4G11890	Signaling	-	3.47
serine/threonine-protein kinase WNK11	AT5G55560	Signaling	-	3.44
leucine-rich repeat protein kinase-like protein	AT4G39270	Signaling	-	3.20
serine/threonine protein kinase	AT1G66920	Signaling	-	2.67
GTP-binding protein Obg/CgtA	AT1G07620	Signaling	-	2.53
cysteine/histidine-rich C1 domain-containing protein	AT2G19650	Signaling	-	2.50
uncharacterized protein	AT4G16670	Signaling	-	2.40
calmodulin-binding receptor-like cytoplasmic kinase 1	AT5G58940	Signaling	-	2.32
CBL-interacting serine/threonine-protein kinase 21	AT5G57630	Signaling	-	2.21
G-type lectin S-receptor-like serine/threonine-protein kinase	AT5G35370	Signaling	-	2.18
wall-associated receptor kinase-like 8	AT1G16260	Signaling	-	2.16
LRR receptor-like serine/threonine-protein kinase	AT1G67720	Signaling	-	-2.00
protein phosphatase 2C 63	AT4G33920	Signaling	-	-2.02
protein kinase family protein	AT3G58690	Signaling	-	-2.04
LRR receptor-like serine/threonine-protein kinase	AT2G16250	Signaling	-	-2.08
CBL-interacting protein kinase	AT4G14580	Signaling	-	-2.10
concanavalin A-like lectin kinase-like protein	AT3G45430	Signaling	-	-2.11
Ras-related protein RABA6b	AT1G18200	Signaling	-	-2.20
serine/threonine-protein kinase RLCKVII	AT1G07870	Signaling	-	-2.21
PRA1 family protein B6	AT5G07110	Signaling	-	-2.29
kinase interacting (KIP1-like) protein	AT1G03470	Signaling	-	-2.32
calmodulin-like protein MSS3	AT2G43290	Signaling	-	-2.45
protein kinase family protein	AT1G70740	Signaling	-	-2.50
purple acid phosphatase 3	AT1G14700	Signaling	-	-2.65
CBL-interacting serine/threonine-protein kinase 15	AT5G01810	Signaling	-	-2.65
remorin-like protein	AT1G45207	Signaling	-	-2.65
G-type lectin S-receptor-like serine/threonine-protein kinase	AT1G61460	Signaling	-	-2.66
plasmodesmata-located protein 3	AT2G33330	Signaling	-	-2.76
CBL-interacting serine/threonine-protein kinase 9	AT1G01140	Signaling	-	-2.95
BTB/POZ domain-containing protein DOT3	AT5G10250	Signaling	-	-3.73
protein phosphatase 2C 25	AT2G30020	Signaling	-	-4.16
protein phosphatase 2C-like protein	AT2G05050	Signaling	-	-4.34
uncharacterized protein	AT2G39530	Transport	27.98	32.65
cation/H(+) antiporter 17	AT4G23700	Transport	25.75	36.72
amino acid transporter 1	AT4G21120	Transport	15.70	22.99
glucose-6-phosphate/phosphate translocator 2	AT1G61800	Transport	8.99	8.52
MATE efflux family protein	AT2G04100	Transport	8.28	9.99
ADP/ATP carrier 3 protein	AT4G28390	Transport	6.61	8.59
copper transport family protein	AT5G52720	Transport	5.87	6.15
inositol transporter 2	AT1G30220	Transport	5.55	4.01
auxin efflux carrier family protein	AT2G17500	Transport	5.47	-
exocyst subunit exo70 family protein H1	AT3G55150	Transport	4.44	7.30
urea-proton symporter DUR3	AT5G45380	Transport	4.42	7.19
inorganic phosphate transporter 1-1	AT5G43350	Transport	4.39	-
inorganic phosphate transporter 1-4	AT2G38940	Transport	3.89	-
vesicle-associated protein 3-1	AT2G23830	Transport	3.80	3.19
MATE efflux family protein	AT2G04070	Transport	3.48	5.73

multidrug resistance-associated protein 8	AT3G13090	Transport	3.46	3.77
cation/H(+) antiporter 16	AT1G64170	Transport	3.39	6.71
peptide/nitrate transporter	AT5G14940	Transport	3.35	3.08
ABC transporter A family member 7	AT3G47780	Transport	3.32	2.90
Sec14p-like phosphatidylinositol transfer family protein	AT1G75170	Transport	3.12	3.18
ABC transporter C family member 7	AT3G13100	Transport	2.95	3.62
MATE efflux family protein	AT1G71140	Transport	2.88	-
potassium transporter 6	AT1G70300	Transport	2.88	3.32
aromatic and neutral transporter 1	AT3G11900	Transport	2.84	3.14
copper transport protein	AT4G05030	Transport	2.80	3.98
copper-transporting ATPase HMA5	AT1G63440	Transport	2.75	-
ABC transporter G family member 18	AT3G55110	Transport	2.67	3.23
sugar transport protein 13	AT5G26340	Transport	2.57	4.34
plasma-membrane choline transporter family protein	AT3G03700	Transport	2.56	2.23
MATE efflux family protein	AT3G21690	Transport	2.55	2.39
sugar transporter ERD6-like 17	AT5G27350	Transport	2.54	-
ABC transporter C family member 4	AT2G47800	Transport	2.51	2.71
TRAF-like family protein	AT5G26280	Transport	2.50	2.86
auxin efflux transmembrane transporter MDR4	AT2G47000	Transport	2.35	2.36
zinc transporter ZIP2 - like protein	AT1G55910	Transport	2.33	-
ABC transporter G family member 31	AT2G29940	Transport	2.32	-
zinc transporter	AT3G08650	Transport	2.25	2.16
cation/H(+) antiporter 20	AT3G53720	Transport	2.24	2.50
clathrin assembly protein	AT5G35200	Transport	2.16	2.76
phospholipid-transporting ATPase 12	AT1G26130	Transport	2.08	2.82
SNARE-like family protein	AT4G27840	Transport	2.07	-
phospholipid-transporting ATPase 1	AT5G04930	Transport	2.05	2.51
H(+)-ATPase 2	AT4G30190	Transport	2.02	2.38
MATE efflux family protein	AT3G26590	Transport	1.99	2.39
cationic amino acid transporter 2	AT1G58030	Transport	1.99	1.65
sodium/metabolite cotransporter BASS6	AT4G22840	Transport	1.90	2.11
transmembrane emp24 domain-containing protein	AT1G26690	Transport	1.85	2.29
vesicle-associated protein 4-1	AT5G54110	Transport	1.85	2.17
aquaporin TIP2-1	AT3G16240	Transport	-1.88	-1.99
envelope ADP,ATP carrier protein	AT3G51870	Transport	-1.90	-2.58
K efflux antiporter KEA1	AT1G01790	Transport	-2.03	-1.90
nucleotide-sugar transporter	AT2G43240	Transport	-2.05	-2.21
SNARE associated Golgi family protein	AT1G12450	Transport	-2.07	-2.56
transmembrane amino acid transporter family protein	AT5G41800	Transport	-2.09	-
cationic amino acid transporter 7	AT3G10600	Transport	-2.10	-
aluminum activated malate transporter family protein	AT5G46600	Transport	-2.16	-1.88
ABC transporter G family member 27	AT3G52310	Transport	-2.18	-2.24
exocyst subunit exo70 family protein B1	AT5G58430	Transport	-2.21	-2.48
patellin-5	AT4G09160	Transport	-2.23	-2.11
peptide transporter PTR5	AT5G01180	Transport	-2.24	-
nucleotide-sugar transporter family protein	AT5G04160	Transport	-2.34	-2.28
golgi nucleotide sugar transporter 3	AT1G76340	Transport	-2.38	-2.50
cyclic nucleotide-gated channel 15	AT2G28260	Transport	-2.42	-
ABC transporter B family member 21	AT3G62150	Transport	-2.52	-1.49
peptide/nitrate transporter	AT1G33440	Transport	-2.55	-2.79
nucleotide-sugar transporter-like protein	AT1G21070	Transport	-2.62	-2.60
Sodium/calcium exchanger family protein	AT5G17850	Transport	-2.74	-4.07
Sec14p-like phosphatidylinositol transfer protein patellin-4	AT1G30690	Transport	-2.76	-2.79
peptide/nitrate transporter	AT5G13400	Transport	-2.81	-2.75
sec14p-like phosphatidylinositol transfer-like protein	AT5G56160	Transport	-2.84	-3.87
sugar transporter ERD6-like 4	AT1G19450	Transport	-2.91	-4.36
inorganic carbon transport protein-related protein	AT1G70760	Transport	-2.97	-
phosphatidylinositol transfer protein COW1	AT4G34580	Transport	-3.13	-
phosphoenolpyruvate (pep)/phosphate translocator 2	AT3G01550	Transport	-3.14	-
amino acid permease 5	AT1G44100	Transport	-3.20	-2.74
sugar transporter ERD6	AT1G08930	Transport	-3.20	-2.77
aluminum-activated, malate transporter 12	AT4G17970	Transport	-3.29	-2.91
hydrolase-like protein	AT5G17670	Transport	-3.35	-3.11
sulfate transporter 2;1	AT5G10180	Transport	-3.36	-
phosphate transporter PHO1-8	AT1G35350	Transport	-3.61	-3.10
nucleotide-sugar transporter family protein	AT4G18205	Transport	-3.64	-2.93
aquaporin TIP1-1	AT2G36830	Transport	-3.65	-3.01
MATE efflux family protein	AT1G15150	Transport	-3.70	-3.03
phosphate transporter PHO1-7	AT1G26730	Transport	-3.78	-4.33
MATE efflux family protein	AT5G17700	Transport	-3.84	-
aquaporin TIP1-1	AT2G36830	Transport	-3.98	-3.39

autoinhibited Ca2+-ATPase 1	AT1G27770	Transport	-4.09	-3.87
exocyst subunit exo70 family protein H7	AT5G59730	Transport	-4.47	-5.35
glucosinolate transporter 1	AT3G47960	Transport	-5.33	-5.79
cyclic nucleotide-gated channel 14	AT2G24610	Transport	-5.96	-5.45
purine permease 18	AT1G57990	Transport	-7.95	-
MATE efflux family protein	AT1G61890	Transport	-10.63	-15.97
MATE efflux family protein	AT5G52050	Transport	-12.52	-18.91
phosphate transporter 3;2	AT3G48850	Transport	-	13.84
ABC transporter-like protein	AT3G21080	Transport	-	8.14
ALA-interacting subunit 5	AT1G79450	Transport	-	6.71
calcium exchanger 7	AT5G17860	Transport	-	6.16
tc|GB|AC004218.3|AAC27844.1 unknown protein	AT2G39530	Transport	-	5.58
cation/H(+) antiporter 2	AT1G79400	Transport	-	5.41
proton pump interactor 2	AT3G15340	Transport	-	3.83
Lysine histidine transporter 1	AT5G40780	Transport	-	3.05
sec14p-like phosphatidylinositol transfer protein	AT4G36640	Transport	-	2.87
sugar transport protein 3	AT5G61520	Transport	-	2.56
ATPase 1	AT4G30190	Transport	-	2.28
transducin/WD40 domain-containing protein	AT3G62770	Transport	-	2.00
K+ uptake permease 9	AT4G19960	Transport	-	-2.22
peptide/nitrate transporter	AT2G40460	Transport	-	-2.60
peptide/nitrate transporter	AT1G22550	Transport	-	-2.91
peptide/nitrate transporter	AT1G22570	Transport	-	-3.07
purine permease 14	AT1G19770	Transport	-	-4.14
EXS (ERD1/XPR1/SYG1) family protein	AT2G03240	Transport	-	-4.92
organic cation/carnitine transporter 6	AT1G16370	Transport	-	-11.05
FAD-binding Berberine family protein	AT1G26390	Unknown	104.75	143.52
uncharacterized protein	AT1G13520	Unknown	47.75	54.94
cytochrome P450, family 82, subfamily C, polypeptide 2	AT4G31970	Unknown	45.42	41.65
plant invertase/pectin methylesterase inhibitor domain-containing protein	AT5G46960	Unknown	44.83	18.44
germin-like protein subfamily 1 member 8	AT4G14630	Unknown	42.21	62.13
uncharacterized protein	AT3G55790	Unknown	36.72	24.68
membrane lipoprotein	AT3G18250	Unknown	33.90	66.29
invertase/pectin methylesterase inhibitor family protein	AT5G46950	Unknown	27.29	8.84
uncharacterized protein	AT2G39518	Unknown	25.43	33.98
uncharacterized protein	AT5G37840	Unknown	22.81	15.69
uncharacterized protein	AT3G19615	Unknown	22.74	20.16
FAD-binding Berberine family protein	AT1G30700	Unknown	20.95	35.33
Cupredoxin superfamily protein	AT2G15780	Unknown	20.42	16.93
Adenine nucleotide alpha hydrolases-like superfamily protein	AT5G47740	Unknown	20.31	17.20
cytochrome P450, family 82, subfamily C, polypeptide 3	AT4G31950	Unknown	19.77	15.32
PLAC8 family protein	AT1G68630	Unknown	17.81	14.84
SPFH/Band 7/PHB domain-containing membrane-associated protein	AT5G25260	Unknown	17.15	30.54
beta glucosidase 46	AT1G61820	Unknown	16.76	12.63
uncharacterized protein	AT1G36640	Unknown	15.65	20.38
uncharacterized protein	AT3G15536	Unknown	12.62	10.70
uncharacterized protein	AT1G15385	Unknown	12.60	8.69
uncharacterized protein	AT5G44575	Unknown	12.57	27.90
uncharacterized protein	AT1G53625	Unknown	12.33	16.08
beta glucosidase 27	AT3G60120	Unknown	12.04	-
clone 155459 sequence	AT1G55525	Unknown	11.73	-
phloem protein 2-A7	AT5G45090	Unknown	11.38	-
uncharacterized protein	AT3G13950	Unknown	10.78	17.59
uncharacterized protein	AT4G11655	Unknown	10.57	8.67
plant invertase/pectin methylesterase inhibitor domain-containing protein	AT1G62760	Unknown	10.13	9.33
pollen Ole e 1 allergen and extensin family protein	AT4G17215	Unknown	9.90	14.95
O-Glycosyl hydrolases family 17 protein	AT5G64790	Unknown	9.31	6.78
VQ motif-containing protein	AT4G20000	Unknown	9.11	17.23
uncharacterized protein	AT1G36622	Unknown	8.48	14.90
polynucleotidyl transferase, ribonuclease H-like superfamily protein	AT3G12470	Unknown	8.32	5.42
glycosyl hydrolase family protein 17	AT5G63225	Unknown	8.29	6.12
germin-like protein 6	AT5G39100	Unknown	8.23	5.47
uncharacterized protein	AT3G61198	Unknown	7.88	4.93
uncharacterized protein	AT3G26440	Unknown	7.86	12.56
late embryogenesis abundant (LEA) hydroxyproline-rich glycoprotein	AT1G65690	Unknown	7.72	14.36
uncharacterized protein	AT2G45360	Unknown	7.66	12.66
uncharacterized protein	AT1G65481	Unknown	7.59	10.43
uncharacterized protein	AT1G53620	Unknown	7.34	6.01
uncharacterized protein	AT3G26440	Unknown	7.29	12.98
cytochrome P450, family 714, subfamily A, polypeptide 1	AT5G24910	Unknown	7.07	5.06
serine-rich protein-like protein	AT3G56500	Unknown	6.77	6.64

uncharacterized protein	AT2G21185	Unknown	6.58	8.16
uncharacterized protein	AT2G29430	Unknown	6.26	-
AAA-type ATPase family protein	AT3G28540	Unknown	6.13	14.58
uncharacterized protein	AT4G28460	Unknown	6.07	6.78
alpha/beta-Hydrolases superfamily protein	AT1G08310	Unknown	5.83	5.57
cytochrome P450 71B6	AT2G24180	Unknown	5.81	6.66
pyruvate kinase-like protein	AT3G49160	Unknown	5.80	-
uncharacterized protein	AT3G29240	Unknown	5.66	3.21
for hypothetical protein, clone: RAFL22-67-I17	AT5G01732	Unknown	5.61	7.41
uncharacterized protein	AT5G44585	Unknown	5.57	13.00
heat stress transcription factor B-1	AT4G36990	Unknown	5.52	6.98
uncharacterized protein	AT1G65500	Unknown	5.43	9.26
carbohydrate-binding X8 domain-containing protein	AT2G03505	Unknown	5.32	5.32
pollen Ole e 1 allergen and extensin family protein	AT2G40113	Unknown	5.29	6.43
uncharacterized protein	AT3G57950	Unknown	5.24	4.81
heavy-metal-associated domain-containing protein	AT5G26690	Unknown	5.19	9.42
phosphorylase superfamily protein	AT4G28940	Unknown	5.19	3.54
VQ motif-containing protein	AT4G15120	Unknown	5.12	4.34
uncharacterized protein	AT3G29240	Unknown	5.10	3.09
uncharacterized protein	AT1G10140	Unknown	4.98	5.50
Endosomal targeting BRO1-like domain-containing protein	AT1G13310	Unknown	4.96	3.40
uncharacterized protein	AT4G37900	Unknown	4.92	4.11
uncharacterized protein	AT5G44572	Unknown	4.86	8.26
cryptdin-related protein	AT1G51915	Unknown	4.85	5.13
uncharacterized protein	AT5G03230	Unknown	4.83	4.02
uncharacterized protein	AT3G14280	Unknown	4.82	7.02
uncharacterized protein	AT3G15760	Unknown	4.80	3.60
Ninja-family protein AFP3	AT3G29575	Unknown	4.79	3.29
cytochrome P450 71B2	AT1G13080	Unknown	4.68	5.72
uncharacterized protein	AT4G19970	Unknown	4.65	4.16
uncharacterized protein	AT3G13435	Unknown	4.60	3.99
Wound-responsive family protein	AT4G05070	Unknown	4.59	4.88
uncharacterized protein	AT3G07350	Unknown	4.43	-
uncharacterized protein	AT1G13480	Unknown	4.42	4.63
Ninja-family protein AFP3	AT3G29575	Unknown	4.41	3.22
uncharacterized protein	AT1G51913	Unknown	4.37	4.93
cytochrome P450, family 81, subfamily H, polypeptide 1	AT4G37310	Unknown	4.36	3.70
uncharacterized protein	AT1G49470	Unknown	4.35	3.82
pyridoxal phosphate phosphatase-related protein	AT1G17710	Unknown	4.34	3.43
SRPBCC ligand-binding domain-containing protein	AT4G32870	Unknown	4.07	4.96
uncharacterized protein	AT5G22270	Unknown	4.05	3.43
uncharacterized protein	AT4G34630	Unknown	4.02	3.90
uncharacterized protein	AT5G40720	Unknown	3.96	3.41
uncharacterized protein	AT5G02020	Unknown	3.89	3.44
uncharacterized protein	AT1G23560	Unknown	3.88	-
D-3-phosphoglycerate dehydrogenase	AT1G17745	Unknown	3.79	4.34
cytochrome P450, family 89, subfamily A, polypeptide 5	AT1G64950	Unknown	3.78	2.86
cytochrome P450 monooxygenase	AT3G26220	Unknown	3.76	5.47
Methyltransferase-related protein	AT5G58375	Unknown	3.68	3.99
uncharacerized protein	AT4G36988	Unknown	3.65	4.63
TRAF-like family protein	AT3G46190	Unknown	3.62	3.40
gunnii alcohol dehydrogenase-like protein	AT1G09480	Unknown	3.61	4.13
uncharacterized protein	AT3G15534	Unknown	3.61	3.07
Mo25 family protein	AT2G03410	Unknown	3.60	3.49
uncharacterized protein	AT4G25070	Unknown	3.56	5.04
core-2/I-branching beta-1,6-N-acetylglucosaminyltransferase family protein	AT1G68390	Unknown	3.45	3.87
calcium-dependent lipid-binding domain-containing protein	AT1G23140	Unknown	3.40	3.89
late embryogenesis abundant hydroxyproline-rich glycoprotein	AT4G35170	Unknown	3.40	3.01
cytochrome P450, family 87, subfamily A, polypeptide 6	AT2G12190	Unknown	3.39	2.92
Exostosin family protein	AT5G25820	Unknown	3.38	3.30
uncharacterized protein	AT3G25240	Unknown	3.37	-
UDP-glycosyltransferase 86A1	AT2G36970	Unknown	3.35	5.48
uncharacterized protein	AT1G13990	Unknown	3.33	3.97
copper amine oxidase family protein	AT4G12290	Unknown	3.30	4.19
uncharacterized protein	AT4G30230	Unknown	3.29	3.05
clone 119768 sequence	AT3G12502	Unknown	3.27	-
uncharacterized protein	AT2G27830	Unknown	3.26	2.39
lysophospholipase 1-like protein	AT2G39410	Unknown	3.24	2.89
formin-like protein 16	AT5G07770	Unknown	3.23	3.17
uncharacterized protein	AT4G19370	Unknown	3.18	3.90
cytochrome P450 89A2	AT1G64900	Unknown	3.13	3.41

TRAM, LAG1 and CLN8 (TLC) lipid-sensing domain containing protein	AT3G27270	Unknown	3.13	2.86
Flavin-binding monooxygenase family protein	AT1G12160	Unknown	3.10	2.81
uncharacterized protein	AT1G13530	Unknown	3.10	3.55
uncharacterized protein	AT1G10410	Unknown	3.09	2.85
uncharacterized protein	AT3G13432	Unknown	3.09	2.92
uncharacterized protein	AT4G39610	Unknown	3.07	2.66
uncharacterized protein	AT1G64405	Unknown	3.06	4.72
O-acyltransferase (WSD1-like) family protein	AT5G12420	Unknown	3.05	4.45
uncharacterized protein	AT3G52480	Unknown	3.04	4.06
uncharacterized protein	AT3G04700	Unknown	3.03	3.32
uncharacterized protein	AT5G60630	Unknown	3.02	-
RNI-like superfamily protein	AT5G45500	Unknown	3.00	3.15
uncharacterized protein	AT3G21710	Unknown	2.99	3.12
cytochrome P450, family 705, subfamily A, polypeptide 33	AT3G20960	Unknown	2.97	3.23
DnaJ domain-containing protein	AT3G08970	Unknown	2.97	4.17
uncharacterized protein	AT2G22320	Unknown	2.95	-
uncharacterized protein	AT1G68650	Unknown	2.94	2.73
uncharacterized protein	AT1G21050	Unknown	2.92	2.80
polyadenylate-binding protein-interacting protein 2	AT4G14270	Unknown	2.90	3.44
uncharacterized protein	AT1G71970	Unknown	2.89	3.05
protein DJ-1-like A	AT3G14990	Unknown	2.89	2.73
cytochrome P450 71B26	AT3G26290	Unknown	2.86	3.14
cytochrome P450, family 76, subfamily C, polypeptide 5	AT1G33730	Unknown	2.85	2.21
uncharacterized protein	AT1G13360	Unknown	2.83	3.31
uncharacterized protein	AT5G44578	Unknown	2.81	-
TRAM, LAG1 and CLN8 (TLC) lipid-sensing domain containing protein	AT4G19645	Unknown	2.79	2.27
uncharacterized protein	AT1G70160	Unknown	2.78	2.60
uncharacterized protein	AT1G71910	Unknown	2.77	3.31
Exostosin family protein	AT5G11610	Unknown	2.77	2.71
uncharacterized protein	AT3G19660	Unknown	2.69	4.21
leucine-rich repeat-containing protein	AT1G15740	Unknown	2.69	2.69
HXXXD-type acyl-transferase-like protein	AT4G15400	Unknown	2.68	2.69
uncharacterized protein	AT4G33666	Unknown	2.64	-
alpha/beta-Hydrolases superfamily protein	AT3G51000	Unknown	2.60	2.34
calcium-dependent lipid-binding domain-containing protein	AT3G55470	Unknown	2.60	3.99
uncharacterized protein	AT2G31160	Unknown	2.60	2.93
uncharacterized protein	AT5G65207	Unknown	2.59	-
uncharacterized protein	AT1G13550	Unknown	2.58	4.33
uncharacterized protein	AT3G03870	Unknown	2.57	2.99
for hypothetical protein, clone: RAFL21-14-O08	AT3G26165	Unknown	2.57	2.16
uncharacterized protein	AT3G13432	Unknown	2.56	2.47
cytochrome P450 71B24	AT3G26230	Unknown	2.55	3.72
Unknown		Unknown	2.54	2.68
cytochrome P450 71A22	AT3G48310	Unknown	2.53	2.83
uncharacterized protein	AT1G52855	Unknown	2.53	2.90
late embryogenesis abundant hydroxyproline-rich glycoprotein	AT2G46150	Unknown	2.52	4.32
uncharacterized protein	AT5G59400	Unknown	2.52	2.02
Agenet domain-containing protein	AT5G52070	Unknown	2.51	2.57
cytochrome P450 71B20	AT3G26180	Unknown	2.49	2.30
ARM repeat superfamily protein	AT3G03440	Unknown	2.49	2.54
HIPL2 protein	AT5G62630	Unknown	2.48	3.00
cytochrome P450, family 81, subfamily D, polypeptide 5	AT4G37320	Unknown	2.48	2.22
uncharacterized protein	AT5G01610	Unknown	2.47	2.69
uncharacterized protein	AT4G23885	Unknown	2.47	2.99
uncharacterized protein	AT5G47580	Unknown	2.44	2.69
uncharacterized protein	AT2G40390	Unknown	2.44	2.16
uncharacterized protein	AT2G28570	Unknown	2.43	3.02
cytochrome P450, family 704, subfamily A, polypeptide 2	AT2G45510	Unknown	2.43	3.27
uncharacterized protein	AT1G10690	Unknown	2.42	2.65
reticulon-like protein B9	AT3G18260	Unknown	2.42	2.21
clone asmbl_2021 unknown sequence	AT1G32172	Unknown	2.42	-
uncharacterized protein	AT3G27880	Unknown	2.42	2.25
Myosin heavy chain-related protein	AT4G40020	Unknown	2.39	3.53
calcium-dependent lipid-binding domain-containing protein	AT2G01540	Unknown	2.39	2.76
uncharacterized protein	AT1G65845	Unknown	2.39	5.14
uncharacterized protein	AT4G23880	Unknown	2.38	-
SART-1 family protein	AT3G14700	Unknown	2.38	2.32
Unknown		Unknown	2.36	2.75
Unknown		Unknown	2.34	3.20
uncharacterized protein	AT4G09830	Unknown	2.34	2.10
Per1-like family protein	AT5G62130	Unknown	2.33	-

uncharacterized protein	AT2G15695	Unknown	2.33	2.39
uncharacterized protein	AT3G10250	Unknown	2.30	2.25
ribosomal protein L18ae family protein	AT4G26060	Unknown	2.29	2.94
Rossmann-fold NAD(P)-binding domain-containing protein	AT4G09750	Unknown	2.29	2.35
uncharacterized protein	AT3G24927	Unknown	2.27	2.30
clone 7553 sequence	AT1G67328	Unknown	2.26	2.23
uncharacterized protein	AT1G15790	Unknown	2.25	3.14
uncharacterized protein	AT4G11860	Unknown	2.24	2.31
uncharacterized protein	AT5G62900	Unknown	2.24	1.92
uncharacterized protein	AT4G14620	Unknown	2.24	2.12
uncharacterized protein	AT5G57910	Unknown	2.20	2.22
uncharacterized protein	AT5G10946	Unknown	2.17	2.15
ARM repeat superfamily protein	AT3G51980	Unknown	2.17	2.36
cytochrome P450 71B11	AT5G25120	Unknown	2.17	2.09
uncharacterized protein	AT3G54880	Unknown	2.16	1.86
uncharacterized protein	AT4G11350	Unknown	2.16	2.24
Erythronate-4-phosphate dehydrogenase family protein	AT1G19400	Unknown	2.15	1.81
uncharacterized protein	AT1G01730	Unknown	2.15	2.22
uncharacterized protein	AT2G41660	Unknown	2.14	-
camphor resistance CrcB-like protein	AT2G41705	Unknown	2.12	2.16
uncharacterized protein	AT5G04790	Unknown	2.12	-
cyclic phosphodiesterase	AT4G18930	Unknown	2.11	2.65
Carbohydrate-binding protein	AT1G10150	Unknown	2.11	1.97
uncharacterized protein	AT1G08180	Unknown	2.09	2.53
uncharacterized protein	AT1G33050	Unknown	2.09	1.74
uncharacterized protein	AT5G22550	Unknown	2.09	-
uncharacterized protein	AT1G29240	Unknown	2.08	2.35
Mediator complex, subunit Med10	AT1G26665	Unknown	2.07	2.07
uncharacterized protein	AT1G65720	Unknown	2.06	2.11
uncharacterized protein	AT5G19860	Unknown	2.06	2.05
uncharacterized protein	AT5G49525	Unknown	2.06	-
ACT domain-containing protein	AT2G39570	Unknown	2.05	2.34
uncharacterized protein	AT4G06676	Unknown	2.04	1.90
Yippee family zinc-binding protein	AT2G40110	Unknown	2.03	2.21
cytochrome P450, family 705, subfamily A, polypeptide 28	AT3G20935	Unknown	2.03	-
uncharacterized protein	AT1G28070	Unknown	2.03	-
uncharacterized protein	AT2G10560	Unknown	2.02	2.78
protein narrow leaf 1-like protein	AT3G12950	Unknown	2.02	1.91
uncharacterized protein	AT5G44574	Unknown	2.01	2.38
aldehyde dehydrogenase 2C4	AT3G24503	Unknown	2.01	2.82
electron transfer flavoprotein subunit alpha	AT1G50940	Unknown	2.01	1.84
chaperone protein dnaJ 20	AT4G13830	Unknown	2.01	2.52
Erythronate-4-phosphate dehydrogenase family protein	AT1G75180	Unknown	2.01	1.47
uncharacterized protein	AT1G65985	Unknown	2.00	2.24
protein PHLOEM protein 2-LIKE A9	AT1G31200	Unknown	2.00	-
protein Fes1C	AT5G02150	Unknown	1.99	1.84
germin-like protein subfamily 1 member 18	AT5G39160	Unknown	1.98	2.11
for hypothetical protein, partial cds, clone: RAFL14-17-D06	AT2G31585	Unknown	1.94	2.10
cytochrome P450 71B4	AT3G26280	Unknown	1.94	2.18
germin-like protein subfamily 2 member 5	AT5G26700	Unknown	1.92	2.48
tudor-like RNA-binding protein	AT4G32440	Unknown	1.91	2.05
wound-responsive protein	AT1G19660	Unknown	1.91	2.02
CFIM-25-like protein	AT4G29820	Unknown	1.89	2.11
VQ motif-containing protein	AT4G39720	Unknown	1.87	2.15
uncharacterized protein	AT1G03290	Unknown	1.84	2.24
cytochrome c oxidase 19-1	AT1G66590	Unknown	1.84	2.04
heme oxygenase-like, multi-helical protein	AT3G16990	Unknown	1.83	2.57
uncharacterized protein	AT1G67850	Unknown	1.82	2.26
Aluminum activated malate transporter family protein	AT1G25480	Unknown	1.75	2.04
uncharacterized protein	AT5G53030	Unknown	1.72	2.13
uncharacterized protein	AT1G13540	Unknown	1.69	2.00
alpha/beta-Hydrolases superfamily protein	AT1G78210	Unknown	1.66	2.25
uncharacterized protein	AT4G36105	Unknown	-1.59	-2.02
uncharacterized protein	AT5G66740	Unknown	-1.66	-1.99
P-loop containing nucleoside triphosphate hydrolase family protein	AT4G34420	Unknown	-1.75	-2.07
alpha-L-fucosidase 2	AT4G34260	Unknown	-1.84	-2.40
Rhodanese/Cell cycle control phosphatase superfamily protein	AT3G25480	Unknown	-1.84	-2.13
uncharacterized protein	AT2G44640	Unknown	-1.84	-2.16
uncharacterized protein	AT1G65295	Unknown	-1.85	-2.05
branched-chain-amino-acid aminotransferase-like protein 1	AT3G05190	Unknown	-1.87	-2.30
uncharacterized protein	AT5G63040	Unknown	-1.92	-2.02

Uroporphyrinogen decarboxylase	AT2G40490	Unknown	-1.93	-2.11
uncharacterized protein	AT3G51220	Unknown	-1.94	-2.09
uncharacterized protein	AT5G57000	Unknown	-1.96	-2.13
uncharacterized protein	AT2G30990	Unknown	-1.97	-2.14
haloacid dehalogenase-like hydrolase domain-containing protein	AT3G58830	Unknown	-1.97	-2.10
dynamin-like protein ARC5	AT3G19720	Unknown	-1.98	-2.04
B-cell receptor-associated protein 31-like protein	AT3G07190	Unknown	-1.99	-1.88
uncharacterized protein	AT3G15480	Unknown	-2.00	-2.37
uncharacterized protein	AT2G21960	Unknown	-2.01	-1.87
uncharacterized protein	AT5G41100	Unknown	-2.01	-2.11
uncharacterized protein	AT2G25250	Unknown	-2.01	-2.11
uncharacterized protein	AT1G73020	Unknown	-2.02	-1.92
Vps4 regulator of MVB pathway	AT2G19710	Unknown	-2.02	-2.05
receptor like protein 51	AT4G18760	Unknown	-2.02	-
pentatricopeptide repeat-containing protein	AT5G01110	Unknown	-2.02	-1.94
uncharacterized protein	AT3G61840	Unknown	-2.03	-
uncharacterized protein	AT1G59865	Unknown	-2.03	-
mesophyll-cell RNAi library line 7-like protein	AT2G31840	Unknown	-2.03	-2.17
Unknown	AT3G53010	Unknown	-2.04	-2.13
uncharacterized protein	AT2G34670	Unknown	-2.04	-2.07
uncharacterized protein	AT5G50610	Unknown	-2.05	-1.92
cotton-Golgi related 3	AT5G65810	Unknown	-2.05	-1.98
uncharacterized protein	AT3G50340	Unknown	-2.05	-2.03
uncharacterized protein	AT5G27560	Unknown	-2.05	-1.90
uncharacterized protein	AT1G64700	Unknown	-2.08	-1.98
armadillo/beta-catenin-like repeat-containing protein	AT5G50900	Unknown	-2.09	-2.27
tetratricopeptide repeat domain-containing protein	AT3G47080	Unknown	-2.11	-2.60
uncharacterized protein	AT4G15790	Unknown	-2.15	-2.37
alpha/beta-hydrolase-like protein	AT1G29840	Unknown	-2.18	-2.48
double Clp-N motif-containing P-loop nucleoside triphosphate hydrolases superfamily protein	AT4G30350	Unknown	-2.19	-1.83
alpha/beta-Hydrolases superfamily protein	AT5G09430	Unknown	-2.20	-2.71
chromosome transmission fidelity protein 8 domain-containing protein	AT5G52220	Unknown	-2.21	-2.23
uncharacterized protein	AT5G64850	Unknown	-2.21	-2.77
uncharacterized protein	AT3G23170	Unknown	-2.22	-
NDH dependent flow 6 protein	AT1G18730	Unknown	-2.24	-
uncharacterized protein	AT5G42110	Unknown	-2.24	-2.42
uncharacterized protein	AT3G59880	Unknown	-2.25	-
methyltransferase	AT5G01710	Unknown	-2.25	-2.65
uncharacterized protein	AT3G28760	Unknown	-2.26	-2.42
uncharacterized protein	AT4G01150	Unknown	-2.26	-
uncharacterized protein	AT1G03730	Unknown	-2.27	-2.23
uncharacterized protein	AT5G66675	Unknown	-2.28	-2.64
uncharacterized protein	AT3G29280	Unknown	-2.28	-2.20
cold regulated 413 plasma membrane 1	AT2G15970	Unknown	-2.28	-2.12
uncharacterized protein	AT1G68430	Unknown	-2.30	-2.43
fringe-related protein	AT4G00300	Unknown	-2.30	-2.19
uncharacterized protein	AT5G11070	Unknown	-2.31	-2.92
tetratricopeptide repeat-containing protein	AT3G05625	Unknown	-2.32	-2.58
uncharacterized protein	AT1G01570	Unknown	-2.33	-2.77
myb domain protein 73	AT4G37260	Unknown	-2.33	-2.96
protein LUTEIN DEFICIENT 5	AT1G31800	Unknown	-2.33	-2.28
uncharacterized protein	AT4G30996	Unknown	-2.34	-2.71
uncharacterized protein	AT3G06150	Unknown	-2.34	-2.69
uncharacterized protein	AT2G43340	Unknown	-2.36	-2.38
uncharacterized protein	AT5G22390	Unknown	-2.37	-2.63
thylakoid lumenal protein 17.9	AT4G24930	Unknown	-2.38	-2.31
BSD domain-containing protein	AT1G69030	Unknown	-2.38	-1.70
uncharacterized protein	AT5G61412	Unknown	-2.39	-2.89
uncharacterized protein	AT4G09640	Unknown	-2.39	-2.97
major facilitator protein	AT2G16990	Unknown	-2.40	-3.26
uncharacterized protein	AT4G22830	Unknown	-2.40	-2.32
uncharacterized protein	AT3G53010	Unknown	-2.41	-2.61
uncharacterized protein	AT3G13275	Unknown	-2.41	-2.76
cytochrome P450 86A2	AT4G00360	Unknown	-2.42	-1.98
uncharacterized protein	AT5G48790	Unknown	-2.43	-
tetratricopeptide repeat-containing protein	AT1G78915	Unknown	-2.44	-
GTP-binding protein Era	AT5G66470	Unknown	-2.44	-2.50
uncharacterized protein	AT5G43880	Unknown	-2.44	-2.57
Rubber elongation factor protein	AT2G47780	Unknown	-2.45	-3.07
transmembrane protein 97	AT1G05210	Unknown	-2.45	-1.99
uncharacterized protein	AT5G03670	Unknown	-2.45	-2.27

uncharacterized protein	AT4G24175	Unknown	-2.50	-2.50
uncharacterized protein	AT1G68330	Unknown	-2.51	-
beta glucosidase 40	AT1G26560	Unknown	-2.51	-2.18
uncharacterized protein	AT1G10020	Unknown	-2.51	-3.16
calcium-dependent lipid-binding domain-containing protein	AT3G19830	Unknown	-2.52	-
uncharacterized protein	AT4G17000	Unknown	-2.54	-
uncharacterized protein	AT4G21570	Unknown	-2.54	-2.69
Rossmann-fold NAD(P)-binding domain-containing protein	AT4G20760	Unknown	-2.55	-2.38
uncharacterized protein	AT1G10522	Unknown	-2.56	-2.67
alpha/beta-Hydrolases superfamily protein	AT1G13820	Unknown	-2.57	-2.63
uncharacterized protein	AT5G48470	Unknown	-2.57	-2.45
uncharacterized protein	AT5G43950	Unknown	-2.58	-2.48
uncharacterized protein	AT2G38465	Unknown	-2.58	-
uncharacterized protein	AT5G62960	Unknown	-2.58	-3.05
uncharacterized protein	AT2G42110	Unknown	-2.59	-2.28
uncharacterized protein	AT3G07380	Unknown	-2.60	-3.06
uncharacterized protein	AT1G69890	Unknown	-2.62	-3.51
uncharacterized protein	AT1G27030	Unknown	-2.62	-3.39
uncharacterized protein	AT3G02640	Unknown	-2.64	-
uncharacterized protein	AT5G37360	Unknown	-2.66	-2.75
pollen Ole e 1 allergen and extensin family protein	AT2G16630	Unknown	-2.67	-
uncharacterized protein	AT2G36410	Unknown	-2.67	-2.95
transducin/WD40 repeat-like superfamily protein	AT1G78070	Unknown	-2.68	-3.62
dihydrodipicolinate reductase 1	AT2G44040	Unknown	-2.69	-3.11
methyltransferase	AT3G28460	Unknown	-2.70	-3.30
LORELEI-LIKE-GPI-ANCHORED PROTEIN 1	AT5G56170	Unknown	-2.71	-2.76
Unknown	AT5G01542	Unknown	-2.72	-3.19
hydrolase, alpha/beta fold family protein	AT4G12830	Unknown	-2.72	-2.72
uncharacterized protein	AT2G21120	Unknown	-2.74	-
polyketide cyclase / dehydrase and lipid transport protein	AT4G01883	Unknown	-2.74	-2.95
beta carbonic anhydrase 4	AT1G70410	Unknown	-2.77	-2.32
uncharacterized protein	AT5G11000	Unknown	-2.77	-
uncharacterized protein	AT2G28140	Unknown	-2.77	-2.90
uncharacterized protein	AT2G33400	Unknown	-2.77	-2.49
D-mannose binding lectin protein with Apple-like carbohydrate-binding domain	AT1G78820	Unknown	-2.78	-2.45
uncharacterized protein	AT3G55646	Unknown	-2.78	-
uncharacterized protein	AT4G24265	Unknown	-2.79	-
uncharacterized protein	AT1G69760	Unknown	-2.79	-2.28
uncharacterized protein	AT4G27350	Unknown	-2.81	-3.11
uncharacterized protein	AT2G20724	Unknown	-2.81	-2.99
uncharacterized protein	AT3G07460	Unknown	-2.81	-2.77
uncharacterized protein	AT5G44005	Unknown	-2.82	-2.60
plastidal glycolate/glycerate translocator 1	AT1G32080	Unknown	-2.82	-
uncharacterized protein	AT4G29400	Unknown	-2.82	-2.82
uncharacterized protein	AT3G01960	Unknown	-2.83	-3.26
magnesium chelatase subunit I2	AT5G45930	Unknown	-2.84	-2.94
uncharacterized protein	AT4G38545	Unknown	-2.85	-
uncharacterized protein	AT5G48310	Unknown	-2.85	-
uncharacterized protein	AT4G18540	Unknown	-2.85	-
heptahelical transmembrane protein1	AT5G20270	Unknown	-2.87	-2.63
uncharacterized protein	AT3G60990	Unknown	-2.87	-3.24
CASP-like protein	AT2G35760	Unknown	-2.88	-2.64
cadmium tolerance 1	AT1G52827	Unknown	-2.89	-
uncharacterized protein	AT1G75860	Unknown	-2.92	-2.59
thylakoid lumenal protein 2	AT5G52970	Unknown	-2.95	-2.81
uncharacterized protein	AT1G53633	Unknown	-2.95	-
cytokine-induced anti-apoptosis inhibitor 1, Fe-S biogenesis	AT5G18400	Unknown	-2.96	-2.52
uncharacterized protein	AT4G20170	Unknown	-2.96	-3.32
hydrolase, alpha/beta fold family protein	AT5G02970	Unknown	-2.97	-2.81
uncharacterized protein	AT3G13437	Unknown	-2.99	-
conserved peptide upstream open reading frame 32	AT1G73602	Unknown	-3.01	-2.64
Stress responsive alpha-beta barrel domain protein	AT2G32500	Unknown	-3.03	-2.78
uncharacterized protein	AT3G14190	Unknown	-3.04	-
uncharacterized protein	AT1G05540	Unknown	-3.06	-2.97
HXXXD-type acyl-transferase-like protein	AT3G23840	Unknown	-3.07	-
uncharacterized protein	AT5G10750	Unknown	-3.09	-4.46
formin-like protein 7	AT1G59910	Unknown	-3.09	-3.33
uncharacterized protein	AT1G69430	Unknown	-3.11	-3.70
uncharacterized protein	AT5G44010	Unknown	-3.16	-3.46
PLAC8 family protein	AT1G11380	Unknown	-3.17	-2.70
O-Glycosyl hydrolases family 17 protein	AT5G55180	Unknown	-3.17	-2.64

uncharacterized protein	AT3G46110	Unknown	-3.19	-
uncharacterized protein	AT1G29980	Unknown	-3.20	-3.54
uncharacterized protein	AT3G46880	Unknown	-3.21	-
for hypothetical protein, clone: RAFL21-49-A06	AT5G36002	Unknown	-3.28	-5.14
early nodulin-like protein 15	AT4G31840	Unknown	-3.30	-
CASP-like protein	AT2G38480	Unknown	-3.33	-3.78
uncharacterized protein	AT4G18070	Unknown	-3.35	-3.37
O-Glycosyl hydrolases family 17 protein	AT3G07320	Unknown	-3.38	-2.74
uncharacterized protein	AT2G24330	Unknown	-3.40	-3.52
uncharacterized protein	AT1G01130	Unknown	-3.44	-4.79
uncharacterized protein	AT1G20070	Unknown	-3.44	-
uncharacterized protein	AT5G03390	Unknown	-3.45	-3.05
cysteine/histidine-rich C1 domain-containing protein	AT4G02540	Unknown	-3.45	-3.63
pollen Ole e 1 allergen and extensin family protein	AT3G26960	Unknown	-3.46	-3.96
FAD-binding and BBE domain-containing protein	AT4G20820	Unknown	-3.47	-3.55
uncharacterized protein	AT3G07470	Unknown	-3.49	-3.44
uncharacterized protein	AT3G63160	Unknown	-3.49	-
hydrolase	AT1G52510	Unknown	-3.57	-3.18
uncharacterized protein	AT5G57770	Unknown	-3.59	-4.84
uncharacterized protein	AT4G17240	Unknown	-3.60	-3.11
uncharacterized protein	AT5G27290	Unknown	-3.63	-3.01
C1 domain-containing protein	AT5G03360	Unknown	-3.64	-5.56
for hypothetical protein, clone: RAFL09-28-L17	AT1G79245	Unknown	-3.72	-3.66
O-fucosyltransferase family protein	AT2G44500	Unknown	-3.72	-4.88
Galactose-binding protein	AT1G22882	Unknown	-3.76	-3.83
Rossmann-fold NAD(P)-binding domain-containing protein	AT2G37540	Unknown	-3.76	-3.32
Exostosin family protein	AT4G32790	Unknown	-3.85	-4.27
early nodulin-like protein 13	AT5G25090	Unknown	-3.87	-
uncharacterized protein	AT3G49720	Unknown	-3.97	-3.94
uncharacterized protein	AT3G11420	Unknown	-4.00	-4.29
uncharacterized protein	AT2G35658	Unknown	-4.05	-3.70
uncharacterized protein	AT3G61920	Unknown	-4.09	-6.60
cysteine/histidine-rich C1 domain-containing protein	AT2G23100	Unknown	-4.09	-3.86
cytochrome P450, family 96, subfamily A, polypeptide 4	AT5G52320	Unknown	-4.15	-4.06
major facilitator protein	AT4G27720	Unknown	-4.17	-5.15
uncharacterized protein	AT1G18060	Unknown	-4.18	-3.30
alpha carbonic anhydrase 1	AT3G52720	Unknown	-4.18	-3.62
uncharacterized protein	AT2G12400	Unknown	-4.36	-4.50
Unknown	AT1G74330	Unknown	-4.44	-4.16
uncharacterized protein	AT1G61667	Unknown	-4.48	-4.91
uncharacterized protein	AT4G25830	Unknown	-4.50	-5.70
uncharacterized protein	AT1G79245	Unknown	-4.53	-4.42
uncharacterized protein	AT3G11420	Unknown	-4.56	-5.43
uncharacterized protein	AT2G27402	Unknown	-4.57	-5.62
uncharacterized protein	AT4G29780	Unknown	-4.76	-13.95
regulator of Vps4 activity protein	AT4G32350	Unknown	-4.95	-4.71
ATP sulfurylase	AT4G14680	Unknown	-5.08	-4.31
Actin cross-linking protein	AT1G69900	Unknown	-5.11	-5.06
uncharacterized protein	AT4G39840	Unknown	-5.25	-4.87
Regulator of Vps4 activity in the MVB pathway protein	AT4G35730	Unknown	-5.31	-5.64
NADPH--cytochrome P450 reductase 2	AT4G30210	Unknown	-5.36	-5.84
Heavy metal transport/detoxification superfamily protein	AT5G05365	Unknown	-5.37	-6.45
uncharacterized protein	AT1G06475	Unknown	-5.47	-6.34
gb|AYBHY07TR pooled cDNA populations Arabidopsis thaliana cDNA, sequence	AT1G79245	Unknown	-5.50	-6.29
uncharacterized protein	AT3G06070	Unknown	-5.58	-6.01
Hs1pro-1 protein	AT3G55840	Unknown	-5.68	-8.34
VQ motif-containing protein	AT2G22880	Unknown	-5.85	-6.60
uncharacterized protein	AT2G34510	Unknown	-5.88	-8.33
uncharacterized protein	AT2G44230	Unknown	-5.94	-8.49
beta-glucosidase 47	AT4G21760	Unknown	-6.01	-5.08
uncharacterized protein	AT2G33850	Unknown	-6.07	-
uncharacterized protein	AT5G25240	Unknown	-6.17	-4.68
UDP-glycosyltransferase-like protein	AT3G46650	Unknown	-6.27	-
hydroxyproline-rich glycoprotein family protein	AT1G72790	Unknown	-6.36	-9.15
uncharacterized protein	AT5G36920	Unknown	-6.59	-4.74
uncharacterized protein	AT1G56660	Unknown	-6.64	-7.12
uncharacterized protein	AT1G16850	Unknown	-7.19	-12.31
uncharacterized protein	AT3G14870	Unknown	-7.25	-8.06
O-methyltransferase family protein	AT1G21110	Unknown	-7.30	-
gb|016G06 Infected Arabidopsis Leaf Arabidopsis thaliana cDNA, sequence	AT1G79245	Unknown	-7.31	-6.91
uncharacterized protein	AT2G32200	Unknown	-7.31	-6.34

late embryogenesis abundant hydroxyproline-rich glycoprotein	AT2G27080	Unknown	-7.51	-12.71
uncharacterized protein	AT1G19380	Unknown	-7.60	-6.47
uncharacterized protein	AT2G36145	Unknown	-7.65	-6.19
uncharacterized protein	AT3G19680	Unknown	-7.91	-7.58
uncharacterized protein	AT4G04745	Unknown	-8.31	-
O-fucosyltransferase family protein	AT3G05320	Unknown	-8.33	-7.55
uncharacterized protein	AT2G20835	Unknown	-8.64	-8.29
Late embryogenesis abundant protein, group 6	AT2G23120	Unknown	-9.16	-12.09
copper amine oxidase	AT1G31690	Unknown	-9.23	-6.17
uncharacterized protein	AT5G03120	Unknown	-9.59	-12.70
uncharacterized protein	AT2G23690	Unknown	-9.92	-9.77
hydrolase, alpha/beta fold family protein	AT3G24420	Unknown	-10.98	-
uncharacterized protein	AT5G28610	Unknown	-11.61	-11.36
uncharacterized protein	AT4G37240	Unknown	-13.24	-12.06
uncharacterized protein	AT5G57760	Unknown	-15.84	-19.10
uncharacterized protein	AT1G50040	Unknown	-18.03	-25.76
uncharacterized protein	AT2G35290	Unknown	-22.34	-37.10
Unknown	AT5G55420	Unknown	-	10.33
uncharacterized protein	AT1G13470	Unknown	-	10.11
DUF679 domain membrane protein 1	AT3G21520	Unknown	-	7.64
FAD-binding and BBE domain-containing protein	AT1G26380	Unknown	-	6.72
uncharacterized protein	AT5G41761	Unknown	-	6.03
microRNA miR163 type 1 precursor, complete sequence; alternatively spliced	AT1G66725	Unknown	-	5.73
cysteine/histidine-rich C1 domain-containing protein	AT2G44370	Unknown	-	4.99
cytochrome P450 71B36	AT3G26320	Unknown	-	3.91
uncharacterized protein	AT1G22890	Unknown	-	3.90
uncharacterized protein	AT5G50660	Unknown	-	3.85
UDP-glycosyltransferase 87A2	AT2G30140	Unknown	-	3.85
uncharacterized protein	AT5G12930	Unknown	-	3.68
SOUL heme-binding protein	AT1G78460	Unknown	-	3.63
uncharacterized protein	AT1G16500	Unknown	-	3.53
uncharacterized protein	AT2G17710	Unknown	-	3.52
uncharacterized protein	AT1G67920	Unknown	-	3.44
cysteine/histidine-rich C1 domain-containing protein	AT2G27660	Unknown	-	3.31
NAD(P)-binding Rossmann-fold superfamily protein	AT3G01980	Unknown	-	3.28
uncharacterized protein	AT4G26990	Unknown	-	3.23
late embryogenesis abundant hydroxyproline-rich glycoprotein	AT4G23610	Unknown	-	3.06
uncharacterized protein	AT1G07090	Unknown	-	3.03
uncharacterized protein	AT1G17744	Unknown	-	3.03
PB1_UP2 domain-containing protein	AT3G26510	Unknown	-	2.96
uncharacterized protein	AT1G70420	Unknown	-	2.91
Trm112p-like protein	AT1G78190	Unknown	-	2.90
uncharacterized protein	AT3G61280	Unknown	-	2.64
carboxyesterase 20	AT5G62180	Unknown	-	2.63
uncharacterized protein	AT1G24145	Unknown	-	2.58
Dihydrolipoamide succinyltransferase	AT4G26910	Unknown	-	2.57
Flotillin-like protein 1	AT5G25250	Unknown	-	2.52
uncharacterized protein	AT4G33980	Unknown	-	2.51
pseudogene of disease resistance protein	AT3G03855	Unknown	-	2.50
uncharacterized protein	AT1G12320	Unknown	-	2.39
tc|Rep: Adagio protein 3 - Arabidopsis thaliana (Mouse-ear cress), partial (10%)	AT5G23410	Unknown	-	2.35
uncharacterized protein	AT3G22240	Unknown	-	2.29
uncharacterized protein	AT3G48640	Unknown	-	2.27
uncharacterized protein	AT3G22235	Unknown	-	2.25
prolyl-4 hydroxylase 2	AT3G06300	Unknown	-	2.25
CCT motif family protein	AT4G27900	Unknown	-	2.24
Phenazine biosynthesis PhzC/PhzF protein	AT1G03210	Unknown	-	2.19
uncharacterized protein	AT1G75810	Unknown	-	2.17
amidase family protein	AT5G07360	Unknown	-	2.17
kinectin-related protein	AT5G66250	Unknown	-	2.17
tetratricopeptide repeat-containing protein-like protein	AT1G07280	Unknown	-	2.16
gb|AYBKB64TR pooled cDNA populations Arabidopsis thaliana cDNA, sequence	AT1G34315	Unknown	-	2.15
uncharacterized protein	AT2G03310	Unknown	-	2.10
uncharacterized protein	AT1G62840	Unknown	-	2.04
uncharacterized protein	AT1G53035	Unknown	-	2.04
uncharacterized protein	AT5G45470	Unknown	-	2.02
uncharacterized protein	AT1G22250	Unknown	-	2.01
uncharacterized protein	AT1G14770	Unknown	-	-1.99
uncharacterized protein	AT4G28590	Unknown	-	-2.03
Restriction endonuclease, type II-like protein	AT1G67660	Unknown	-	-2.03
Patched family protein	AT1G42470	Unknown	-	-2.05

uncharacterized protein	AT4G39900	Unknown	-	-2.06
thylakoid lumenal protein-17.4	AT5G53490	Unknown	-	-2.09
tetrapyrrole (corrin/porphyrin)methylase	AT1G45110	Unknown	-	-2.10
DNAJ heat shock N-terminal domain-containing protein	AT2G17880	Unknown	-	-2.13
uncharacterized protein	AT2G17300	Unknown	-	-2.14
uncharacterized protein	AT5G19260	Unknown	-	-2.16
Uroporphyrinogen-III synthase	AT2G26540	Unknown	-	-2.17
uncharacterized protein	AT3G17330	Unknown	-	-2.18
transacting siRNA generating locus	AT1G63130	Unknown	-	-2.19
uncharacterized protein	AT5G13100	Unknown	-	-2.19
uncharacterized protein	AT1G57680	Unknown	-	-2.21
tc|Rep: F22C12.9 - Arabidopsis thaliana (Mouse-ear cress), partial (56%)	AT1G64150	Unknown	-	-2.22
uncharacterized protein	AT5G22340	Unknown	-	-2.26
uncharacterized protein	AT3G47510	Unknown	-	-2.27
uncharacterized protein	AT2G35470	Unknown	-	-2.27
uncharacterized protein	AT1G49840	Unknown	-	-2.29
uncharacterized protein	AT3G08030	Unknown	-	-2.40
uncharacterized protein	AT5G26770	Unknown	-	-2.40
uncharacterized protein	AT3G50120	Unknown	-	-2.46
cytochrome c biogenesis protein CCS1	AT1G49380	Unknown	-	-2.47
P-loop containing nucleoside triphosphate hydrolases superfamily protein	AT5G52882	Unknown	-	-2.50
NHL domain-containing protein	AT1G70280	Unknown	-	-2.53
uncharacterized protein	AT2G29180	Unknown	-	-2.57
tropinone reductase-like protein	AT2G29300	Unknown	-	-2.58
uncharacterized protein	AT1G16170	Unknown	-	-2.64
protein PHLOEM protein 2-LIKE A2	AT4G19850	Unknown	-	-2.70
uncharacterized protein	AT5G46220	Unknown	-	-2.75
protein MID1-complementing activity 2	AT2G17780	Unknown	-	-2.86
uncharacterized protein	AT3G56810	Unknown	-	-2.87
uncharacterized protein	AT1G02380	Unknown	-	-2.88
uncharacterized protein	AT3G06435	Unknown	-	-2.95
uncharacterized protein	AT5G37550	Unknown	-	-2.96
early nodulin-like protein 2	AT4G27520	Unknown	-	-2.98
uncharacterized protein	AT1G18740	Unknown	-	-3.03
uncharacterized protein	AT5G66800	Unknown	-	-3.07
uncharacterized protein	AT2G39650	Unknown	-	-3.15
uncharacterized protein	AT3G01430	Unknown	-	-3.38
uncharacterized protein	AT5G67370	Unknown	-	-3.48
armadillo/beta-catenin-like repeats-containing protein	AT2G05810	Unknown	-	-3.53
early nodulin-like protein 18	AT1G08500	Unknown	-	-3.57
uncharacterized protein	AT1G09575	Unknown	-	-3.67
COBRA-like protein 8	AT3G16860	Unknown	-	-3.73
uncharacterized protein	AT3G54000	Unknown	-	-3.78
uncharacterized protein	AT3G06890	Unknown	-	-3.96
uncharacterized protein	AT1G80120	Unknown	-	-4.98
uncharacterized protein	AT5G17350	Unknown	-	-5.15
uncharacterized protein	AT4G02170	Unknown	-	-5.40
uncharacterized protein	AT1G22470	Unknown	-	-5.74
uncharacterized protein	AT2G01300	Unknown	-	-7.15
uncharacterized protein	AT1G74450	Unknown	-	-7.63
uncharacterized protein	AT5G56980	Unknown	-	-9.63
uncharacterized protein	AT5G38700	Unknown	-	-9.79

Supplementary Table S6. Sequences of oligonucleotides used

Oligo ID	*Accesion number*		Sequence (5'-3')
**For expression analysis**			
β-Tubulin 2	*At5g62690*	*Frw*	TGTTCAGGCGAGTGAGTGAG
		Rev	ATGTTGCTCTCCGCTTCTGT
Ubiquitin21	*At5g25760*	*Frw*	AAAGGACCTTCGGAGACTCCTTACG
		Rev	GGTCAAGAAtCGAACTTGAGGAGGTT
P.cucumerina _tubulin	-	*Frw*	CAAGTAtGTTCCCCGAGCCGT
		Rev	GAAGAGCTGACCGAAGGGACC
B.cinerea _tubulin	-	*Frw*	TTCCAtGAAGGAGGTTGAGG
		Rev	TACCAACGAAGGTGGAGGAC
FOC_chsV	-	*Frw*	ACAGCTCCAACGAACTCTCTT
		Rev	GGAGGTACTTGGTCAtGTCGT
Ch_ITS2	-	*Frw*	AAAGGTAGTGGCGGACCCTC
		Rev	GGCAAGAGTCCCTCCGGAt
miR396	-	*Frw*	GCGCGGTTCCACAGCTTTCT
		Rev	GTGCAGGGTCCGAGGT
miR396a Stem loop			GTCGTAtCCAGTGCAGGGTCCGAGGTAtTCGCACTGGAtACGACCAGTTCA
miR396b Stem loop			GTCGTAtCCAGTGCAGGGTCCGAGGTAtTCGCACTGGAtACGACAAGTTCA
Pre-miR396a	*At2g10606*	*Frw*	GCTTTCTTGAACTGCAAAACTT
		Rev	TCAAtCGAGCAGAGAtAtGAAGA
Pre-miR396b	*At5g35407*	*Frw*	TTGAACTTTCTTTTTCAtTTCCA
		Rev	CAGCTTTCTTGAGCTTCCAAA
GRF1	*At2g22840*	*Frw*	CGCTGTTCCCGAtCAAAAGTA
		Rev	CAGCAtTAGTAtTGTGGCCAtT
GRF2	*At4g37740*	*Frw*	ACAtCAACAGAGGCCGTCAt
		Rev	TTGTGGTGTAGTAACCGCTTTG
GRF3	*At2g36400*	*Frw*	GAAGTGGAGAtGTTCAAGAGACG
		Rev	GTTGGAGTTTCCACAGGCTTT
GRF4	*At3g52910*	*Frw*	CACAtTCACCGTGGAAGAAAC
		Rev	ACCAGAGGAGAAGAAGTGGTTG
GRF7	*At5g53660*	*Frw*	CGCGAAAGAAGTCGTCTCTAAt
		Rev	GTTAGGGCGAGAAtAAGGAGGT
GRF8	*At4g24150*	*Frw*	GAACAGAtGGGAAGAAAtGGAG
		Rev	GGTGAGAtGAtTGGTGAGAtGA
GRF9	*At2g45480*	*Frw*	AACACGGTCCTTCTAtTCGAGA
		Rev	TGAtGAAGCAACCTCAGAAGAA
bHLH74	*At1g10120*	*Frw*	TTTCAGAtGAtGCTGTTCTTGG
		Rev	CTCTCCTTGTTCACCGTCTCTT
Thioredoxin	*At5g38900*	*Frw*	ACACCGCTTCAAAAAAGCTCA
		Rev	CACCATGGACACACCGAATC
GST 6	*At1g02930*	*Frw*	GCAAGGACATGGCGATCATA
		Rev	ACTGGGTCAAACTCATGCGAC
GST F2	*At4g02520*	*Frw*	CGCCCTCCACGAGAAAAAC
		Rev	GCTCACCGTCTTTGAGTTCGA
APX1	*At1g07890*	*Frw*	ACACTCTGGGACGATGCCAC
		Rev	GGGTTTGATGTCCATGCACC
CAT3	*At1g20620*	*Frw*	CGTTGAAACCTAACCCGAAA
		Rev	AAACATCCAGCACCATGTGA
Aldehyde oxidase 1	*At5g20960*	*Frw*	CGGAAAATTGTCTATACGGCG
		Rev	CCCTTAATTCGCGCTAGCG
Aldo-ketoreductase 5	*At1g60730*	*Frw*	CTGTTCACCCGATAACCGCT
		Rev	TCTTCCACGTCTCTCGACCAT
R gene	*At4g11170*	*Frw*	TATGCGTGGAAAGGGTAAGG
		Rev	CAAGCCATCATACCCGACTT
LRR receptor kinase	*At1g74360*	*Frw*	AGTATGGGAACATGCCGGG
		Rev	CGAACCGGTCAGCTTGTTAAA
R gene (TIR-NBS-LRR)	*At5g45000*	*Frw*	CAACACGTCGCCCACAAGT
		Rev	ATAAAGTTGTCGCCAAGCTCG
EDS1B	*At3g48080*	*Frw*	GGAGCCGGTTTTTCGTAAACT
		Rev	GCAAGCATAATCCGAGGGAC
PAD4	*At3g52430*	*Frw*	CGAATACATTGGTGACGAAGAA
		Rev	ACCCATTTTGCACTTGAACTCT
PDF1.2	*At2g26020*	*Frw*	CAACAATGGTGGAAGCACAG
		Rev	CTTGCATGCATTGCTGTTTC
PR4	*AT3G04720*	*Frw*	TGTGAGAATAGTGGACCAATGC
		Rev	CCATCGGTGTCTATTTGATTGA
VSP2	*At5g24770*	*Frw*	CTCGTCGATTCGAAAACCAT
		Rev	TTCTGCAGTTGGCGTAGTTG
PR-1	*AT2G14610*	*Frw*	GATGTGCCAAAGTGAGGTGTAA
		Rev	GGCTTCTCGTTCACATAATTCC
NPR1	*At1g64280*	*Frw*	CCGGAAGAGCTTGTTAAAGAGA
		Rev	ATCCGAGTCAAGTGCCTTATGT
**For Construct preparation**			
MIR396B	*At5g35407*		CGCGGTACCGGTCCTCTTGAGTTTCTTCTATATGC
			CGCTCTAGAtGGAAAGAAGAAtCTTGACAAGTG
